# Automatic validation of computational models using pseudo-3D spatio-temporal model checking

**DOI:** 10.1186/s12918-014-0124-0

**Published:** 2014-12-02

**Authors:** Ovidiu Pârvu, David Gilbert

**Affiliations:** Department of Computer Science, Brunel University, Kingston Lane, Uxbridge, UB8 3PH London UK

**Keywords:** Stochastic spatial discrete event system (SSpDES), Probabilistic bounded linear spatial temporal logic (PBLSTL), Spatio-temporal, Multidimensional, Model checking, Mudi, Computational model, Model validation, Systems biology, Synthetic biology

## Abstract

**Background:**

Computational models play an increasingly important role in systems biology for generating predictions and in synthetic biology as executable prototypes/designs. For real life (clinical) applications there is a need to scale up and build more complex spatio-temporal multiscale models; these could enable investigating how changes at small scales reflect at large scales and viceversa. Results generated by computational models can be applied to real life applications only if the models have been validated first. Traditional *in silico* model checking techniques only capture how non-dimensional properties (e.g. concentrations) evolve over time and are suitable for small scale systems (e.g. metabolic pathways). The validation of larger scale systems (e.g. multicellular populations) additionally requires capturing how spatial patterns and their properties change over time, which are not considered by traditional non-spatial approaches.

**Results:**

We developed and implemented a methodology for the automatic validation of computational models with respect to both their spatial and temporal properties. Stochastic biological systems are represented by abstract models which assume a linear structure of time and a pseudo-3D representation of space (2D space plus a density measure). Time series data generated by such models is provided as input to parameterised image processing modules which automatically detect and analyse spatial patterns (e.g. cell) and clusters of such patterns (e.g. cellular population). For capturing how spatial and numeric properties change over time the Probabilistic Bounded Linear Spatial Temporal Logic is introduced. Given a collection of time series data and a formal spatio-temporal specification the model checker Mudi (http://mudi.modelchecking.org) determines probabilistically if the formal specification holds for the computational model or not. Mudi is an approximate probabilistic model checking platform which enables users to choose between frequentist and Bayesian, estimate and statistical hypothesis testing based validation approaches. We illustrate the expressivity and efficiency of our approach based on two biological case studies namely phase variation patterning in bacterial colony growth and the chemotactic aggregation of cells.

**Conclusions:**

The formal methodology implemented in Mudi enables the validation of computational models against spatio-temporal logic properties and is a precursor to the development and validation of more complex multidimensional and multiscale models.

**Electronic supplementary material:**

The online version of this article (doi:10.1186/s12918-014-0124-0) contains supplementary material, which is available to authorized users.

## Background

### Introduction

Computational modelling is a key element of both systems [1,2] and synthetic [3,4] biology research. In systems biology models are constructed from biological observations and are used to generate predictions of system behaviour under various conditions. Conversely in synthetic biology models are employed as executable prototypes/designs for engineering useful synthetic biological systems.

One of the major limitations of the existing models is that they are restricted to small scale biological subsystems [5]. For real life application areas such as medicine or biotechnology there is a need to scale up and build more complex multiscale models which cover multiple spatial and/or temporal scales [6,7]; the Virtual Physiological Human [8] and High-Definition Physiology [9] projects are international initiatives attempting to (partially) address this challenge.

Results generated through computational model simulation can be used for real life applications only if the model has been validated first. Traditionally this has been done by comparing time series data generated by models with biological observations recorded in the wet lab. If significant inconsistencies are detected the model needs to be updated and/or the experiments have to be repeated which is both expensive and time consuming. In an attempt to detect modelling errors as soon as possible *in silico* formal validation methods are additionally employed [10]. One of the most employed model validation methods in systems and synthetic biology is model checking.

In this paper we present a model checking methodology for the validation of multidimensional (spatio-temporal) computational models, and its application to two multicellular population based examples from systems biology.

### Model checking

Model checking [11,12] is a validation method which automatically verifies if a model of a system is correct according to a given formal specification. The general model checking steps are:

1. **Modelling**: Creating an abstract representation of the system (e.g. a computational model);
2. **Specification**: Encoding the formal specification of the system.
3. **Verification**: Automatically validating the model against the specification.

Models which are validated using model checking approaches are dynamic i.e. they can be simulated in order to generate timeseries data which illustrate how they change over time.

System specifications are usually encoded using formal languages due to their rigorous syntax and semantics. Traditionally in model checking the system specification is formalised using a class of formal languages called temporal logics because they enable reasoning about how the state of the system changes over time.

Linear time temporal logics assume the structure of time to be linear which means that at each moment in time a system state has at most one possible successor state [13,14]. The first temporal formalism considering a linear time structure used for model checking (concurrent systems) was Linear Temporal Logic (LTL) [15,16].

Logic statements written in LTL are composed of atomic, Boolean and temporal logic propositions.

An atomic proposition is a statement which evaluates to true/false and cannot be divided into simpler logic statements. For biological systems specifications the set of atomic properties usually comprises (but is not limited to) arithmetic expressions of the form {*A*}≍*r*, where {*A*} denotes the concentration of species/protein *A*, ≍ ∈{<,<=,=,>=,>} and $r \in \mathbb {R}$. In addition {*A*} can be prefixed with the difference/differential operator *d* such that *d*({*A*}) represents the rate of change for concentration {*A*} from the current to the next timepoint.

Conversely a Boolean proposition is a compound statement comprising a Boolean operator and logic proposition(s) (denoted here by *ϕ*):

- ¬*ϕ* (not): The **negation** of logic proposition *ϕ* is true i.e. *ϕ* is false.
- *ϕ*_1_∧*ϕ*_2_ (and): logic proposition *ϕ*_1_ is true **and** logic proposition *ϕ*_2_ is true.
- *ϕ*_1_∨*ϕ*_2_ (or): logic proposition *ϕ*_1_ is true **or** logic proposition *ϕ*_2_ is true.
- *ϕ*_1_⇒*ϕ*_2_ (implication): logic proposition *ϕ*_1_ is true **implies** logic proposition *ϕ*_2_ is true.
- *ϕ*_1_⇔*ϕ*_2_ (equivalence): logic proposition *ϕ*_1_ is true **equivalent to** logic proposition *ϕ*_2_ is true.

where ¬ is a unary Boolean operator, and ∧,∨,⇒,⇔ are binary Boolean operators.

Finally temporal propositions are used to reason about how the system changes over time. They comprise a temporal operator and logic proposition(s):

- *Fϕ* (**F**uture): Eventually logic proposition *ϕ* holds.
- *Gϕ* (**G**lobally): Logic proposition *ϕ* holds always.
- *ϕ*_1_*Uϕ*_2_ (**U**ntil): Logic proposition *ϕ*_1_ holds until logic proposition *ϕ*_2_ holds.
- *Xϕ* (ne**X**t): Logic proposition *ϕ* holds in the next time point.

where *F*, *G*, *U*, *X* are temporal operators.

Bounded Linear Temporal Logic (BLTL) [17,18] is an extension of LTL where a bounded time interval is associated to the temporal operators:

- *F*[ *a*,*b*] *ϕ*: Eventually logic proposition *ϕ* holds within the time interval [ *a*,*b*].
- *G*[ *a*,*b*] *ϕ*: Logic proposition *ϕ* holds always within the time interval [ *a*,*b*].
- *ϕ*_1_*U*[ *a*,*b*] *ϕ*_2_: Logic proposition *ϕ*_1_ holds until logic proposition *ϕ*_2_ holds within the time interval [ *a*,*b*].
- *X*[ *k*] *ϕ*: Logic proposition *ϕ* holds in the next *k*-th timepoint.

The advantage of employing BLTL instead of LTL is that only a bounded simulation time interval has to be considered for the evaluation of the temporal logic propositions.

Considering a model and formal BLTL specification a software called “model checker” automatically verifies if the model is valid or not with respect to the specification.

### Model checking in systems and synthetic biology

In systems biology model checking has been proposed as a methodology for validation [19,20] and parameter synthesis/estimation [21–24], respectively in synthetic biology for efficient design [25,26] and *in silico* validation [27].

Probabilistic model checking approaches are employed for the formal validation of systems which exhibit stochastic behaviour and can be either exhaustive or approximate. Exhaustive approaches potentially explore the entire state space (i.e. all possible system states) to decide if a model is valid and are therefore highly accurate. The disadvantage is that their complexity increases with the size of the state space which means they are not scalable. Conversely approximate probabilistic approaches decide if a model is valid based on methods from statistical theory using only a finite set of simulations. Therefore they are scalable because the state space is only partially explored. Although such approaches provide an answer based on an approximation, which is not guaranteed to be correct, the user can place an upper bound on the approximation error.

Due to the stochastic nature, high complexity and multiscale representation of biological systems approximate probabilistic model checking approaches are preferred both for systems and synthetic biology applications; an extensive review of relevant model checking approaches is provided in [28], respectively a review of statistical model checking methods employed in systems biology is provided in [29]. Two known model checkers which support both exhaustive and approximate approaches are MARCIE [30] and PRISM [31].

Traditional model checking approaches in systems and synthetic biology only capture how non-dimensional properties (e.g. concentrations) evolve over time, and are appropriate for small scale systems such as signalling/ metabolic pathway models. The evolution over time of spatial patterns and their properties has to be additionally considered when building computational models of more complex systems (e.g. multicellular organisms or populations of microorganisms). Such multidimensional (i.e. spatio-temporal) properties cannot be captured by the traditional non-spatial approach.

### Contributions

**Figure 1:**
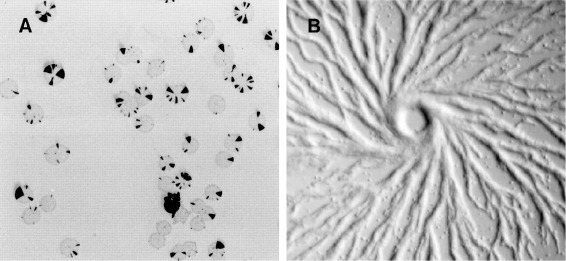
**Real-life images for considered case studies.** **(A)** Multiple bacterial colonies with phase variable genes. Sector-like patterns (highlighted in black) are an indication of high proportions of “mutant” cells (i.e. cells with switched gene expression). Conversely gray parts of the colony are an indication of high proportions of wild-type cells. (Reproduced with permission from [32]. Copyright Ⓒ 1998, American Society for Microbiology). **(B)** Population of *Dictyostelium discoideum* cells chemotactically aggregating in the centre where the chemical attractant concentration is highest (Reproduced with permission from [33]).

Since one of the main aims of systems and synthetic biology is to scale up the development of computational models corresponding formal validation methods need to be in place. In this paper we attempt to address this challenge by developing and implementing a methodology for automatic spatio-temporal model validation. Due to the high complexity inherent to spatial computational model only approximate probabilistic model checking approaches will be considered throughout. In the interdisciplinary spirit of computational biology research this paper covers aspects ranging from theory development to software implementation and application to biological case studies. Our main contributions are:

- The definition of a stochastic spatial discrete-event system (SSpDES) as an abstract representation for describing how stochastic biological systems evolve in time and space;
- A formal Probabilistic Bounded Linear Spatial Temporal Logic (PBLSTL) for specifying spatio-temporal logic statements;
- The implementation of the methodology in the multidimensional model checking platform Mudi which enables validating spatio-temporal models against PBLSTL properties. Mudi comprises both Bayesian and frequentist, estimate and hypothesis testing based validation approaches.
- Parameterised image processing algorithms for detecting and analysing spatial patterns and clusters of such patterns in time series data;
- The Spatial Temporal Markup Language (STML) for representing spatio-temporal properties extracted from time series data;

Our methodology was validated against two biological case studies namely phase variation patterning in bacterial colony growth and the chemotactic aggregation of cells; see Figure 1 for illustrative real-life images.

The contents of this paper are organised as follows. All theoretical computer science and software implementation details are provided in the “Methods” section, while the application of the methodology to two biological case studies is described in the section “Results”. The interpretation of the results, limitations, future work and a comparison to related work are provided in the “Discussion” section. Finally a summary of our contribution is provided in the section “Conclusions”.

## Methods

**Figure 2:**
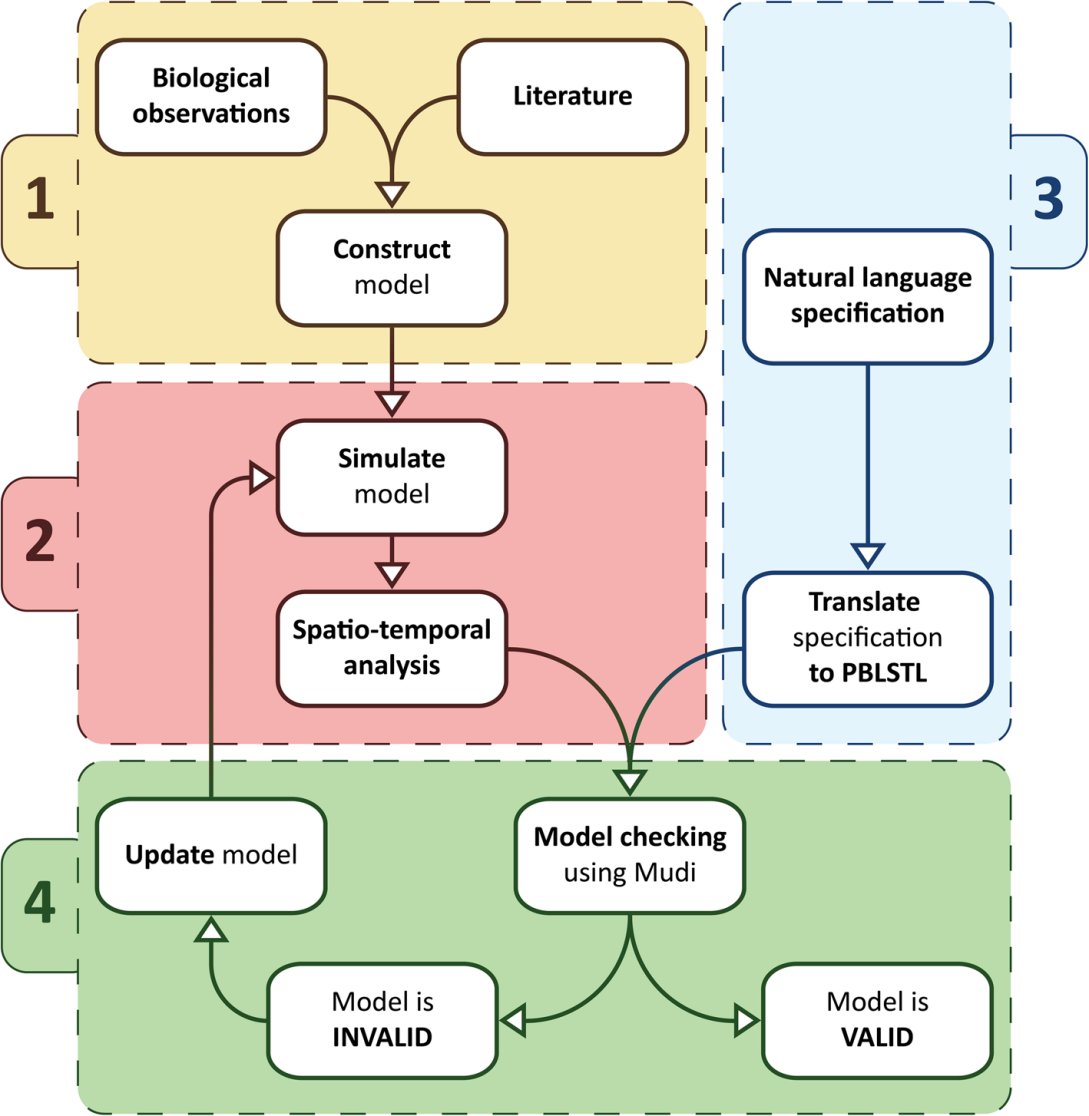
**Spatio-temporal model validation workflow.** Workflow comprising all steps from model construction to model validation. The first step (1) describes the construction of the model from biological observations and/or literature. In the second step (2) the model is simulated to generate time series data which is passed to the spatio-temporal analysis module for automatic detection and analysis of spatial patterns and clusters of such patterns. The third step (3) comprises the translation of the natural language specification of the system to a formal probabilistic BLSTL (PBLSTL) specification. Finally the fourth step (4) describes the validation of the model with respect to the PBLSTL specification using the model checker Mudi. In case the model is invalid it is updated and the validation procedure repeated.

The general workflow for spatio-temporal model construction and validation is depicted in Figure 2 and comprises the following steps:

1. **Model construction.** Building the computational model from biological observations and/or relevant references from the literature.
2. **Spatio-temporal analysis.** The model is simulated to generate time series data in which spatial patterns and clusters of such patterns are automatically detected and analysed. The output of the spatio-temporal analysis is formatted according to the STML standard specification.
3. **Formal specification.** Natural language properties representing the specification of the system are translated to formal PBLSTL statements.
4. **Model checking.** The model checker Mudi takes the spatio-temporal analysis output and the PBLSTL statements as input and decides if the model is valid or not using the validation method chosen by the user (e.g. frequentist statistical model checking). In case the model is invalid it is updated and then checked again.

### Model construction

Biological systems are usually modelled as stochastic processes which transition from the current state to the successor state when an event occurs (e.g. a biochemical reaction). This specific type of stochastic process is called a stochastic discrete-event system (SDES).

#### **Definition1**.

The factored representation of an SDES  (see [34], Chapter 2) is a 5-tuple 〈*S*, *T*, *μ*, *SV*, *V*〉 where:

- *S* is the set of all possible *states* of the system;
- *T* is the *transition rates* matrix which records the probability of the system to transition from the current state *s*_*i*_ to the next state *s*_*j*_, ∀*s*_*i*_,*s*_*j*_∈*S*;
- *μ* is a *probability measure* computing the probability of the system to reach a certain state along the sequences of states described by a set of simulation traces;
- *SV* is the set of *state variables* describing the state of the system;
- *V* is the *value assignment function* which computes the value ${\in \mathbb {R}}$ of each state variable for a given simulation trace and state of the system.

Our aim is to additionally reason about properties of spatial patterns in such systems, and to quantify how these properties change over time. The following assumptions are made regarding the representation of space:

1. Only the *discretised* version of the 2D, respectively pseudo-3D Euclidean space is considered. A pseudo-3D space extends a 2D Euclidean space with a density measure for each position. The density measure indicates the proportion of occupied positions on the Oz axis for a fixed (x, y) position. Compared to a full 3D representation it does not specify explicitly which positions of the Oz axis are occupied but only their proportion.
2. The 2D Euclidean space is discretised by splitting it into *m* rows and *n* columns obtaining an *m*×*n* regular grid where *m* and *n* are finite, natural, positive numbers. The resolution of the results depends on the values of *m* and *n*. Higher values guarantee a fine-grained resolution while lower values account for a coarse-grained resolution.

The evolution of an SDES in space could be represented using one/multiple collections of *m*·*n* state variables such that each state variable represents one discretised position in space. The main advantage of this is that the structure of SDES does not change when adding spatial information to a model. However the main disadvantage is that semantically different state variables (e.g. concentrations, value of discretised position in space) belong to the same set without the possibility to explicitly distinguish between them at the entire set level. In the following we would like to reason about how subsets of positions in the discretised space (e.g. representing subpopulations of cells) and their geometric properties (e.g. area) change over time. Therefore there is a need to define detection and analysis methods which are specific to the collection of state variables encoding space, and do not apply to state variables encoding numeric values such as concentrations. For this reason the state variables encoding spatial information will be extracted in a separate set denoted as spatial state variables (*SpSV*). Moreover instead of representing space using *m*·*n* spatial state variables such that the value of each state variable $\in \mathbb {R}$, a single spatial state variable whose value $\in \mathbb {R}_{+}^{m \times n}$ is employed. The evaluation of such state variables to *m*×*n* real-valued non-negative matrices cannot be performed by the existing value assignment function *V* whose codomain is . Thus a corresponding spatial value assignment function (*SpV*) is defined.

Considering the above notations we define stochastic spatial discrete-event systems (SSpDES) as an extension of SDES with a set of spatial state variables *SpSV* and a spatial value assignment function *SpV*.

#### **Definition2**.

An SSpDES  is a 7-tuple 〈*S*, *T*, *μ*, *NSV*, *SpSV*, *NV*, *SpV*〉 where:

- 〈*S*, *T*, *μ*, *NSV*, *NV*〉 is a SDES (see Definition 1);
- *SpSV* is the set of *spatial state variables*;
- *SpV* is the *spatial value assignment function*.

The set *SpSV* contains all spatial state variables i.e. the variables recording the configuration of the discretised space in the current system state. The value of these variables is computed using the spatial value assignment function *SpV*:

$$ {SpV}: E \times S \times {SpSV} \rightarrow \mathbb{R}_{+}^{m \times n} $$

where *E* denotes the set of all possible model executions/simulations, *S* the set of states, *SpSV* the set of spatial state variables, and *m* and *n* the dimensions of the discretised space. Given a model simulation *σ* at state *s* and a spatial state variable *ssv*, *SpV*(*σ*,*s*,*ssv*)=*sv* such that $sv \in \mathbb {R}_{+}^{m \times n}$ returns a *m*×*n* matrix of real non-negative values, where each element of the matrix corresponds to a position in the discretised space. For explanatory purposes an illustrative example of a simple SSpDES is provided in Additional file 1.

The size of the discretised space and the semantics of the values stored for each spatial compartment depends on the biological problem one tries to address. For instance space was discretised in 101×101 compartments for the phase variation case study because the sector-like patterns (see Figure 1A) should be easily recognizable. Employing a more coarse-grained spatial resolution would distort the shape of the sectors, respectively a more fine-grained resolution would lead to an increased model simulation time. The values recorded for each position of the discretised space are the number of wild-type, respectively “mutant” cells. Conversely in case of the chemotaxis case study the size of the discretised space was 100×100 with 1% of the spatial compartments occupied by cells. The reason for choosing this spatial resolution was to ensure that the formation of clusters is not an artifact of the inability of cells to move due to lack of space, but is a consequence of their chemotactic behaviour (see Figure 1B). In this case both number of cells and chemical attractant concentration were recorded for each position in the discretised space.

Finally one of the main advantages of defining SSpDESs as an extension of SDESs is backwards compatibility i.e. existing SDES models can be interpreted as SSpDESs having an empty set of spatial state variables *SpSV*. Moreover SSpDESs enable scaling up the development of computational models by extending existing non-spatial models, typical for subcellular scales (e.g. intracellular networks), with spatial information relevant to potentially higher scales (e.g. cellular/tissue level). For instance the computational model employed for the phase variation case study [35] is an illustrative example of constructing a spatial stochastic computational model from an initially non-spatial deterministic model [36].

### Spatio-temporal analysis

Simulations of an SSpDES (see Definition 2) provide timeseries data describing how each position of the discretised space changes over time. In order to reason about (clusters of) spatial patterns an automatic mechanism for detecting and analysing the relevant subsets of positions in the discretised space is required. Two parameterised mechanisms will be employed for automatically detecting subsets of the discretised space; one for spatial patterns denoted in the rest of the paper as *regions* and the other for *clusters*. Depending on the values of the detection parameters a more fine- or coarse-grained subset of the discretised space is considered.

#### Regions

One of the main assumptions of the region detection mechanism is that subsets and not individual positions of the discretised space are considered. Secondly the value of each position of the discretised space records the number/density of entities of interest. Each position can hold 0 or more entities without pileup. The identity of the elements forming the region is not relevant. It is assumed that the type and size of the entities is constant throughout the entire space. Therefore the region detection mechanism operates in a homogeneous context with respect to the type of modelled entities. In the case that the system comprises multiple types of entities, each type is represented by a different spatial variable. Therefore the regions defined by groups of different entities can be computed by repeatedly applying the region detection mechanism for each spatial variable.

Given a model execution/simulation *σ*, the *i*-th state *σ*[ *i*], 0≤*i*≤|*σ*|, where |*σ*| represents the length of *σ*, and a spatial state variable *ssv*, let *SP*=*SpV*(*σ*,*σ*[ *i*],*ssv*).

##### **Definition3**.

A **region***R* with respect to *σ*[ *i*] and *ssv* is a subset of neighbouring positions in *SP* such that ∀*x*∈*R*,*value*(*x*)≥*THRESHOLD* and |*R*|>*ε*_*size*_, where $THRESHOLD, \epsilon _{\textit {size}} \in \mathbb {R}$ are user-definedparameters.

**Figure 3:**
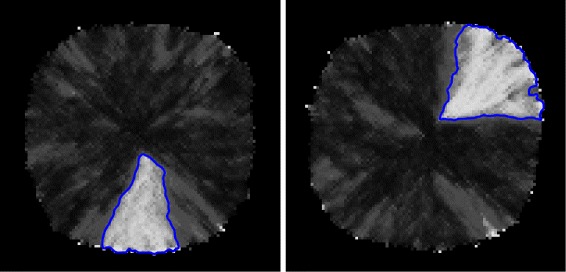
**Detection of sector-like patterns in bacterial colonies.** Grayscale images depicting the final state of two phase variation model simulations. Sector-like patterns corresponding to high-proportions of “mutant” cells are automatically detected and outlined in blue. Note that the colour scheme in these images is the inverse of the one in Figure 1A i.e. sector-like patterns are highlighted in white instead of black, respectively patches of wild-type cells are highlighted in black instead of white (gray). Moreover only one bacterial colony is depicted in each one of these images while in Figure 1A multiple bacterial colonies are shown.

The problem of finding regions is similar to the segmentation problem in the Computer Vision literature [37]. 2D images can be represented as vectors or matrices where each position records the colour (multi-channel) or intensity (single channel) of the image. In order to apply Computer Vision methods for finding regions the matrix *SP* is translated to a grayscale image. The value of each position in the matrix is normalised and converted to the intensity value of the corresponding pixel in the resulting image. Examples of grayscale images in which sector-like patterns (phase variation) have been detected are depicted in Figure 3.

The parameterised mechanism for detecting regions in grayscale images is described in Algorithm ??. All mentioned subalgorithms are implemented in the open source Computer Vision library OpenCV [38]; see Table 1 for a mapping between the subalgorithms described in Algorithm ?? and the OpenCV functions. Detailed descriptions of the OpenCV function parameters are provided in [39] and will not be restated here.

**Table 1:** **Mapping between subalgorithms employed by Algorithm ?? and OpenCV functions**

**Subalgorithm signature**	**OpenCV function signature**
ChangeBrightnessAndContrast(image, alpha, beta)	convertTo(image, -1, alpha, beta)
MorphologicalCloseOperation(image, morphCloseNrOfIter)	morphologyEx(image, outputImage, MORPH_CLOSE, Mat(), Point(-1, -1), morphCloseNrOfIter)
GaussianBlur(image, kernelSize, standardDev)	GaussianBlur(image, outputImage, kernelSize, standardDev)
Threshold(image, thresholdValue)	threshold(image, outputImage, thresholdValue, 255 THRESH_BINARY)
DetectAndApproximateContours(image, approximationLevel)	findContours(image, contours, contoursHierarchy, CV_ RETR_CCOMP, CV_CHAIN_APPROX_NONE, Point())
	approxPolyDP(image, outputImage, approximationLevel, true)

The left column describes the signature of the subalgorithms employed by Algorithm ??. The right column describes the signature of the corresponding OpenCV function(s).



#### Clusters

Given a collection of regions, the cluster detection mechanism constructs groups of sufficiently similar regions. During this procedure no assumption is made regarding the size and type of the regions. In contrast to the region detection mechanism, the mechanism for detecting clusters operates in a heterogeneous context where both fixed and variable size subsets of the discretised space are considered.

Our assumption is that two regions should belong to the same cluster if the distance between them is below a certain threshold. A distance pseudometric *d* is defined for this purpose:

$$d : REG \times REG \rightarrow \mathbb{R}, d(A, B) = \sqrt{(x_{B} - x_{A})^{2} + (y_{B} - y_{A})^{2}} $$

where *REG* is the set of all regions, and *d*(*A*,*B*) computes the Euclidean distance between the centroids of two regions *A*,*B*∈*REG*.

##### **Definition4**.

A **cluster***C* with respect to a set of regions *REG*, and a pseudometric *d*, is a subset of regions in *REG* such that ∀*x*,*y*∈*C*,*d*(*x*,*y*)≤*ε*_*distance*_ and |*C*|>*ε*_*size*_, where *ε*_*distance*_ and *ε*_*size*_ are user-defined parameters.

**Figure 4:**
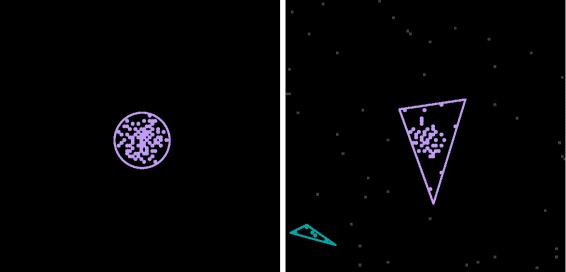
**Detection of clusters in population of cells.** Grayscale images representing the distribution of cells at a particular timepoint in two chemotaxis model simulations. Clusters comprising at least 5 sufficiently close cells are automatically detected and outlined using different colours. Cells are represented as gray points if they do not belong to a cluster. Otherwise they are represented as coloured points such that the colour of the cell matches the colour of the cluster it is a member of. Each cluster is enclosed by a polygon whose shape (triangular, rectangular or circular) best matches the shape of the cluster. Similarly to Figure 1B cells aggregate in the centre where the concentration of chemical attractant is highest.

The problem of grouping entities into clusters is addressed by the cluster analysis literature [40]. A popular algorithm which considers distance (not necessarily Euclidean) as a criterion for grouping objects is DBSCAN [41]. The original algorithm has a known issue because the assignment of border objects (i.e. objects between multiple clusters) to clusters depends on the order in which the set of objects is iterated. An improved version of the DBSCAN algorithm was introduced in [42] for addressing this issue and is employed by our cluster detection mechanism considering the pseudometric *d* as the distance function. Illustrative examples of grayscale images in which clusters of cells (chemotaxis) are automatically detected are depicted in Figure 4.

#### Spatial measures

**Figure 5:**
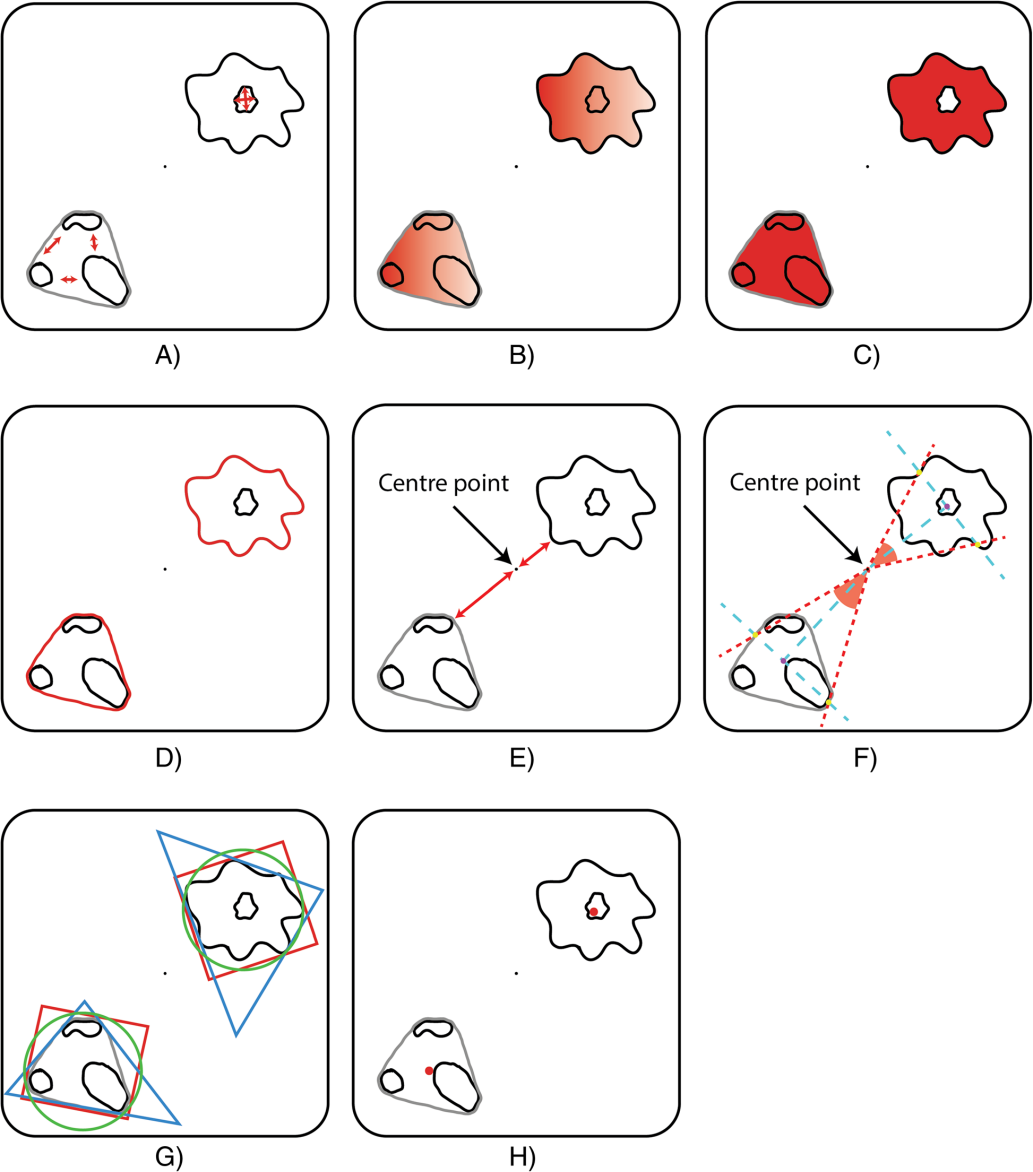
**Visual description of the spatial measures.** The clusteredness **(A)** computes how close regions/neighbouring positions are to each other in a cluster/region. Density **(B)** measures the average value (e.g. concentration) of the considered positions in the discretised space. Area **(C)** and perimeter **(D)** have the usual meaning from discrete 2D geometry. Distance from the origin **(E)** represents the minimum distance between the point from the centre of the discretised space and the considered region/cluster. The angle **(F)** associated to a region/cluster is determined by three points: the origin, and the points found at the intersections of the region/cluster convex hull with the line perpendicular on the line determined by the origin and the centroid of the region/cluster. The shape **(G)** is determined by computing the degree of similarity between the shape of the region/cluster and a triangle, rectangle and circle. The centroid **(H)** is the geometric centre of the considered region/cluster.

Each detected region/cluster is characterised by the set of spatial measures SM = {clusteredness, density, area, perimeter, distance from origin, angle(degrees), triangle measure, rectangle measure, circle measure, centroid (x-coord), centroid (y-coord)}. A detailed description of the semantics specific to regions and clusters is provided below; see Figure 5 for a graphical illustration.

#### Semantics of spatial measures for regions

The *clusteredness* of a set of regions represents the inverse of the average Euclidean distance between the centroids of the regions. Conversely the clusteredness of a single region is computed as follows:

$$ clusteredness(r) = \frac {area(r)} {area(r) + \sum\limits_{h \in holes}{area(h)}} $$

where *r* is a region and *holes* is the set of holes contained by *r*. As the area of the holes contained by regions increases the value of the clusteredness degree decreases and vice-versa.

The *density* of a set of regions is equal to the average density of the regions divided by the average Euclidean distance between the centroids of the regions. Conversely the density of a single region represents the average density value of the positions defining the region in the discretised space.

The *area* of the region is equal to the area of the polygon defined by the neighbouring positions in the Euclidean plane (subtracting the area of holes).

The *perimeter* of the region is equal to the perimeter of the polygon defined by the neighbouring positions in the Euclidean plane. Holes contained by the region are ignored in this case.

The *distance from the origin* is equal to the minimum distance between the polygon defined by the region and the centre point of the discretised space (origin).

The *angle (degrees)* is equal to the angle determined by the centre point of the discretised space *P* and the points obtained from the intersection of the line perpendicular on the line determined by *P* and the centroid of the region, and the convex hull of the polygon defined by the region.

The shape of the region is determined in a fuzzy manner by the *triangular*, *rectangular* and *circular* measures. Each one of these measures computes the likelihood of the region to have a triangular, rectangular, respectively circular shape using the following formula:

$${\small\begin{aligned} &measure_{s}(r)\\ &\quad= \frac {area(r)} {area(\textnormal{minimum area} s \textnormal{-shaped polygon enclosing}\,\, r)} \end{aligned}} $$

where *r* is a region, and the value of *measure*_*s*_(*r*)∈ [ 0,1],∀*s*∈ {triangular, rectangular, circular}. Algorithms for computing the minimal enclosing triangles are provided in [43], respectively [44,45] for rectangles and [46] for circles. For the phase variation case study, where the region detection mechanism is employed, the triangular shape is most relevant because it closely matches the shape of the sector-like patterns in the bacterial colonies; see Figure 1A for examples of such patterns highlighted in black.

The *x/y-coordinates* of the centroid are computed using *moments* of the polygon defined by the neighbouring positions in the Euclidean plane [47].

#### Semantics of spatial measures for clusters

The *clusteredness* of a set of clusters represents the inverse of the average Euclidean distance between the centroids of the clusters^a^. Conversely the clusteredness of a single cluster represents the inverse of the average Euclidean distance between the centroids of the regions in the cluster.

The *density* of a set of clusters is equal to the average density of the clusters divided by the average Euclidean distance between the centroids of the clusters. Conversely the density of a single cluster represents the average density value of the spatial entities defining the cluster in the discretised space.

The *area* of a cluster is equal to the area of the polygon defined by the convex hull of all regions in the cluster (ignoring the holes between regions).

The *perimeter*, *distance from the origin*, *angle (degrees)*, *shape* and *x/y-coordinates* of the centroid of the cluster are determined using the same methods employed for regions. The main difference is that the polygon used to determine the outer boundary of the cluster is the convex hull computed for a group of regions instead of a single one. Moreover for the chemotaxis case study the circular and rectangular shapes are most relevant because they closely resemble the shape of cells clusters, respectively the shape of cell streams moving towards the point where the chemical attractant concentration is highest; see Figure 1B for an example of a circular cluster forming in the middle, respectively streams of cells moving towards it.

#### Spatio-temporal markup language (STML)

The output of the region/cluster detection mechanism comprises the spatial measures computed for each region/cluster. A standard data representation format is employed to describe the evolution of these spatial and other numeric measures over time. The main advantage of such a format is that data is represented in a uniform and consistent manner which facilitates exchange of data sets and integration of software tools. We define the Spatial Temporal Markup Language (STML) as an initial attempt to standardise the representation of spatio-temporal time series data.

For portability, structuring and readability purposes spatio-temporal time series data is stored in eXtensible Markup Language (xml) files. The rules and constraints for the structure of these xml files are formalised in XML Schema Definition (xsd) files with the filename format STML_LxVy.xsd (Spatial Temporal Markup Language Level x, Version y); see [48] for the latest version of the format. An example of an xml file recording experimental spatio-temporal data is depicted in Listing 1.



The results of an (*in silico/vitro/vivo*) experiment are recorded as a list of time points. The constraint imposed on experiment elements are that they must contain at least one time point.

Each timepoint element can be identified by a non-negative integer value representing when the data was recorded. In case values are missing (e.g. *in silico* experiments) the value is determined automatically using the following formula:

$${\small{t_{i}=\left\{ \begin{array}{ll} val, &\ \text{if the value}\ val\ \text{was predefined for}\ t_{i}\\ 0, &\ \text{if no value was predefined for}\ t_{i}\ \text{and}\ i = 0\\ t_{i}-1 + 1, &\ \text{otherwise} \end{array}\right.} }$$

The information stored in timepoint elements are a list of zero or more unique spatial entities, and a list of zero or more unique numeric state variables.

A spatialEntity element currently comprises only one element called pseudo3D which stores a pseudo3D spatial description of the entity. In the future if 2D or full3D representations are of interest they can be added as additional child elements to the spatialEntity element.

Every pseudo3D element has an associated type which can be either *cluster* or *region*. Similarly to the detected regions/clusters every pseudo3D element is characterised by a set of spatial measures constrained as described below:

- *clusteredness*, *density*, *area*, *perimeter*, *distanceFromOrigin*, *centroidX* and *centroidY* - real non-negative values;
- *angle* - a real non-negative value between 0 and 360;
- *shape* - an optional element which can take the values “triangular”, “rectangular” or “circular”;
- *triangularMeasure*, *rectangularMeasure* and *circularMeasure* - real non-negative values between 0 and 1;

The basic shapes considered by the current version of STML are appropriate to describe simple spatial patterns such as patches which spread outwards as they develop (triangular), ordered structures/streams (rectangular), and (uniform) groups/clusters (circular). Illustrative real-life examples of such shapes are the sector-like patches highlighted in black in Figure 1A (triangular), and the streams of cells (rectangular) depicted in Figure 1B which chemotactically migrate towards the centre and form a cluster (circular). In contrast complex patterns composed of multiple basic shapes cannot be described appropriately by the current shape similarity measures. In order to address this issue a potential future version of STML could include a more complex suite of shape descriptors.

Finally numericStateVariable elements contain a name and a value child element where the name is a string and the value a real number.

All timeseries data are translated to STML using the region/cluster detection mechanisms and are then provided to the model checker Mudi for evaluation.

### Bounded Linear Spatial Temporal Logic

We define a logic called Bounded Linear Spatial Temporal Logic (BLSTL) for specifying quantitative spatio-temporal properties against which STML files are automatically evaluated. BLSTL is an extension of BLTL, and LTL, with spatial, arithmetic and statistical functions. The temporal and Boolean propositions specific to BLTL remain unchanged, but new functions are introduced enabling to reason about how (distributions of) regions/clusters and their spatial properties change over time. For brevity purposes only an informal description of BLSTL is provided below; see Additional file 2 for more details and the formal syntax and semantics definition.

The same non-dimensional properties, spatial entities (regions and clusters) and measures (clusteredness, density, area, perimeter, distance from origin, angle(degrees), triangle measure, rectangle measure, circle measure, centroid (x-coord) and centroid (y-coord)) are considered both by the STML specification and the BLSTL formal language. Therefore BLSTL enables encoding logic statements with respect to both non-dimensional (e.g. species/proteins concentrations) and spatial properties, and correlations between the two.

In order to allow the construction of more complex logic statements BLSTL additionally enables specifying how arithmetic expressions comprising non-dimensional or spatial properties change over time. The considered functions which enable the construction of complex logic statements are either unary (e.g. absolute value, round, square root etc.) or binary (e.g. addition, division, power etc.).

These arithmetic functions take a single real value as input and are directly applicable to non-dimensional properties. However in order to apply the same functions to collections of regions/clusters, the distribution of spatial measures characterising the regions/clusters has to be reduced to a single real value. A set of statistical functions is made available in the specification of BLSTL in order to address this problem. The considered statistical functions are either unary (e.g. count), binary (e.g. median with respect to a user specified spatial measure), ternary (e.g. percentile with respect to a user specified spatial measure) or quaternary (e.g. covariance between two potentially different types of spatial entities and measures). One of the main differences between BLSTL and traditional BLTL-based formal languages is that the former enables reasoning about dynamic sets of spatial patterns whose cardinality changes over time, while the latter usually only considers fixed sets of non-dimensional variables.

Although the arithmetic and statistical functions described above enable the construction of more complex logic statements, there is a need for a mechanism which enables reasoning about particular subsets of the detected regions/clusters. For instance it may be the case that only regions with the area greater than a certain value, or clusters close to a particular point in space are of interest. In order to address this challenge BLSTL comprises a constraint-based mechanism which filters out all regions/clusters whose spatial measures do not meet a set of user-defined conditions.

Examples of natural language statements which can be encoded in BLSTL using the logic constructs defined above are:

- Considering the time interval [0, 100], at some point in the future the number of cell clusters emerging in the environment, when the concentration of *cAMP* is less than 20, is greater than zero (Natural language);
  *F*[ 0,100] (({*cAMP*}<20)∧(*count*(*clusters*)>0)) (BLSTL).
- The mean area of all cancerous regions grows throughout the entire simulation interval [5, 25] (Natural language);
  *G*[ 5,25] (*d*(*mean*(*regions*,*area*))>0) (BLSTL).
- Within the time interval [0, 300] the number of mutant cell populations emerging at a distance smaller than 10 from the area of inflammation (origin) is greater than 0 until the concentration of *X* drops below 5 (Natural language);
  (*count*(*filter*(*clusters*,*distanceFromOrigin*<10))>0)*U*[ 0,300]({*X*}<5) (BLSTL).

BLSTL can be employed for specifying properties of individual simulation traces. However for specifying properties over a collection of traces we will extend BLSTL to Probabilistic BLSTL.

#### Probabilistic BLSTL

##### **Definition5**.

A Probabilistic Bounded Linear Spatial Temporal Logic (PBLSTL) property *ϕ* is a logic property of the form *P*_⋈*θ*_[ *ψ*] where ⋈ ∈{<,≤,>,≥}, *θ*∈(0,1) and *ψ* is a BLSTL property.

A PBLSTL property *ϕ*≡*P*_⋈*θ*_[ *ψ*] holds for a SSpDES  ($\mathcal {M} \models P_{\bowtie \theta } [\!\psi ]$) if and only if the probability of *ψ* to hold for an execution of  is ⋈*θ*. Therefore in order to determine the truth value of a PBLSTL property *ϕ* the likelihood of it being true is computed.

Similarly to [17] evaluating the truth value of a PBLSTL property *ϕ* is harder than determining the truth value of a BLSTL property *ψ*. One counterexample for a BLSTL property is sufficient to decide that the property does not hold. Conversely one counterexample for a PBLSTL property *ϕ* does not necessarily imply that *ϕ* is not satisfied. A PBLSTL property *ϕ* does not hold if the likelihood of all counterexamples provides sufficient evidence to invalidate *ϕ*.

### PBLSTL Model checking

#### **Definition6**.

The *probabilistic spatio-temporal model checking problem* is to automatically verify if a SSpDES  satisfies a PBLSTL property *ϕ*≡*P*_⋈*θ*_[ *ψ*].

Different approximate probabilistic model checking algorithms can be employed depending on the method of constraining the approximation error and the approach for deciding if a logic property holds. For flexibility and completeness purposes in our approach both Bayesian and frequentist, statistical hypothesis test and estimate based methods are considered. The specific algorithms which were considered are provided in Table 2.

**Table 2:** **Considered approximate probabilistic model checking approaches**

	**Frequentist**	**Bayesian**
**Estimate**	Chernoff-Hoeffding	
	bounds [49]	Mean and variance [50]
**Hypothesis testing**	Statistical [51]	Statistical [17]
	Probabilistic	
	black-box [52,53]	

Bayesian methods consider prior knowledge about the parameters and variables in the model when deciding if a logic property holds. Conversely frequentist approaches assume no prior knowledge is available. All methods except probabilistic black-box take as input a user-defined upper bound on the approximation error. They request additional model executions until the result is sufficiently accurate. Probabilistic black-box model checking takes a fixed number of model simulations as input and computes a p-value as the confidence measure of the result.

All methods except probabilistic black-box take a user-defined (set of) parameter(s) as input representing the acceptable value of the approximation error. Such methods request and evaluate a variable number of model simulations until the approximation error constraints are satisfied. Conversely the probabilistic black-box model checking approach decides based on a fixed number of model simulations if the logic property is satisfied. However in this case the confidence measure of the provided result is not specified by the user and varies depending on the number of available model simulations.

Bayesian approaches should be used when information about the prior probability distribution of parameters in the model is available. This could lead to a reduced number of required samples in order to decide if a logic property holds. Conversely if no prior knowledge is available frequentist methods could be employed instead.

Statistical hypothesis test based approaches should be employed whenever deciding between two hypotheses where usually the null hypothesis represents the PBLSTL logic property *ϕ*, respectively the alternative hypothesis ¬*ϕ*. Conversely if the true probability of *ϕ* being true is computed and then compared to *θ* estimate based methods should be considered.

The algorithms provided in the original papers describing the model checking methods (see Table 2) were employed for all approaches except frequentist statistical. An improved version of this model checking method requiring less input parameters is described in [54]. However the initialisation step of the improved algorithm could potentially lead to invalid arithmetic expressions if extra conditions are not added to the algorithm implementation (C.H. Koh, personal communication, 2 ^*nd*^ of June, 2014). We propose a variant of the algorithm described in [54] with a modified initisalisation step which no longer requires adding extra conditions to the implementation. A more detailed description of the proposed solution which we consider in our approach is given in Additional file 3.

Finally the semantics of all considered approximate probabilistic approaches is described in Additional file 4. Moreover we prove in Additional file 5 that the model checking problem is *well-defined*; see ([55], Appendix A) for a similar proof for BLTL.

#### Implementation

All spatio-temporal model validation algorithms were implemented in the multidimensional model checking platform Mudi^b^. For both efficiency and cross platform compatibility reasons Mudi was implemented in C++. The current version of the model checker was designed to be executed only from the command line. The user chooses the desired model checking algorithm and enters the required parameters via command line flags; run Mudi with the “–help” command line argument for more details.

**Figure 6:**
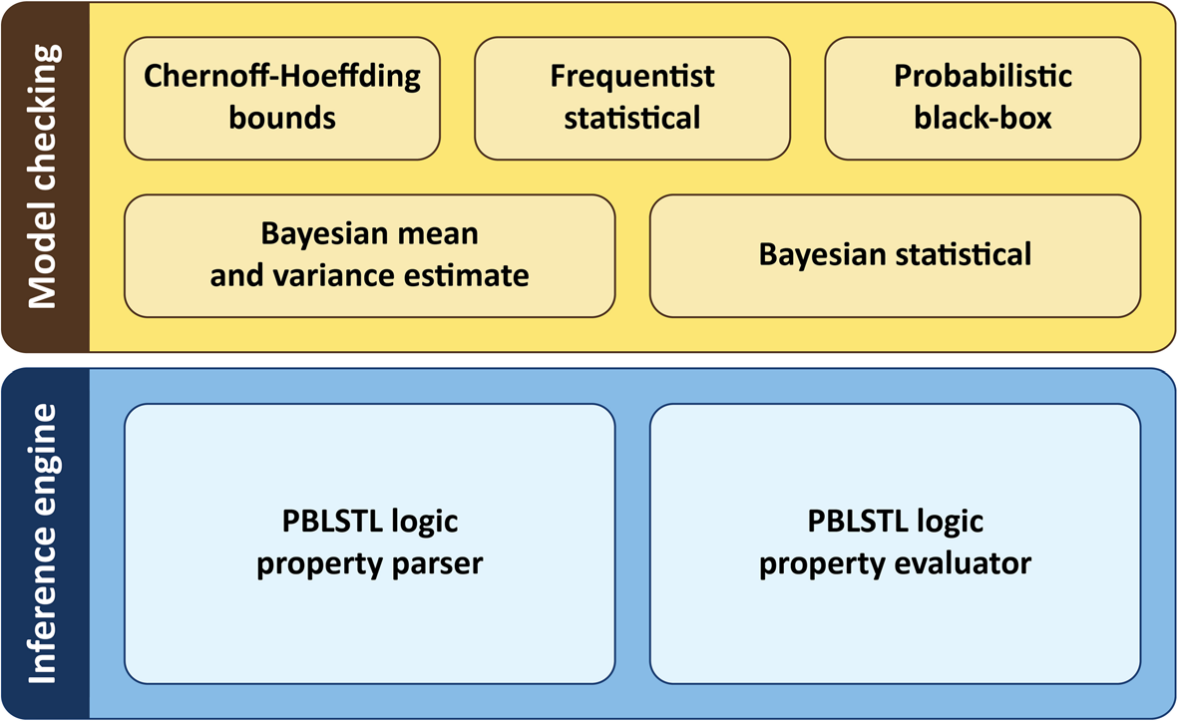
**The architecture of the model checker Mudi.** The model checking layer is decoupled from the inference engine and comprises all model checking types supported by Mudi. All model checking types depend on the same inference engine layer which contains the PBLSTL logic property parser (considering BLSTL syntax) and evaluator (considering BLSTL semantics).

The modular architecture of Mudi is separated into the inference engine and the model checking layers as depicted in Figure 6. The main advantage of this design choice is that changes at the inference engine layer do not require updates at the model checking layer and viceversa.

The model checking layer comprises all supported model checking algorithms. Independently of the chosen algorithm the same inference engine is used for the logic statements’ evaluation. Conversely the inference engine layer comprises the logic statements parsing and evaluation algorithms. The parsing module verifies if a given logic property is syntactically correct and the evaluation module determines if the property is true/false considering a spatio-temporal execution of the model.

## Results

We have illustrated the efficiency and expressivity of our methodology based on two case studies: phase variation patterning in bacterial colony growth (regions) and the chemotactic aggregation of cells (clusters). The datasets employed for both case studies were generated *in silico* through model simulation.

In both cases our assumption was that no prior knowledge is available and therefore a frequentist model checking approach was employed. Moreover for simplicity purposes only the frequentist statistical model checking results will be presented here. However all model checking approaches have been tested against these datasets. Relevant comparisons between different approximate probabilistic model checking approaches are given in the original papers introducing them. Since these approaches abstract away from particular model representations and logic formalisms the comparison results should not change and therefore will not be restated here.

### Phase variation patterning in bacterial colony growth

Phase variation is a stochastic gene expression switching mechanism employed by microbial populations to potentially develop variants which adapt to foreseeable frequent environmental or selective conditions [36,56,57]. In particular it is of interest to better understand how pathogenic organisms use this mechanism to adapt to different hosts and evade host defenses and immune responses. The most readily observable compositional effect of phase variation in cultures grown *in vitro* is the development of sector-like patterns. To study the growth of bacterial colonies with phase variable genes a computational model was constructed [35] enabling the investigation of different parameter sets (e.g. mutation and fitness rates) and geometries (rectangular and circular). The model was constructed using Coloured Stochastic Petri Nets in Snoopy [58] and was executed on a Unix cluster using MARCIE [30]; see ([35], Section 5) for details on how to obtain a copy of the model(s). For brevity purposes only the rectangular version of the model was considered here.

**Figure 7:**
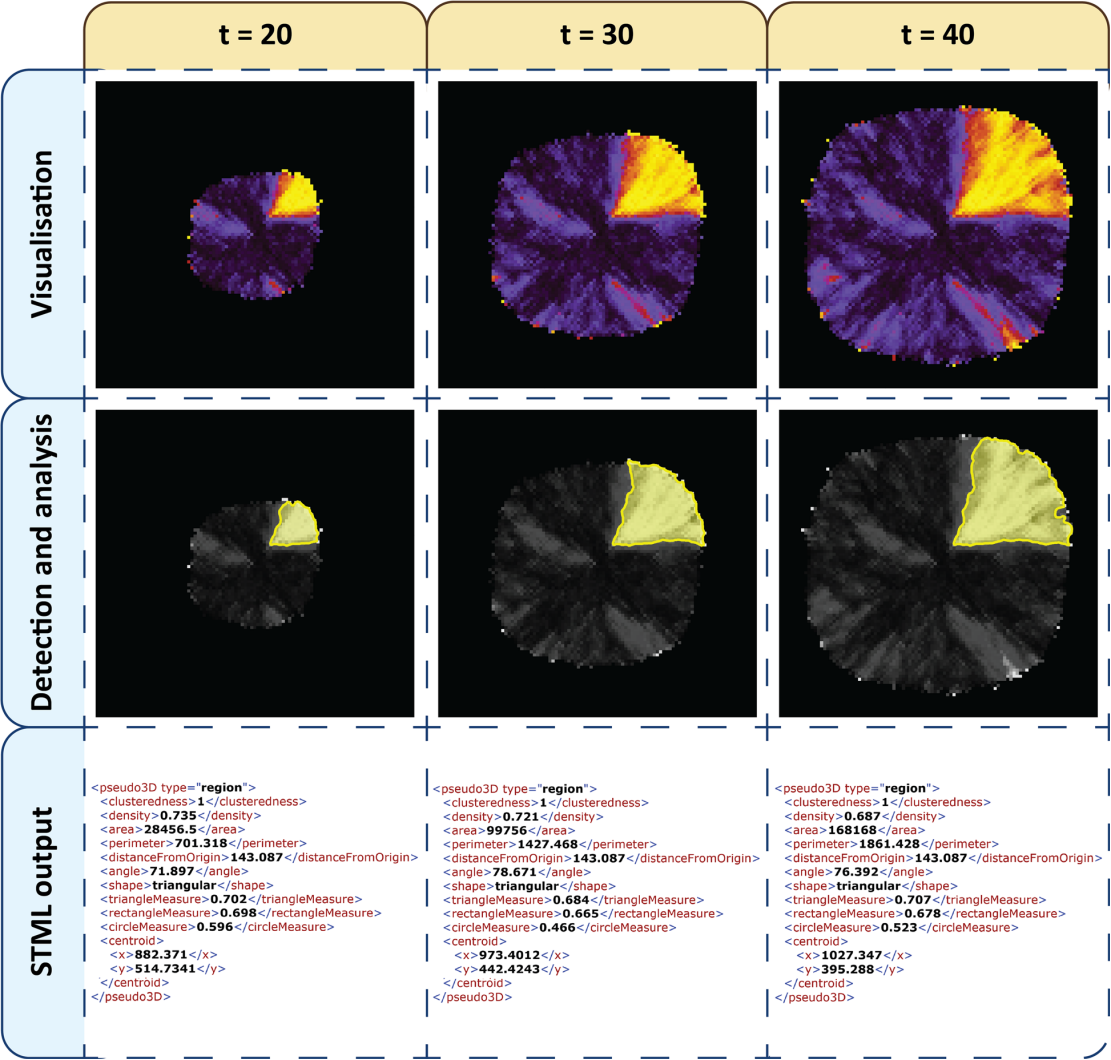
**Spatio-temporal detection and analysis of a phase variation model simulation.** Each column corresponds to a different timepoint from the simulation (t = 20, 30 and 40). The rows considered from top to bottom represent the stages of translating timeseries data to STML output files (Visualisation as images, automatic detection and analysis of regions/sector-like patterns, and output in STML format).

All one thousand stochastic simulations which were executed for the rectangular model during our previous study [35] will be reused. In order for the simulation output to be processable by Mudi it needs to be translated into STML format. For this case study the region detection and analysis module was employed because sector-like patterns (and not clusters of such patterns) are of interest. An example of the translation steps applied to each spatio-temporal timeseries is depicted in Figure 7. The simulation output is visualised as images from which regions are extracted and analysed. Results corresponding to each timeseries are stored in a separate STML file.

The generated STML dataset is evaluated against the formal specification comprising PBLSTL logic properties. Depending on the modelled microorganism and the associated mutation rates the values and/or parameters of the logic properties will vary. We will describe here a generic set of logic statements to illustrate the expressivity of the formal language PBLSTL. Therefore the structure of PBLSTL statements is emphasized and not particular parameter values. Moreover the chosen PBLSTL probability values are approximations relative to their expected level (e.g. high, medium or low). For simplicity purposes the specification, which partially relates to Figure 1A, will be described in natural language below; see Additional file 6 for the equivalent specification written in PBLSTL.

1. One of the first requirements is that the probability of the number of sector-like patterns to increase or stay constant (but never decrease) during the bacterial colony growth is greater or equal to a threshold value. In our case we set this threshold to 0.95. The reason for this requirement is that we do not expect developed sectors to disappear.
2. In case sector-like patterns emerge the probability that one of them will contain holes is less than 0.05. This statement can be rewritten using the clusteredness measure of the regions i.e. the probability that the minimum clusteredness degree of all sectors is less than a certain threshold value (in our case 0.9) is less than 0.05.
3. The average density of the detected sectors, representing the concentration of “mutant” cells relative to “normal” cells, should be greater than 0.5 with probability greater than 0.95.
4. Moreover the average area of the sectors oscillatesat least one time during the growth of the bacterial colony with probability greater than 0.5. By oscillations we mean an increase of the average area followed eventually by a decrease or viceversa. In PBLSTL oscillations can be represented using the difference operator *d*. For this particular statement we will specify that at some point in the future the rate of change (difference) of the average area will be positive and then eventually negative or viceversa. Such oscillations are expected because the relative density of “mutant” cells with respect to “normal” cells is considered when detecting sectors. Therefore as the colony grows it may be the case that at the most outward edge of a sector initially the “mutant” cells dominate a position in the discretised space but then they are overrun by the “normal” cells. In other words it may be the case that a position which is contained by a sector will no longer do so in the future.
5. Following the same reasoning we also specify that the average perimeter value of the sectors oscillates at least five times during the growth of the bacterial colony with probability greater than 0.6. From an implementation point of view this logic statement was added to check the increase in runtime due to nesting multiple temporal logic propositions.
6. The maximum angle described by any sector with respect to the origin is expected to be greater than 120° with probability less than 0.1.
7. Moreover sectors are expected to develop from the origin outwards. Therefore the minimum distance from the origin would be expected to be greater than 100 (relative to scale of analysed images) with probability greater or equal to 0.95.
8. Finally on average most of the sectors should develop and maintain a triangular-like shape throughout the entire bacterial colony growth with probability greater than 0.8.

The natural language specification was translated to PBLSTL such that the *i*-th PBLSTL logic statement corresponds to the *i*-th natural language statement. Each PBLSTL statement (stored in a separate input file) was individually evaluated against the STML dataset 500 times using the frequentist statistical model checking approach implemented in Mudi. The results corresponding to each PBLSTL statement and execution of the model checker are described in Additional file 7. Conclusions drawn from the statistical analysis of the results corresponding to each PBLSTL statement are summarized in Table 3.

**Table 3:** **Model checking statistical analysis results for the phase variation case study**

**id**	**% true**	**#total STML**		**#true STML**		**#false STML**		**Exec. times**	
	**PBLSTL**	***μ***	***σ***	***μ***	***σ***	***μ***	***σ***	***μ***	***σ***
1	100	67.95	12.51	67.12	11.52	0.83	0.98	0:2.87	0:0.57
2	100	103.21	57.70	0.81	1.18	102.40	56.56	0:4.40	0:2.60
3	100	63.05	8.43	62.62	7.74	0.43	0.69	0:2.75	0:0.43
4	99.6	3.29	12.79	1.84	7.51	1.45	5.36	0:0.22	0:0.83
5	74.6	15.35	14.17	6.98	5.94	8.36	8.72	0:0.99	0:0.92
6	99.8	982.63	111.18	10.74	1.62	971.88	109.59	0:43.71	0:9.70
7	100	106.21	42.53	102.53	39.42	3.68	3.11	0:4.67	0:2.02
8	99.8	30.04	63.24	24.87	51.40	5.17	11.99	0:1.33	0:2.59

Entries in the “id” column represent the numeric identifiers placed at the right of each PBLSTL statement. The “% true PBLSTL” column describes what percentage of the 500 executions concluded that the PBLSTL statement is true. “#total STML” represents the total number of STML files evaluated for the PBLSTL statement; columns “#true STML” and “#false STML” represent the number of STML files for which the PBLSTL statement was evaluated true, respectively false.“ *μ*” and “ *σ*” represent the mean and standard deviation. “Exec. times” presents the average model checking execution time for each PBLSTL evaluation using the “minutes:seconds” format.

For half of the PBLSTL statements (id = 1, 2, 3, 7) 100% of the 500 model checker executions concluded with the answer true. However in case of PBLSTL statements 6 and 8 the percentage was 99.8%, respectively 99.6% for PBLSTL statement 4 and 75.6% for PBLSTL statement 5. It is important to note that this does not mean that the model checking results are incorrect. Moreover in the approximate probabilistic setting if the model checking result is false for a logic property *ϕ* this does not imply that ¬*ϕ* is true. The variation in the results obtained for PBLSTL statement 4, 5, 6 and 8 are due to the fact the we executed the model checker with the maximum probability of type I and type II errors equal to 5%. Under these assumptions the evaluation result for a PBLSTL statement depends on the order and number of obtained true/false evaluations for individual STML files. To reduce the variation of the PBLSTL evaluations the value of the probability of type I/II errors needs to be decreased. The required number of evaluated simulations is indirectly proportional to the type I/II error probability. Thus more simulation evaluations are required as the error probabilities are decreased. In the extreme case if the probability of both type I and type II errors is set to zero the expected number of evaluated simulations is infinite i.e. the entire state space of the model would be potentially investigated.

Similarly there is a significant difference in the average total number of STML files against which the PBLSTL statement was evaluated. Depending on the comparison operator (>,<,>=,<=) and the specificity of the probability *θ* corresponding to each PBLSTL statement more/less evidence is required to prove that the statement is true/false. In our case the logic statement 6 required on average more than 950 STML evaluations and most of the time more than the maximum number of available simulations 1000 (see Additional file 7 for results corresponding to each model checker execution). Since no path to an external model simulator was specified the model checker did not have enough evidence to decide using the frequentist statistical model checking approach if the PBLSTL statement holds. Therefore the provided answer was computed using the probabilistic black-box model checking approach.

The considerable difference in the number of required STML files is additionally reflected in the average execution times of the model checker. Thus the highest average execution time was recorded for the evaluation of PBLSTL statement 6. Since the formal specification for this case study comprises all PBLSTL statements the average execution time for the entire specification is computed as the sum of all average execution times (see column 9, Table 3):

$$ \begin{aligned}  \text{Execution time}_{\text{specification}} &\!= 0:2.87 + 0:4.40 + 0:2.75 \\ &\quad+ 0:0.22 + 0:0.99 \\ &\quad + 0:43.71 + 0:4.67\\ &\quad+ 0:1.33 \\ &= 01:0.94\ \text{(minutes:seconds)}. \end{aligned} $$

In order to decrease the overall execution time the model checker was extended such that it can evaluate the specification comprising all PBLSTL statements in a single run. In this case each STML file is read into memory only once and thus reduces the number of required input/output (I/O) operations. Under these conditions the average execution time for the entire specification considering 500 runs was 0:44.41 (minutes:seconds), compared to 01:0.94 when the PBLSTL statements were evaluated individually.

For reproducibility purposes the dataset of generated STML files and the file containing the spatio-temporal PBLSTL statements are made available in Additional files 6 and 8.

### Chemotactic aggregation of cells

Chemotaxis is the process through which cells detect concentration changes in chemical gradients and move towards chemical attractants, respectively away from chemical repellants. It is employed both by prokaryotic and eukaryotic cells and underpins many biological processes (e.g. human leukocytes migrate to sites of inflammation, cancer cells metastasize to other organs) [59,60]. In an attempt to better understand the intracellular mechanisms underlying chemotaxis computational models for various type of cells have been constructed [61]. Although such models differ at the intracellular level they exhibit relatively similar behaviours at the population level i.e. cells aggregate in the area with the highest concentration of chemical attractants. In this work we were only interested in the evolution over time of the spatial distribution of cells and therefore have abstracted away from all the intracellular details.

A computational model illustrating the chemotactic aggregation of cells was constructed using the modelling and simulation software Morpheus [62]. The discretised 2D space was represented using a rectangular lattice of size 100 ×100 on which 100 cells were randomly distributed; cells’ positions are recomputed for each model simulation. In order to activate the chemotactic behaviour of the cells a chemical gradient was added in the environment according to a Gaussian distribution with parameters *μ*_*x*_=*μ*_*y*_=50 and *σ*_*x*_=*σ*_*y*_=10. The cells and their movement in the environment was represented using a Cellular Potts model [63] and the distribution of the chemical gradient was encoded using partial differential equations. A copy of the model is made available as Additional file 9.

**Figure 8:**
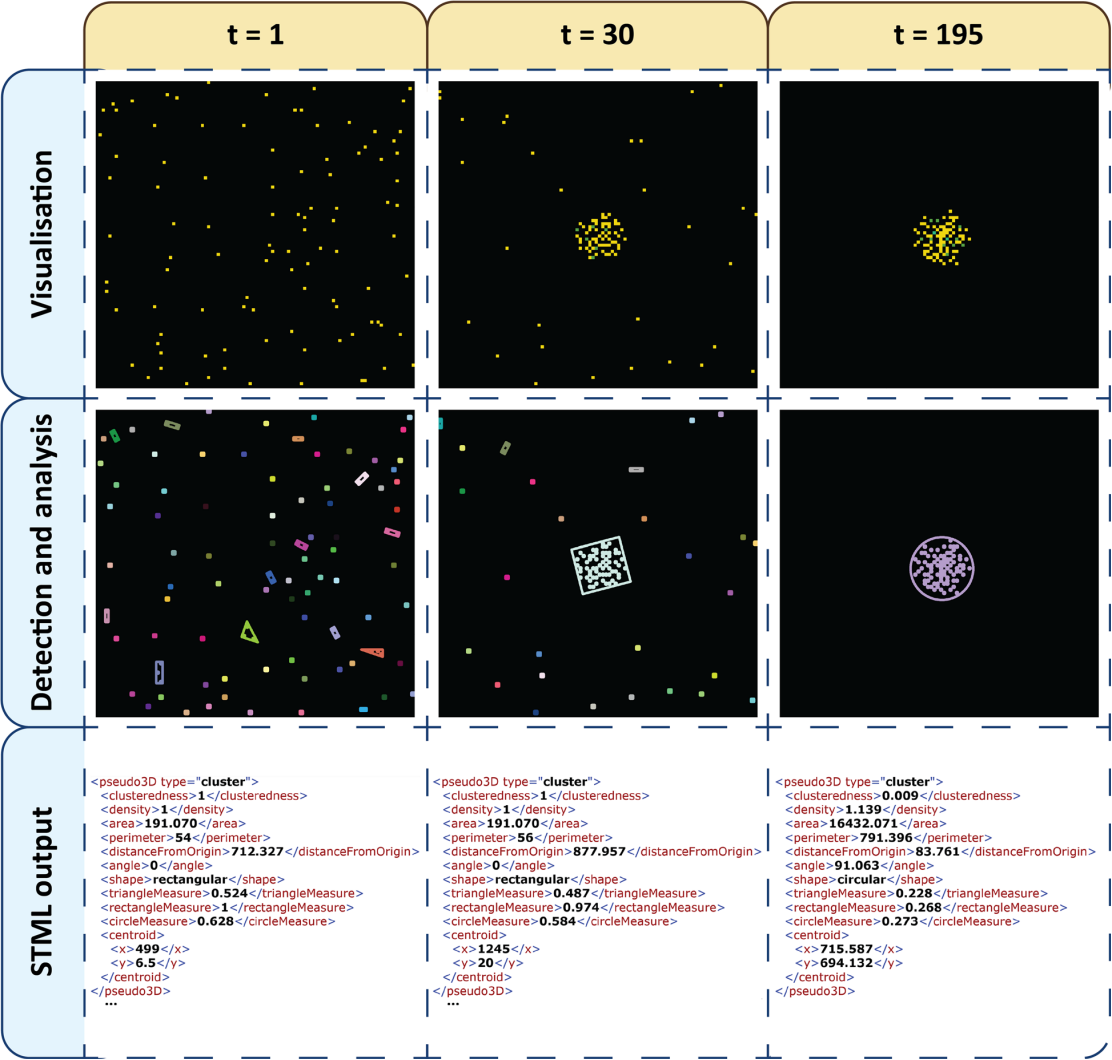
**Spatio-temporal detection and analysis of a chemotaxis model simulation.** Each column corresponds to a different timepoint from the simulation (t = 1, 30 and 195). The rows considered from top to bottom represent the stages of translating timeseries data to STML output files (Visualisation as images, automatic detection and analysis of regions/sector-like patterns clusters, and output in STML format). The colour employed in the first row plots represents degree of pileup. “Yellow” positions in the discretised 2D space are occupied by 1 cell, respectively “green” positions by 2 cells and “teal” positions by 3 cells. The colours employed in the second row plots are used only to distinguish between different clusters. Each cluster is surrounded by a polygon whose shape (triangular/rectangular/circular) best matches the shape of the cluster.

The output of each model simulation was translated to STML using the cluster detection mechanism because groups of (and not individual) cells were of interest. Cells occupied only one position of the discretised space and therefore their detection in images was straightforward. Instead of employing the region detection mechanism we implemented a custom lightweight cell detector which verifies the presence/absence of cells in each position (including pileup) considering the average pixel intensity; see Figure 8 for an example of the translation steps performed by the cluster detection mechanism for each model simulation.

In order to illustrate the integration of Mudi with a model simulator the model checker was executed initially without making available any STML files. Instead an external script responsible for simulating the model and converting the output to STML was provided as a command-line parameter. Thus Mudi executed the script on demand whenever extra model simulations were required. In general if a large number of simulations is required the maximum model checking time can be bounded via a user-defined parameter.

Considering this scenario the computational model was validated against a formal PBLSTL specification. Similarly to the phase variation case study the chosen set of logic statements is generic and was chosen to illustrate the expressivity of PBLSTL and not the phenotypic characteristics specific to a particular type of cells. Moreover the specification, which partially relates to Figure 1B, will be described in natural language below; see Additional file 10 for the equivalent specification written in PBLSTL.

9. One of the most important properties is that cells aggregate in the area with highest concentration of chemical attractant. This means that at least one cluster is formed at a distance smaller than *δ*>0 from the chemical gradient centre. Let us assume that the cluster centroid is the point (x, y), respectively the centroid of the chemical gradient is (703.5, 678.5). Then a cluster is at a distance smaller or equal to *δ* from (703.5, 678.5) if and only if:
  $${\small\begin{aligned}  d[\!(x, y), (703.5, 678.5)] = \sqrt{(x - 703.5)^{2} + (y - 678.5)^{2}} < \delta \end{aligned} } $$
  Considering that *y*∈(678.5−*δ*,678.5+*δ*) this means that:
  $$\left\{ \begin{aligned} & x < 703.5 + \sqrt{\delta^{2} - (678.5 - y)^{2}} \\ & x > 703.5 - \sqrt{\delta^{2} - (678.5 - y)^{2}} \\ & y < 678.5 + \delta \\ & y > 678.5 - \delta. \end{aligned}\right. $$
  For this particular case study we set the value of *δ* to 50.
10. In addition the average clusteredness degree of individual clusters increases at least 5 times during the simulation time interval [0, 200] with probability greater than 0.8. This means that the average distance between cells in the clusters is reduced at least five times during the specified time interval.
11. In order to quantify the degree of clusteredness within and between different clusters a cluster validity index such as the Sillhouette can be employed. The value of the Silhouette is recorded for each timepoint by the *avgClusterednessClusters* numeric state variable. The probability of the *avgClusterednessClusters* to decrease under a threshold value (in our case 0.5) during the time interval [0, 50] is less than 0.05. Note that *avgClusterednessClusters* could be replaced by any other numeric state variable representing the concentration of a species/protein. Therefore our approach can be employed to reason about both spatial and non-spatial properties, and how changes of non-spatial properties reflect on the spatial properties and viceversa.
12. Similarly the number of clusters is expected to decrease and remain throughout the entire simulation less than 5 with probability greater than 0.75. The reason for this is that simulations start with multiple small clusters which are then expected to merge and form larger clusters close to the area where the chemical attractant concentration is highest.
13. The chemical gradient is distributed such that the areas of approximately equal chemical concentration have a circular/ring shape. Therefore the shape of at least one aggregated cells cluster should be eventually circular with probability greater or equal to 0.6.
14. Finally the probability of the average clusters’ density to never oscillate is less than 0.1. Oscillations of the density are expected because sometimes cells pile up.

Similarly to the phase variation case study the natural language specification was translated to PBLSTL such that the *i*-th PBLSTL logic statement corresponds to the *i*-th natural language statement. Each PBLSTL statement (stored in a separate input file) was individually evaluated against the STML dataset 500 times using the frequentist statistical model checking approach. The results corresponding to each PBLSTL statement and execution of the model checker are described in Additional file 11. The output of the statistical analysis of the results corresponding to each PBLSTL statement are reported in Table 4.

**Table 4:** **Model checking statistical analysis results for the chemotaxis case study**

**id**	**% true**	**#total STML**		**#true STML**		**#false STML**		**Exec. times**	
	**PBLSTL**	***μ***	***σ***	***μ***	***σ***	***μ***	***σ***	***μ***	***σ***
9	100	28	0	28	0	0	0	0:22.04	0:0.13
10	100	14	0	14	0	0	0	0:11.50	0:0.08
11	100	58	0	0	0	58	0	0:44.87	0:0.44
12	100	10.96	0.18	10.92	0.37	0.03	0.18	0:9.27	0:0.16
13	95.6	17.04	73.33	9.57	40.67	7.46	32.73	0:13.73	0:55.45
14	100	28	0	0	0	28	0	0:22.10	0:0.20

Entries in the “id” column represent the numeric identifiers placed at the right of each PBLSTL statement. The “% true PBLSTL” column describes what percentage of the 500 executions concluded that the PBLSTL statement is true. “#total STML” represents the total number of STML files evaluated for the PBLSTL statement; columns “#true STML” and “#false STML” represent the number of STML files for which the PBLSTL statement was evaluated true, respectively false.“ *μ*” and “ *σ*” represent the mean and standard deviation. “Exec. times” presents the average model checking execution time for each PBLSTL evaluation using the “minutes:seconds” format.

Similarly to the phase variation case study there are fluctuations in the evaluation results of some PBLSTL statements. Moreover the number of required STML files to reach a conclusion differs depending on the specificity of the logic statement and the distribution of PBLSTL truth evaluations. In contrast to the phase variation case study for many PBLSTL statements the variation in the number of required STML files, respectively the number of true and false STML evaluations, is equal to zero. Furthermore although the average number of required STML files for the evaluation of a PBLSTL statement (≈ 26) is less than for the phase variation case study (≈ 171.47), the average execution time is higher (chemotaxis: 20.585s, phase variation: 7.6175s). The reason for this is that most of the execution time of the model checker is spent on I/O operations. Thus the execution time depends on both the number and size of STML files which are read into memory. The average STML file size for the phase variation case study is 64759.2 bytes, respectively 1397460 bytes for the chemotaxis case study. Thus the ratio between the file size for the phase variation and chemotaxis case study is 0.04, respectively the ratio between their average execution times is only 0.37.

Finally the average execution time for the entire specification is computed as the sum of all average execution times (see column 9, Table 4):

$$ \begin{aligned}  \text{Execution time}_{\text{specification}} &= 0:22.04 + 0:11.50 \\ &\quad+ 0:44.87 + 0:9.27 \\ &\quad+ 0:13.73 + 0:22.10 \\ &= 02:3.51\ \text{(minutes:seconds)}. \end{aligned} $$

Similarly to the phase variation case study evaluating the specification comprising all PBLSTL statements in the same model checker run leads to a decrease in the execution time. The average execution time recorded for the entire chemotaxis specification considering 500 runs of the model checker was 0:56.18 (minutes:seconds) i.e. less than 50% of the average execution time when each PBLSTL statement was evaluated separately.

For reproducibility purposes a subdataset of 250 STML files generated on demand and the set of PBLSTL statements for the chemotaxis case study are made available in Additional files 10 and 12. Due to file size constraints the full dataset of 2500 STML files is made available only on the “Case studies” subpage of [48].

## Discussion

The need for spatio-temporal models and corresponding analysis and validation methods was mentioned previously in the literature. An example framework for spatio-temporal modelling and simulation based on the automatic detection and analysis of biochemical species in microscopy images is described in [64]. Most of the existing formal methods employed for the quantitative validation of such models only consider the evolution over time of non-spatial properties such as concentrations. To the best of our knowledge the only existing quantitative spatio-temporal model checking approach is described in [65] for reasoning about uncertainty in epidemiological models. The authors define a Bounded Spatio-Temporal Logic which extends BLTL with two spatial functions *P*(*A*,*C*) for computing the number of type *A* entities present in the compartment *C* and *N*(*A*,*B*,*r*) for computing the number of type *A* entities lying within a radius *r* of one or more type *B* entities. More recently this work was extended in [66] where a probabilistic spatio-temporal specification language called EpiSpec is defined. Compared to the previous approach EpiSpec is based on first-order logic, defines functions with a similar semantics to *P* and *N* and additionally enables the use of potentially complex arithmetic $\left (\text {e.g.} ~\frac {dE}{dt}, \int _{t_{1}}^{t_{2}}{E}{dt}\right)$ expressions. From a spatial point of view in both approaches only the number of entities in a location or the neighbourhood of a location are considered. Thus spatial patterns described by locations or clusters of locations are not detected and analysed. Moreover geometric properties (e.g. area, perimeter, angle etc.) are not considered.

Our methodology is an extension of the existing model checking approaches because it enables the validation of models with respect to (clusters of) spatial patterns and how their geometric properties change over time. The ability to reason about spatial structures and the interactions between such structures proves useful for the automatic *in silico* validation of complex spatio-temporal models. Stochastic biological systems are represented as SSpDESs and the formal specification is encoded in PBLSTL.

The presented methodology and the model checker Mudi have been designed to not place any restrictions on the relevantly employed modelling formalism. In order to illustrate the generalisability of our approach the computational model for the phase variation case study was formalised as a Coloured Stochastic Petri Net, respectively the computational model for the chemotaxis case study as a Cellular Potts model integrated with a system of partial differential equations.

Although Mudi is not dependent on the model type it does place a restriction on the simulation output format. All timeseries data need to be translated to the standard data representation format STML. In our approach this conversion is carried out automatically by the parameterised region (phase variation) and cluster (chemotaxis) detection mechanisms.

The main reason for choosing image processing functions for the translation of timeseries data to STML is that images could be generated from *in silico* simulations but also recorded during wet-lab experiments. Therefore our methodology could potentially be used in the future to automatically determine if certain spatio-temporal properties hold for both *in silico* and *in vitro* generated datasets. Quantifying how many logic statements hold for computational models vs wet-lab datasets could prove to be useful as a measure of similarlity/fitness and therefore be employed in automatic model construction and/or parameter estimation/synthesis algorithms. Although image processing functions were employed here to translate timeseries data to STML the system was designed in a modular fashion such that the model checker Mudi (and the associated binary) is decoupled from the region/cluster detection mechanism (and their implementation). Thus potential users of the model checker could extend our implementation with their own customized timeseries translators.

In addition Mudi supports validating models based on pre-generated STML files (phase variation) or it can generate STML files on demand (chemotaxis). In case STML files are generated on demand a user-defined script calling the model simulator needs to be made available. For the chemotaxis case study a Bash script was created to execute the Linux version of the Morpheus model simulator and translate the simulation output to STML. Although writing scripts for the integration of Mudi with various model simulators requires expert knowledge, the scripts, if designed properly, need to be potentially written only once.

The efficiency and complexity of the methodology was illustrated for the phase variation and chemotaxis case studies by employing only the frequentist statistical model checking algorithm. However Mudi comprises both Bayesian and frequentist, estimate and statistical hypothesis test based model validation approaches. Depending on the availability of prior knowledge and the preferred method to formulate the model validity problem different algorithms could beused.

The scalability of the methodology depends (in)directly on the size and representation of the modelled system. An increase in the size of the system will negatively impact the model simulation time directly, respectively the spatio-temporal analysis and the evaluation of logic properties indirectly. The rate at which the model simulation time changes, with respect to the system size, can vary considerably depending on the employed model representation and simulation algorithm. For instance the systems considered by the phase variation and chemotaxis case studies were of similar size (discretised space of size 101×101 for phase variation, respectively 100×100 for chemotaxis) and complexity but their simulation time was significantly different (average model simulation time was 50 minutes for phase variation, respectively 5 seconds for chemotaxis). In contrast both the spatio-temporal analysis and evaluation of logic properties only depend on the size of the simulation traces and are expected to scale well (polynomially) with respect to the size of the system. Therefore one potential bottleneck, if any, for the scalability of the methodology is the model representation and/or simulation algorithm.

Although the methodology was applied only to uniscale computational models a certain class of multiscale models are supported as well. These are models for which the model checking specification can be decomposed into *n* logic properties such that each logic property corresponds to a single uniscale submodel. In this case the assumption is that, from a model checking point of view, the interactions between different uniscale models are not relevant and can be ignored. If this is true the multiscale model validation task could be executed as a batch of *n* uniscale model validation tasks where the results are aggregated accordingly.

The model checker Mudi and supplementary materials are made freely available on the official webpage [48].

In spite of the above described features our approach has the following limitations. First one of the main assumptions made is that space is represented in pseudo-3D dimensions. This means that models ranging from 0D (time only) to pseudo-3D (time and 2D space including density) are supported without the possibility of explicitly referring to positions on the Oz axis. Therefore the current version of the model checker cannot be employed for full 3D models and spatial properties of such models (e.g. volume, 3D shape). The extension of the methodology to the full 3D scenario would require defining a set of 3D specific spatial properties, including them in the logic PBLSTL and developing algorithms for automatically extracting such spatial properties from 3D images. Second the presented methodology is limited to spatio-temporal uni-scale models i.e. it assumes that all spatial properties correspond to the same spatial scale. However for real life applications there is a need to build and integrate models across multiple temporal and/or spatial scales which are not covered here. Multiple spatial scale models are not currently supported because the methodology does not include a mechanism to explicitly distinguish between spatial patterns from different scales. Finally our approach has been validated only on simulated data but it should be applicable to real life datasets as well. Moreover the usefulness of our methodology was illustrated only on biological case studies. However there is nothing inherent to the methodology which limits it to the biological and/or medical scenarios. Therefore we would like to consider applying this approach to non-biological case studies as well in an attempt to test its applicability limits and/or discover new features which should be included. In the future we would like to extend our methodology and address the limitations presented above.

## Conclusions

In this paper we defined and implemented a methodology for the automatic *in silico* formal validation of computational models using pseudo-3D spatio-temporal model checking. The advantage of this methodology, in contrast to the existing ones, is that it enables validating computational models with respect to both spatial (e.g. area) and numeric (e.g. concentrations) properties, which means it can be employed for small (e.g. intracellular) as well as large (e.g. tissue) scale systems. Implicitly it also enables verifying correlations between changes in spatial properties with respect to numeric properties or viceversa.

We implemented the methodology in the freely available model checking platform Mudi using a cross-platform programming language. No restriction is placed on the type and representation of the computational model because Mudi operates directly on timeseries data. Therefore it can be potentially integrated with most existing model construction workflows. For flexibility purposes Mudi supports both frequentist and Bayesian, estimate and statistical hypothesis testing based probabilistic model checking algorithms.

The efficiency and applicability of the methodology was illustrated based on two biological case studies namely phase variation patterning in bacterial colony growth and the chemotactic aggregation of cells. Although both models were uni-scale, Mudi could be employed for multiscale models by applying it iteratively for each scale without the possibility of relating properties between scales. We would like to address this limitation in the future. Our work is a precursor to the development of more complex multidimensional and multiscale computational models.

## Availability of supporting data

The data sets supporting the results of this article are included within the article and its Additional files.

## Endnotes

^a^ The *clusteredness* is usually measured using what is known in the cluster detection and analysis literature as cluster validity indices. Although there is no index which performs best for all scenarios Silhouette [67] obtains good/best results in the majority of cases according to [68]. The Silhouette value is computed with respect to the regions in all clusters. Thus in our case it could be determined only at cluster detection and analysis time when the information about individual regions is available. At a particular timepoint we associate to a set of clusters a unique Silhouette value which means we could encode it as a numeric state variable in our model.

^b^ The name of the model checker is composed from the uppercase letters in the word MUltiDImensional.

## Additional files

Additional file 1
**Example of a simple SSpDES.** An example illustrating how to construct a SSpDES model for a simple system.

Additional file 2
**Formal BLSTL syntax and semantics definition.** A detailed description of the BLSTL formal language syntax and semantics.

Additional file 3
**Improved frequentist statistical model checking.** A brief description of the proposed frequentist statistical model checking algorithm improvement.

Additional file 4
**Semantics of the considered approximate probabilistic model checking approaches.** A description of the semantics of the considered approximate probabilistic model checking approaches.

Additional file 5
**Well-defined model checking problem.** A proof that the model checking problem is well-defined.

Additional file 6
**Input file containing all PBLSTL statements for the phase variation case study.** The input file containing all PBLSTL statements for the phase variation case study. Any regular text editor can be used to open this file.

Additional file 7
**Model checking results for the phase variation case study.** The results obtained for each model checker execution and PBLSTL statement considering the phase variation case study. For statistical analysis of the results the pdf file can be converted to a Microsoft Word document, using the online converter (http://www.pdfonline.com/pdf-to-word-converter), from which the table of results can be directly copied in a spreadsheet-like software such as Microsoft Excel (or LibreOffice Calc). In case you would like to use statistical software such as R you can save the results from Excel/Calc in csv format and then import them into R.

Additional file 8
**Dataset of STML files for the phase variation case study.** The dataset of one thousand STML files corresponding to the stochastic simulations of the rectangular model for the phase variation case study. We recommend using the freely available 7zip software (Download link: http://www.7-zip.org/download.html) to extract this archive.

Additional file 9
**Chemotaxis model.** Computational model for the chemotaxis case study. Please use the morpheus modelling environment (Download link: http://imc.zih.tu-dresden.de/wiki/morpheus/doku.php?id=download:download) to open this file.

Additional file 10
**Input file containing all PBLSTL statements for the chemotaxis case study.** The input file containing all PBLSTL statements for the chemotaxis case study. Any regular text editor can be used to open this file.

Additional file 11
**Model checking results for the chemotaxis case study.** The results obtained for each model checker execution and PBLSTL statement considering the chemotaxis case study. For statistical analysis of the results the pdf file can be converted to a Microsoft Word document, using the online converter (http://www.pdfonline.com/pdf-to-word-converter), from which the table of results can be directly copied in a spreadsheet-like software such as Microsoft Excel (or LibreOffice Calc). In case you would like to use statistical software such as R you can save the results from Excel/Calc in csv format and then import them into R.

Additional file 12
**Dataset of STML files for the chemotaxis case study.** The subdataset of 250 STML files corresponding to the stochastic model simulations for the chemotaxis case study. We recommend using the freely available 7zip software (Download link: http://www.7-zip.org/download.html) to extract this archive.

## Abbreviations

BLSTL: Bounded linear spatial temporal logic
BNF: Backus-Naur form
PBLSTL: Probabilistic bounded linear spatial temporal logic
SDES: Stochastic discrete event system
SSpDES: Stochastic spatial discrete event system
STML: Spatial temporal markup language
