# Supplementary material for: Automatic validation of computational models using pseudo-3D spatio-temporal model checking
Source: BMC Syst Biol. 2014 Dec 2;8:124. doi: 10.1186/s12918-014-0124-0 (PMC4272535; doi:10.1186/s12918-014-0124-0)
Supplement: Additional file 1 — Example of a simple SSpDES. An example illustrating how to construct a SSpDES model for a simple system. [file 12918_2014_124_MOESM1_ESM.pdf]

Example of a simple stochastic spatial discrete event system (SSpDES)

Let us assume that we would like to model the growth of a population of cells in a fixed size environment. For simplicity purposes let us consider that the environment comprises 2×2 spatial compartments where each compartment can hold at most one cell. Cells can be of two types, wild type (A) or mutant (B). The probability of obtaining a type A (B) offspring cell when a parent cell of type A (B) divides is 70%, respectively 30% if the parent cell is of type B (A). Since each compartment can be occupied by at most one cell, whenever a parent cell divides the offspring cell is displaced to a neighbouring compartment (considering the von Neumann neighbourhood relation). Finally the cell population survival condition is that the concentration of O_2 in the environment is greater or equal to 50%. Each new type A cell reduces the O_2 concentration with 20%, respectively type B cell by 15%.

Although the above described scenario is not realistic for practical applications, it is sufficient to illustrate how a SSpDES model can be constructed for a biological system which evolves in time and space. The reason for strongly constraining the size of the environment and the behaviour of the cells was to limit the number of possible system states such that they can all be explicitly enumerated.

The behaviour of this simple system is characterised at each moment in time by a set of state variables. Spatial state variables of interest are $Cells\_A$ and $Cells\_B$ representing the number of type A, respectively type B cells in the environment. Conversely the numeric state variable O_2 is used to record the concentration of O_2 in the environment. Considering these spatial and numeric state variables the initial state/configuration S_0 of the system is depicted in Figure 1.

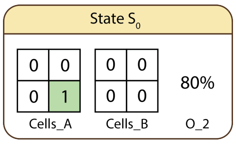

Figure 1: Initial state of the system. $Cells\_A$ and $Cells\_B$ are the spatial state variables representing the number of type A, respectively type B cells in the environment. O_2 represents the current concentration of O_2 in the environment.

Starting from S_0 the system probabilistically transitions from one state to the next until it reaches its final configuration; see Figure 2 for all possible states which can be reached starting from the initial state.

Considering the initial state S_0 the system can transition to four possible states described by the following behaviours: the type A cell from the lower right corner either divides and the offspring is of the same type (S_1, S_3) or of type B (S_2, S_4). In both cases the offspring can be either displaced above the parent (S_3, S_4) or to its left (S_1, S_2). Given that the overall probability of a cell to produce offspring of the same type is 70% and in our case there are 2 relevant state transitions ($S_0 \to S_1$, $S_0 \to S_3$), the probability associated with

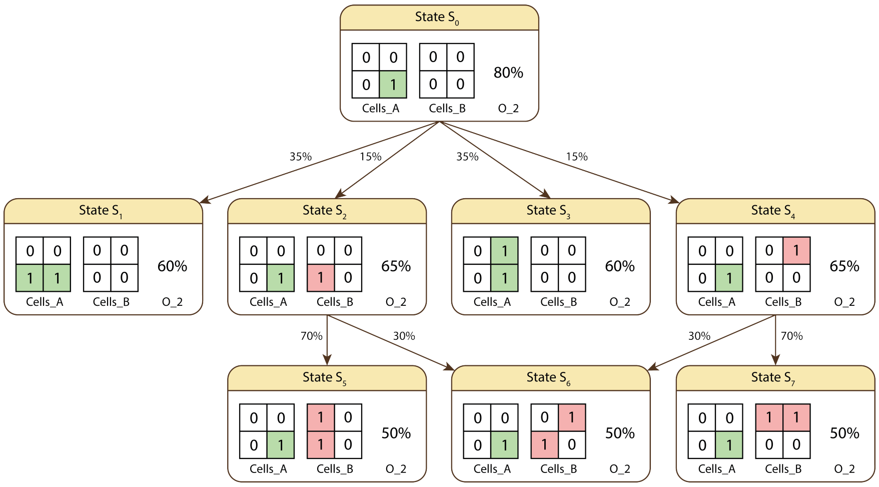

Figure 2: The state space of the system i.e. all possible states which can be reached from the initial state S_0. *Cells_A* and *Cells_B* are the spatial state variables representing the number of type A, respectively type B cells in the environment. *O_2* represents the current concentration of O_2 in the environment. The percentages associated with the arrows connecting each pair of states represents the probability of transitioning from one state to the other.

each of these state transitions is 70% / 2 = 35%. Analogously the probability associated with each state transition where the offspring cell is of different type ($S_0 \rightarrow S_2$, $S_0 \rightarrow S_4$) is equal to 30% / 2 = 15%. The concentration of O_2 has been decreased by 20% in states S_1 and S_3 due to a new type A cell, respectively by 15% in states S_2 and S_4 due to a new type B cell. Therefore the O_2 level is 80% - 20% = 60% in states S_1 and S_3, respectively 80% - 15% = 65% in states S_2 and S_4. Since the birth of a new cell reduces the O_2 concentration by at least 15%, and the minimal O_2 concentration required by the cell population to survive is 50%, no further cellular division can occur starting from states S_1 and S_3 (60% - 15% < 50%). Conversely starting from states S_2 and S_4 at most one new type B cell can be created (65% - 15% $\geq$ 50%). Given state S_2 a type B cell can be produced either from the existing type A ($S_2 \rightarrow S_6$, probability 30%) or type B ($S_2 \rightarrow S_5$, probability 70%) cell. Similarly given state S_4 a type B cell can be produced either from the existing type A ($S_2 \rightarrow S_6$, probability 30%) or type B ($S_2 \rightarrow S_7$, probability 70%) cell.

Using the above descriptions the formal SSpDES $\mathcal{M} = \langle S, T, \mu, NSV, SpSV, NV, SpV \rangle$ corresponding to the system is defined as follows:

- $S = \{S_0, S_1, S_2, S_3, S_4, S_5, S_6, S_7\}$;

- $T = \begin{array}{c} \\ S_0 \\ S_1 \\ S_2 \\ S_3 \\ S_4 \\ S_5 \\ S_6 \\ S_7 \end{array} \begin{array}{c} \begin{array}{cccccccc} S_0 & S_1 & S_2 & S_3 & S_4 & S_5 & S_6 & S_7 \end{array} \\ \begin{pmatrix} 0 & 35\% & 15\% & 35\% & 15\% & 0 & 0 & 0 \\ 0 & 0 & 0 & 0 & 0 & 0 & 0 & 0 \\ 0 & 0 & 0 & 0 & 0 & 70\% & 30\% & 0 \\ 0 & 0 & 0 & 0 & 0 & 0 & 0 & 0 \\ 0 & 0 & 0 & 0 & 0 & 0 & 30\% & 70\% \\ 0 & 0 & 0 & 0 & 0 & 0 & 0 & 0 \\ 0 & 0 & 0 & 0 & 0 & 0 & 0 & 0 \\ 0 & 0 & 0 & 0 & 0 & 0 & 0 & 0 \end{pmatrix} \end{array};$

- μ is the function used to compute the probability of a set of paths starting from S_0. The probability value for a single path is computed by multiplying the probabilities of the state transitions associated with the path. For instance $\mu(S_0, S_2, S_6) = P(S_0, S_2) \cdot P(S_2, S_6) = T[S_0, S_2] \cdot T[S_2, S_6] = 15\% \cdot 30\% = 4.5\%$.

- $NSV = \{O\_2\}$, and NV is the function used to compute the value of O_2 in the current state;

- $SpSV = \{Cells\_A, Cells\_B\}$, and SpV is the function used to evaluate $Cells\_A$ and $Cells\_B$ in the current state.

Although only a simple example was considered here the same modelling principles are employed to construct SSpDES models of more complex (realistic) systems. One of the main differences is that due to the high complexity associated with some real systems the number of possible system states is very large, even potentially infinite. Therefore in such cases explicitly enumerating all possible paths starting from the initial state is not possible.
