# Supplementary material for: Automatic validation of computational models using pseudo-3D spatio-temporal model checking
Source: BMC Syst Biol. 2014 Dec 2;8:124. doi: 10.1186/s12918-014-0124-0 (PMC4272535; doi:10.1186/s12918-014-0124-0)
Supplement: Additional file 2 — Formal BLSTL syntax and semantics definition. A detailed description of the BLSTL formal language syntax and semantics. [file 12918_2014_124_MOESM2_ESM.pdf]

Formal definition of Bounded Linear Spatial Temporal Logic (BLSTL)

Syntax

The syntax of BLSTL will be defined by a context-free grammar using the Backus-Naur Form notation. A definition of a non-terminal symbol (element) in such grammars has the following form:

⟨*defined-element*⟩ ::= ⟨*element1*⟩
  | ⟨*element2*⟩
  | ...

where ::= introduces a new definition and | represents an alternative. In natural language this reads <defined−element> is either an <element1> or <element2> or (...).

In contrast to the BLTL definition the symbol ϕ was replaced by the non-terminal symbol <logic−property>. Furthermore new spatial (e.g. <numeric−spatial−measure>) and arithmetic (e.g. <mean>) functions were introduced.

Definition. *The* syntax *of BLSTL is given by the following grammar formally expressed in Backus-Naur Form:*

⟨logic-property⟩ ::= ⟨numeric-spatial-measure⟩ ⟨comparator⟩ ⟨numeric-measure⟩
  | *⟨numeric-state-variable⟩ ⟨comparator⟩ ⟨numeric-measure⟩*
  | *d(⟨numeric-measure⟩) ⟨comparator⟩ ⟨numeric-measure⟩*
  | ¬ *⟨logic-property⟩*
  | *⟨logic-property⟩* ∨ *⟨logic-property⟩*
  | *⟨logic-property⟩ U[⟨natural-number⟩,⟨natural-number⟩] ⟨logic-property⟩*
  | *X ⟨logic-property⟩*
  | *(⟨logic-property⟩)*

⟨numeric-measure⟩ ::= ⟨numeric-spatial-measure⟩
  | *⟨real-number⟩*
  | *⟨numeric-state-variable⟩*
  | *⟨unary-numeric-measure⟩(⟨numeric-measure⟩)*
  | *⟨binary-numeric-measure⟩(⟨numeric-measure⟩, ⟨numeric-measure⟩)*

⟨numeric-spatial-measure⟩ ::= ⟨unary-subset-measure⟩(⟨subset⟩)
  | *⟨binary-subset-measure⟩(⟨subset⟩, ⟨spatial-measure⟩)*
  | *⟨ternary-subset-measure⟩(⟨subset⟩, ⟨spatial-measure⟩, ⟨real-number⟩)*
  | *⟨quaternary-subset-measure⟩(⟨subset⟩, ⟨spatial-measure⟩, ⟨subset⟩, ⟨spatial-measure⟩)*

⟨unary-subset-measure⟩ ::= count
  | *clusteredness*
  | *density*

⟨binary-subset-measure⟩ ::= avg
  | *geomean*
  | *harmean*
  | *kurt*
  | *max*

| *median*
| *min*
| *mode*
| *product*
| *skew*
| *stdev*
| *sum*
| *var*

⟨*ternary-subset-measure*⟩ *::= percentile*
| *quartile*

⟨*quaternary-subset-measure*⟩ *::= covar*

⟨*unary-numeric-measure*⟩ *::= abs*
| *ceil*
| *floor*
| *round*
| *sign*
| *sqrt*
| *trunc*

⟨*binary-numeric-measure*⟩ *::= add*
| *div*
| *log*
| *mod*
| *multiply*
| *power*
| *subtract*

⟨*subset*⟩ *::=* ⟨*subset-specific*⟩
| *filter(*⟨*subset-specific*⟩*,* ⟨*constraint*⟩*)*

⟨*subset-specific*⟩ *::= regions*
| *clusters*

⟨*constraint*⟩ *::=* ¬ ⟨*constraint*⟩
| ⟨*constraint*⟩ ∨ ⟨*constraint*⟩
| ⟨*spatial-measure*⟩ ⟨*comparator*⟩ ⟨*filter-numeric-measure*⟩

⟨*filter-numeric-measure*⟩ *::=* ⟨*numeric-measure*⟩
| ⟨*spatial-measure*⟩
| ⟨*unary-numeric-measure*⟩*(*⟨*filter-numeric-measure*⟩*)*
| ⟨*binary-numeric-measure*⟩*(*⟨*filter-numeric-measure*⟩*,* ⟨*filter-numeric-measure*⟩*)*

⟨*spatial-measure*⟩ *::= clusteredness*
| *density*
| *area*
| *perimeter*
| *distanceFromOrigin*
| *angle*

| *triangleMeasure*
| *rectangleMeasure*
| *circleMeasure*
| *centroidX*
| *centroidY*

⟨*comparator*⟩ ::= >
| >=
| <
| <=
| =

⟨*numeric-state-variable*⟩ ::= ⟨*state-variable*⟩

⟨*state-variable*⟩ ::= { ⟨*string*⟩ }

⟨*string*⟩ ::= ⟨*character*⟩ | ⟨*character*⟩ ⟨*string*⟩

⟨*character*⟩ ::= *based on the Unicode character set except "{" and "}"*

Similarly to BLTL a set of additional operators can be derived from the definition of the BLSTL syntax using the following equivalences:

1. *Boolean*: (applies to both <logic-property> and <constraint> elements)

$$p \wedge q \equiv \neg(\neg p \vee \neg q)$$
$$p \Rightarrow q \equiv \neg p \vee q$$
$$p \Leftrightarrow q \equiv (p \Rightarrow q) \wedge (q \Rightarrow p)$$

2. *Temporal*:

$$F[a,b]\ p \equiv \text{True}\ U[a,b]\ p$$
$$G[a,b]\ p \equiv \neg F[a,b]\ \neg p$$
$$X[k]\ p \equiv \overbrace{X...X}^{\text{k times}}\ p$$

The order of *precedence* of the operators is given by the definition of the extended BLSTL syntax (see Appendix A). In the *absence* of parentheses the logic expressions are evaluated from left to right.

Semantics

The semantics of BLSTL is defined with respect to executions/simulations of a SSpDES $\mathcal{M}$; see [1, 2] for similar examples. Let us assume that

$$\sigma = (s_0, t_0), (s_1, t_1), ...$$

is an execution of $\mathcal{M}$ along the sequence of states s_0, s_1, ... with t_0, t_1, ... $\in \mathbb{R}$ time durations spent in each state. The execution trace starting at the k-th state is denoted by σ^k, the length of the execution trace by $|\sigma|$, the i-th state of the execution trace by $\sigma[i]$, and the fact that the execution σ satisfies a property

ϕ by $\sigma \models \phi$. For an execution σ at state s the value of a numeric state variable nsv is given by $NV(\sigma, s, nsv)$ and the value of a spatial state variable ssv is given by $SpV(\sigma, s, ssv)$.

In order to have a compact and easy to follow semantics' description the full symbol names provided in the BLSTL syntax definition were replaced with shorter abbreviations as described in Table 1.

Table 1: Translation of full BLSTL symbol names to abbreviated forms. The left column contains the full BLSTL symbol name. The right column contains the corresponding abbreviated form.

**Full BLSTL symbol name**	**Abbreviated BLSTL symbol name**
<numeric-measure>	nm
<unary-numeric-measure>	unm
<binary-numeric-measure>	bnm
<numeric-spatial-measure>	nsm
<unary-subset-measure>	usm
<binary-subset-measure>	bsm
<ternary-subset-measure>	tsm
<quaternary-subset-measure>	qsm
<subset>	ss
<spatial-measure>	sm
<filter-numeric-measure>	fnm
<comparator>	$\asymp$
<numeric-state-variable>	nsv
<spatial-state-variable>	ssv
<meta-spatial-state-variable>	$mssv$
<logic-property>	ψ
<real-number>	r
<natural-number>	n

Definition. *Let* $\mathcal{M} = \langle S, T, \mu, NSV, SpSV, NV, SpV \rangle$ *be a SSpDES and* σ *an execution of* $\mathcal{M}$*. The* semantics *of BLSTL for* σ *is defined as follows:*

- $\sigma \models nsm \asymp nm$ **if and only if** $nsm \asymp nm$*, where* nsm *and* $nm \in \mathbb{R}$ *and* $\asymp \in \{>, >=, <, <=, =\}$*;*
- $\sigma \models nsv \asymp nm$ **if and only if** $NV(\sigma, \sigma[0], nsv) \asymp nm$*, where* $nsv \in NSV$*,* $nm \in \mathbb{R}$ *and* $\asymp \in \{>, >=, <, <=, =\}$*;*
- $\sigma \models d(nm1) \asymp nm2$ **if and only if** $|\sigma| > 1$ *and* $(nm1^1 - nm1^0) \asymp nm2$*, where* $nm1^i \in \mathbb{R}$ *represents the value of the numeric measure* $nm1$ *considering the execution suffix* σ^i *and* $\asymp \in \{>, >=, <, <=, =\}$*.*
- $\sigma \models \neg\psi$ **if and only if** $\sigma \not\models \psi$*;*
- $\sigma \models \psi_1 \vee \psi_2$ **if and only if** $\sigma \models \psi_1$ *or* $\sigma \models \psi_2$*;*
- $\sigma \models \psi_1\ U[a, b]\ \psi_2$ **if and only if** *there exists* i*,* $a \leq i \leq b$ *such that* $\sigma^i \models \psi_2$*, and for all* j*,* $a \leq j < i$*,* $\sigma^j \models \psi_1$*;*

- $\sigma \models X\psi$ **if and only if** $|\sigma| > 1$ *and* $\sigma^1 \models \psi$*;*
- $\sigma \models (\psi)$ **if and only if** $\sigma \models \psi$*;*

The nm symbol represents the category of real-valued numeric measures. Considering a given execution σ, nm is evaluated according to one of the definitions described below:

- **Numeric spatial measure:** $nm = nsm$;
- **Real number:** $nm = r \in \mathbb{R}$;
- **Numeric state variable:** $nm = NV(\sigma, \sigma[0], nsv)$ where nsv is a numeric state variable;
- **Unary numeric measure:** $nm = unm(nm')$ where nm' is a numeric measure;
- **Binary numeric measure:** $nm = bnm(nm', nm'')$ where nm' and nm'' are numeric measures.

The values of the *unary* (unm) and *binary* (bnm) *numeric measures* are computed as described in Appendix B.

The nsm symbol represents the category of numeric (real-valued) spatial measures. Considering a given execution σ, nsm is evaluated according to one of the definitions described below:

- **Unary subset measure:** $nsm = usm(ss)$ where ss is a subset of the considered spatial entities (clusters or regions);
- **Binary subset measure:** $nsm = bsm(ss, sm)$ where ss is a subset of the considered spatial entities (clusters or regions) and sm is a spatial measure;
- **Ternary subset measure:** $nsm = tsm(ss, sm, r)$ where ss is a subset of the considered spatial entities (clusters or regions), sm is a spatial measure and r is a real value;
- **Quaternary subset measure:** $nsm = qsm(ss, sm, ss', sm')$ where ss and ss' are subsets of the considered spatial entities (clusters or regions), and sm and sm' are spatial measures;

In case the considered subset of spatial entities is empty the numeric spatial measures are evaluated to zero.

Spatial measures sm are defined over the set {clusteredness, density, area, perimeter, distanceFromOrigin, angle, triangleMeasure, rectangleMeasure, circleMeasure, centroidX, centroidY} which is identical to the set of spatial measures recorded in an STML file for each detected region/cluster.

The value of *unary* (usm), *binary* (bsm), *ternary* (tsm) and *quaternary* (qsm) *subset measures* are computed as described in Appendix C. Some of the binary and all ternary and quaternary subset measures are statistical functions which can be employed for reasoning about the distribution of the regions/-clusters measures at a particular time point. In contrast to traditional logic formalisms BLSTL allows specifying properties of both single spatial properties and/or distributions of spatial properties.

Subsets of the collections of regions/clusters are represented by the ss symbol. Considering a given execution σ, ss is evaluated according to one of the definitions described below:

- **Specific subset:** $ss = specificSubset$ where $specificSubset$ represents either the collection of all clusters (see Definition 4) or the collection of all regions (see Definition 3) corresponding to $\sigma[0]$;
- **Filtered specific subset:** $ss = filter(specificSubset, constraints)$ where $specificSubset$ has the semantics defined above, and $constraints$ is a set of logic properties restricting the considered spatial entities to a subset of $specificSubset$;

Given an execution σ the value of the $specificSubset$ symbol is computed using one of the definitions described below:

- **Regions:** $specificSubset = \bigcup_{ssv \in SpSV} \{region \mid region \in \text{regionDetectionMechanism(ssv)}\}, \forall ssv \in SpSV$ considering the state $\sigma[0]$;
- **Clusters:** $specificSubset = \text{clustersDetectionMechanism}(se)$, where $se = \bigcup_{ssv \in SpSV} \{region \mid region \in \text{regionDetectionMechanism(ssv)}\}, \forall ssv \in SpSV$ considering the state $\sigma[0]$;

Subsets of the collection returned by $specificSubset$ can be computed using the $filter$ predicate. Considering an execution σ $filter$ is evaluated using the definition described below:

$$filter = \{e \in specificSubset \mid e \models c, \forall c \in constraints\}.$$

The semantics of the constraint satisfaction problem considering a region/cluster e and a constraint c is defined below:

- $e \models sm \asymp fnm$ **if and only if** $sm(e) \asymp fnm$, where $sm(e)$ evaluates the spatial measure sm for the given spatial entity e, $sm \in$ {clusteredness, density, area, perimeter, distanceFromOrigin, angle, triangleMeasure, rectangleMeasure, circleMeasure, centroidX, centroidY}, fnm is a filter numeric measure $\in \mathbb{R}$ and $\asymp \in \{>, >=, <, <=, =\}$;
- $e \models \neg c$ **if and only if** $e \not\models c$;
- $e \models c_1 \vee c_2$ **if and only if** $e \models c_1$ or $e \models c_2$;

The fnm symbol represents the (real-valued) numeric measure computed for the $filter$ predicate. Given an execution σ and a region/cluster e, the value of fnm is computed using one of the definitions given below:

- **Numeric measure:** $fnm = nm$ where nm is a numeric measure;
- **Spatial measure:** $fnm = sm(e)$ where $sm \in$ {clusteredness, density, area, perimeter, distanceFromOrigin, angle, triangleMeasure, rectangleMeasure, circleMeasure, centroidX, centroidY};
- **Unary filter numeric measure:** $fnm = unm(fnm')$ where fnm' is a filter numeric measure;

- **Binary filter numeric measure:** $fnm = bnm(fnm', fnm'')$ where fnm' and fnm'' are filter numeric measures.

The *extended semantics* of BLSTL for an execution σ (corresponding to the extended syntax of BLSTL) additionally includes the following rules:

1. *Boolean*:

   - $\sigma \models \psi_1 \wedge \psi_2$ **if and only if** $\sigma \models \psi_1$ and $\sigma \models \psi_2$;
   - $\sigma \models \psi_1 \Rightarrow \psi_2$ **if and only if** $\sigma \models \neg\psi_1$ or $\sigma \models \psi_2$;
   - $\sigma \models \psi_1 \Leftrightarrow \psi_2$ **if and only if** $\sigma \models \psi_1 \Rightarrow \psi_2$ and $\sigma \models \psi_2 \Rightarrow \psi_1$;

2. *Temporal*:

   - $\sigma \models F[a,b]\ \psi$ **if and only if** there exists i, $a \leq i \leq b$ such that $\sigma^i \models \psi$;
   - $\sigma \models G[a,b]\ \psi$ **if and only if** for all i, $a \leq i \leq b$, $\sigma^i \models \psi$;
   - $\sigma \models X[k]\ \psi$ **if and only if** $|\sigma| > k$ and $\sigma^k \models \psi$;

Conversely the *extended semantics* of BLSTL for a spatial entity e and constraint c (corresponding to the extended syntax of BLSTL) additionally includes the following Boolean rules:

- $e \models c_1 \wedge c_2$ **if and only if** $e \models c_1$ and $e \models c_2$;
- $e \models c_1 \Rightarrow c_2$ **if and only if** $e \models \neg c_1$ or $e \models c_2$;
- $e \models c_1 \Leftrightarrow c_2$ **if and only if** $e \models c_1 \Rightarrow c_2$ and $e \models c_2 \Rightarrow c_1$;

References

[1] Sumit K. Jha, Edmund M. Clarke, Christopher J. Langmead, Axel Legay, André Platzer, and Paolo Zuliani. A bayesian approach to model checking biological systems. In Pierpaolo Degano and Roberto Gorrieri, editors, *Computational Methods in Systems Biology*, number 5688 in Lecture Notes in Computer Science, pages 218–234. Springer Berlin Heidelberg, Bologna, Italy, January 2009.

[2] Paolo Zuliani, André Platzer, and Edmund M. Clarke. Bayesian statistical model checking with application to Simulink/Stateflow verification. In *Proceedings of the 13th ACM international conference on Hybrid systems: computation and control*, HSCC '10, page 243–252, New York, NY, USA, 2010. ACM.

Appendices

Appendix A Extended BLSTL syntax

Definition. *Let* $\mathcal{M} = \langle S, T, \mu, NSV, SpSV, NV, SpV \rangle$ *be a SSpDES. The* extended syntax *of BLSTL is given by the following grammar expressed in Backus-Naur Form (BNF):*

⟨logic-property⟩ ::= ⟨numeric-spatial-measure⟩ ⟨comparator⟩ ⟨numeric-measure⟩
| ⟨numeric-state-variable⟩ ⟨comparator⟩ ⟨numeric-measure⟩
| d(⟨numeric-measure⟩) ⟨comparator⟩ ⟨numeric-measure⟩
| ¬ ⟨logic-property⟩
| ⟨logic-property⟩ ∧ ⟨logic-property⟩
| ⟨logic-property⟩ ∨ ⟨logic-property⟩
| ⟨logic-property⟩ ⇒ ⟨logic-property⟩
| ⟨logic-property⟩ ⇔ ⟨logic-property⟩
| ⟨logic-property⟩ U[⟨natural-number⟩,⟨natural-number⟩] ⟨logic-property⟩
| F[⟨natural-number⟩,⟨natural-number⟩] ⟨logic-property⟩
| G[⟨natural-number⟩,⟨natural-number⟩] ⟨logic-property⟩
| X ⟨logic-property⟩
| X[⟨natural-number⟩] ⟨logic-property⟩
| (⟨logic-property⟩)

⟨numeric-measure⟩ ::= ⟨numeric-spatial-measure⟩
| ⟨real-number⟩
| ⟨numeric-state-variable⟩
| ⟨unary-numeric-measure⟩(⟨numeric-measure⟩)
| ⟨binary-numeric-measure⟩(⟨numeric-measure⟩, ⟨numeric-measure⟩)

⟨numeric-spatial-measure⟩ ::= ⟨unary-subset-measure⟩(⟨subset⟩)
| ⟨binary-subset-measure⟩(⟨subset⟩, ⟨spatial-measure⟩)
| ⟨ternary-subset-measure⟩(⟨subset⟩, ⟨spatial-measure⟩, ⟨real-number⟩)
| ⟨quaternary-subset-measure⟩(⟨subset⟩, ⟨spatial-measure⟩, ⟨subset⟩, ⟨spatial-measure⟩)

⟨unary-subset-measure⟩ ::= count
| clusteredness
| density

⟨binary-subset-measure⟩ ::= avg
| geomean
| harmean
| kurt
| max
| median
| min
| mode
| product
| skew
| stdev

| *sum*
| *var*

⟨*ternary-subset-measure*⟩ ::= *percentile*
| *quartile*

⟨*quaternary-subset-measure*⟩ ::= *covar*

⟨*unary-numeric-measure*⟩ ::= *abs*
| *ceil*
| *floor*
| *round*
| *sign*
| *sqrt*
| *trunc*

⟨*binary-numeric-measure*⟩ ::= *add*
| *div*
| *log*
| *mod*
| *multiply*
| *power*
| *subtract*

⟨*subset*⟩ ::= ⟨*subset-specific*⟩
| *filter(*⟨*subset-specific*⟩, ⟨*constraint*⟩*)*

⟨*subset-specific*⟩ ::= *regions*
| *clusters*

⟨*constraint*⟩ ::= ¬ ⟨*constraint*⟩
| ⟨*constraint*⟩ ∧ ⟨*constraint*⟩
| ⟨*constraint*⟩ ∨ ⟨*constraint*⟩
| ⟨*constraint*⟩ ⇒ ⟨*constraint*⟩
| ⟨*constraint*⟩ ⇔ ⟨*constraint*⟩
| ⟨*spatial-measure*⟩ ⟨*comparator*⟩ ⟨*filter-numeric-measure*⟩
| *(*⟨*constraint*⟩*)*

⟨*filter-numeric-measure*⟩ ::= ⟨*numeric-measure*⟩
| ⟨*spatial-measure*⟩
| ⟨*unary-numeric-measure*⟩*(*⟨*filter-numeric-measure*⟩*)*
| ⟨*binary-numeric-measure*⟩*(*⟨*filter-numeric-measure*⟩, ⟨*filter-numeric-measure*⟩*)*

⟨*spatial-measure*⟩ ::= *clusteredness*
| *density*
| *area*
| *perimeter*
| *distanceFromOrigin*
| *angle*
| *triangleMeasure*
| *rectangleMeasure*

| *circleMeasure*
| *centroidX*
| *centroidY*

⟨*real-number*⟩ ::= ⟨*unsigned-real-number*⟩
| ⟨*sign*⟩ ⟨*unsigned-real-number*⟩

⟨*unsigned-real-number*⟩ ::= ⟨*fractional-part*⟩
| ⟨*fractional-part*⟩ ⟨*exponent-part*⟩

⟨*fractional-part*⟩ ::= ⟨*digit-sequence*⟩ . ⟨*digit-sequence*⟩
| . ⟨*digit-sequence*⟩
| ⟨*digit-sequence*⟩ .
| ⟨*natural-number*⟩

⟨*digit-sequence*⟩ ::= ⟨*digit*⟩
| ⟨*digit*⟩ ⟨*digit-sequence*⟩

⟨*digit*⟩ ::= *0*
| *1*
| *2*
| *3*
| *4*
| *5*
| *6*
| *7*
| *8*
| *9*

⟨*natural-number*⟩ ::= ⟨*digit-sequence*⟩
| + ⟨*digit-sequence*⟩

⟨*exponent-part*⟩ ::= *e* ⟨*digit-sequence*⟩
| *E* ⟨*digit-sequence*⟩
| *e* ⟨*sign*⟩ ⟨*digit-sequence*⟩
| *E* ⟨*sign*⟩ ⟨*digit-sequence*⟩

⟨*sign*⟩ ::= +
| -

⟨*comparator*⟩ ::= >
| >=
| <
| <=
| =

⟨*numeric-state-variable*⟩ ::= ⟨*state-variable*⟩

⟨*state-variable*⟩ ::= {⟨*string*⟩}

⟨*string*⟩ ::= ⟨*character*⟩ | ⟨*character*⟩ ⟨*string*⟩

$\langle$*character*$\rangle$ *::= based on the Unicode character set except "{" and "}"*

Appendix B Numeric measures

Table 2: Name, description and semantics of unary numeric measures

Name	Description	Semantics
abs	Returns the absolute value of a number	$abs(n) = \|n\|$
ceil	Rounds the number upward, returning the smallest integral value that is not less than the number	$ceil(n) = \lceil n \rceil$
floor	Rounds the number downward, returning the largest integral value that is not greater than the number	$floor(n) = \lfloor n \rfloor$
round	Returns the integral value that is nearest to x, with halfway cases rounded away from zero	$round(n) = \begin{cases} \lfloor n + 0.5 \rfloor, & \text{if } n \geq 0 \\ \lceil n - 0.5 \rceil, & \text{otherwise} \end{cases}$
sign	Returns the sign of a number	$sign(n) = \begin{cases} 1, & \text{if } n > 0 \\ 0, & \text{if } n = 0 \\ -1, & \text{otherwise} \end{cases}$
sqrt	Returns the square root of a number	$sqrt(n) = \sqrt{n}$
trunc	Rounds number toward zero, returning the nearest integral value that is not larger in magnitude than the number	$trunc(n) = sign(n)\lfloor \|n\| \rfloor$

Table 3: Name, description and semantics of binary numeric measures

Name	Description	Semantics
add	Returns the sum of two numbers	$add(n_1, n_2) = n_1 + n_2$
div	Returns the integer part of a division	$div(n_1, n_2) = \lfloor n_1/n_2 \rfloor$
log	Returns the logarithm of a number in the given base	$log(n, b) = \log_b(\lvert n \rvert), n > 0, b > 0, b \neq 1$
mod	Returns the remainder of a division	$mod(n_1, n_2) = n_1 - (n_2 \times div(n_1, n_2))$
multiply	Return the multiplication of two numbers	$multiply(n_1, n_2) = n_1 \times n_2$
power	Returns the base raised at the power exponent	$pow(b, e) = b^e$
subtract	Returns the difference between two numbers	$subtract(n_1, n_2) = n_1 - n_2$

Appendix C Subset measures

Table 4: Name, description and semantics of unary subset measures

Name	Description	Semantics
count	Returns the number of clusters/regions	$count(subset) = \lvert subset \rvert$
clusteredness	Returns the clusteredness of a set of clusters/regions	As described in the main manuscript
density	Returns the density of a set of clusters/regions	As described in the main manuscript

Table 5: Name, description and semantics of binary subset measures

Name	Description	Semantics
avg	Returns the arithmetic mean considering the given *subset* and spatial measure *sm*	$avg(subset, sm) = \frac{1}{n} \sum_{i=1}^{n} sm(subset_i)$, where $n = \lvert subset \rvert$

geomean	Returns the geometric mean considering the given *subset* and spatial measure *sm*	$geomean(subset, sm) = \left(\prod_{i=1}^{n} sm(subset_i)\right)^{\frac{1}{n}}$, where $n = \|subset\|$
harmean	Returns the harmonic mean considering the given *subset* and spatial measure *sm*	$harmean(subset, sm) = \frac{n}{\sum_{i=1}^{n} \frac{1}{sm(subset_i)}}$, where $n = \|subset\|$
kurt	Returns the kurtosis considering the given *subset* and spatial measure *sm*	$kurt(subset, sm) = \frac{n(n+1)}{(n-1)(n-2)(n-3)} \sum_{i=1}^{n} \left(\frac{sm(subset_i)-avg(subset,sm)}{stdev(subset,sm)}\right)^4 - \frac{3(n-1)^2}{(n-2)(n-3)}$, where $n = \|subset\|$ and $n \geq 4$
max	Returns the maximum considering the given *subset* and spatial measure *sm*	$max(subset, sm) = \max_{i=1,n} sm(subset_i)$, where $n = \|subset\|$
median	Returns the median considering the given *subset* and spatial measure *sm*	$median(subset, sm)$ = middle value in the ordered list of spatial measures
min	Returns the minimum considering the given *subset* and spatial measure *sm*	$min(subset, sm) = \min_{i=1,n} sm(subset_i)$, where $n = \|subset\|$
mode	Returns the mode considering the given *subset* and spatial measure *sm*	$mode(subset, sm)$ = value which appears most often in the list of spatial measures
product	Returns the product considering the given *subset* and spatial measure *sm*	$product(subset, sm) = \prod_{i=1}^{n} sm(subset_i)$, where $n = \|subset\|$
skew	Returns the skewness considering the given *subset* and spatial measure *sm*	$skew(subset, sm) = \frac{n}{(n-1)(n-2)} \sum_{i=1}^{n} \left(\frac{sm(subset_i)-avg(subset,sm)}{stdev(subset,sm)}\right)^3$, where $n = \|subset\|$
stdev	Returns the standard deviation considering the given *subset* and spatial measure *sm*	$stdev(subset, sm) = \sqrt{\frac{\sum_{i=1}^{n} (sm(subset_i)-avg(subset,sm))^2}{(n-1)}}$, where $n = \|subset\|$
sum	Returns the sum considering the given *subset* and spatial measure *sm*	$sum(subset, sm) = \sum_{i=1}^{n} sm(subset_i)$, where $n = \|subset\|$

var	Returns the variance considering the given *subset* and spatial measure *sm*	$var(subset, sm) = \frac{\sum_{i=1}^{n} (sm(subset_i) - avg(subset, sm))^2}{(n-1)}$, where $n = \lvert subset \rvert$

Table 6: Name, description and semantics of ternary subset measures

Name	Description	Semantics
percentile	Returns the pv-th ($0 \leq pv \leq 100$) percentile considering the given *subset* and spatial measure *sm*	$percentile(subset, sm, pv) = i$-th spatial measure considering an ordered list of N spatial measures, N is the number of regions/clusters and $i = \lfloor \frac{pv}{100} \times N + \frac{1}{2} \rfloor$
quartile	Returns the i-th ($i \in \{25, 50, 75\}$) quartile considering the given *subset* and spatial measure *sm*	Let v be the ordered list of spatial measures, $m = median(subset, sm)$, L the sublist of values in v smaller than m and U the sublist of values in v greater than m. $quartile(subset, sm, i) = median$ of L if $i = 25$, m if $i = 50$, and *median* of U if $i = 75$.

Table 7: Name, description and semantics of quaternary subset measures

Name	Description	Semantics
covar	Returns the covariance considering $subset_1$ and $subset_2$, and the spatial measures sm_1 and sm_2	$covar(subset_1, sm_1, subset_2, sm_2) = \frac{1}{N-1} \sum_{i=1}^{N} ((sm_1(subset_1[i])$ - $mean(subset_1, sm_1))$ * $(sm_2(subset_2[i])$ - $mean(subset_2, sm_2))$), where $N = min(\|subset_1\|, \|subset_2\|)$
