# Supplementary material for: Automatic validation of computational models using pseudo-3D spatio-temporal model checking
Source: BMC Syst Biol. 2014 Dec 2;8:124. doi: 10.1186/s12918-014-0124-0 (PMC4272535; doi:10.1186/s12918-014-0124-0)
Supplement: Additional file 3 — Improved frequentist statistical model checking. A brief description of the proposed frequentist statistical model checking algorithm improvement. [file 12918_2014_124_MOESM3_ESM.pdf]

Improved frequentist statistical model checking

The algorithms OSM A/B described in [1] contain an invalid initialisation. First of all the notations relevant for describing the initialisation error will be explained. Then a brief proof will be provided illustrating the presence of the error followed by the proposed solution which is considered in our approach. Let us assume for the remainder of this section that the logic property to be verified ϕ is of the form $P_{\bowtie\theta}[\psi], \theta \in (0, 1)$.

Notations

The width of the indifference region (p_1, p_0) is equal to 2δ where

$$p_1 = \theta - \delta$$
$$p_0 = \theta + \delta$$

and $0 \leq \delta \leq 1$. Using δ two values f_n and f'_n are computed to decide if ϕ holds:

$$\begin{aligned} f_n &= d\left(\log\frac{\theta-\delta}{\theta}\right) + (n-d)\left(\log\frac{1-(\theta-\delta)}{1-\theta}\right) \\ f'_n &= d\left(\log\frac{\theta}{\theta+\delta}\right) + (n-d)\left(\log\frac{1-\theta}{1-(\theta+\delta)}\right) \end{aligned} \tag{1}$$

where n represents the total number of evaluated model simulations and d the number of simulations for which ϕ evaluated true.

The nominator and denominator of each fraction in Equation 1 represents a probability value $\in (0, 1)$. This additionally ensures that the values provided to the logarithms are positive and therefore valid. Thus the following inequalities must hold:

$$0 < \theta - \delta < 1; \tag{2a}$$

$$0 < \theta < 1; \tag{2b}$$

$$0 < 1 - (\theta - \delta) < 1; \tag{2c}$$

$$0 < 1 - \theta < 1; \tag{2d}$$

$$0 < \theta + \delta < 1; \tag{2e}$$

$$0 < 1 - (\theta + \delta) < 1; \tag{2f}$$

Considering that $\theta \in (0, 1)$ the δ independent Inequalities 2b and 2d hold always.

Description of initialisation error

The cause of the error in the OSM A/B algorithm is the initialisation of δ with the value 1. We will prove this by assuming that the initialisation is valid ($\delta = 1$) and employing proof by contradiction.

Proof. The first instruction in the OSM A/B algorithm is the initialisation of δ with the value 1. This value is then passed to the `IncrementalYounesB` function. After computing the value of four variables (A_1, B_1, A_2, B_2) the main `repeat` loop is entered. A new sample is generated and evaluated, and the

values of the variables n and d are updated. Afterwards the values of f_n and f'_n are computed according to Equation 1. In order to show that the initialisation of δ is invalid it is sufficient to show that one of the Inequalities in Equation 2 does not hold. According to Inequality 2a:

$$0 < \theta - \delta < 1.$$

In our case $\delta = 1$. Therefore Inequality 2a is evaluated as follows:

$$
\begin{aligned}
& 0 < \theta - \delta < 1 \\
& \Leftrightarrow && \text{(replace } \delta \text{ with 1)} \\
& 0 < \theta - 1 < 1 \\
& \Leftrightarrow && (+1) \\
& 0 + 1 < \theta - 1 + 1 < 1 + 1 \\
& \Leftrightarrow && \text{(evaluate arithmetic expressions)} \\
& 1 < \theta < 2 \\
& \Rightarrow && \text{(conclusion from inequality)} \\
& \theta \in (1, 2).
\end{aligned}
$$

However $\theta \in (0, 1)$ which contradicts $\theta \in (1, 2)$. Hence the initialisation $\delta = 1$ is invalid. □

As a side note one of the arithmetic expressions which are invalid when δ is initialised with the value 1 is $\log \frac{\theta-\delta}{\theta}$ because $\theta - \delta < 0$ which means that a negative value is provided to the logarithm.

Solution

In both OSM A/B algorithms the width of the indifference region 2δ is reduced to half whenever an *undecided* result is obtained. The method employed by the algorithms is to start with the maximum valid δ value and then decrease it until a true/false result can be obtained.

The domain of all valid δ values is computed based on Inequalities 2a-2f; see Table 1 for the interval of valid δ values computed for each inequality.

Table 1: The valid interval of δ values corresponding to Inequalities 2a-2f

**Nr.**	**Inequality**	**Valid interval of δ values**
2a	$0 < \theta - \delta < 1$	$\delta \in (\theta - 1, \theta) \cap (0, 1) = (0, \theta)$
2b	$0 < \theta < 1$	$\delta \in (0, 1)$
2c	$0 < 1 - (\theta - \delta) < 1$	$\delta \in (\theta - 1, \theta) \cap (0, 1) = (0, \theta)$
2d	$0 < 1 - \theta < 1$	$\delta \in (0, 1)$
2e	$0 < \theta + \delta < 1$	$\delta \in (-\theta, 1-\theta) \cap (0, 1) = (0, 1-\theta)$
2f	$0 < 1 - (\theta + \delta) < 1$	$\delta \in (-\theta, 1-\theta) \cap (0, 1) = (0, 1-\theta)$

By intersecting all intervals provided in Table 1 the domain of valid δ values is obtained:

$$D_\delta = (0,\theta) \cap (0,1) \cap (0,\theta) \cap (0,1) \cap (0,1-\theta) \cap (0,1-\theta)$$

$$\Leftrightarrow \quad \text{(remove duplicates)}$$

$$D_\delta = (0,\theta) \cap (0,1) \cap (0,1-\theta)$$

$$\Leftrightarrow \quad \text{(remove enclosing interval (0, 1))}$$

$$D_\delta = (0,\theta) \cap (0,1-\theta)$$

which is equivalent to

$$D_\delta = (0, \min(\theta, 1-\theta)).$$

The maximum value δ_{init} defined in D_δ should be employed during the initialisation step of the improved statistical model checking algorithm. Thus

$$\delta_{init} = \max_{v \in D_\delta}(v)$$

$$\Leftrightarrow \quad \text{(expand rhs. of the equation)}$$

$$\delta_{init} = \max_{v \in (0, \min(\theta, 1-\theta))}(v)$$

$$\Leftrightarrow \quad \text{(0 and } \min(\theta, 1-\theta) \text{ can not be considered)}$$

$$\delta_{init} = \min(\theta, 1-\theta) - \epsilon$$

where $0 < \epsilon \ll \min(\theta, 1-\theta)$. For implementation purposes the value of ϵ can be chosen as follows:

$$0 < \epsilon = \frac{1}{k}\min(\theta, 1-\theta) < \min(\theta, 1-\theta)$$

where $k \gg 1$ is a user-defined or hard coded finite constant. Thus

$$\delta_{init} = \min(\theta, 1-\theta) - \frac{1}{k}\min(\theta, 1-\theta)$$

$$\Leftrightarrow \quad \text{(arithmetic operations)}$$

$$\delta_{init} = \frac{k}{k}\min(\theta, 1-\theta) - \frac{1}{k}\min(\theta, 1-\theta)$$

$$\Leftrightarrow \quad \text{(arithmetic operations)}$$

$$\boxed{\delta_{init} = \frac{k-1}{k}\min(\theta, 1-\theta)}.$$

Proving that $\delta_{init} \in D_\delta$ for a finite constant value $k \gg 1$ is trivial. Moreover

$$\lim_{k\to\infty} \delta_{init}$$

$=$ (replace δ_{init} with its value)

$$\lim_{k\to\infty} \frac{k-1}{k} \cdot \min(\theta, 1-\theta)$$

$=$ (extract k independent terms outside limit)

$$\min(\theta, 1-\theta) \cdot \lim_{k\to\infty} \frac{k-1}{k}$$

$=$ (compute value of limit)

$$\min(\theta, 1-\theta) \cdot 1$$

$=$ (evaluate arithmetic expression)

$$\min(\theta, 1-\theta).$$

The value of δ_{init} gets closer to $\min(\theta, 1-\theta)$ as the value of k approaches ∞. Thus high values of k should be employed in the implementation.

References

[1] Chuan Hock Koh, Sucheendra K. Palaniappan, P. S. Thiagarajan, and Limsoon Wong. Improved statistical model checking methods for pathway analysis. *BMC Bioinformatics*, 13(Suppl 17):S15, December 2012. PMID: 23282174.
