# Supplementary material for: Automatic validation of computational models using pseudo-3D spatio-temporal model checking
Source: BMC Syst Biol. 2014 Dec 2;8:124. doi: 10.1186/s12918-014-0124-0 (PMC4272535; doi:10.1186/s12918-014-0124-0)
Supplement: Additional file 4 — Semantics of the considered approximate probabilistic model checking approaches. A description of the semantics of the considered approximate probabilistic model checking approaches. [file 12918_2014_124_MOESM4_ESM.pdf]

Semantics of considered approximate probabilistic model checking approaches

Approximate or simulation-based probabilistic model checking approaches employ methods from statistical theory to decide if logic properties hold for a model with a certain confidence level. In contrast to exhaustive approaches the state space is only partially explored. The truth value of logic properties is approximated based on a finite set of simulations.

Let us assume that a logic property ϕ is verified against a model $\mathcal{M}$. For each simulation of the model $\mathcal{M}$ the logic property ϕ evaluates to *true* with a probability p, respectively *false* with probability $1 - p$.

Therefore each simulation can be represented by a *Bernoulli* variable X which takes the value 1 with probability p (success) and 0 with probability $1-p$ (failure). Moreover n simulations can be represented by a sequence of independent, identically distributed (iid) Bernoulli variables $X_1, X_2, ..., X_n$, where each X_i is a Bernoulli variable with the success probability p. The sum of a sequence of iid Bernoulli variables $Y = \sum_{i=1}^{n} X_i$ is a random variable that follows a *binomial* distribution with parameters n and p.

Based on the Bernoulli or binomial distribution representation many approximate model checkers have been developed. The considered approximate probabilistic approaches are given in Table 1 and a brief description of each approach in the following Subsections.

Table 1: Classification of considered approximate probabilistic model checking approaches

	**Frequentist**	**Bayesian**
**Estimate**	Chernoff-Hoeffding bounds	Mean and variance
**Hypothesis testing**	Statistical<br>Probabilistic black-box	Statistical

Simulation evaluations are represented as a sequence $X_1, X_2, ..., X_n$ of iid Bernoulli variables in all model checking approaches described below.

Chernoff-Hoeffding bounds based model checking

Approximate probabilistic model checking [2] is a simulation-based approach which estimates the true probability p of a logic property being true.

The approximation error of the method is controlled using a derived form of the Chernoff-Hoeffding inequalities [3]:

$$P[\mid \overline{X} - p \mid > \epsilon] < 2e^{-\frac{N\epsilon^2}{4}} \quad (1)$$

where $\overline{X}$ is the sample mean

$$\overline{X} = \frac{1}{n}\sum_{i=1}^{n} X_i$$

and $0 < \epsilon < 1$.

Equation 1 states that the probability of $\overline{X}$ to deviate from the true probability p more than ϵ is bounded above by $\delta = 2e^{-\frac{N\epsilon^2}{4}}$. The number of simulations n required to meet the constraints of Equation 1 is computed with respect to parameters ϵ and δ:

$$n = \frac{4}{\epsilon^2} log(\frac{2}{\delta})$$

where $0 < \epsilon, \delta < 1$. Therefore ϵ and δ are input parameters of the algorithm.

A detailed description of the approach accompanied by examples are provided in [2]. From the point of view of the computational complexity the algorithm is *linear* with respect to the value of δ and *quadratic* with respect to the value of ϵ.

Frequentist statistical model checking

Frequentist statistical model checking methods [11, 13] verify if a logical property ϕ holds for a model $\mathcal{M}$ using acceptance sampling tests [14]. An implicit requirement of this method is that a model which can be simulated on demand is available.

Let us assume that ϕ is a logic property of the form $P_{\geq\theta}[\psi]$. The null hypothesis $H_0 : p \geq \theta$ is tested against the alternative hypothesis $H_1 : p < \theta$ and model simulations are evaluated until one of the hypotheses is accepted. In case ϕ is of the form $P_{\leq\theta}[\psi]$ the roles of the null and alternative hypotheses switch. Moreover in terms of hypothesis testing $P_{>\theta}[\psi]$ is equivalent to $P_{\geq\theta}[\psi]$, respectively $P_{<\theta}[\psi]$ is equivalent to $P_{\leq\theta}[\psi]$.

The approximation error of this method is determined by the strength $\langle\alpha, \beta\rangle$ of the acceptance sampling test where

- $\alpha = \mathrm{P}[H_1 \text{ is accepted} \mid H_0 \text{ is true}]$ (Probability of type I error);
- $\beta = \mathrm{P}[H_0 \text{ is accepted} \mid H_1 \text{ is true}]$ (Probability of type II error).

In case the probability θ specified in the logic property ϕ is close to the true probability p a large number of simulations is required to validate a hypothesis and it is impossible to ensure a low probability of type I and type II errors simultaneously; see [15] for details.

Therefore the hypothesis testing problem constraints are relaxed. An *indifference region* $(p - \delta, p + \delta)$ of width 2δ is introduced where neither of the two hypotheses is true. In this new setting three hypotheses are considered:

- The null hypothesis $H'_0 : p \geq \theta + \delta$;
- The alternative hypothesis: $H'_1 : p < \theta - \delta$;
- The undecided hypothesis: $H_2 : \theta - \delta \leq p < \theta + \delta$.

Using two acceptance sampling tests it is possible to decide if $\phi \equiv P_{\geq\theta}[\psi]$ holds:

Test 1 with strength $\langle\alpha, \gamma\rangle(H_0 : p \geq \theta, H'_1 : p < \theta - \delta)$
Test 2 with strength $\langle\gamma, \beta\rangle(H'_0 : p \geq \theta + \delta, H_1 : p < \theta - \delta)$

where γ represents the probability of *undecided* results. Whenever H_0 and H'_0 are accepted ϕ is declared to hold. Conversely if H_1 and H'_1 are accepted then ϕ is declared not to hold. Otherwise the validity of ϕ is undecided.

Two types of acceptance sampling plans can be employed to determine the true hypothesis:

- Single acceptance sampling plan;
- Sequential acceptance sampling plan.

Single sampling plan methods compute the values of the acceptance number c and the smallest number of required simulations n which ensure that the strength of the test $\langle \alpha, \beta \rangle$ is guaranteed. The number of simulations n is fixed in the beginning and the hypothesis H_0 is accepted if

$$\sum_{i=1}^{n} x_i > c$$

where x_i represents an observation of the $i-th$ Bernoulli variable (1 = true, 0 = false). Otherwise the hypothesis H_1 is true. Values for c and n can be obtained from a precomputed table of values [1] or approximated using binary search [11, p. 21].

The disadvantage of employing single sampling plans is that the number of required simulations is fixed and not updated while evaluating the simulations. This means that although sufficient evidence is available to validate one of the hypotheses the method will not stop early. For instance if the first $c < n$ simulations have been evaluated true H_0 is validated and further simulations are irrelevant to the final result. However since n is fixed the remaining $n - c$ simulations will be generated and evaluated as well.

Sequential acceptance sampling plans address this issue by verifying after each simulation evaluation if sufficient evidence is available to validate one of the hypotheses. An efficient sequential acceptance sampling plan is Wald's sequential probability ratio test [9].

After evaluating each simulation a value is computed

$$f_m = \prod_{i=1}^{m} \frac{P[X_i = xi \mid p = p_1]}{P[X_i = xi \mid p = p_0]} = \frac{p_1^d (1-p_1)^{m-d}}{p_0^d (1-p_0)^{m-d}}$$

where m is the number of simulations evaluated so far, $d = \sum_{i=1}^{m} x_i$ is the number of true evaluations, $p_0 = \theta + \delta$ and $p_1 = \theta - \delta$. The hypothesis H_0 is accepted if $f_m \leq B$, respectively hypothesis H_1 is accepted if $f_m \geq A$. Otherwise if $B \leq f_m \leq A$ insufficient evidence is available and additional simulations are required.

In practical applications an approximation of the optimal A and B values is used in order to reduce the overall complexity of the method [9, Section 3.4]:

$$A = \frac{1-\beta}{\alpha}; \quad B = \frac{\beta}{1-\alpha}.$$

The strength of the test $\langle \alpha', \beta' \rangle$ given by the approximated A and B values closely matches the initial strength $\langle \alpha, \beta \rangle$. In [9] it is shown that

$$\alpha' \leq \frac{\alpha}{1-\beta}; \qquad \beta' \leq \frac{\beta}{1-\alpha} \quad \alpha' + \beta' \leq \alpha + \beta$$

which means that at least one of the inequalities $\alpha' \leq \alpha$ and $\beta' \leq \beta$ must hold. Moreover if the values of α and β are small (e.g. less than 5%) then $\frac{\alpha}{1-\beta} \approx \alpha$ and $\frac{\beta}{1-\alpha} \approx \beta$ which means both inequalities hold.

The input parameters of the algorithm are α, β and δ. A detailed description of the statistical model checking algorithm is given in [15] and an example of a model checker implementing the algorithm is given in [12].

Statistical black-box model checking

Statistical black-box model checking initially introduced in [8] and further extended in [10] verifies if a logic property ϕ holds for a model $\mathcal{M}$ using statistical hypothesis testing based on p-values. In contrast to statistical model checking a fixed number of simulations is provided and the model cannot be simulated on demand.

Let us denote the sum of all Bernoulli variables by $Y = \sum_{i=1}^{n} X_i$. Then Y has a binomial distribution with the cumulative distribution function:

$$F(c; n, p) = \sum_{i=1}^{c} \binom{n}{i} p^i (1-p)^{n-i}.$$

If ϕ is a logic property of the form $P_{\geq \theta}[\psi]$ then the null hypothesis $H_0 : p \geq \theta$ is tested against the alternative hypothesis $H_1 : p < \theta$. A p-value is computed for each hypothesis and the hypothesis with the lowest p-value is accepted [10]:

$$p_{H_0} = 1 - F(d-1; n, \theta)$$
$$p_{H_1} = F(d; n, \theta)$$

where n is the number of Bernoulli variables, $d = \sum_{i=1}^{m} x_i$ is the number of true evaluations and θ is the probability specified within the logic property ϕ. In case the p-values are equal the alternative hypothesis H_1 is accepted.

More details and usage examples regarding the extended statistical black-box model checking method are given in [10].

Bayesian mean and variance estimate based model checking

Bayesian mean and variance estimate based model checking [7] verifies if a logic property ϕ holds for a model $\mathcal{M}$ by estimating the true probability p of ϕ being true. In contrast to the frequentist model checking approach the present approach uses prior information during the estimation process.

Simulation evaluations are represented as iid Bernoulli variables with the probability of the logic property ϕ being true equal to p. Therefore we can assume that the *posterior* has a Bernoulli distribution with parameter p. The conjugate *prior* of a Bernoulli distribution is a Beta distribution with shape parameters α and β. Thus the prior information considered during the estimation process is represented by a Beta distribution. If prior information is unavailable an unbiased prior can be used ($\alpha = 1$, $\beta = 1$). Both shape parameters are provided by the user as input to the algorithm.

Considering the user-defined Beta distribution shape parameters α and β the algorithm updates the estimate of the true probability ρ and variance ν after

evaluating ϕ for each newly generated sample. The formulae for computing the estimates ρ and ν are:

$$\rho = \frac{k+\alpha}{\alpha+\beta+n}$$
$$\nu = \frac{(\alpha+k)(n-k+\beta)}{(\alpha+n+\beta)^2(\alpha+n+\beta+1)}$$

where n represents the number of generated samples, k represents the number of samples for which ϕ was evaluated true, and $\alpha > 0$ and $\beta > 0$ are the Beta distribution shape parameters.

New samples are generated and the estimates ρ and ν are updated until the condition $\nu < T$ is true, where $T > 0$ is a user-defined threshold value provided as input to the algorithm. Considering that the logic property ϕ is of the form $P_{\geq\theta}[\psi]$ ($P_{\leq\theta}[\psi]$) ϕ will be evaluated true if $\rho \geq \theta$ ($\rho \leq \theta$).

A detailed description of the algorithm and usage examples are provided in [7].

Bayesian statistical model checking

Bayesian statistical model checking [5,6] verifies if a logic property ϕ holds for a model $\mathcal{M}$ using statistical hypothesis testing. In contrast to frequentist statistical model checking approaches the present approach employs prior information for validating one of the hypotheses.

Let us assume that ϕ is a logic property of the form $P_{\geq\theta}[\psi]$. The null hypothesis $H_0 : p \geq \theta$ is tested against the alternative hypothesis $H_1 : p < \theta$ and model simulations are evaluated until one of the hypotheses is accepted. In case ϕ is of the form $P_{\leq\theta}[\psi]$ the roles of the null and alternative hypotheses switch.

A measure of relative confidence in H_0 with respect to H_1 is defined called Bayes factor $\mathcal{B}$. The value of $\mathcal{B}$ considering a sequence of simulation evaluations $s = (x_1, x_2, ..., x_n)$ and hypotheses H_0 and H_1 is computed as follows:

$$\mathcal{B} = \frac{P(s \mid H_0)}{P(s \mid H_1)} = \frac{P(H_0 \mid s)P(H_1)}{P(H_1 \mid s)P(H_0)}.$$

Similarly to the Bayesian mean and variance estimation based model checking approach the posterior is assumed to have a Bernoulli distribution. Therefore the conjugate prior has a Beta distribution with shape parameters $\alpha > 0$ and $\beta > 0$. Both Beta distribution shape parameters are provided as input to the algorithm.

Considering these assumptions it is shown in [5] that the value of $\mathcal{B}$ can be computed with respect to the cumulative Beta distribution function:

$$\mathcal{B} = \frac{1}{F_{(x+\alpha,n-x+\beta)}(\theta)} - 1$$

where n represents the total number of simulation evaluations, x represents the number of simulations for which ϕ was evaluated true, and

$$F_{(\alpha',\beta')}(\theta) = I_\theta(\alpha', \beta')$$

is the cumulative Beta distribution function with shape parameters $\alpha' = x + \alpha$ and $\beta' = n - x + \beta$ such that $I_\theta(\alpha', \beta')$ is the regularized incomplete beta function.

The null hypothesis H_0 is accepted if $\mathcal{B} > T$ where T is a user-defined threshold value provided as input to the algorithm. Conversely the alternative hypothesis H_1 is accepted if $\mathcal{B} < 1/T$. Otherwise if $1/T \leq \mathcal{B} \leq T$ insufficient evidence is available and additional simulations need to be generated and evaluated. A threshold value $\mathcal{T} = 10^{-2}$ suggests to provide decisive evidence against H_0 and in favour of H_1 [4, Appendix B]. Conversely a threshold value of 10^2 suggests to provide decisive evidence in favour of H_0.

A detailed description of the algorithm and usage examples are provided in [5]. Moreover an example of applying Bayesian statistical model checking to Simulink/Stateflow is given in [16].

References

[1] Frank E. Grubbs. On designing single sampling inspection plans. *The Annals of Mathematical Statistics*, 20(2):242–256, June 1949.

[2] Thomas Hérault, Richard Lassaigne, Frédéric Magniette, and Sylvain Peyronnet. Approximate probabilistic model checking. In Bernhard Steffen and Giorgio Levi, editors, *Verification, Model Checking, and Abstract Interpretation*, number 2937 in Lecture Notes in Computer Science, pages 73–84. Springer Berlin Heidelberg, Venice, Italy, January 2004.

[3] Wassily Hoeffding. Probability inequalities for sums of bounded random variables. *Journal of the American Statistical Association*, 58(301):13–30, March 1963.

[4] Sir Harold Jeffreys. *The Theory of Probability*. Oxford University Press, 3 edition, 1961.

[5] Sumit Jha, Edmund Clarke, Christopher Langmead, Axel Legay, Andre Platzer, and Paolo Zuliani. Statistical model checking for complex stochastic models in systems biology. *Computer Science Department*, March 2009.

[6] Sumit K. Jha, Edmund M. Clarke, Christopher J. Langmead, Axel Legay, André Platzer, and Paolo Zuliani. A bayesian approach to model checking biological systems. In Pierpaolo Degano and Roberto Gorrieri, editors, *Computational Methods in Systems Biology*, number 5688 in Lecture Notes in Computer Science, pages 218–234. Springer Berlin Heidelberg, Bologna, Italy, January 2009.

[7] Christopher Langmead. Generalized queries and bayesian statistical model checking in dynamic bayesian networks: Application to personalized medicine. *Computer Science Department*, August 2009.

[8] Koushik Sen, Mahesh Viswanathan, and Gul Agha. Statistical model checking of black-box probabilistic systems. In Rajeev Alur and Doron A. Peled, editors, *Computer Aided Verification*, number 3114 in Lecture Notes in Computer Science, pages 202–215. Springer Berlin Heidelberg, Boston, MA, USA, January 2004.

[9] A. Wald. Sequential tests of statistical hypotheses. *The Annals of Mathematical Statistics*, 16(2):117–186, June 1945.

[10] Håkan L. S. Younes. Probabilistic verification for "Black-Box" systems. In Kousha Etessami and Sriram K. Rajamani, editors, *Computer Aided Verification*, number 3576 in Lecture Notes in Computer Science, pages 253–265. Springer Berlin Heidelberg, Edinburgh, Scotland, UK, January 2005.

[11] Håkan L. S. Younes. *Verification and Planning for Stochastic Processes with Asynchronous Events.* Doctor of philosophy, Carnegie Mellon, Pittsburgh, 2005.

[12] Håkan L. S. Younes. Ymer: A statistical model checker. In Kousha Etessami and Sriram K. Rajamani, editors, *Computer Aided Verification*, number 3576 in Lecture Notes in Computer Science, pages 429–433. Springer Berlin Heidelberg, January 2005.

[13] Håkan L. S. Younes, Marta Kwiatkowska, Gethin Norman, and David Parker. Numerical vs. statistical probabilistic model checking. *International Journal on Software Tools for Technology Transfer*, 8(3):216–228, June 2006.

[14] Håkan L. S. Younes and Reid G. Simmons. Probabilistic verification of discrete event systems using acceptance sampling. In Ed Brinksma and Kim Guldstrand Larsen, editors, *Computer Aided Verification*, number 2404 in Lecture Notes in Computer Science, pages 223–235. Springer Berlin Heidelberg, January 2002.

[15] Håkan L.S. Younes and Reid G. Simmons. Statistical probabilistic model checking with a focus on time-bounded properties. *Information and Computation*, 204(9):1368–1409, September 2006.

[16] Paolo Zuliani, André Platzer, and Edmund M. Clarke. Bayesian statistical model checking with application to Simulink/Stateflow verification. In *Proceedings of the 13th ACM international conference on Hybrid systems: computation and control*, HSCC '10, page 243–252, New York, NY, USA, 2010. ACM.
