# Supplementary material for: Automatic validation of computational models using pseudo-3D spatio-temporal model checking
Source: BMC Syst Biol. 2014 Dec 2;8:124. doi: 10.1186/s12918-014-0124-0 (PMC4272535; doi:10.1186/s12918-014-0124-0)
Supplement: Additional file 5 — Well-defined model checking problem. A proof that the model checking problem is well-defined. [file 12918_2014_124_MOESM5_ESM.pdf]

Well-defined model checking problem

In order to show that the model checking problem is *well-defined* we will first prove that the number of required simulations and state transitions within each simulation are finite.

Finite number of required simulations

Probabilistic black-box model checking is the only approach considered which can provide an answer regardless of the number of available model simulations. Conversely all other considered methods require a minimum number of model simulations, which can be computed at the beginning (Chernoff-Hoeffding bounds) or not (Frequentist and Bayesian statistical, Bayesian mean and variance estimate), to provide an answer considering a given confidence level. Although all model checking methods require a finite number of model simulations the expected time required for an answer to be provided varies with the value of the true probability p and the user-defined probability θ. However for practical applications users might want to set an upper bound on the time to wait until an answer is provided. Thus we employ the wrapper Algorithm 1 to execute each specific model checking algorithm in Table 1. If an answer can be provided using the requested approach within the specified extra evaluation time interval then it is reported to the user. Otherwise probabilistic black-box model checking is employed to report the answer based on the model simulations generated and evaluated so far.

Table 1: Considered approximate probabilistic model checking approaches. Bayesian methods consider prior knowledge about the parameters and variables in the model when deciding if a logic property holds. Conversely frequentist approaches assume no prior knowledge is available. All methods except probabilistic black-box take as input a user-defined upper bound on the approximation error. They request additional model executions until the result is sufficiently accurate. Probabilistic black-box model checking takes a fixed number of model simulations as input and computes a p-value as the confidence measure of the result.

	**Frequentist**	**Bayesian**
**Estimate**	Chernoff-Hoeffding bounds [1]	Mean and variance [3]
**Hypothesis testing**	Statistical [7]<br>Probabilistic black-box [5, 6]	Statistical [2]

In the initialisation step of Algorithm 1 `nrOfTimeoutSeconds`, the number of seconds to wait between re-executing the extra evaluation program, is fixed. The reason for introducing such a variable is to temporarily wait and allow the model simulator to finish its execution before verifying if new simulations were provided. Afterwards the collection of valid model `simulations` is initialised based on the given `simulationsInputSet`. The model checker of type `modelCheckingType` is then executed to verify if the logic property `logicProperty` holds considering the available `simulations` and set

Algorithm 1 The wrapper algorithm employed to call specific model checking algorithms (see Table 1 for the considered approaches). If sufficient model simulations are available, respectively generated and evaluated within *extraEvaluationTime* minutes, then the chosen specific model checking algorithm is used to provide an answer. Otherwise the user is informed that the maximum extra evaluation time threshold was reached and the answer is provided using the probabilistic black-box model checking approach. Model simulations are generated and stored in an input set *simulationsInputSet* using the external model simulation program *extraEvaluationProgram*. The logic property to be verified is stored in the variable *logicProperty*.

Require: *modelCheckingType* is the specific model checking approach, *modelCheckingParameters* is the collection of parameters required by the chosen *modelCheckingType*, *extraEvaluationTime* is the maximum number of minutes allowed for generating and evaluating additional model simulations, *extraEvaluationProgram* is the model simulation program which is called whenever new simulations are required, *simulationsInputSet* is the set containing the simulations and *logicProperty* is the PBLSTL logic property to be verified

Ensure: A true/false answer together with a measure of confidence is provided

```
nrOfTimeoutSeconds ← 30;          ▷ The default number of seconds to wait
                                     between re-executing the extra
                                     evaluation program and evaluating
                                     the generated traces

simulations ← GetSimulations(simulationsInputSet);

RunModelChecker(modelCheckingType, modelCheckingParameters,
                simulations, logicProperty, result, confidence);

while (elapsed number of minutes < extraEvaluationTime) AND
      (more model simulations are required) do
    GenerateModelSimulations(extraEvaluationProgram);
    Wait(nrOfTimeoutSeconds);
    UpdateCollectionOfSimulations(simulations, simulationsInputSet);
    RunModelChecker(modelCheckingType, modelCheckingParameters,
                    simulations, logicProperty, result, confidence);
end while

if more model simulations are required then
    RunProbBlackBoxModelChecker(simulations, logicProperty,
                                result, confidence);
end if

Output result and confidence;
```

of `modelCheckingParameters`. While the number of elapsed minutes is less than `extraEvaluationTime` and the number of available model simulations

is insufficient to evaluate `logicProperty` the loop comprising the following steps is executed:

1. Run `extraEvaluationProgram` to generate new simulations.
2. Wait for `nrOfTimeoutSeconds` to give the extra evaluation program enough time to output results.
3. The collection of `simulations` is updated considering valid and previously unevaluated simulation input files.
4. The `modelCheckingType` model checker execution is resumed considering the additional `simulations`.

The loop is exited when either `extraEvaluationTime` minutes elapsed or enough model simulations have been provided. In the former case the probabilistic black-box model checker is executed to provide a `result`. Otherwise the `result` is computed using the `modelCheckingType` model checker. In the end both `result` and `confidence` measure are reported to the user.

The main advantages of Algorithm 1 are:

- The model checking execution time and number of generated and evaluated simulations is finite. Depending on the parameters of the model checker, the distribution of the data and the number of required simulations the answer will be provided using the desired model checker type or the default probabilistic black-box model checker.
- In contrast to traditional model checking methods in our approach the model checking task is decoupled from a specific model and model simulation environment (e.g. Matlab [4]). An external program which can generate simulations is provided as input to the model checker. Whenever additional model simulations are required this external program is executed. For the algorithm implementation our recommendation is that the employed external program should be a script (e.g. Bash [UNIX], Batch [Windows]) which calls the model simulator and stores the output into the specified location.

Finite number of state transitions

Logic properties are evaluated with respect to simulations of computational models. In order to be able to decide if the logic property is satisfied, the model simulation must cover a sufficiently long time frame. Stopping the simulation early could potentially render the evaluation of temporal logic properties undecidable. Therefore there is a need for a mechanism to decide when a simulation execution can be stopped.

When verifying BLSTL logic properties an upper bound can be placed on the required simulation time because all temporal logic operators are bounded. Let us denote the upper bound corresponding to a BLSTL logic property ψ by $\lceil \psi \rceil$.

Definition. *The* upper bound $\lceil \psi \rceil \in \mathbb{N}$ *corresponding to a BLSTL logic property* ψ *considering an execution* σ *is defined recursively on the structure of the logic property as follows:*

- $\lceil nsm \asymp nm \rceil = 0$ *because the value of* nsm *and* nm *is computed considering only* $\sigma[0]$*;*
- $\lceil nsv \asymp nm \rceil = 0$ *because the value of* nsv *and* nm *is computed considering only* $\sigma[0]$*;*
- $\lceil d(nm1) \asymp nm2 \rceil = 1$ *because the value of* $nm1$ *is computed considering both* $\sigma[0]$ *and* $\sigma[1]$*;*
- $\lceil \neg\psi \rceil = \lceil \psi \rceil$*;*
- $\lceil \psi_1 \wedge \psi_2 \rceil = \max(\lceil \psi_1 \rceil, \lceil \psi_2 \rceil)$*;*
- $\lceil \psi_1 \vee \psi_2 \rceil = \max(\lceil \psi_1 \rceil, \lceil \psi_2 \rceil)$*;*
- $\lceil \psi_1 \Rightarrow \psi_2 \rceil = \max(\lceil \psi_1 \rceil, \lceil \psi_2 \rceil)$*;*
- $\lceil \psi_1 \Leftrightarrow \psi_2 \rceil = \max(\lceil \psi_1 \rceil, \lceil \psi_2 \rceil)$*;*
- $\lceil \psi_1 \ U[a,b]\ \psi_2 \rceil = \max(b - 1 + \lceil \psi_1 \rceil, b + \lceil \psi_2 \rceil) \leq b + \max(\lceil \psi_1 \rceil, \lceil \psi_2 \rceil)$*;*
- $\lceil F[a,b]\ \psi \rceil = b + \lceil \psi \rceil$*;*
- $\lceil G[a,b]\ \psi \rceil = b + \lceil \psi \rceil$*;*
- $\lceil X\psi \rceil = 1 + \lceil \psi \rceil$*;*
- $\lceil X[k]\ \psi \rceil = k + \lceil \psi \rceil$*;*
- $\lceil (\psi) \rceil = \lceil \psi \rceil$*;*

Thus the minimum simulation time frame to be covered by model executions when verifying a BLSTL logic property ψ is $[0, \lceil \psi \rceil]$.

Lemma 1. *Let us assume that a BLSTL logic property* ψ *is verified against an infinite execution* $\sigma = \{(s_0, t_0), (s_1, t_1), (s_2, t_2), ...\}$*. Moreover let us denote a finite prefix of* σ *by* $\hat{\sigma} = \{(\hat{s_0}, \hat{t_0}), (\hat{s_1}, \hat{t_1}), ..., (\hat{s_m}, \hat{t_m})\}$ *where*

$$\hat{s_i} = s_i \text{ and } \hat{t_i} = t_i, \forall i = \overline{0, m} \text{ with } \sum_{i=0}^{m} t_i \geq \lceil \psi \rceil \text{ and } \sum_{i=0}^{m-1} t_i < \lceil \psi \rceil.$$

Then $\sigma \models \psi$ **if and only if** $\hat{\sigma} \models \psi$*.*

Proof. We will prove the results of Lemma 1 recursively on the structure of the logic property ψ as described below:

1. $\sigma \models nsm \asymp nm$ **if and only if** $\hat{\sigma} \models nsm \asymp nm$

   Proof.

   (a) $\sigma \models nsm \asymp nm$ **if and only if** $nsm \asymp nm$.

   (b) $\hat{\sigma} \models nsm \asymp nm$ **if and only if** $nsm \asymp nm$.

   (c) By Definition 9 $\lceil \psi \rceil = 0$ which means that according to the assumptions of Lemma 1 $\hat{s}_0 = s_0$. Hence the symbols nsm and nm are evaluated to the same values for both σ and $\hat{\sigma}$.

(d) From 1a, 1b and 1c it follows that $\sigma \models nsm \asymp nm$ **if and only if** $\hat{\sigma} \models nsm \asymp nm$.

□

2. $\sigma \models nsv \asymp nm$ **if and only if** $\hat{\sigma} \models nsv \asymp nm$ (Proof is similar to the one provided for 1).

3. $\sigma \models d(nm1) \asymp nm2$ **if and only if** $\hat{\sigma} \models d(nm1) \asymp nm2$

Proof.

(a) $\sigma \models d(nm1) \asymp nm2$ **if and only if** $|\sigma| > 1$ and $(nm1^1$ - $nm1^0) \asymp nm2$.

(b) $\hat{\sigma} \models d(nm1) \asymp nm2$ **if and only if** $|\hat{\sigma}| > 1$ and $(nm1^1$ - $nm1^0) \asymp nm2$.

(c) By Definition 9 $\lceil\psi\rceil = 1$ which means that according to the assumptions of Lemma 1 $\hat{s}_0 = s_0$ and $\hat{s}_1 = s_1$. Hence the symbols $nm1^0, nm1^1$ and $nm2$ are evaluated to the same values for both σ and $\hat{\sigma}$.

(d) From 3a, 3b and 3c it follows that $\sigma \models d(nm1) \asymp nm2$ **if and only if** $\hat{\sigma} \models d(nm1) \asymp nm2$.

□

4. $\sigma \models \neg\psi$ **if and only if** $\hat{\sigma} \models \neg\psi$

Proof.

(a) $\sigma \models \neg\psi$ **if and only if** $\sigma \not\models \psi$.

(b) $\hat{\sigma} \models \neg\psi$ **if and only if** $\hat{\sigma} \not\models \psi$.

(c) By Definition 9 $\lceil\neg\psi\rceil = \lceil\psi\rceil$ which means that according to the assumptions of Lemma 1 $\hat{s}_0 = s_0$, $\hat{s}_1 = s_1$, ..., $\hat{s}_m = s_m$ where the value of m is determined such that sufficient timepoints are recorded for the evaluation of ψ. Hence the semantics of ψ considering σ is equivalent to the semantics of ψ considering $\hat{\sigma}$.

(d) From 4c it follows that $\sigma \not\models \psi$ **if and only if** $\hat{\sigma} \not\models \psi$.

(e) From 4a and 4d it follows that $\sigma \models \neg\psi$ **if and only if** $\hat{\sigma} \not\models \psi$.

(f) From 4b and 4e it follows that $\sigma \models \neg\psi$ **if and only if** $\hat{\sigma} \models \neg\psi$.

□

5. $\sigma \models \psi_1 \wedge \psi_2$ **if and only if** $\hat{\sigma} \models \psi_1 \wedge \psi_2$

Proof.

(a) $\sigma \models \psi_1 \wedge \psi_2$ **if and only if** $\sigma \models \psi_1$ and $\sigma \models \psi_2$.

(b) $\hat{\sigma} \models \psi_1 \wedge \psi_2$ **if and only if** $\hat{\sigma} \models \psi_1$ and $\hat{\sigma} \models \psi_2$.

(c) By Definition 9 $\lceil \psi_1 \wedge \psi_2 \rceil = \max(\lceil \psi_1 \rceil, \lceil \psi_2 \rceil)$ which means that according to the assumptions of Lemma 1 $\hat{s}_0 = s_0$, $\hat{s}_1 = s_1$, ..., $\hat{s}_m = s_m$ where the value of m is determined such that sufficient timepoints are recorded for the evaluation of both ψ_1 and ψ_2. Hence the semantics of ψ_1 and ψ_2 is the same considering both σ and $\hat{\sigma}$.

(d) From 5c it follows that $\sigma \models \psi_1$ **if and only if** $\hat{\sigma} \models \psi_1$, respectively $\sigma \models \psi_2$ **if and only if** $\hat{\sigma} \models \psi_2$.

(e) From 5d it follows that $\sigma \models \psi_1$ and $\sigma \models \psi_2$ **if and only if** $\hat{\sigma} \models \psi_1$ and $\hat{\sigma} \models \psi_2$.

(f) From 5a and 5e it follows that $\sigma \models \psi_1 \wedge \psi_2$ **if and only if** $\hat{\sigma} \models \psi_1$ and $\hat{\sigma} \models \psi_2$.

(g) From 5b and 5f it follows that $\sigma \models \psi_1 \wedge \psi_2$ **if and only if** $\hat{\sigma} \models \psi_1 \wedge \psi_2$.

□

6. $\sigma \models \psi_1 \vee \psi_2$ **if and only if** $\hat{\sigma} \models \psi_1 \vee \psi_2$ (Proof is similar to the one provided for 5).

7. $\sigma \models \psi_1 \Rightarrow \psi_2$ **if and only if** $\hat{\sigma} \models \psi_1 \Rightarrow \psi_2$ (Proof is similar to the one provided for 5).

8. $\sigma \models \psi_1 \Leftrightarrow \psi_2$ **if and only if** $\hat{\sigma} \models \psi_1 \Leftrightarrow \psi_2$ (Proof is similar to the one provided for 5).

9. $\sigma \models \psi_1\ U[a,b]\ \psi_2$ **if and only if** $\hat{\sigma} \models \psi_1\ U[a,b]\ \psi_2$

Proof.

(a) $\sigma \models \psi_1\ U[a,b]\ \psi_2$ **if and only if** $\exists i$, $a \leq i \leq b$ such that $\sigma^i \models \psi_2$, and for all j, $a \leq j < i$, $\sigma^j \models \psi_1$;

(b) $\hat{\sigma} \models \psi_1\ U[a,b]\ \psi_2$ **if and only if** $\exists i'$, $a \leq i' \leq b$ such that $\hat{\sigma}^{i'} \models \psi_2$, and for all j', $a \leq j' < i'$, $\hat{\sigma}^{j'} \models \psi_1$;

(c) By Definition 9 $\lceil \psi_1\ U[a,b]\ \psi_2 \rceil = b + \max(\lceil \psi_1 \rceil, \lceil \psi_2 \rceil)$. This means that according to the assumptions of Lemma 1 $\hat{s}_0 = s_0$, $\hat{s}_1 = s_1$, ..., $\hat{s}_m = s_m$ where the value of m is determined such that sufficient time points are recorded for the evaluation of both ψ_1 and ψ_2 considering any execution suffix $\sigma^h/\hat{\sigma}^h$, $a \leq h \leq b$.

(d) From 9c it follows that for any suffix execution $\sigma^h/\hat{\sigma}^h$, $a \leq h \leq b$ the semantics of ψ_1 and ψ_2 is the same.

(e) From 9d it follows that $\exists i$, $a \leq i \leq b$ such that $\sigma^i \models \psi_2$ **if and only if** $\exists i'$, $a \leq i' \leq b$, $i' = i$ such that $\hat{\sigma}^{i'} \models \psi_2$.

(f) From 9d it follows that $\forall j$, $a \leq j < i \leq b$ such that $\sigma^j \models \psi_1$ **if and only if** $\forall j'$, $a \leq j' < i' \leq b$, $i' = i$, $j' = j$ such that $\hat{\sigma}^{j'} \models \psi_1$.

(g) From 9e and 9f it follows that $\exists i$, $a \leq i \leq b$ such that $\sigma^i \models \psi_2$ and $\forall j$, $a \leq j < i \leq b$ such that $\sigma^j \models \psi_1$ **if and only if** $\exists i'$, $a \leq i' \leq b$, $i' = i$ such that $\hat{\sigma}^{i'} \models \psi_2$ and $\forall j'$, $a \leq j' < i' \leq b$, $j' = j$ such that $\hat{\sigma}^{j'} \models \psi_1$.

(h) From 9a and 9g it follows that $\sigma \models \psi_1 \; U[a,b] \; \psi_2$ **if and only if** $\exists i'$, $a \leq i' \leq b$, such that $\hat{\sigma}^{i'} \models \psi_2$ and $\forall j'$, $a \leq j' < i' \leq b$, $j' = j$ such that $\hat{\sigma}^{j'} \models \psi_1$.

(i) From 9b and 9h it follows that $\sigma \models \psi_1 \; U[a,b] \; \psi_2$ **if and only if** $\hat{\sigma} \models \psi_1 \; U[a,b] \; \psi_2$.

□

10. $\sigma \models F[a,b] \; \psi$ **if and only if** $\hat{\sigma} \models F[a,b] \; \psi$

Proof.

(a) $\sigma \models F[a,b] \; \psi$ **if and only if** $\exists i$, $a \leq i \leq b$ such that $\sigma^i \models \psi$;

(b) $\hat{\sigma} \models F[a,b] \; \psi$ **if and only if** $\exists i'$, $a \leq i' \leq b$ such that $\hat{\sigma}^{i'} \models \psi$;

(c) By Definition 9 $\lceil F[a,b] \; \psi \rceil = b + \lceil \psi \rceil$. This means that according to the assumptions of Lemma 1 $\hat{s}_0 = s_0$, $\hat{s}_1 = s_1$, ..., $\hat{s}_m = s_m$ where the value of m is determined such that sufficient time points are recorded for the evaluation of ψ considering any execution suffix $\sigma^h/\hat{\sigma}^h$, $a \leq h \leq b$.

(d) From 10c it follows that the semantics of ψ is equivalent for suffix executions σ^h and $\hat{\sigma}^h$, $\forall h, a \leq h \leq b$.

(e) From 10d it follows that $\exists i$, $a \leq i \leq b$ such that $\sigma^i \models \psi$ **if and only if** $\exists i'$, $a \leq i' \leq b$, $i' = i$ such that $\hat{\sigma}^{i'} \models \psi$.

(f) From 10a and 10e it follows that $\sigma \models F[a,b] \; \psi$ **if and only if** $\exists i'$, $a \leq i' \leq b$ such that $\hat{\sigma}^{i'} \models \psi$.

(g) From 10b and 10f it follows that $\sigma \models F[a,b] \; \psi$ **if and only if** $\hat{\sigma} \models F[a,b] \; \psi$.

□

11. $\sigma \models G[a,b] \; \psi$ **if and only if** $\hat{\sigma} \models G[a,b] \; \psi$

Proof.

(a) $\sigma \models G[a,b] \; \psi$ **if and only if** $\forall i$, $a \leq i \leq b$, $\sigma^i \models \psi$;

(b) $\hat{\sigma} \models G[a,b] \; \psi$ **if and only if** $\forall i'$, $a \leq i' \leq b$, $\hat{\sigma}^{i'} \models \psi$;

(c) By Definition 9 $\lceil G[a,b] \; \psi \rceil = b + \lceil \psi \rceil$. This means that according to the assumptions of Lemma 1 $\hat{s}_0 = s_0$, $\hat{s}_1 = s_1$, ..., $\hat{s}_m = s_m$ where the value of m is determined such that sufficient time points are recorded for the evaluation of ψ considering any execution suffix $\sigma^h/\hat{\sigma}^h$, $a \leq h \leq b$.

(d) From 11c it follows that the semantics of ψ is equivalent for suffix executions σ^h and $\hat{\sigma}^h$, $\forall h, a \leq h \leq b$.

(e) From 11d it follows that $\forall i$, $a \leq i \leq b$, $\sigma^i \models \psi$ **if and only if** $\forall i'$, $a \leq i' \leq b$, $i' = i$, $\hat{\sigma}^{i'} \models \psi$.

(f) From 11a and 11e it follows that $\sigma \models G[a, b]\ \psi$ **if and only if** $\forall i'$, $a \leq i' \leq b$, $\hat{\sigma}^{i'} \models \psi$.

(g) From 11b and 11f it follows that $\sigma \models G[a, b]\ \psi$ **if and only if** $\hat{\sigma} \models G[a, b]\ \psi$.

□

12. $\sigma \models X\ \psi$ **if and only if** $\hat{\sigma} \models X\psi$

Proof.

(a) $\sigma \models X\ \psi$ **if and only if** $|\sigma| > 1$ and $\sigma^1 \models \psi$;

(b) $\hat{\sigma} \models X\ \psi$ **if and only if** $|\hat{\sigma}| > 1$ and $\hat{\sigma}^1 \models \psi$;

(c) By Definition 9 $\lceil X\ \psi \rceil = 1 + \lceil \psi \rceil$. This means that according to the assumptions of Lemma 1 $\hat{s}_0 = s_0$, $\hat{s}_1 = s_1$, ..., $\hat{s}_m = s_m$ where the value of m is determined such that sufficient time points are recorded for the evaluation of ψ considering the execution suffix $\sigma^1/\hat{\sigma}^1$.

(d) From 12c it follows that the semantics of ψ is equivalent for suffix executions σ^1 and $\hat{\sigma}^1$.

(e) From 12d it follows that $\sigma^1 \models \psi$ **if and only if** $\hat{\sigma}^1 \models \psi$.

(f) From 12a and 12e it follows that $\sigma \models X\ \psi$ **if and only if** $\hat{\sigma}^1 \models \psi$.

(g) From 12b and 12f it follows that $\sigma \models X\ \psi$ **if and only if** $\hat{\sigma} \models X\ \psi$.

□

13. $\sigma \models X[k]\ \psi$ **if and only if** $\hat{\sigma} \models X[k]\ \psi$

Proof.

(a) $\sigma \models X[k]\ \psi$ **if and only if** $|\sigma| > k$ and $\sigma^k \models \psi$;

(b) $\hat{\sigma} \models X[k]\ \psi$ **if and only if** $|\hat{\sigma}| > k$ and $\hat{\sigma}^k \models \psi$;

(c) By Definition 9 $\lceil X[k]\ \psi \rceil = k + \lceil \psi \rceil$. This means that according to the assumptions of Lemma 1 $\hat{s}_0 = s_0$, $\hat{s}_1 = s_1$, ..., $\hat{s}_m = s_m$ where the value of m is determined such that sufficient time points are recorded for the evaluation of ψ considering the execution suffix $\sigma^k/\hat{\sigma}^k$.

(d) From 13c it follows that the semantics of ψ is equivalent for suffix executions σ^k and $\hat{\sigma}^k$.

(e) From 13d it follows that $\sigma^k \models \psi$ **if and only if** $\hat{\sigma}^k \models \psi$.

(f) From 13a and 13e it follows that $\sigma \models X[k]\ \psi$ **if and only if** $\hat{\sigma}^k \models \psi$.

(g) From 13b and 13f it follows that $\sigma \models X[k]\ \psi$ **if and only if** $\hat{\sigma} \models X[k]\ \psi$.

□

14. $\sigma \models (\psi)$ **if and only if** $\hat{\sigma} \models (\psi)$

Proof.

(a) $\sigma \models (\psi)$ **if and only if** $\sigma \models \psi$;

(b) $\hat{\sigma} \models (\psi)$ **if and only if** $\hat{\sigma} \models \psi$;

(c) By Definition 9 $\lceil(\psi)\rceil = \lceil\psi\rceil$. This means that according to the assumptions of Lemma 1 $\hat{s}_0 = s_0$, $\hat{s}_1 = s_1$, ..., $\hat{s}_m = s_m$ where the value of m is determined such that sufficient time points are recorded for the evaluation of ψ.

(d) From 14c it follows that the semantics of ψ is equivalent for both σ and $\hat{\sigma}$.

(e) From 14d it follows that $\sigma \models \psi$ **if and only if** $\hat{\sigma} \models \psi$.

(f) From 14a and 14e it follows that $\sigma \models (\psi)$ **if and only if** $\hat{\sigma} \models \psi$.

(g) From 14b and 14f it follows that $\sigma \models (\psi)$ **if and only if** $\hat{\sigma} \models (\psi)$.

□

□

Lemma 2. *The number of state transitions required to verify a BLSTL logic property is* finite*.*

Proof. From Lemma 1 it follows that a BLSTL logic property ψ can be verified against a model simulation σ based on a finite prefix $\hat{\sigma}$. The minimum time interval captured by $\hat{\sigma}$ is bounded and can be computed using Definition . Since we assume the time divergence property holds for all the considered systems only a finite number of state transitions can occur in a bounded interval of time. □

Well-defined model checking problem

Theorem 1. *The spatio-temporal model checking problem is* well-defined*.*

Proof. It was shown that the number of required model executions in order to verify if a PBLSTL logic property ϕ holds is finite. Moreover considering Lemmas 1 and 2 only a finite prefix and a finite number of state transitions has to be considered for each model execution. Thus the evaluation of ϕ is reduced to the problem of evaluating non-temporal properties over a finite number of states for each model execution. This implies evaluating arithmetic expressions and/or detecting spatial entities which are both decidable. Hence the model checking problem is well-defined. □

References

[1] Thomas Hérault, Richard Lassaigne, Frédéric Magniette, and Sylvain Peyronnet. Approximate probabilistic model checking. In Bernhard Steffen and Giorgio Levi, editors, *Verification, Model Checking, and Abstract Interpretation*, number 2937 in Lecture Notes in Computer Science, pages 73–84. Springer Berlin Heidelberg, Venice, Italy, January 2004.

[2] Sumit K. Jha, Edmund M. Clarke, Christopher J. Langmead, Axel Legay, André Platzer, and Paolo Zuliani. A bayesian approach to model checking biological systems. In Pierpaolo Degano and Roberto Gorrieri, editors, *Computational Methods in Systems Biology*, number 5688 in Lecture Notes in Computer Science, pages 218–234. Springer Berlin Heidelberg, Bologna, Italy, January 2009.

[3] Christopher Langmead. Generalized queries and bayesian statistical model checking in dynamic bayesian networks: Application to personalized medicine. *Computer Science Department*, August 2009.

[4] Sucheendra K. Palaniappan, Benjamin M. Gyori, Bing Liu, David Hsu, and P. S. Thiagarajan. Statistical model checking based calibration and analysis of bio-pathway models. In Ashutosh Gupta and Thomas A. Henzinger, editors, *Computational Methods in Systems Biology*, number 8130 in Lecture Notes in Computer Science, pages 120–134. Springer Berlin Heidelberg, Klosterneuburg, Austria, January 2013.

[5] Koushik Sen, Mahesh Viswanathan, and Gul Agha. Statistical model checking of black-box probabilistic systems. In Rajeev Alur and Doron A. Peled, editors, *Computer Aided Verification*, number 3114 in Lecture Notes in Computer Science, pages 202–215. Springer Berlin Heidelberg, Boston, MA, USA, January 2004.

[6] Håkan L. S. Younes. Probabilistic verification for "Black-Box" systems. In Kousha Etessami and Sriram K. Rajamani, editors, *Computer Aided Verification*, number 3576 in Lecture Notes in Computer Science, pages 253–265. Springer Berlin Heidelberg, Edinburgh, Scotland, UK, January 2005.

[7] Håkan L.S. Younes and Reid G. Simmons. Statistical probabilistic model checking with a focus on time-bounded properties. *Information and Computation*, 204(9):1368–1409, September 2006.
