# Supplementary material for: Automatic validation of computational models using pseudo-3D spatio-temporal model checking
Source: BMC Syst Biol. 2014 Dec 2;8:124. doi: 10.1186/s12918-014-0124-0 (PMC4272535; doi:10.1186/s12918-014-0124-0)
Supplement: Additional file 7 — Model checking results for the phase variation case study. The results obtained for each model checker execution and PBLSTL statement considering the phase variation case study. For statistical analysis of the results the pdf file can be converted to a Microsoft Word document, using the online converter (http://www.pdfonline.com/pdf-to-word-converter), from which the table of results can be directly copied in a spreadsheet-like software such as Microsoft Excel (or LibreOffice Calc). In case you would like to use statistical software such as R you can save the results from Excel/Calc in csv format and then import them into R. [file 12918_2014_124_MOESM7_ESM.pdf]

Model checking results for the phase variation case study

For the convenience of the reader the set of PBLSTL statements considered for the phase variation case study will be restated below:

$$P >= 0.95\ [G\ [0,100]\ (d(count(regions)) >= 0)]. \tag{1}$$

$$\begin{aligned} P < 0.05\ [F\ [0,100]\ ((count(regions) > 0) \wedge \\ (min(regions, clusteredness) < 0.9))]. \end{aligned} \tag{2}$$

$$\begin{aligned} P > 0.95\ [(F\ [0,100]\ (G\ [0,100]\ ((count(regions) > 0) \wedge \\ (avg(regions, density) > 0.5)))) \vee \\ (G\ [0,100]\ (count(regions) = 0))]. \end{aligned} \tag{3}$$

$$\begin{aligned} P > 0.5\ [(F\ [0,100]\ ((count(regions) > 0) \wedge \\ (d(avg(regions, area)) > 0) \wedge \\ F\ [0,100]\ ((d(avg(regions, area)) < 0)))) \vee \\ (F\ [0,100]\ ((d(avg(regions, area)) < 0) \wedge \\ F\ [0,100]\ ((count(regions) > 0) \wedge \\ (d(avg(regions, area)) > 0)))) \vee \\ (G\ [0,100]\ (count(regions) = 0))] \end{aligned} \tag{4}$$

$$
\begin{aligned}
P > 0.6\ [(&F\ [0,100]\ ((count(regions) > 0) \wedge \\
&(d(avg(regions, perimeter)) > 0) \wedge \\
&F\ [0,100]\ ((d(avg(regions, perimeter)) < 0) \wedge \\
&F\ [0,100]\ ((count(regions) > 0) \wedge \\
&(d(avg(regions, perimeter)) > 0) \wedge \\
&F\ [0,100]\ ((d(avg(regions, perimeter)) < 0) \wedge \\
&F\ [0,100]\ ((count(regions) > 0) \wedge \\
&(d(avg(regions, perimeter)) > 0) \wedge \\
&F\ [0,100]\ ((d(avg(regions, perimeter)) < 0)))))))) \vee \\
&(F\ [0,100]\ ((d(avg(regions, perimeter)) < 0) \wedge \\
&F\ [0,100]\ ((count(regions) > 0) \wedge \\
&(d(avg(regions, perimeter)) > 0) \wedge \\
&F\ [0,100]\ ((d(avg(regions, perimeter)) < 0) \wedge \\
&F\ [0,100]\ ((count(regions) > 0) \wedge \\
&(d(avg(regions, perimeter)) > 0) \wedge \\
&F\ [0,100]\ ((d(avg(regions, perimeter)) < 0) \wedge \\
&F\ [0,100]\ ((count(regions) > 0) \wedge \\
&(d(avg(regions, perimeter)) > 0)))))))) \vee \\
&(G\ [0,100]\ (count(regions) = 0))].
\end{aligned} \tag{5}
$$

$$
\begin{aligned}
P < 0.1\ [&F\ [0,100]\ ((count(regions) > 0) \wedge \\
&(max(regions, angle) > 120))].
\end{aligned} \tag{6}
$$

$$
\begin{aligned}
P\ \ >=\ \ 0.95\ [(&F\ [0,100]\ (G\ [0,100]\ ((count(regions) > 0) \wedge \\
&(min(regions, distanceFromOrigin) > 100)))) \vee \\
&(G\ [0,100]\ (count(regions) = 0))].
\end{aligned} \tag{7}
$$

$$
\begin{aligned}
P > 0.8\ [(&F\ [0,100]\ (G\ [0,100]\ ((count(regions) > 0) \wedge \\
&(min(regions, triangleMeasure)\ \ > \\
&max(regions, rectangleMeasure)) \wedge \\
&(min(regions, triangleMeasure)\ \ > \\
&max(regions, circleMeasure))))) \vee \\
&(G\ [0,100]\ (count(regions) = 0))].
\end{aligned} \tag{8}
$$

Each table describes the results corresponding to one of the PBLSTL statements. The first column of each row represents the identifier of the model checking execution. The second column represents the evaluation result (T = true, F

= false) of the PBLSTL statement for that particular model checker execution. The number of STML files against which the PBLSTL statement was executed, respectively how many of them evaluated true and how many evaluated false is provided in columns three, four and five. Finally column six presents the execution time (minutes:seconds format) corresponding to each model checker run. All executions of the model checker employed the frequentist statistical model checking approach with both probability of type I and type II errors equal to 5%. No extra evaluation program was provided and the extra evaluation time was set to zero. Therefore in case the available number of simulations did not suffice the answer was provided using the probabilistic black-box model checking approach.

Table 1: Model checking results corresponding to logic property 1

**Id**	**Result**	**#total**	**#true**	**#false**	**Execution time (min:sec)**
1	T	58	58	0	0:02.42
2	T	69	68	1	0:02.94
3	T	69	68	1	0:02.89
4	T	69	68	1	0:02.85
5	T	69	68	1	0:02.87
6	T	69	68	1	0:02.89
7	T	58	58	0	0:02.46
8	T	69	68	1	0:02.89
9	T	83	81	2	0:03.49
10	T	69	68	1	0:02.84
11	T	58	58	0	0:02.45
12	T	83	81	2	0:03.47
13	T	69	68	1	0:02.85
14	T	69	68	1	0:02.90
15	T	58	58	0	0:03.12
16	T	96	93	3	0:04.26
17	T	96	93	3	0:04.52
18	T	58	58	0	0:02.36
19	T	58	58	0	0:02.43
20	T	58	58	0	0:02.40
21	T	58	58	0	0:02.44
22	T	69	68	1	0:02.92
23	T	58	58	0	0:02.38
24	T	69	68	1	0:02.85
25	T	58	58	0	0:02.42
26	T	58	58	0	0:02.42
27	T	83	81	2	0:03.40
28	T	69	68	1	0:02.86
29	T	69	68	1	0:02.85
30	T	83	81	2	0:03.49
31	T	69	68	1	0:02.89
32	T	69	68	1	0:02.93
33	T	58	58	0	0:02.41
34	T	69	68	1	0:02.90

Id	Result	#total	#true	#false	Execution time (min:sec)
35	T	69	68	1	0:02.91
36	T	83	81	2	0:04.11
37	T	83	81	2	0:03.50
38	T	110	106	4	0:04.53
39	T	58	58	0	0:02.41
40	T	69	68	1	0:02.88
41	T	83	81	2	0:03.45
42	T	58	58	0	0:02.47
43	T	58	58	0	0:02.44
44	T	58	58	0	0:02.47
45	T	58	58	0	0:02.44
46	T	58	58	0	0:02.46
47	T	69	68	1	0:02.89
48	T	58	58	0	0:02.41
49	T	58	58	0	0:02.42
50	T	58	58	0	0:02.42
51	T	96	93	3	0:03.95
52	T	58	58	0	0:02.41
53	T	58	58	0	0:02.38
54	T	58	58	0	0:02.45
55	T	58	58	0	0:02.40
56	T	83	81	2	0:03.46
57	T	69	68	1	0:02.86
58	T	69	68	1	0:02.83
59	T	58	58	0	0:02.46
60	T	69	68	1	0:02.85
61	T	83	81	2	0:03.39
62	T	58	58	0	0:02.40
63	T	58	58	0	0:02.41
64	T	58	58	0	0:02.43
65	T	58	58	0	0:02.40
66	T	58	58	0	0:02.45
67	T	58	58	0	0:03.34
68	T	83	81	2	0:03.43
69	T	83	81	2	0:03.41
70	T	58	58	0	0:02.43
71	T	58	58	0	0:02.44
72	T	69	68	1	0:02.92
73	T	58	58	0	0:02.46
74	T	96	93	3	0:03.94
75	T	83	81	2	0:03.45
76	T	58	58	0	0:02.50
77	T	69	68	1	0:02.86
78	T	69	68	1	0:02.85
79	T	69	68	1	0:02.95
80	T	69	68	1	0:02.85
81	T	58	58	0	0:02.42
82	T	58	58	0	0:02.39

Id	Result	#total	#true	#false	Execution time (min:sec)
83	T	69	68	1	0:02.89
84	T	58	58	0	0:02.44
85	T	58	58	0	0:02.38
86	T	83	81	2	0:03.49
87	T	58	58	0	0:02.44
88	T	69	68	1	0:02.90
89	T	58	58	0	0:02.40
90	T	69	68	1	0:02.83
91	T	83	81	2	0:03.40
92	T	69	68	1	0:02.88
93	T	58	58	0	0:02.42
94	T	69	68	1	0:02.88
95	T	69	68	1	0:02.81
96	T	69	68	1	0:02.83
97	T	58	58	0	0:02.42
98	T	58	58	0	0:02.46
99	T	58	58	0	0:02.40
100	T	69	68	1	0:02.92
101	T	58	58	0	0:02.45
102	T	83	81	2	0:03.50
103	T	58	58	0	0:02.42
104	T	58	58	0	0:02.43
105	T	69	68	1	0:02.85
106	T	83	81	2	0:03.41
107	T	83	81	2	0:03.46
108	T	58	58	0	0:02.40
109	T	58	58	0	0:02.40
110	T	58	58	0	0:02.47
111	T	58	58	0	0:02.43
112	T	58	58	0	0:02.41
113	T	83	81	2	0:03.48
114	T	69	68	1	0:02.89
115	T	83	81	2	0:03.44
116	T	83	81	2	0:03.42
117	T	58	58	0	0:02.46
118	T	83	81	2	0:04.94
119	T	96	93	3	0:03.98
120	T	58	58	0	0:02.44
121	T	69	68	1	0:02.91
122	T	69	68	1	0:02.92
123	T	58	58	0	0:02.45
124	T	69	68	1	0:03.54
125	T	69	68	1	0:02.87
126	T	58	58	0	0:02.47
127	T	69	68	1	0:02.85
128	T	69	68	1	0:02.92
129	T	58	58	0	0:02.42
130	T	58	58	0	0:02.42

Id	Result	#total	#true	#false	Execution time (min:sec)
131	T	58	58	0	0:02.39
132	T	58	58	0	0:02.42
133	T	58	58	0	0:02.45
134	T	69	68	1	0:03.55
135	T	58	58	0	0:02.45
136	T	69	68	1	0:02.91
137	T	69	68	1	0:02.92
138	T	69	68	1	0:02.47
139	T	69	68	1	0:02.90
140	T	58	58	0	0:02.43
141	T	58	58	0	0:02.43
142	T	69	68	1	0:02.84
143	T	58	58	0	0:02.43
144	T	58	58	0	0:02.42
145	T	58	58	0	0:02.73
146	T	83	81	2	0:03.42
147	T	96	93	3	0:04.04
148	T	69	68	1	0:03.03
149	T	58	58	0	0:02.44
150	T	110	106	4	0:04.55
151	T	69	68	1	0:02.82
152	T	58	58	0	0:02.41
153	T	58	58	0	0:02.47
154	T	58	58	0	0:03.42
155	T	69	68	1	0:02.88
156	T	58	58	0	0:02.42
157	T	69	68	1	0:02.85
158	T	83	81	2	0:03.40
159	T	58	58	0	0:02.40
160	T	83	81	2	0:03.41
161	T	58	58	0	0:02.42
162	T	58	58	0	0:02.46
163	T	58	58	0	0:02.41
164	T	69	68	1	0:02.86
165	T	69	68	1	0:02.87
166	T	83	81	2	0:03.41
167	T	58	58	0	0:02.43
168	T	69	68	1	0:02.92
169	T	58	58	0	0:02.40
170	T	58	58	0	0:02.41
171	T	96	93	3	0:03.97
172	T	110	106	4	0:04.50
173	T	58	58	0	0:02.44
174	T	83	81	2	0:03.43
175	T	96	93	3	0:03.98
176	T	96	93	3	0:03.99
177	T	58	58	0	0:03.46
178	T	69	68	1	0:02.86

**Id**	**Result**	**#total**	**#true**	**#false**	**Execution time (min:sec)**
179	T	69	68	1	0:02.87
180	T	69	68	1	0:02.89
181	T	58	58	0	0:02.45
182	T	58	58	0	0:02.46
183	T	83	81	2	0:03.42
184	T	58	58	0	0:02.45
185	T	96	93	3	0:03.95
186	T	58	58	0	0:02.39
187	T	110	106	4	0:04.56
188	T	58	58	0	0:02.43
189	T	69	68	1	0:02.85
190	T	58	58	0	0:02.44
191	T	96	93	3	0:03.96
192	T	69	68	1	0:02.85
193	T	58	58	0	0:02.45
194	T	83	81	2	0:03.46
195	T	83	81	2	0:03.42
196	T	69	68	1	0:02.93
197	T	83	81	2	0:03.43
198	T	58	58	0	0:02.41
199	T	58	58	0	0:03.46
200	T	96	93	3	0:03.94
201	T	69	68	1	0:02.88
202	T	58	58	0	0:02.41
203	T	58	58	0	0:02.44
204	T	69	68	1	0:02.84
205	T	58	58	0	0:02.41
206	T	83	81	2	0:03.48
207	T	58	58	0	0:02.44
208	T	58	58	0	0:02.45
209	T	58	58	0	0:02.44
210	T	58	58	0	0:02.42
211	T	69	68	1	0:02.84
212	T	58	58	0	0:02.44
213	T	58	58	0	0:02.40
214	T	58	58	0	0:02.44
215	T	58	58	0	0:02.39
216	T	58	58	0	0:03.85
217	T	58	58	0	0:02.39
218	T	58	58	0	0:02.43
219	T	58	58	0	0:02.43
220	T	69	68	1	0:02.91
221	T	96	93	3	0:04.00
222	T	58	58	0	0:02.41
223	T	58	58	0	0:02.42
224	T	69	68	1	0:03.60
225	T	69	68	1	0:02.84
226	T	83	81	2	0:03.42

Id	Result	#total	#true	#false	Execution time (min:sec)
227	T	83	81	2	0:03.46
228	T	69	68	1	0:03.63
229	T	58	58	0	0:02.48
230	T	58	58	0	0:02.43
231	T	58	58	0	0:02.44
232	T	83	81	2	0:03.43
233	T	69	68	1	0:02.89
234	T	69	68	1	0:03.02
235	T	83	81	2	0:03.43
236	T	58	58	0	0:02.40
237	T	83	81	2	0:03.44
238	T	58	58	0	0:02.46
239	T	83	81	2	0:03.45
240	T	69	68	1	0:02.88
241	T	110	106	4	0:04.52
242	T	58	58	0	0:02.44
243	T	69	68	1	0:02.90
244	T	69	68	1	0:02.88
245	T	69	68	1	0:02.92
246	T	96	93	3	0:03.93
247	T	69	68	1	0:02.87
248	T	58	58	0	0:02.45
249	T	69	68	1	0:02.89
250	T	69	68	1	0:02.87
251	T	69	68	1	0:02.93
252	T	69	68	1	0:02.83
253	T	83	81	2	0:03.46
254	T	69	68	1	0:02.86
255	T	58	58	0	0:02.38
256	T	58	58	0	0:02.42
257	T	58	58	0	0:02.41
258	T	58	58	0	0:02.42
259	T	69	68	1	0:04.35
260	T	69	68	1	0:02.89
261	T	83	81	2	0:03.40
262	T	69	68	1	0:03.56
263	T	69	68	1	0:02.85
264	T	58	58	0	0:02.43
265	T	58	58	0	0:02.39
266	T	58	58	0	0:02.43
267	T	58	58	0	0:02.43
268	T	58	58	0	0:02.40
269	T	58	58	0	0:02.44
270	T	83	81	2	0:03.44
271	T	69	68	1	0:02.91
272	T	83	81	2	0:03.45
273	T	58	58	0	0:02.41
274	T	58	58	0	0:02.43

Id	Result	#total	#true	#false	Execution time (min:sec)
275	T	58	58	0	0:02.44
276	T	69	68	1	0:02.89
277	T	69	68	1	0:02.84
278	T	69	68	1	0:02.85
279	T	58	58	0	0:02.43
280	T	69	68	1	0:02.87
281	T	69	68	1	0:02.89
282	T	69	68	1	0:02.91
283	T	58	58	0	0:02.42
284	T	69	68	1	0:02.86
285	T	69	68	1	0:02.91
286	T	69	68	1	0:02.85
287	T	58	58	0	0:02.43
288	T	69	68	1	0:02.88
289	T	83	81	2	0:03.45
290	T	58	58	0	0:02.42
291	T	69	68	1	0:02.89
292	T	69	68	1	0:02.86
293	T	58	58	0	0:02.45
294	T	83	81	2	0:05.13
295	T	58	58	0	0:02.38
296	T	69	68	1	0:03.46
297	T	69	68	1	0:02.81
298	T	58	58	0	0:02.43
299	T	58	58	0	0:02.43
300	T	83	81	2	0:03.41
301	T	69	68	1	0:02.87
302	T	69	68	1	0:02.90
303	T	124	119	5	0:06.67
304	T	69	68	1	0:02.87
305	T	58	58	0	0:02.44
306	T	58	58	0	0:02.47
307	T	96	93	3	0:04.06
308	T	58	58	0	0:02.75
309	T	69	68	1	0:03.19
310	T	69	68	1	0:02.87
311	T	83	81	2	0:03.44
312	T	69	68	1	0:02.90
313	T	58	58	0	0:02.44
314	T	69	68	1	0:02.88
315	T	58	58	0	0:02.41
316	T	69	68	1	0:02.86
317	T	69	68	1	0:02.92
318	T	58	58	0	0:02.41
319	T	58	58	0	0:02.43
320	T	69	68	1	0:02.86
321	T	69	68	1	0:02.85
322	T	58	58	0	0:02.43

Id	Result	#total	#true	#false	Execution time (min:sec)
323	T	58	58	0	0:02.42
324	T	58	58	0	0:02.41
325	T	69	68	1	0:02.86
326	T	69	68	1	0:02.90
327	T	69	68	1	0:02.84
328	T	58	58	0	0:02.41
329	T	58	58	0	0:02.40
330	T	58	58	0	0:02.47
331	T	58	58	0	0:02.43
332	T	83	81	2	0:03.46
333	T	58	58	0	0:02.43
334	T	124	119	5	0:05.16
335	T	58	58	0	0:02.42
336	T	69	68	1	0:02.86
337	T	58	58	0	0:02.41
338	T	69	68	1	0:02.84
339	T	58	58	0	0:02.43
340	T	58	58	0	0:02.43
341	T	58	58	0	0:02.38
342	T	69	68	1	0:02.88
343	T	58	58	0	0:02.42
344	T	69	68	1	0:02.86
345	T	58	58	0	0:02.46
346	T	58	58	0	0:02.41
347	T	69	68	1	0:02.90
348	T	69	68	1	0:02.85
349	T	58	58	0	0:02.41
350	T	83	81	2	0:03.45
351	T	69	68	1	0:02.84
352	T	58	58	0	0:02.45
353	T	58	58	0	0:02.40
354	T	58	58	0	0:02.39
355	T	58	58	0	0:02.45
356	T	69	68	1	0:02.86
357	T	58	58	0	0:02.45
358	T	58	58	0	0:02.43
359	T	83	81	2	0:03.42
360	T	69	68	1	0:03.45
361	T	58	58	0	0:03.60
362	T	69	68	1	0:02.83
363	T	69	68	1	0:02.85
364	T	69	68	1	0:02.87
365	T	83	81	2	0:03.43
366	T	58	58	0	0:02.46
367	T	83	81	2	0:03.44
368	T	110	106	4	0:04.54
369	T	69	68	1	0:02.89
370	T	83	81	2	0:03.44

Id	Result	#total	#true	#false	Execution time (min:sec)
371	T	69	68	1	0:02.89
372	T	58	58	0	0:02.44
373	T	83	81	2	0:03.41
374	T	83	81	2	0:03.44
375	T	58	58	0	0:02.41
376	T	58	58	0	0:02.43
377	T	58	58	0	0:02.38
378	T	58	58	0	0:02.42
379	T	58	58	0	0:02.38
380	T	58	58	0	0:02.37
381	T	58	58	0	0:02.39
382	T	83	81	2	0:03.44
383	T	58	58	0	0:02.39
384	T	58	58	0	0:02.42
385	T	69	68	1	0:02.82
386	T	58	58	0	0:02.45
387	T	69	68	1	0:02.90
388	T	83	81	2	0:03.45
389	T	69	68	1	0:02.84
390	T	69	68	1	0:02.85
391	T	69	68	1	0:02.86
392	T	58	58	0	0:02.40
393	T	58	58	0	0:02.39
394	T	58	58	0	0:02.39
395	T	58	58	0	0:02.44
396	T	83	81	2	0:03.48
397	T	69	68	1	0:02.89
398	T	69	68	1	0:02.86
399	T	83	81	2	0:03.40
400	T	58	58	0	0:02.45
401	T	69	68	1	0:02.85
402	T	58	58	0	0:02.43
403	T	110	106	4	0:04.59
404	T	58	58	0	0:02.45
405	T	58	58	0	0:02.42
406	T	58	58	0	0:02.40
407	T	58	58	0	0:03.61
408	T	58	58	0	0:02.38
409	T	58	58	0	0:02.39
410	T	58	58	0	0:02.76
411	T	58	58	0	0:02.43
412	T	58	58	0	0:02.46
413	T	69	68	1	0:02.91
414	T	58	58	0	0:02.38
415	T	69	68	1	0:02.85
416	T	58	58	0	0:02.39
417	T	58	58	0	0:02.38
418	T	58	58	0	0:02.46

Id	Result	#total	#true	#false	Execution time (min:sec)
419	T	69	68	1	0:02.83
420	T	58	58	0	0:02.39
421	T	69	68	1	0:02.85
422	T	69	68	1	0:02.88
423	T	83	81	2	0:03.44
424	T	58	58	0	0:02.40
425	T	83	81	2	0:03.45
426	T	69	68	1	0:02.90
427	T	69	68	1	0:02.89
428	T	58	58	0	0:02.44
429	T	69	68	1	0:02.89
430	T	96	93	3	0:05.60
431	T	69	68	1	0:02.85
432	T	69	68	1	0:02.81
433	T	83	81	2	0:03.41
434	T	58	58	0	0:02.42
435	T	58	58	0	0:02.41
436	T	69	68	1	0:02.88
437	T	58	58	0	0:02.39
438	T	58	58	0	0:02.39
439	T	58	58	0	0:02.46
440	T	58	58	0	0:02.40
441	T	69	68	1	0:02.87
442	T	83	81	2	0:03.46
443	T	83	81	2	0:03.43
444	T	58	58	0	0:02.49
445	T	83	81	2	0:03.42
446	T	83	81	2	0:03.41
447	T	58	58	0	0:02.44
448	T	96	93	3	0:04.31
449	T	124	119	5	0:05.17
450	T	96	93	3	0:03.96
451	T	69	68	1	0:02.89
452	T	58	58	0	0:02.44
453	T	96	93	3	0:04.02
454	T	58	58	0	0:02.40
455	T	58	58	0	0:02.43
456	T	69	68	1	0:02.88
457	T	58	58	0	0:02.46
458	T	69	68	1	0:02.85
459	T	58	58	0	0:02.40
460	T	58	58	0	0:02.40
461	T	69	68	1	0:02.84
462	T	69	68	1	0:02.88
463	T	58	58	0	0:02.39
464	T	58	58	0	0:02.41
465	T	58	58	0	0:02.39
466	T	83	81	2	0:03.43

Id	Result	#total	#true	#false	Execution time (min:sec)
467	T	96	93	3	0:03.99
468	T	58	58	0	0:02.41
469	T	58	58	0	0:02.42
470	T	58	58	0	0:02.40
471	T	69	68	1	0:02.84
472	T	58	58	0	0:02.37
473	T	58	58	0	0:02.47
474	T	96	93	3	0:03.98
475	T	83	81	2	0:03.46
476	T	83	81	2	0:03.41
477	T	58	58	0	0:02.40
478	T	58	58	0	0:02.44
479	T	69	68	1	0:02.85
480	T	83	81	2	0:03.44
481	T	58	58	0	0:02.39
482	T	69	68	1	0:02.86
483	T	69	68	1	0:02.88
484	T	69	68	1	0:02.88
485	T	69	68	1	0:02.83
486	T	58	58	0	0:02.43
487	T	83	81	2	0:03.42
488	T	58	58	0	0:02.43
489	T	69	68	1	0:02.80
490	T	58	58	0	0:02.40
491	T	58	58	0	0:02.43
492	T	58	58	0	0:02.97
493	T	83	81	2	0:03.43
494	T	83	81	2	0:03.44
495	T	69	68	1	0:02.85
496	T	69	68	1	0:02.87
497	T	58	58	0	0:02.43
498	T	83	81	2	0:03.49
499	T	69	68	1	0:02.83
500	T	83	81	2	0:03.48

Table 2: Model checking results corresponding to logic property 2

Id	Result	#total	#true	#false	Execution time (min:sec)
1	T	142	1	141	0:05.83
2	T	225	4	221	0:09.19
3	T	169	2	167	0:09.95
4	T	169	2	167	0:10.88
5	T	197	3	194	0:12.61
6	T	225	4	221	0:10.41
7	T	58	0	58	0:02.38

Id	Result	#total	#true	#false	Execution time (min:sec)
8	T	58	0	58	0:03.03
9	T	58	0	58	0:02.40
10	T	58	0	58	0:02.40
11	T	142	1	141	0:05.81
12	T	58	0	58	0:02.40
13	T	142	1	141	0:05.83
14	T	142	1	141	0:05.81
15	T	197	3	194	0:08.31
16	T	58	0	58	0:02.42
17	T	58	0	58	0:02.43
18	T	142	1	141	0:05.83
19	T	58	0	58	0:03.47
20	T	58	0	58	0:02.40
21	T	58	0	58	0:02.39
22	T	252	5	247	0:16.79
23	T	58	0	58	0:02.41
24	T	169	2	167	0:06.95
25	T	58	0	58	0:02.47
26	T	58	0	58	0:02.45
27	T	58	0	58	0:02.40
28	T	58	0	58	0:02.40
29	T	58	0	58	0:02.38
30	T	142	1	141	0:05.83
31	T	58	0	58	0:02.45
32	T	169	2	167	0:07.00
33	T	58	0	58	0:02.41
34	T	142	1	141	0:05.86
35	T	142	1	141	0:05.79
36	T	142	1	141	0:05.85
37	T	197	3	194	0:08.04
38	T	58	0	58	0:02.40
39	T	142	1	141	0:09.28
40	T	169	2	167	0:07.01
41	T	142	1	141	0:05.75
42	T	142	1	141	0:06.91
43	T	197	3	194	0:12.41
44	T	142	1	141	0:05.82
45	T	58	0	58	0:02.43
46	T	169	2	167	0:06.94
47	T	58	0	58	0:02.44
48	T	58	0	58	0:02.44
49	T	169	2	167	0:06.91
50	T	58	0	58	0:02.39
51	T	142	1	141	0:05.81
52	T	58	0	58	0:02.41
53	T	197	3	194	0:08.00
54	T	169	2	167	0:06.98
55	T	58	0	58	0:02.43

Id	Result	#total	#true	#false	Execution time (min:sec)
56	T	58	0	58	0:02.41
57	T	58	0	58	0:02.42
58	T	197	3	194	0:12.47
59	T	58	0	58	0:02.41
60	T	197	3	194	0:08.06
61	T	58	0	58	0:02.44
62	T	169	2	167	0:06.92
63	T	58	0	58	0:02.43
64	T	197	3	194	0:08.06
65	T	58	0	58	0:02.38
66	T	58	0	58	0:02.42
67	T	58	0	58	0:03.38
68	T	197	3	194	0:08.04
69	T	58	0	58	0:02.40
70	T	58	0	58	0:02.42
71	T	58	0	58	0:02.99
72	T	58	0	58	0:02.39
73	T	58	0	58	0:02.48
74	T	58	0	58	0:02.45
75	T	142	1	141	0:05.79
76	T	58	0	58	0:02.40
77	T	58	0	58	0:02.41
78	T	169	2	167	0:06.87
79	T	58	0	58	0:02.38
80	T	169	2	167	0:06.94
81	T	58	0	58	0:02.46
82	T	197	3	194	0:08.04
83	T	58	0	58	0:02.42
84	T	142	1	141	0:05.80
85	T	58	0	58	0:02.00
86	T	142	1	141	0:05.81
87	T	58	0	58	0:02.45
88	T	58	0	58	0:02.42
89	T	58	0	58	0:02.40
90	T	58	0	58	0:02.40
91	T	58	0	58	0:02.42
92	T	169	2	167	0:06.91
93	T	58	0	58	0:02.84
94	T	58	0	58	0:02.46
95	T	142	1	141	0:05.83
96	T	58	0	58	0:02.40
97	T	58	0	58	0:02.44
98	T	197	3	194	0:08.08
99	T	58	0	58	0:02.40
100	T	169	2	167	0:06.89
101	T	58	0	58	0:02.42
102	T	58	0	58	0:02.42
103	T	58	0	58	0:02.43

Id	Result	#total	#true	#false	Execution time (min:sec)
104	T	58	0	58	0:02.45
105	T	142	1	141	0:05.86
106	T	58	0	58	0:02.39
107	T	169	2	167	0:07.17
108	T	58	0	58	0:02.39
109	T	169	2	167	0:06.88
110	T	142	1	141	0:05.86
111	T	169	2	167	0:06.89
112	T	225	4	221	0:09.27
113	T	197	3	194	0:07.98
114	T	197	3	194	0:08.08
115	T	225	4	221	0:09.24
116	T	142	1	141	0:05.87
117	T	58	0	58	0:02.43
118	T	58	0	58	0:02.43
119	T	58	0	58	0:02.43
120	T	197	3	194	0:08.02
121	T	225	4	221	0:09.19
122	T	58	0	58	0:02.43
123	T	169	2	167	0:06.92
124	T	58	0	58	0:02.43
125	T	197	3	194	0:07.37
126	T	58	0	58	0:02.45
127	T	58	0	58	0:02.44
128	T	58	0	58	0:02.39
129	T	169	2	167	0:06.98
130	T	58	0	58	0:02.46
131	T	142	1	141	0:05.81
132	T	169	2	167	0:06.93
133	T	142	1	141	0:05.84
134	T	58	0	58	0:02.46
135	T	58	0	58	0:02.41
136	T	169	2	167	0:06.97
137	T	58	0	58	0:02.44
138	T	197	3	194	0:08.08
139	T	142	1	141	0:05.86
140	T	197	3	194	0:08.10
141	T	58	0	58	0:02.43
142	T	58	0	58	0:02.42
143	T	142	1	141	0:05.82
144	T	197	3	194	0:08.07
145	T	169	2	167	0:06.94
146	T	58	0	58	0:02.41
147	T	58	0	58	0:02.62
148	T	142	1	141	0:05.82
149	T	58	0	58	0:02.37
150	T	58	0	58	0:02.43
151	T	58	0	58	0:02.47

Id	Result	#total	#true	#false	Execution time (min:sec)
152	T	58	0	58	0:02.43
153	T	142	1	141	0:05.82
154	T	142	1	141	0:05.82
155	T	58	0	58	0:02.41
156	T	58	0	58	0:02.44
157	T	58	0	58	0:02.42
158	T	58	0	58	0:02.40
159	T	142	1	141	0:05.89
160	T	58	0	58	0:02.43
161	T	58	0	58	0:02.41
162	T	58	0	58	0:02.45
163	T	169	2	167	0:06.89
164	T	169	2	167	0:06.94
165	T	58	0	58	0:02.39
166	T	58	0	58	0:02.39
167	T	197	3	194	0:08.06
168	T	58	0	58	0:02.45
169	T	142	1	141	0:05.82
170	T	58	0	58	0:02.42
171	T	142	1	141	0:05.96
172	T	58	0	58	0:02.40
173	T	169	2	167	0:06.92
174	T	169	2	167	0:06.95
175	T	58	0	58	0:02.41
176	T	58	0	58	0:02.41
177	T	58	0	58	0:02.46
178	T	58	0	58	0:02.43
179	T	142	1	141	0:07.77
180	T	58	0	58	0:02.42
181	T	58	0	58	0:02.39
182	T	197	3	194	0:11.33
183	T	58	0	58	0:03.54
184	T	197	3	194	0:08.05
185	T	58	0	58	0:02.44
186	T	58	0	58	0:02.42
187	T	58	0	58	0:02.41
188	T	58	0	58	0:02.66
189	T	142	1	141	0:05.83
190	T	169	2	167	0:06.93
191	T	58	0	58	0:02.49
192	T	142	1	141	0:05.80
193	T	58	0	58	0:02.41
194	T	58	0	58	0:02.40
195	T	58	0	58	0:02.44
196	T	58	0	58	0:02.44
197	T	58	0	58	0:02.38
198	T	58	0	58	0:02.43
199	T	58	0	58	0:02.42

Id	Result	#total	#true	#false	Execution time (min:sec)
200	T	58	0	58	0:02.45
201	T	142	1	141	0:08.38
202	T	169	2	167	0:06.94
203	T	58	0	58	0:02.42
204	T	142	1	141	0:05.78
205	T	58	0	58	0:02.43
206	T	58	0	58	0:02.42
207	T	142	1	141	0:03.90
208	T	142	1	141	0:05.81
209	T	142	1	141	0:05.78
210	T	58	0	58	0:02.41
211	T	169	2	167	0:07.04
212	T	58	0	58	0:02.43
213	T	58	0	58	0:02.44
214	T	58	0	58	0:02.44
215	T	197	3	194	0:08.04
216	T	58	0	58	0:02.44
217	T	58	0	58	0:02.44
218	T	58	0	58	0:02.41
219	T	58	0	58	0:02.41
220	T	169	2	167	0:06.94
221	T	58	0	58	0:02.40
222	T	169	2	167	0:06.91
223	T	225	4	221	0:09.21
224	T	197	3	194	0:08.08
225	T	58	0	58	0:02.41
226	T	58	0	58	0:02.42
227	T	58	0	58	0:02.42
228	T	58	0	58	0:02.46
229	T	58	0	58	0:02.42
230	T	169	2	167	0:06.91
231	T	169	2	167	0:06.92
232	T	197	3	194	0:07.84
233	T	58	0	58	0:02.37
234	T	58	0	58	0:02.42
235	T	58	0	58	0:02.42
236	T	58	0	58	0:03.82
237	T	58	0	58	0:02.44
238	T	58	0	58	0:02.43
239	T	58	0	58	0:02.44
240	T	58	0	58	0:02.45
241	T	58	0	58	0:02.42
242	T	142	1	141	0:05.85
243	T	169	2	167	0:06.98
244	T	58	0	58	0:02.38
245	T	58	0	58	0:02.44
246	T	58	0	58	0:02.46
247	T	169	2	167	0:06.91

Id	Result	#total	#true	#false	Execution time (min:sec)
248	T	58	0	58	0:02.42
249	T	142	1	141	0:05.79
250	T	142	1	141	0:05.92
251	T	169	2	167	0:06.89
252	T	58	0	58	0:02.38
253	T	58	0	58	0:02.48
254	T	58	0	58	0:02.42
255	T	58	0	58	0:02.38
256	T	58	0	58	0:02.45
257	T	169	2	167	0:06.90
258	T	142	1	141	0:05.81
259	T	225	4	221	0:09.25
260	T	58	0	58	0:02.41
261	T	58	0	58	0:02.45
262	T	58	0	58	0:02.44
263	T	169	2	167	0:06.92
264	T	58	0	58	0:02.40
265	T	58	0	58	0:02.46
266	T	58	0	58	0:02.43
267	T	58	0	58	0:02.42
268	T	58	0	58	0:02.40
269	T	58	0	58	0:02.41
270	T	58	0	58	0:02.43
271	T	58	0	58	0:02.41
272	T	58	0	58	0:02.45
273	T	58	0	58	0:02.42
274	T	58	0	58	0:02.42
275	T	58	0	58	0:02.43
276	T	142	1	141	0:05.89
277	T	58	0	58	0:02.39
278	T	58	0	58	0:02.43
279	T	142	1	141	0:05.92
280	T	58	0	58	0:02.43
281	T	142	1	141	0:05.83
282	T	58	0	58	0:02.45
283	T	58	0	58	0:02.42
284	T	58	0	58	0:02.44
285	T	197	3	194	0:08.08
286	T	58	0	58	0:02.40
287	T	169	2	167	0:06.92
288	T	169	2	167	0:06.90
289	T	280	6	274	0:11.49
290	T	58	0	58	0:02.46
291	T	58	0	58	0:02.23
292	T	58	0	58	0:02.45
293	T	225	4	221	0:09.19
294	T	58	0	58	0:02.45
295	T	58	0	58	0:02.42

Id	Result	#total	#true	#false	Execution time (min:sec)
296	T	169	2	167	0:09.16
297	T	58	0	58	0:02.41
298	T	225	4	221	0:09.35
299	T	225	4	221	0:10.63
300	T	58	0	58	0:02.46
301	T	142	1	141	0:05.85
302	T	58	0	58	0:02.39
303	T	58	0	58	0:02.41
304	T	58	0	58	0:02.41
305	T	58	0	58	0:02.40
306	T	197	3	194	0:08.06
307	T	58	0	58	0:02.41
308	T	58	0	58	0:02.43
309	T	58	0	58	0:02.41
310	T	58	0	58	0:02.49
311	T	225	4	221	0:09.22
312	T	58	0	58	0:02.40
313	T	58	0	58	0:02.44
314	T	169	2	167	0:07.01
315	T	58	0	58	0:02.41
316	T	58	0	58	0:02.39
317	T	58	0	58	0:02.46
318	T	197	3	194	0:08.05
319	T	142	1	141	0:05.84
320	T	58	0	58	0:02.40
321	T	142	1	141	0:05.88
322	T	142	1	141	0:05.91
323	T	58	0	58	0:02.41
324	T	169	2	167	0:06.93
325	T	58	0	58	0:02.42
326	T	58	0	58	0:02.47
327	T	169	2	167	0:06.96
328	T	58	0	58	0:02.44
329	T	58	0	58	0:02.40
330	T	169	2	167	0:06.95
331	T	58	0	58	0:02.42
332	T	58	0	58	0:02.44
333	T	58	0	58	0:02.47
334	T	169	2	167	0:06.93
335	T	58	0	58	0:02.42
336	T	58	0	58	0:02.41
337	T	142	1	141	0:05.86
338	T	58	0	58	0:02.41
339	T	58	0	58	0:02.47
340	T	58	0	58	0:02.42
341	T	58	0	58	0:02.44
342	T	58	0	58	0:02.38
343	T	197	3	194	0:08.05

Id	Result	#total	#true	#false	Execution time (min:sec)
344	T	169	2	167	0:06.94
345	T	225	4	221	0:09.22
346	T	252	5	247	0:10.44
347	T	58	0	58	0:02.43
348	T	58	0	58	0:02.40
349	T	58	0	58	0:02.44
350	T	142	1	141	0:05.81
351	T	142	1	141	0:05.81
352	T	197	3	194	0:08.04
353	T	58	0	58	0:02.42
354	T	58	0	58	0:02.40
355	T	58	0	58	0:02.40
356	T	58	0	58	0:02.42
357	T	58	0	58	0:02.41
358	T	58	0	58	0:02.41
359	T	197	3	194	0:08.09
360	T	142	1	141	0:05.87
361	T	142	1	141	0:05.82
362	T	169	2	167	0:09.73
363	T	58	0	58	0:03.09
364	T	58	0	58	0:02.43
365	T	58	0	58	0:02.42
366	T	58	0	58	0:02.39
367	T	58	0	58	0:02.40
368	T	197	3	194	0:08.04
369	T	58	0	58	0:02.42
370	T	58	0	58	0:02.45
371	T	58	0	58	0:02.45
372	T	58	0	58	0:02.40
373	T	142	1	141	0:05.85
374	T	169	2	167	0:06.88
375	T	58	0	58	0:02.41
376	T	142	1	141	0:05.85
377	T	169	2	167	0:06.91
378	T	169	2	167	0:06.93
379	T	58	0	58	0:02.41
380	T	169	2	167	0:04.66
381	T	169	2	167	0:06.95
382	T	142	1	141	0:05.80
383	T	197	3	194	0:09.70
384	T	169	2	167	0:06.97
385	T	197	3	194	0:08.02
386	T	252	5	247	0:14.55
387	T	58	0	58	0:02.44
388	T	142	1	141	0:05.78
389	T	169	2	167	0:06.90
390	T	169	2	167	0:06.98
391	T	58	0	58	0:02.42

Id	Result	#total	#true	#false	Execution time (min:sec)
392	T	58	0	58	0:02.44
393	T	142	1	141	0:05.80
394	T	169	2	167	0:07.00
395	T	58	0	58	0:02.44
396	T	197	3	194	0:08.16
397	T	58	0	58	0:02.43
398	T	142	1	141	0:05.80
399	T	225	4	221	0:09.29
400	T	58	0	58	0:02.42
401	T	169	2	167	0:06.95
402	T	58	0	58	0:02.62
403	T	58	0	58	0:02.46
404	T	58	0	58	0:02.43
405	T	58	0	58	0:02.48
406	T	58	0	58	0:02.40
407	T	142	1	141	0:05.86
408	T	58	0	58	0:02.38
409	T	142	1	141	0:05.85
410	T	225	4	221	0:09.21
411	T	58	0	58	0:02.44
412	T	58	0	58	0:02.42
413	T	58	0	58	0:02.39
414	T	142	1	141	0:06.08
415	T	58	0	58	0:02.39
416	T	58	0	58	0:02.46
417	T	58	0	58	0:02.40
418	T	58	0	58	0:02.45
419	T	58	0	58	0:03.80
420	T	58	0	58	0:02.44
421	T	169	2	167	0:07.22
422	T	58	0	58	0:02.40
423	T	142	1	141	0:05.04
424	T	58	0	58	0:02.44
425	T	58	0	58	0:02.44
426	T	169	2	167	0:06.95
427	T	58	0	58	0:02.45
428	T	58	0	58	0:02.44
429	T	142	1	141	0:05.85
430	T	169	2	167	0:06.93
431	T	58	0	58	0:02.40
432	T	58	0	58	0:02.42
433	T	58	0	58	0:02.43
434	T	58	0	58	0:02.41
435	T	58	0	58	0:02.42
436	T	58	0	58	0:02.43
437	T	58	0	58	0:02.43
438	T	58	0	58	0:02.39
439	T	58	0	58	0:03.71

Id	Result	#total	#true	#false	Execution time (min:sec)
440	T	142	1	141	0:08.79
441	T	142	1	141	0:05.82
442	T	58	0	58	0:02.37
443	T	169	2	167	0:06.92
444	T	58	0	58	0:02.41
445	T	142	1	141	0:05.87
446	T	58	0	58	0:02.44
447	T	58	0	58	0:02.46
448	T	58	0	58	0:02.44
449	T	142	1	141	0:05.88
450	T	142	1	141	0:05.87
451	T	142	1	141	0:05.79
452	T	58	0	58	0:02.41
453	T	58	0	58	0:02.44
454	T	169	2	167	0:09.66
455	T	58	0	58	0:02.42
456	T	142	1	141	0:05.83
457	T	58	0	58	0:02.46
458	T	58	0	58	0:03.90
459	T	58	0	58	0:02.41
460	T	58	0	58	0:02.40
461	T	142	1	141	0:05.83
462	T	58	0	58	0:02.42
463	T	58	0	58	0:02.41
464	T	58	0	58	0:02.41
465	T	58	0	58	0:02.42
466	T	225	4	221	0:09.24
467	T	169	2	167	0:06.98
468	T	169	2	167	0:06.91
469	T	169	2	167	0:06.90
470	T	58	0	58	0:02.45
471	T	197	3	194	0:11.06
472	T	58	0	58	0:02.48
473	T	169	2	167	0:05.88
474	T	58	0	58	0:02.37
475	T	58	0	58	0:02.42
476	T	58	0	58	0:02.41
477	T	58	0	58	0:02.38
478	T	58	0	58	0:02.41
479	T	169	2	167	0:06.93
480	T	58	0	58	0:02.42
481	T	142	1	141	0:05.83
482	T	58	0	58	0:02.44
483	T	58	0	58	0:02.43
484	T	58	0	58	0:02.39
485	T	142	1	141	0:05.78
486	T	58	0	58	0:02.43
487	T	142	1	141	0:05.87

Id	Result	#total	#true	#false	Execution time (min:sec)
488	T	169	2	167	0:11.26
489	T	58	0	58	0:02.44
490	T	58	0	58	0:02.39
491	T	58	0	58	0:02.43
492	T	169	2	167	0:06.94
493	T	197	3	194	0:08.15
494	T	58	0	58	0:02.43
495	T	142	1	141	0:05.92
496	T	58	0	58	0:02.41
497	T	142	1	141	0:05.78
498	T	58	0	58	0:02.42
499	T	58	0	58	0:02.39
500	T	58	0	58	0:02.41

Table 3: Model checking results corresponding to logic property 3

Id	Result	#total	#true	#false	Execution time (min:sec)
1	T	58	58	0	0:02.46
2	T	58	58	0	0:02.48
3	T	58	58	0	0:02.48
4	T	58	58	0	0:02.47
5	T	58	58	0	0:02.46
6	T	69	68	1	0:02.93
7	T	69	68	1	0:02.91
8	T	58	58	0	0:02.50
9	T	58	58	0	0:02.49
10	T	58	58	0	0:02.50
11	T	83	81	2	0:03.50
12	T	58	58	0	0:02.68
13	T	58	58	0	0:03.79
14	T	58	58	0	0:02.48
15	T	58	58	0	0:02.51
16	T	58	58	0	0:02.47
17	T	58	58	0	0:02.53
18	T	58	58	0	0:02.52
19	T	58	58	0	0:02.55
20	T	69	68	1	0:02.99
21	T	58	58	0	0:02.46
22	T	69	68	1	0:02.97
23	T	69	68	1	0:02.95
24	T	58	58	0	0:02.52
25	T	58	58	0	0:03.48
26	T	58	58	0	0:02.50
27	T	58	58	0	0:02.56
28	T	69	68	1	0:02.94

Id	Result	#total	#true	#false	Execution time (min:sec)
29	T	69	68	1	0:02.92
30	T	69	68	1	0:02.92
31	T	58	58	0	0:02.48
32	T	58	58	0	0:02.48
33	T	69	68	1	0:03.00
34	T	69	68	1	0:02.94
35	T	69	68	1	0:02.94
36	T	58	58	0	0:02.50
37	T	58	58	0	0:02.50
38	T	58	58	0	0:02.51
39	T	58	58	0	0:03.07
40	T	69	68	1	0:02.98
41	T	58	58	0	0:02.48
42	T	58	58	0	0:02.46
43	T	58	58	0	0:02.48
44	T	58	58	0	0:02.50
45	T	58	58	0	0:02.50
46	T	96	93	3	0:04.04
47	T	58	58	0	0:02.48
48	T	69	68	1	0:02.93
49	T	69	68	1	0:03.36
50	T	58	58	0	0:02.50
51	T	58	58	0	0:02.47
52	T	58	58	0	0:02.52
53	T	58	58	0	0:02.49
54	T	58	58	0	0:02.48
55	T	58	58	0	0:02.51
56	T	69	68	1	0:02.98
57	T	58	58	0	0:02.97
58	T	58	58	0	0:02.50
59	T	58	58	0	0:02.47
60	T	58	58	0	0:02.51
61	T	58	58	0	0:02.50
62	T	58	58	0	0:02.50
63	T	58	58	0	0:03.74
64	T	69	68	1	0:02.95
65	T	69	68	1	0:02.99
66	T	69	68	1	0:02.95
67	T	58	58	0	0:02.47
68	T	69	68	1	0:02.93
69	T	58	58	0	0:02.49
70	T	58	58	0	0:02.48
71	T	58	58	0	0:02.49
72	T	69	68	1	0:02.97
73	T	69	68	1	0:02.98
74	T	58	58	0	0:02.50
75	T	58	58	0	0:02.47
76	T	58	58	0	0:02.46

Id	Result	#total	#true	#false	Execution time (min:sec)
77	T	58	58	0	0:02.50
78	T	58	58	0	0:02.52
79	T	58	58	0	0:02.49
80	T	58	58	0	0:02.48
81	T	69	68	1	0:02.99
82	T	69	68	1	0:02.98
83	T	58	58	0	0:02.53
84	T	69	68	1	0:02.99
85	T	58	58	0	0:02.49
86	T	58	58	0	0:02.48
87	T	58	58	0	0:02.72
88	T	69	68	1	0:02.95
89	T	58	58	0	0:02.50
90	T	58	58	0	0:02.46
91	T	96	93	3	0:04.02
92	T	58	58	0	0:02.45
93	T	69	68	1	0:02.93
94	T	69	68	1	0:02.92
95	T	69	68	1	0:03.17
96	T	69	68	1	0:02.94
97	T	69	68	1	0:02.93
98	T	83	81	2	0:03.55
99	T	69	68	1	0:02.95
100	T	69	68	1	0:02.95
101	T	58	58	0	0:02.51
102	T	69	68	1	0:02.95
103	T	83	81	2	0:03.52
104	T	69	68	1	0:02.91
105	T	69	68	1	0:02.99
106	T	58	58	0	0:02.49
107	T	69	68	1	0:02.93
108	T	58	58	0	0:02.45
109	T	83	81	2	0:03.54
110	T	69	68	1	0:02.95
111	T	69	68	1	0:02.90
112	T	69	68	1	0:02.92
113	T	69	68	1	0:02.94
114	T	58	58	0	0:02.50
115	T	69	68	1	0:02.95
116	T	58	58	0	0:03.08
117	T	69	68	1	0:02.92
118	T	69	68	1	0:02.92
119	T	58	58	0	0:02.50
120	T	58	58	0	0:02.46
121	T	58	58	0	0:02.50
122	T	58	58	0	0:02.49
123	T	69	68	1	0:02.94
124	T	58	58	0	0:02.47

Id	Result	#total	#true	#false	Execution time (min:sec)
125	T	58	58	0	0:02.49
126	T	58	58	0	0:02.48
127	T	58	58	0	0:02.49
128	T	58	58	0	0:02.46
129	T	58	58	0	0:02.52
130	T	58	58	0	0:03.83
131	T	58	58	0	0:02.46
132	T	58	58	0	0:02.50
133	T	69	68	1	0:02.95
134	T	58	58	0	0:02.45
135	T	58	58	0	0:02.48
136	T	58	58	0	0:03.98
137	T	58	58	0	0:02.48
138	T	58	58	0	0:02.50
139	T	58	58	0	0:02.49
140	T	58	58	0	0:02.46
141	T	58	58	0	0:02.47
142	T	58	58	0	0:02.47
143	T	58	58	0	0:02.47
144	T	58	58	0	0:02.45
145	T	69	68	1	0:02.93
146	T	58	58	0	0:02.48
147	T	58	58	0	0:02.48
148	T	58	58	0	0:02.51
149	T	69	68	1	0:02.95
150	T	58	58	0	0:02.49
151	T	58	58	0	0:02.50
152	T	58	58	0	0:02.49
153	T	58	58	0	0:02.49
154	T	83	81	2	0:03.55
155	T	69	68	1	0:02.96
156	T	69	68	1	0:04.19
157	T	58	58	0	0:02.48
158	T	58	58	0	0:02.49
159	T	58	58	0	0:02.44
160	T	58	58	0	0:02.48
161	T	83	81	2	0:03.53
162	T	69	68	1	0:02.93
163	T	69	68	1	0:02.95
164	T	58	58	0	0:02.50
165	T	58	58	0	0:02.49
166	T	58	58	0	0:02.47
167	T	58	58	0	0:03.88
168	T	58	58	0	0:02.53
169	T	58	58	0	0:02.48
170	T	58	58	0	0:02.48
171	T	58	58	0	0:02.52
172	T	58	58	0	0:02.48

Id	Result	#total	#true	#false	Execution time (min:sec)
173	T	69	68	1	0:02.92
174	T	58	58	0	0:02.50
175	T	58	58	0	0:02.49
176	T	69	68	1	0:02.94
177	T	58	58	0	0:02.52
178	T	69	68	1	0:02.95
179	T	69	68	1	0:02.96
180	T	58	58	0	0:02.49
181	T	58	58	0	0:02.47
182	T	58	58	0	0:02.45
183	T	69	68	1	0:02.94
184	T	58	58	0	0:02.49
185	T	69	68	1	0:02.96
186	T	58	58	0	0:02.20
187	T	58	58	0	0:02.47
188	T	58	58	0	0:02.48
189	T	58	58	0	0:02.47
190	T	58	58	0	0:02.50
191	T	58	58	0	0:02.45
192	T	58	58	0	0:02.51
193	T	58	58	0	0:02.48
194	T	69	68	1	0:02.94
195	T	69	68	1	0:02.82
196	T	58	58	0	0:02.48
197	T	58	58	0	0:02.50
198	T	58	58	0	0:02.55
199	T	58	58	0	0:02.47
200	T	58	58	0	0:02.46
201	T	58	58	0	0:02.46
202	T	58	58	0	0:02.48
203	T	58	58	0	0:02.51
204	T	69	68	1	0:04.59
205	T	58	58	0	0:02.51
206	T	58	58	0	0:02.48
207	T	69	68	1	0:02.99
208	T	58	58	0	0:02.47
209	T	58	58	0	0:02.49
210	T	58	58	0	0:02.65
211	T	69	68	1	0:02.93
212	T	69	68	1	0:02.96
213	T	58	58	0	0:02.47
214	T	58	58	0	0:02.46
215	T	58	58	0	0:02.48
216	T	58	58	0	0:02.51
217	T	58	58	0	0:02.49
218	T	58	58	0	0:02.46
219	T	83	81	2	0:03.57
220	T	58	58	0	0:02.48

Id	Result	#total	#true	#false	Execution time (min:sec)
221	T	58	58	0	0:02.47
222	T	58	58	0	0:02.48
223	T	69	68	1	0:02.89
224	T	69	68	1	0:02.93
225	T	69	68	1	0:02.94
226	T	69	68	1	0:02.97
227	T	58	58	0	0:02.49
228	T	69	68	1	0:02.92
229	T	58	58	0	0:02.49
230	T	69	68	1	0:02.94
231	T	58	58	0	0:02.50
232	T	58	58	0	0:02.48
233	T	69	68	1	0:02.92
234	T	58	58	0	0:02.47
235	T	69	68	1	0:02.96
236	T	58	58	0	0:02.53
237	T	58	58	0	0:02.87
238	T	58	58	0	0:02.49
239	T	83	81	2	0:03.51
240	T	58	58	0	0:02.46
241	T	58	58	0	0:02.47
242	T	58	58	0	0:02.49
243	T	58	58	0	0:03.83
244	T	58	58	0	0:02.51
245	T	83	81	2	0:03.56
246	T	58	58	0	0:02.44
247	T	58	58	0	0:02.47
248	T	58	58	0	0:02.48
249	T	58	58	0	0:02.52
250	T	58	58	0	0:02.49
251	T	69	68	1	0:02.95
252	T	58	58	0	0:02.45
253	T	69	68	1	0:02.96
254	T	58	58	0	0:02.46
255	T	58	58	0	0:02.49
256	T	58	58	0	0:02.49
257	T	58	58	0	0:02.46
258	T	58	58	0	0:02.48
259	T	69	68	1	0:02.93
260	T	69	68	1	0:02.98
261	T	83	81	2	0:03.52
262	T	58	58	0	0:02.58
263	T	58	58	0	0:02.47
264	T	58	58	0	0:02.46
265	T	58	58	0	0:02.46
266	T	58	58	0	0:02.50
267	T	58	58	0	0:02.50
268	T	58	58	0	0:02.51

Id	Result	#total	#true	#false	Execution time (min:sec)
269	T	58	58	0	0:02.46
270	T	58	58	0	0:02.47
271	T	58	58	0	0:03.04
272	T	58	58	0	0:02.51
273	T	58	58	0	0:02.47
274	T	58	58	0	0:02.48
275	T	69	68	1	0:02.97
276	T	69	68	1	0:02.92
277	T	83	81	2	0:03.52
278	T	58	58	0	0:02.48
279	T	58	58	0	0:02.45
280	T	69	68	1	0:02.98
281	T	58	58	0	0:02.52
282	T	58	58	0	0:02.46
283	T	58	58	0	0:02.50
284	T	58	58	0	0:02.48
285	T	69	68	1	0:02.94
286	T	83	81	2	0:03.60
287	T	58	58	0	0:02.47
288	T	58	58	0	0:02.72
289	T	58	58	0	0:03.15
290	T	96	93	3	0:04.14
291	T	58	58	0	0:02.52
292	T	58	58	0	0:02.46
293	T	58	58	0	0:02.47
294	T	69	68	1	0:02.93
295	T	83	81	2	0:03.53
296	T	96	93	3	0:04.10
297	T	58	58	0	0:02.47
298	T	58	58	0	0:02.51
299	T	58	58	0	0:02.48
300	T	58	58	0	0:02.48
301	T	58	58	0	0:02.45
302	T	58	58	0	0:02.54
303	T	58	58	0	0:02.53
304	T	58	58	0	0:04.01
305	T	69	68	1	0:03.00
306	T	110	106	4	0:04.74
307	T	69	68	1	0:02.95
308	T	58	58	0	0:02.47
309	T	58	58	0	0:02.48
310	T	58	58	0	0:02.46
311	T	58	58	0	0:02.47
312	T	69	68	1	0:02.94
313	T	58	58	0	0:02.47
314	T	58	58	0	0:02.50
315	T	58	58	0	0:02.47
316	T	58	58	0	0:02.48

Id	Result	#total	#true	#false	Execution time (min:sec)
317	T	58	58	0	0:02.47
318	T	58	58	0	0:02.47
319	T	58	58	0	0:02.46
320	T	58	58	0	0:02.45
321	T	83	81	2	0:03.53
322	T	69	68	1	0:02.96
323	T	69	68	1	0:02.96
324	T	69	68	1	0:02.96
325	T	83	81	2	0:03.54
326	T	69	68	1	0:02.91
327	T	58	58	0	0:02.50
328	T	58	58	0	0:02.47
329	T	58	58	0	0:03.64
330	T	58	58	0	0:02.49
331	T	69	68	1	0:02.98
332	T	58	58	0	0:02.46
333	T	58	58	0	0:02.51
334	T	58	58	0	0:02.49
335	T	58	58	0	0:02.51
336	T	69	68	1	0:02.96
337	T	58	58	0	0:02.51
338	T	58	58	0	0:02.50
339	T	58	58	0	0:02.46
340	T	58	58	0	0:02.49
341	T	58	58	0	0:02.54
342	T	58	58	0	0:02.45
343	T	69	68	1	0:03.47
344	T	69	68	1	0:02.99
345	T	58	58	0	0:02.48
346	T	58	58	0	0:02.50
347	T	58	58	0	0:02.46
348	T	96	93	3	0:04.12
349	T	58	58	0	0:02.49
350	T	69	68	1	0:02.96
351	T	69	68	1	0:02.93
352	T	58	58	0	0:02.46
353	T	83	81	2	0:04.99
354	T	69	68	1	0:02.92
355	T	83	81	2	0:03.53
356	T	58	58	0	0:02.47
357	T	58	58	0	0:02.52
358	T	69	68	1	0:02.94
359	T	69	68	1	0:02.95
360	T	58	58	0	0:02.45
361	T	69	68	1	0:02.95
362	T	58	58	0	0:02.46
363	T	58	58	0	0:02.48
364	T	58	58	0	0:02.47

Id	Result	#total	#true	#false	Execution time (min:sec)
365	T	58	58	0	0:02.47
366	T	69	68	1	0:02.92
367	T	69	68	1	0:02.92
368	T	58	58	0	0:03.84
369	T	58	58	0	0:02.50
370	T	69	68	1	0:02.94
371	T	83	81	2	0:03.52
372	T	69	68	1	0:02.95
373	T	58	58	0	0:02.47
374	T	96	93	3	0:04.05
375	T	69	68	1	0:02.91
376	T	69	68	1	0:02.93
377	T	69	68	1	0:02.93
378	T	58	58	0	0:02.49
379	T	58	58	0	0:02.52
380	T	58	58	0	0:02.46
381	T	69	68	1	0:02.98
382	T	58	58	0	0:02.51
383	T	69	68	1	0:02.91
384	T	58	58	0	0:02.49
385	T	58	58	0	0:02.46
386	T	58	58	0	0:02.50
387	T	69	68	1	0:02.92
388	T	58	58	0	0:02.46
389	T	58	58	0	0:02.47
390	T	69	68	1	0:02.95
391	T	83	81	2	0:03.51
392	T	58	58	0	0:02.50
393	T	83	81	2	0:03.51
394	T	58	58	0	0:02.48
395	T	83	81	2	0:03.52
396	T	69	68	1	0:02.97
397	T	83	81	2	0:04.57
398	T	58	58	0	0:03.39
399	T	69	68	1	0:02.92
400	T	69	68	1	0:02.94
401	T	69	68	1	0:02.93
402	T	58	58	0	0:02.45
403	T	69	68	1	0:04.81
404	T	69	68	1	0:02.91
405	T	69	68	1	0:02.92
406	T	58	58	0	0:02.47
407	T	58	58	0	0:02.46
408	T	58	58	0	0:02.49
409	T	69	68	1	0:02.95
410	T	58	58	0	0:02.50
411	T	69	68	1	0:02.91
412	T	58	58	0	0:02.46

Id	Result	#total	#true	#false	Execution time (min:sec)
413	T	69	68	1	0:02.97
414	T	58	58	0	0:02.49
415	T	58	58	0	0:02.49
416	T	58	58	0	0:04.08
417	T	69	68	1	0:02.94
418	T	69	68	1	0:02.93
419	T	58	58	0	0:02.49
420	T	58	58	0	0:02.46
421	T	69	68	1	0:02.97
422	T	58	58	0	0:02.48
423	T	58	58	0	0:02.45
424	T	83	81	2	0:03.52
425	T	58	58	0	0:02.48
426	T	58	58	0	0:02.48
427	T	58	58	0	0:04.04
428	T	58	58	0	0:02.49
429	T	69	68	1	0:02.97
430	T	58	58	0	0:02.52
431	T	58	58	0	0:02.51
432	T	69	68	1	0:02.96
433	T	58	58	0	0:02.49
434	T	58	58	0	0:02.49
435	T	58	58	0	0:02.68
436	T	58	58	0	0:02.48
437	T	58	58	0	0:02.48
438	T	83	81	2	0:03.51
439	T	58	58	0	0:02.50
440	T	58	58	0	0:02.48
441	T	58	58	0	0:02.47
442	T	69	68	1	0:02.97
443	T	58	58	0	0:02.47
444	T	58	58	0	0:02.48
445	T	69	68	1	0:02.97
446	T	58	58	0	0:02.51
447	T	58	58	0	0:02.48
448	T	69	68	1	0:02.92
449	T	58	58	0	0:02.47
450	T	58	58	0	0:02.43
451	T	58	58	0	0:02.48
452	T	96	93	3	0:04.10
453	T	69	68	1	0:02.89
454	T	58	58	0	0:02.50
455	T	58	58	0	0:02.48
456	T	69	68	1	0:02.94
457	T	58	58	0	0:02.49
458	T	69	68	1	0:02.96
459	T	58	58	0	0:02.48
460	T	69	68	1	0:02.94

Id	Result	#total	#true	#false	Execution time (min:sec)
461	T	58	58	0	0:02.48
462	T	58	58	0	0:02.48
463	T	69	68	1	0:02.96
464	T	69	68	1	0:02.97
465	T	58	58	0	0:02.51
466	T	58	58	0	0:02.50
467	T	58	58	0	0:02.48
468	T	69	68	1	0:02.93
469	T	58	58	0	0:02.46
470	T	58	58	0	0:02.48
471	T	83	81	2	0:03.52
472	T	58	58	0	0:02.52
473	T	58	58	0	0:02.49
474	T	58	58	0	0:02.44
475	T	58	58	0	0:02.50
476	T	58	58	0	0:02.48
477	T	58	58	0	0:02.52
478	T	58	58	0	0:02.67
479	T	58	58	0	0:03.54
480	T	83	81	2	0:03.48
481	T	58	58	0	0:02.49
482	T	58	58	0	0:02.49
483	T	58	58	0	0:02.48
484	T	58	58	0	0:03.06
485	T	58	58	0	0:02.50
486	T	96	93	3	0:02.55
487	T	58	58	0	0:02.47
488	T	58	58	0	0:02.51
489	T	58	58	0	0:02.50
490	T	69	68	1	0:02.92
491	T	58	58	0	0:02.52
492	T	69	68	1	0:02.91
493	T	58	58	0	0:02.54
494	T	58	58	0	0:02.49
495	T	58	58	0	0:02.46
496	T	96	93	3	0:04.06
497	T	58	58	0	0:02.44
498	T	58	58	0	0:02.51
499	T	58	58	0	0:02.48
500	T	58	58	0	0:02.85

Table 4: Model checking results corresponding to logic property 4

Id	Result	#total	#true	#false	Execution time (min:sec)
1	T	3	2	1	0:00.46

Id	Result	#total	#true	#false	Execution time (min:sec)
2	T	3	2	1	0:00.19
3	F	8	0	8	0:00.64
4	F	8	0	8	0:00.42
5	T	2	1	1	0:00.20
6	T	2	1	1	0:00.15
7	T	2	1	1	0:00.15
8	T	2	1	1	0:00.16
9	T	2	1	1	0:00.15
10	T	2	1	1	0:00.15
11	T	2	1	1	0:00.18
12	T	2	1	1	0:00.15
13	T	2	1	1	0:00.14
14	T	2	1	1	0:00.14
15	T	2	1	1	0:00.15
16	T	2	1	1	0:00.14
17	T	2	1	1	0:00.19
18	T	2	1	1	0:00.16
19	T	2	1	1	0:00.15
20	T	2	1	1	0:00.15
21	T	2	1	1	0:00.15
22	T	4	1	3	0:00.29
23	T	4	1	3	0:00.22
24	T	4	1	3	0:00.24
25	T	4	1	3	0:00.23
26	T	3	1	2	0:00.25
27	T	3	1	2	0:00.20
28	T	3	1	2	0:00.19
29	T	3	1	2	0:00.18
30	T	3	1	2	0:00.19
31	T	3	2	1	0:00.23
32	T	3	2	1	0:00.19
33	T	3	2	1	0:00.19
34	T	3	2	1	0:00.18
35	T	3	2	1	0:00.18
36	T	2	1	1	0:00.27
37	T	2	1	1	0:00.15
38	T	2	1	1	0:00.16
39	T	2	1	1	0:00.15
40	T	2	1	1	0:00.15
41	T	3	2	1	0:00.29
42	T	3	2	1	0:00.20
43	T	3	2	1	0:00.19
44	T	3	2	1	0:00.21
45	T	2	1	1	0:00.19
46	T	2	1	1	0:00.14
47	T	2	1	1	0:00.14
48	T	2	1	1	0:00.14
49	T	2	1	1	0:00.13

Id	Result	#total	#true	#false	Execution time (min:sec)
50	T	2	1	1	0:00.15
51	T	3	1	2	0:00.23
52	T	3	1	2	0:00.19
53	T	3	1	2	0:00.18
54	T	3	1	2	0:00.20
55	T	3	1	2	0:00.18
56	T	3	2	1	0:00.22
57	T	3	2	1	0:00.18
58	T	3	2	1	0:00.20
59	T	3	2	1	0:00.18
60	T	3	2	1	0:00.18
61	T	5	5	0	0:00.36
62	T	5	5	0	0:00.28
63	T	5	5	0	0:00.28
64	T	2	1	1	0:00.20
65	T	2	1	1	0:00.13
66	T	2	1	1	0:00.14
67	T	2	1	1	0:00.13
68	T	2	1	1	0:00.14
69	T	2	1	1	0:00.15
70	T	5	1	4	0:00.35
71	T	5	1	4	0:00.29
72	T	5	1	4	0:00.29
73	T	4	1	3	0:00.28
74	T	4	1	3	0:00.24
75	T	4	1	3	0:00.23
76	T	4	1	3	0:00.24
77	T	5	5	0	0:00.40
78	T	5	5	0	0:00.27
79	T	5	5	0	0:00.27
80	T	3	2	1	0:00.26
81	T	3	2	1	0:00.21
82	T	3	2	1	0:00.20
83	T	3	2	1	0:00.18
84	T	3	2	1	0:00.21
85	T	2	1	1	0:00.18
86	T	2	1	1	0:00.16
87	T	2	1	1	0:00.15
88	T	2	1	1	0:00.16
89	T	2	1	1	0:00.15
90	T	3	1	2	0:00.23
91	T	3	1	2	0:00.19
92	T	3	1	2	0:00.19
93	T	3	1	2	0:00.19
94	T	3	1	2	0:00.19
95	T	2	1	1	0:00.18
96	T	2	1	1	0:00.14
97	T	2	1	1	0:00.13

Id	Result	#total	#true	#false	Execution time (min:sec)
98	T	2	1	1	0:00.15
99	T	2	1	1	0:00.14
100	T	2	1	1	0:00.15
101	T	3	2	1	0:00.27
102	T	3	2	1	0:00.19
103	T	3	2	1	0:00.19
104	T	3	2	1	0:00.19
105	T	2	1	1	0:00.17
106	T	2	1	1	0:00.15
107	T	2	1	1	0:00.15
108	T	2	1	1	0:00.15
109	T	2	1	1	0:00.15
110	T	2	1	1	0:00.16
111	T	4	1	3	0:00.34
112	T	4	1	3	0:00.22
113	T	4	1	3	0:00.24
114	T	4	1	3	0:00.24
115	T	3	1	2	0:00.23
116	T	3	1	2	0:00.20
117	T	3	1	2	0:00.20
118	T	3	1	2	0:00.19
119	T	2	1	1	0:00.18
120	T	2	1	1	0:00.16
121	T	2	1	1	0:00.16
122	T	2	1	1	0:00.14
123	T	2	1	1	0:00.15
124	T	2	1	1	0:00.15
125	T	2	1	1	0:00.23
126	T	2	1	1	0:00.15
127	T	2	1	1	0:00.16
128	T	2	1	1	0:00.15
129	T	2	1	1	0:00.14
130	T	2	1	1	0:00.15
131	T	3	1	2	0:00.23
132	T	3	1	2	0:00.19
133	T	3	1	2	0:00.18
134	T	3	1	2	0:00.18
135	T	3	1	2	0:00.19
136	T	2	1	1	0:00.17
137	T	2	1	1	0:00.16
138	T	2	1	1	0:00.15
139	T	2	1	1	0:00.15
140	T	2	1	1	0:00.15
141	T	2	1	1	0:00.15
142	T	2	1	1	0:00.21
143	T	2	1	1	0:00.15
144	T	2	1	1	0:00.14
145	T	2	1	1	0:00.15

Id	Result	#total	#true	#false	Execution time (min:sec)
146	T	2	1	1	0:00.14
147	T	4	3	1	0:00.31
148	T	4	3	1	0:00.24
149	T	4	3	1	0:00.23
150	T	4	3	1	0:00.25
151	T	5	4	1	0:00.33
152	T	5	4	1	0:00.27
153	T	5	4	1	0:00.26
154	T	4	3	1	0:00.27
155	T	4	3	1	0:00.23
156	T	4	3	1	0:00.23
157	T	4	3	1	0:00.24
158	T	3	1	2	0:00.22
159	T	3	1	2	0:00.21
160	T	3	1	2	0:00.19
161	T	3	1	2	0:00.18
162	T	3	1	2	0:00.19
163	T	2	1	1	0:00.18
164	T	2	1	1	0:00.16
165	T	2	1	1	0:00.15
166	T	2	1	1	0:00.15
167	T	2	1	1	0:00.14
168	T	2	1	1	0:00.15
169	T	3	2	1	0:00.24
170	T	3	2	1	0:00.19
171	T	3	2	1	0:00.21
172	T	3	2	1	0:00.18
173	T	3	2	1	0:00.19
174	T	2	1	1	0:00.19
175	T	2	1	1	0:00.14
176	T	2	1	1	0:00.15
177	T	2	1	1	0:00.14
178	T	2	1	1	0:00.15
179	T	3	2	1	0:00.23
180	T	3	2	1	0:00.19
181	T	3	2	1	0:00.19
182	T	3	2	1	0:00.20
183	T	3	2	1	0:00.18
184	T	5	4	1	0:00.34
185	T	5	4	1	0:00.27
186	T	5	4	1	0:00.27
187	T	2	1	1	0:00.18
188	T	2	1	1	0:00.15
189	T	2	1	1	0:00.14
190	T	2	1	1	0:00.15
191	T	2	1	1	0:00.16
192	T	2	1	1	0:00.14
193	T	2	1	1	0:00.20

Id	Result	#total	#true	#false	Execution time (min:sec)
194	T	2	1	1	0:00.16
195	T	2	1	1	0:00.15
196	T	2	1	1	0:00.15
197	T	2	1	1	0:00.16
198	T	2	1	1	0:00.15
199	T	3	2	1	0:00.21
200	T	3	2	1	0:00.19
201	T	3	2	1	0:00.18
202	T	3	2	1	0:00.20
203	T	3	2	1	0:00.19
204	T	2	1	1	0:00.17
205	T	2	1	1	0:00.14
206	T	2	1	1	0:00.16
207	T	2	1	1	0:00.16
208	T	2	1	1	0:00.15
209	T	2	1	1	0:00.15
210	T	2	1	1	0:00.17
211	T	2	1	1	0:00.15
212	T	2	1	1	0:00.14
213	T	2	1	1	0:00.14
214	T	2	1	1	0:00.15
215	T	2	1	1	0:00.13
216	T	3	2	1	0:00.26
217	T	3	2	1	0:00.18
218	T	3	2	1	0:00.20
219	T	3	2	1	0:00.20
220	T	288	168	120	0:18.72
221	T	2	1	1	0:00.16
222	T	2	1	1	0:00.15
223	T	5	4	1	0:00.34
224	T	5	4	1	0:00.27
225	T	5	4	1	0:00.27
226	T	3	2	1	0:00.22
227	T	3	2	1	0:00.19
228	T	3	2	1	0:00.19
229	T	3	2	1	0:00.19
230	T	3	2	1	0:00.21
231	T	5	1	4	0:00.30
232	T	5	1	4	0:00.30
233	T	5	1	4	0:00.29
234	T	2	1	1	0:00.16
235	T	2	1	1	0:00.14
236	T	2	1	1	0:00.14
237	T	2	1	1	0:00.14
238	T	2	1	1	0:00.14
239	T	2	1	1	0:00.14
240	T	2	1	1	0:00.14
241	T	3	1	2	0:00.21

Id	Result	#total	#true	#false	Execution time (min:sec)
242	T	3	1	2	0:00.20
243	T	3	1	2	0:00.21
244	T	3	1	2	0:00.20
245	T	3	1	2	0:00.20
246	T	2	1	1	0:00.19
247	T	2	1	1	0:00.15
248	T	2	1	1	0:00.15
249	T	2	1	1	0:00.14
250	T	2	1	1	0:00.16
251	T	2	1	1	0:00.17
252	T	2	1	1	0:00.15
253	T	2	1	1	0:00.15
254	T	2	1	1	0:00.15
255	T	2	1	1	0:00.14
256	T	2	1	1	0:00.15
257	T	2	1	1	0:00.15
258	T	2	1	1	0:00.15
259	T	2	1	1	0:00.15
260	T	2	1	1	0:00.14
261	T	2	1	1	0:00.15
262	T	2	1	1	0:00.14
263	T	2	1	1	0:00.16
264	T	2	1	1	0:00.14
265	T	2	1	1	0:00.15
266	T	2	1	1	0:00.15
267	T	2	1	1	0:00.15
268	T	2	1	1	0:00.15
269	T	5	5	0	0:00.31
270	T	5	5	0	0:00.28
271	T	5	5	0	0:00.27
272	T	5	5	0	0:00.28
273	T	2	1	1	0:00.16
274	T	2	1	1	0:00.16
275	T	2	1	1	0:00.16
276	T	2	1	1	0:00.14
277	T	2	1	1	0:00.16
278	T	2	1	1	0:00.18
279	T	2	1	1	0:00.14
280	T	2	1	1	0:00.15
281	T	2	1	1	0:00.16
282	T	2	1	1	0:00.14
283	T	2	1	1	0:00.15
284	T	3	2	1	0:00.22
285	T	3	2	1	0:00.18
286	T	3	2	1	0:00.19
287	T	3	2	1	0:00.18
288	T	3	2	1	0:00.19
289	T	2	1	1	0:00.15

**Id**	**Result**	**#total**	**#true**	**#false**	**Execution time (min:sec)**
290	T	2	1	1	0:00.16
291	T	2	1	1	0:00.15
292	T	2	1	1	0:00.14
293	T	2	1	1	0:00.15
294	T	2	1	1	0:00.15
295	T	5	5	0	0:00.32
296	T	5	5	0	0:00.28
297	T	5	5	0	0:00.26
298	T	3	2	1	0:00.22
299	T	3	2	1	0:00.19
300	T	3	2	1	0:00.18
301	T	3	2	1	0:00.19
302	T	3	2	1	0:00.19
303	T	2	1	1	0:00.17
304	T	2	1	1	0:00.14
305	T	2	1	1	0:00.13
306	T	2	1	1	0:00.14
307	T	2	1	1	0:00.14
308	T	2	1	1	0:00.14
309	T	2	1	1	0:00.16
310	T	2	1	1	0:00.14
311	T	2	1	1	0:00.14
312	T	2	1	1	0:00.14
313	T	2	1	1	0:00.14
314	T	2	1	1	0:00.14
315	T	2	1	1	0:00.14
316	T	2	1	1	0:00.15
317	T	2	1	1	0:00.15
318	T	2	1	1	0:00.14
319	T	2	1	1	0:00.13
320	T	2	1	1	0:00.14
321	T	2	1	1	0:00.15
322	T	5	5	0	0:00.32
323	T	5	5	0	0:00.29
324	T	5	5	0	0:00.30
325	T	2	1	1	0:00.15
326	T	2	1	1	0:00.15
327	T	2	1	1	0:00.15
328	T	2	1	1	0:00.13
329	T	2	1	1	0:00.14
330	T	2	1	1	0:00.14
331	T	2	1	1	0:00.14
332	T	3	2	1	0:00.20
333	T	3	2	1	0:00.19
334	T	3	2	1	0:00.18
335	T	3	2	1	0:00.18
336	T	3	2	1	0:00.19
337	T	2	1	1	0:00.17

Id	Result	#total	#true	#false	Execution time (min:sec)
338	T	2	1	1	0:00.14
339	T	2	1	1	0:00.15
340	T	2	1	1	0:00.14
341	T	2	1	1	0:00.15
342	T	2	1	1	0:00.16
343	T	2	1	1	0:00.17
344	T	2	1	1	0:00.14
345	T	2	1	1	0:00.14
346	T	2	1	1	0:00.13
347	T	2	1	1	0:00.14
348	T	2	1	1	0:00.14
349	T	5	5	0	0:00.31
350	T	5	5	0	0:00.28
351	T	5	5	0	0:00.27
352	T	2	1	1	0:00.16
353	T	2	1	1	0:00.14
354	T	2	1	1	0:00.14
355	T	2	1	1	0:00.14
356	T	2	1	1	0:00.14
357	T	2	1	1	0:00.14
358	T	2	1	1	0:00.14
359	T	2	1	1	0:00.16
360	T	2	1	1	0:00.16
361	T	2	1	1	0:00.15
362	T	2	1	1	0:00.15
363	T	2	1	1	0:00.15
364	T	2	1	1	0:00.15
365	T	2	1	1	0:00.17
366	T	2	1	1	0:00.15
367	T	2	1	1	0:00.14
368	T	2	1	1	0:00.16
369	T	2	1	1	0:00.14
370	T	2	1	1	0:00.15
371	T	5	4	1	0:00.32
372	T	5	4	1	0:00.29
373	T	5	4	1	0:00.28
374	T	2	1	1	0:00.19
375	T	2	1	1	0:00.15
376	T	2	1	1	0:00.14
377	T	2	1	1	0:00.14
378	T	2	1	1	0:00.14
379	T	2	1	1	0:00.14
380	T	2	1	1	0:00.18
381	T	2	1	1	0:00.15
382	T	2	1	1	0:00.15
383	T	2	1	1	0:00.14
384	T	2	1	1	0:00.15
385	T	2	1	1	0:00.15

Id	Result	#total	#true	#false	Execution time (min:sec)
386	T	2	1	1	0:00.19
387	T	2	1	1	0:00.15
388	T	2	1	1	0:00.15
389	T	2	1	1	0:00.15
390	T	2	1	1	0:00.15
391	T	2	1	1	0:00.15
392	T	2	1	1	0:00.14
393	T	2	1	1	0:00.14
394	T	2	1	1	0:00.15
395	T	2	1	1	0:00.15
396	T	2	1	1	0:00.14
397	T	2	1	1	0:00.14
398	T	4	3	1	0:00.27
399	T	4	3	1	0:00.23
400	T	4	3	1	0:00.24
401	T	4	3	1	0:00.24
402	T	4	1	3	0:00.27
403	T	4	1	3	0:00.24
404	T	4	1	3	0:00.24
405	T	4	1	3	0:00.24
406	T	2	1	1	0:00.16
407	T	2	1	1	0:00.14
408	T	2	1	1	0:00.14
409	T	2	1	1	0:00.15
410	T	2	1	1	0:00.14
411	T	2	1	1	0:00.14
412	T	3	1	2	0:00.21
413	T	3	1	2	0:00.19
414	T	3	1	2	0:00.19
415	T	3	1	2	0:00.20
416	T	3	1	2	0:00.19
417	T	2	1	1	0:00.15
418	T	2	1	1	0:00.16
419	T	2	1	1	0:00.16
420	T	2	1	1	0:00.15
421	T	2	1	1	0:00.15
422	T	2	1	1	0:00.14
423	T	3	2	1	0:00.21
424	T	3	2	1	0:00.19
425	T	3	2	1	0:00.19
426	T	3	2	1	0:00.19
427	T	3	2	1	0:00.19
428	T	2	1	1	0:00.18
429	T	2	1	1	0:00.14
430	T	2	1	1	0:00.14
431	T	2	1	1	0:00.15
432	T	2	1	1	0:00.15
433	T	3	2	1	0:00.21

Id	Result	#total	#true	#false	Execution time (min:sec)
434	T	3	2	1	0:00.19
435	T	3	2	1	0:00.19
436	T	3	2	1	0:00.19
437	T	3	2	1	0:00.19
438	T	5	4	1	0:00.32
439	T	5	4	1	0:00.27
440	T	5	4	1	0:00.28
441	T	5	4	1	0:00.29
442	T	5	1	4	0:00.31
443	T	5	1	4	0:00.30
444	T	5	1	4	0:00.28
445	T	4	3	1	0:00.25
446	T	4	3	1	0:00.23
447	T	4	3	1	0:00.22
448	T	4	3	1	0:00.23
449	T	5	5	0	0:00.27
450	T	5	5	0	0:00.29
451	T	5	5	0	0:00.27
452	T	2	1	1	0:00.19
453	T	2	1	1	0:00.17
454	T	2	1	1	0:00.16
455	T	2	1	1	0:00.14
456	T	2	1	1	0:00.16
457	T	2	1	1	0:00.14
458	T	2	1	1	0:00.15
459	T	2	1	1	0:00.15
460	T	2	1	1	0:00.14
461	T	2	1	1	0:00.16
462	T	2	1	1	0:00.14
463	T	2	1	1	0:00.16
464	T	4	1	3	0:00.29
465	T	4	1	3	0:00.23
466	T	4	1	3	0:00.25
467	T	4	1	3	0:00.25
468	T	3	1	2	0:00.21
469	T	3	1	2	0:00.18
470	T	3	1	2	0:00.19
471	T	3	1	2	0:00.19
472	T	3	1	2	0:00.23
473	T	3	1	2	0:00.19
474	T	3	1	2	0:00.20
475	T	3	1	2	0:00.22
476	T	3	1	2	0:00.20
477	T	3	2	1	0:00.23
478	T	3	2	1	0:00.18
479	T	3	2	1	0:00.19
480	T	3	2	1	0:00.19
481	T	3	2	1	0:00.20

Id	Result	#total	#true	#false	Execution time (min:sec)
482	T	5	4	1	0:00.31
483	T	5	4	1	0:00.27
484	T	5	4	1	0:00.28
485	T	2	1	1	0:00.16
486	T	2	1	1	0:00.15
487	T	2	1	1	0:00.15
488	T	2	1	1	0:00.16
489	T	2	1	1	0:00.15
490	T	2	1	1	0:00.15
491	T	2	1	1	0:00.14
492	T	2	1	1	0:00.15
493	T	2	1	1	0:00.14
494	T	2	1	1	0:00.14
495	T	2	1	1	0:00.15
496	T	2	1	1	0:00.15
497	T	2	1	1	0:00.17
498	T	2	1	1	0:00.15
499	T	2	1	1	0:00.15
500	T	2	1	1	0:00.14

Table 5: Model checking results corresponding to logic property 5

Id	Result	#total	#true	#false	Execution time (min:sec)
1	T	8	5	3	0:00.52
2	T	6	4	2	0:00.49
3	T	6	4	2	0:00.46
4	T	6	4	2	0:00.49
5	T	6	4	2	0:00.44
6	T	19	9	10	0:01.35
7	T	6	4	2	0:00.39
8	T	6	4	2	0:00.34
9	T	26	12	14	0:01.82
10	F	14	3	11	0:01.07
11	F	14	3	11	0:01.15
12	F	66	29	37	0:04.22
13	T	6	5	1	0:00.38
14	T	16	8	8	0:01.16
15	T	5	4	1	0:00.28
16	T	5	4	1	0:00.28
17	T	5	4	1	0:00.27
18	T	19	9	10	0:01.60
19	F	20	6	14	0:01.68
20	F	46	19	27	0:02.93
21	T	16	8	8	0:00.89
22	T	6	4	2	0:00.36

Id	Result	#total	#true	#false	Execution time (min:sec)
23	T	6	4	2	0:00.32
24	T	6	4	2	0:00.32
25	F	8	0	8	0:00.59
26	F	8	0	8	0:00.55
27	T	6	4	2	0:00.40
28	T	6	4	2	0:00.37
29	F	12	2	10	0:00.85
30	T	23	11	12	0:01.37
31	F	16	4	12	0:01.13
32	F	12	2	10	0:00.82
33	F	16	4	12	0:01.00
34	T	16	8	8	0:00.99
35	T	6	4	2	0:00.39
36	T	6	4	2	0:00.36
37	T	11	6	5	0:00.96
38	T	6	4	2	0:00.36
39	T	6	4	2	0:00.34
40	T	6	4	2	0:00.34
41	T	50	21	29	0:03.26
42	F	14	3	11	0:01.08
43	T	18	9	9	0:01.14
44	T	8	5	3	0:00.45
45	T	6	4	2	0:00.36
46	T	6	4	2	0:00.37
47	F	22	7	15	0:01.54
48	T	15	8	7	0:01.13
49	T	6	4	2	0:00.35
50	F	8	0	8	0:00.62
51	F	8	0	8	0:00.59
52	T	11	6	5	0:00.77
53	T	6	4	2	0:00.36
54	T	6	4	2	0:00.35
55	T	6	4	2	0:00.35
56	T	5	4	1	0:00.29
57	T	5	4	1	0:00.28
58	T	5	4	1	0:00.28
59	T	16	8	8	0:01.13
60	T	21	10	11	0:01.36
61	T	23	11	12	0:01.65
62	F	14	3	11	0:00.98
63	T	15	8	7	0:00.91
64	F	25	8	17	0:01.83
65	F	117	57	60	0:06.85
66	T	6	4	2	0:00.33
67	T	10	6	4	0:00.65
68	T	10	6	4	0:00.58
69	F	38	13	25	0:02.55
70	T	11	6	5	0:01.19

Id	Result	#total	#true	#false	Execution time (min:sec)
71	F	30	11	19	0:01.75
72	T	6	4	2	0:00.34
73	T	6	4	2	0:00.34
74	T	6	4	2	0:00.33
75	T	6	4	2	0:00.42
76	T	6	4	2	0:00.39
77	T	6	4	2	0:00.39
78	T	6	4	2	0:00.38
79	T	6	4	2	0:00.38
80	T	13	7	6	0:00.68
81	T	13	7	6	0:00.68
82	T	50	21	29	0:03.27
83	T	65	27	38	0:03.76
84	T	13	7	6	0:00.83
85	T	18	9	9	0:01.14
86	T	8	5	3	0:00.41
87	T	10	6	4	0:00.83
88	T	10	6	4	0:00.84
89	F	18	5	13	0:01.15
90	T	41	18	23	0:02.65
91	F	12	2	10	0:00.67
92	T	11	6	5	0:00.63
93	T	11	6	5	0:00.60
94	T	5	4	1	0:00.31
95	T	5	4	1	0:00.32
96	F	14	3	11	0:00.84
97	T	6	4	2	0:00.34
98	T	6	4	2	0:00.35
99	T	6	4	2	0:00.33
100	F	8	0	8	0:00.51
101	F	8	0	8	0:00.53
102	T	5	4	1	0:00.32
103	T	5	4	1	0:00.31
104	T	5	4	1	0:00.34
105	T	5	4	1	0:00.32
106	T	5	4	1	0:00.28
107	T	5	4	1	0:00.28
108	T	8	5	3	0:00.50
109	T	8	5	3	0:00.48
110	T	6	4	2	0:00.40
111	T	6	4	2	0:00.39
112	T	6	4	2	0:00.37
113	T	5	4	1	0:00.29
114	T	5	4	1	0:00.29
115	T	5	4	1	0:00.31
116	F	18	5	13	0:01.35
117	F	18	5	13	0:01.31
118	T	16	8	8	0:00.86

Id	Result	#total	#true	#false	Execution time (min:sec)
119	T	19	9	10	0:01.09
120	F	16	4	12	0:01.32
121	T	8	5	3	0:00.42
122	T	8	5	3	0:00.43
123	T	21	10	11	0:01.57
124	F	22	7	15	0:01.45
125	T	5	4	1	0:00.30
126	T	5	4	1	0:00.28
127	T	5	4	1	0:00.27
128	F	33	11	22	0:02.91
129	F	28	9	19	0:02.22
130	F	23	7	16	0:03.22
131	F	28	9	19	0:03.01
132	T	8	5	3	0:00.59
133	T	5	4	1	0:00.28
134	T	5	4	1	0:00.29
135	T	5	4	1	0:00.30
136	T	5	4	1	0:00.30
137	T	6	4	2	0:00.43
138	T	6	4	2	0:00.40
139	T	6	4	2	0:00.86
140	F	54	19	35	0:03.10
141	T	11	6	5	0:00.64
142	T	11	6	5	0:00.60
143	F	20	6	14	0:01.14
144	F	12	2	10	0:00.71
145	T	6	4	2	0:00.45
146	T	6	4	2	0:00.41
147	T	10	6	4	0:00.56
148	T	10	6	4	0:00.53
149	T	18	9	9	0:01.29
150	T	11	6	5	0:00.65
151	T	5	4	1	0:00.30
152	T	5	4	1	0:00.29
153	T	5	4	1	0:00.29
154	T	11	6	5	0:00.68
155	T	11	6	5	0:00.67
156	T	42	18	24	0:02.85
157	T	6	4	2	0:00.43
158	T	6	4	2	0:00.41
159	F	28	9	19	0:01.54
160	T	21	10	11	0:01.57
161	T	34	15	19	0:02.51
162	T	15	8	7	0:00.91
163	T	13	7	6	0:00.86
164	T	6	4	2	0:00.34
165	F	33	11	22	0:02.09
166	T	19	9	10	0:01.40

Id	Result	#total	#true	#false	Execution time (min:sec)
167	F	14	3	11	0:00.90
168	T	16	8	8	0:00.96
169	T	10	6	4	0:00.67
170	T	8	5	3	0:00.48
171	T	8	5	3	0:00.47
172	F	60	26	34	0:03.64
173	T	5	4	1	0:00.28
174	F	18	5	13	0:01.57
175	F	26	9	17	0:01.69
176	T	18	9	9	0:01.01
177	T	10	6	4	0:00.54
178	T	11	6	5	0:00.67
179	T	11	6	5	0:00.67
180	T	23	11	12	0:01.49
181	F	28	9	19	0:01.63
182	T	13	7	6	0:00.79
183	F	20	6	14	0:01.20
184	F	16	4	12	0:00.92
185	T	5	4	1	0:00.29
186	T	5	4	1	0:00.29
187	T	5	4	1	0:00.31
188	T	5	4	1	0:00.30
189	T	5	4	1	0:00.31
190	F	34	13	21	0:01.97
191	T	29	13	16	0:01.82
192	F	22	7	15	0:01.94
193	F	16	4	12	0:00.94
194	T	5	4	1	0:00.38
195	T	11	6	5	0:00.60
196	T	11	6	5	0:00.60
197	T	13	7	6	0:01.02
198	F	46	16	30	0:03.53
199	T	8	5	3	0:00.48
200	T	8	5	3	0:00.47
201	T	23	11	12	0:01.20
202	T	41	18	23	0:02.66
203	T	8	5	3	0:00.45
204	T	5	4	1	0:00.31
205	T	5	4	1	0:00.31
206	T	49	21	28	0:03.20
207	T	19	9	10	0:01.24
208	F	20	6	14	0:01.43
209	T	21	10	11	0:01.27
210	F	20	6	14	0:01.21
211	F	38	13	25	0:02.35
212	T	6	4	2	0:00.33
213	T	34	15	19	0:02.41
214	F	16	4	12	0:00.98

Id	Result	#total	#true	#false	Execution time (min:sec)
215	T	16	8	8	0:01.07
216	T	6	5	1	0:00.35
217	T	6	4	2	0:00.36
218	T	6	4	2	0:00.39
219	T	6	4	2	0:00.40
220	F	24	8	16	0:01.60
221	T	8	5	3	0:00.58
222	T	34	15	19	0:02.13
223	F	20	6	14	0:01.39
224	F	25	8	17	0:01.53
225	F	16	4	12	0:00.91
226	T	13	7	6	0:00.75
227	F	16	4	12	0:00.92
228	F	20	6	14	0:01.09
229	F	46	19	27	0:02.80
230	T	11	6	5	0:00.69
231	T	11	6	5	0:00.67
232	F	14	3	11	0:00.87
233	T	10	6	4	0:00.55
234	T	10	6	4	0:00.54
235	F	38	13	25	0:02.59
236	T	11	6	5	0:00.61
237	T	11	6	5	0:00.61
238	T	6	5	1	0:00.33
239	T	6	5	1	0:00.34
240	T	6	4	2	0:00.36
241	T	6	4	2	0:00.37
242	T	6	4	2	0:00.36
243	T	11	6	5	0:00.71
244	T	11	6	5	0:00.69
245	F	20	6	14	0:01.71
246	F	10	1	9	0:00.66
247	F	10	1	9	0:00.67
248	T	6	4	2	0:00.42
249	F	16	4	12	0:01.09
250	F	18	5	13	0:01.18
251	T	19	9	10	0:01.11
252	T	18	9	9	0:01.25
253	T	11	6	5	0:00.72
254	F	22	7	15	0:01.48
255	T	19	9	10	0:01.09
256	T	21	10	11	0:01.48
257	T	6	4	2	0:00.54
258	F	20	6	14	0:01.47
259	T	5	4	1	0:00.29
260	T	5	4	1	0:00.30
261	T	11	6	5	0:00.67
262	T	11	6	5	0:00.65

Id	Result	#total	#true	#false	Execution time (min:sec)
263	T	5	4	1	0:00.34
264	T	5	4	1	0:00.33
265	F	10	1	9	0:00.57
266	F	10	1	9	0:00.55
267	T	5	4	1	0:00.28
268	T	5	4	1	0:00.27
269	T	21	10	11	0:01.30
270	F	14	3	11	0:01.02
271	F	16	4	12	0:01.37
272	T	15	8	7	0:00.91
273	T	5	4	1	0:00.28
274	F	18	5	13	0:01.36
275	T	11	6	5	0:00.60
276	T	24	11	13	0:01.73
277	T	6	5	1	0:00.33
278	T	8	5	3	0:00.63
279	T	8	5	3	0:00.61
280	T	5	4	1	0:00.29
281	T	5	4	1	0:00.29
282	T	5	4	1	0:00.28
283	T	6	4	2	0:00.37
284	T	6	4	2	0:00.37
285	F	86	39	47	0:05.34
286	T	23	11	12	0:01.49
287	T	6	4	2	0:00.34
288	T	5	4	1	0:00.29
289	T	5	4	1	0:00.31
290	T	5	4	1	0:00.30
291	F	14	3	11	0:00.73
292	F	14	3	11	0:00.71
293	T	10	6	4	0:00.57
294	F	20	6	14	0:01.14
295	T	16	8	8	0:00.89
296	F	54	19	35	0:03.69
297	T	5	4	1	0:00.27
298	T	5	4	1	0:00.28
299	T	5	4	1	0:00.28
300	T	5	4	1	0:00.28
301	F	18	5	13	0:01.05
302	T	13	7	6	0:00.80
303	T	10	6	4	0:00.54
304	T	10	6	4	0:00.56
305	T	24	11	13	0:01.32
306	T	23	11	12	0:01.26
307	T	8	5	3	0:00.46
308	T	8	5	3	0:00.77
309	T	6	4	2	0:00.38
310	T	6	4	2	0:00.38

Id	Result	#total	#true	#false	Execution time (min:sec)
311	T	6	4	2	0:00.38
312	T	11	6	5	0:00.76
313	T	16	8	8	0:00.85
314	T	16	8	8	0:00.84
315	T	23	11	12	0:01.36
316	T	5	4	1	0:00.29
317	T	5	4	1	0:00.30
318	T	5	4	1	0:00.30
319	T	28	13	15	0:01.73
320	F	10	1	9	0:00.67
321	T	21	10	11	0:01.58
322	T	23	11	12	0:01.52
323	F	38	13	25	0:02.14
324	F	24	8	16	0:01.41
325	T	37	16	21	0:02.27
326	T	37	16	21	0:02.62
327	T	10	6	4	0:00.67
328	T	10	6	4	0:00.66
329	F	23	7	16	0:01.46
330	T	26	12	14	0:01.53
331	F	52	22	30	0:03.27
332	F	14	3	11	0:00.78
333	T	44	19	25	0:02.63
334	T	11	6	5	0:00.72
335	T	11	6	5	0:00.70
336	T	19	9	10	0:01.06
337	F	10	1	9	0:00.68
338	T	42	18	24	0:02.49
339	T	39	17	22	0:02.13
340	T	8	5	3	0:00.56
341	T	8	5	3	0:00.54
342	T	8	5	3	0:00.44
343	T	8	5	3	0:00.43
344	T	18	9	9	0:01.13
345	T	6	4	2	0:00.34
346	T	6	4	2	0:00.34
347	F	28	10	18	0:01.66
348	T	16	8	8	0:00.87
349	T	5	4	1	0:00.29
350	T	5	4	1	0:00.29
351	T	49	21	28	0:02.83
352	T	5	4	1	0:00.28
353	T	5	4	1	0:00.28
354	T	5	4	1	0:00.28
355	T	5	4	1	0:00.28
356	T	5	4	1	0:00.31
357	T	5	4	1	0:00.30
358	T	5	4	1	0:00.29

Id	Result	#total	#true	#false	Execution time (min:sec)
359	T	10	6	4	0:00.51
360	T	10	6	4	0:00.51
361	T	39	17	22	0:03.09
362	T	16	8	8	0:01.16
363	T	5	4	1	0:00.32
364	T	5	4	1	0:00.34
365	T	10	6	4	0:00.57
366	T	10	6	4	0:00.52
367	F	16	4	12	0:01.15
368	F	14	3	11	0:01.07
369	T	37	16	21	0:02.09
370	F	14	3	11	0:01.26
371	F	16	4	12	0:01.07
372	T	6	4	2	0:00.33
373	T	24	11	13	0:01.42
374	T	11	6	5	0:00.62
375	T	16	8	8	0:01.00
376	T	19	9	10	0:01.15
377	T	10	6	4	0:00.58
378	T	26	12	14	0:01.88
379	T	23	11	12	0:01.26
380	F	116	54	62	0:07.17
381	T	15	8	7	0:01.02
382	F	40	16	24	0:02.16
383	T	29	13	16	0:01.66
384	F	12	2	10	0:01.11
385	T	8	5	3	0:00.47
386	F	12	2	10	0:00.84
387	F	12	2	10	0:00.84
388	F	20	6	14	0:01.39
389	F	20	6	14	0:01.25
390	T	16	8	8	0:00.90
391	T	5	4	1	0:00.28
392	T	5	4	1	0:00.29
393	T	5	4	1	0:00.30
394	F	40	16	24	0:02.39
395	T	5	4	1	0:00.36
396	T	8	5	3	0:00.56
397	T	8	5	3	0:00.57
398	F	8	0	8	0:00.61
399	F	8	0	8	0:00.61
400	T	10	6	4	0:00.60
401	F	72	32	40	0:04.74
402	T	6	4	2	0:00.43
403	F	18	5	13	0:01.28
404	F	14	3	11	0:00.88
405	T	5	4	1	0:00.34
406	T	5	4	1	0:00.33

Id	Result	#total	#true	#false	Execution time (min:sec)
407	T	6	4	2	0:00.64
408	T	6	4	2	0:00.64
409	F	33	11	22	0:02.12
410	T	16	8	8	0:00.88
411	F	20	6	14	0:01.26
412	T	11	6	5	0:00.76
413	T	19	9	10	0:01.08
414	F	42	17	25	0:02.67
415	T	26	12	14	0:01.35
416	T	23	11	12	0:01.18
417	T	8	5	3	0:00.49
418	T	19	9	10	0:01.04
419	F	18	5	13	0:01.09
420	T	28	13	15	0:01.47
421	T	8	5	3	0:00.46
422	T	8	5	3	0:00.47
423	T	8	5	3	0:00.47
424	F	40	16	24	0:02.46
425	F	20	6	14	0:01.30
426	T	21	10	11	0:01.12
427	T	6	4	2	0:00.40
428	T	6	4	2	0:00.40
429	T	37	16	21	0:02.48
430	T	15	8	7	0:00.79
431	T	6	4	2	0:00.36
432	T	6	4	2	0:00.37
433	F	106	51	55	0:07.82
434	T	15	8	7	0:00.90
435	T	19	9	10	0:01.15
436	F	26	9	17	0:01.56
437	F	18	5	13	0:01.13
438	T	36	16	20	0:01.84
439	T	10	6	4	0:00.59
440	T	24	11	13	0:01.34
441	T	8	5	3	0:00.46
442	T	6	4	2	0:00.40
443	T	6	4	2	0:00.41
444	T	13	7	6	0:00.74
445	T	13	7	6	0:00.73
446	T	16	8	8	0:00.93
447	T	6	4	2	0:00.42
448	T	6	4	2	0:00.40
449	T	5	4	1	0:00.35
450	T	5	4	1	0:00.37
451	T	16	8	8	0:00.93
452	T	42	18	24	0:02.67
453	T	5	4	1	0:00.33
454	T	11	6	5	0:00.72

Id	Result	#total	#true	#false	Execution time (min:sec)
455	T	11	6	5	0:00.75
456	T	24	11	13	0:01.44
457	T	39	17	22	0:02.04
458	T	6	4	2	0:00.36
459	T	6	4	2	0:00.37
460	T	6	4	2	0:00.37
461	T	18	9	9	0:01.04
462	F	16	4	12	0:01.03
463	T	5	4	1	0:00.38
464	T	5	4	1	0:00.36
465	T	49	21	28	0:03.19
466	F	12	2	10	0:01.23
467	F	18	5	13	0:01.71
468	F	18	5	13	0:01.36
469	F	10	1	9	0:01.15
470	T	18	9	9	0:00.95
471	T	16	8	8	0:00.96
472	T	21	10	11	0:01.27
473	T	6	4	2	0:00.44
474	T	6	4	2	0:00.46
475	T	6	4	2	0:00.46
476	F	28	9	19	0:02.24
477	T	18	9	9	0:01.55
478	T	6	4	2	0:00.68
479	T	6	4	2	0:00.68
480	T	19	9	10	0:01.07
481	F	67	24	43	0:04.33
482	F	16	4	12	0:00.90
483	T	19	9	10	0:01.07
484	F	24	8	16	0:01.66
485	T	11	6	5	0:00.67
486	F	12	2	10	0:01.69
487	T	10	6	4	0:00.73
488	T	10	6	4	0:00.75
489	T	21	10	11	0:01.26
490	T	16	8	8	0:00.96
491	T	5	4	1	0:00.29
492	T	16	8	8	0:00.93
493	F	25	8	17	0:01.50
494	F	25	8	17	0:01.38
495	T	6	4	2	0:00.42
496	T	6	4	2	0:00.41
497	T	6	4	2	0:00.41
498	T	6	4	2	0:00.38
499	T	6	4	2	0:00.39
500	F	20	6	14	0:01.20

Table 6: Model checking results corresponding to logic property 6

Id	Result	#total	#true	#false	Execution time (min:sec)
1	T	1000	11	989	0:41.32
2	T	1000	11	989	0:39.72
3	T	1000	11	989	0:39.46
4	T	1000	11	989	0:48.18
5	T	1000	11	989	0:41.36
6	T	1000	11	989	0:38.96
7	T	1000	11	989	0:41.37
8	T	1000	11	989	0:40.89
9	T	1000	11	989	0:35.92
10	T	1000	11	989	0:43.91
11	T	1000	11	989	0:39.15
12	T	1000	11	989	0:37.19
13	T	1000	11	989	0:43.43
14	T	1000	11	989	0:53.38
15	T	1000	11	989	0:37.08
16	T	1000	11	989	0:52.87
17	T	1000	11	989	0:36.61
18	T	1000	11	989	0:38.96
19	T	1000	11	989	0:44.88
20	T	1000	11	989	0:55.87
21	T	1000	11	989	0:55.22
22	T	1000	11	989	0:40.70
23	T	1000	11	989	0:39.83
24	T	1000	11	989	0:55.58
25	T	1000	11	989	0:42.43
26	T	1000	11	989	0:52.55
27	T	1000	11	989	0:40.71
28	T	1000	11	989	0:40.82
29	T	1000	11	989	0:47.45
30	T	1000	11	989	1:02.97
31	T	1000	11	989	0:36.39
32	T	1000	11	989	0:22.61
33	T	1000	11	989	0:41.99
34	T	1000	11	989	0:37.28
35	T	1000	11	989	0:59.43
36	T	1000	11	989	0:38.45
37	T	1000	11	989	0:40.98
38	T	1000	11	989	0:40.70
39	T	1000	11	989	0:54.73
40	T	1000	11	989	0:25.91
41	T	1000	11	989	0:41.81
42	T	1000	11	989	0:35.81
43	T	1000	11	989	0:51.26
44	T	1000	11	989	0:52.38
45	T	1000	11	989	0:49.41
46	T	1000	11	989	0:35.31

Id	Result	#total	#true	#false	Execution time (min:sec)
47	T	1000	11	989	0:42.05
48	T	1000	11	989	0:40.83
49	T	1000	11	989	0:53.05
50	T	1000	11	989	0:52.20
51	T	1000	11	989	0:40.80
52	T	1000	11	989	0:39.29
53	T	1000	11	989	0:55.80
54	T	1000	11	989	0:48.61
55	T	1000	11	989	0:42.14
56	T	1000	11	989	0:43.06
57	T	1000	11	989	0:32.14
58	T	1000	11	989	0:43.89
59	T	1000	11	989	1:00.00
60	T	1000	11	989	0:47.06
61	T	1000	11	989	0:40.72
62	T	1000	11	989	0:47.34
63	T	1000	11	989	0:41.20
64	T	1000	11	989	1:00.49
65	T	1000	11	989	0:27.72
66	T	1000	11	989	1:00.59
67	T	1000	11	989	0:47.27
68	T	1000	11	989	0:41.02
69	T	1000	11	989	0:54.78
70	T	1000	11	989	0:46.59
71	T	1000	11	989	0:48.29
72	T	1000	11	989	0:40.79
73	T	1000	11	989	0:58.97
74	T	1000	11	989	0:37.76
75	T	1000	11	989	0:53.19
76	T	1000	11	989	0:55.39
77	T	1000	11	989	0:38.76
78	T	1000	11	989	0:40.05
79	T	1000	11	989	0:42.99
80	T	1000	11	989	0:48.14
81	T	1000	11	989	0:38.39
82	T	1000	11	989	0:51.41
83	T	1000	11	989	0:32.89
84	T	1000	11	989	0:48.53
85	T	1000	11	989	0:54.04
86	T	1000	11	989	0:53.03
87	T	1000	11	989	1:05.00
88	T	1000	11	989	0:55.61
89	T	1000	11	989	0:42.16
90	T	1000	11	989	0:38.52
91	T	1000	11	989	0:43.55
92	T	1000	11	989	0:38.73
93	T	1000	11	989	0:46.47
94	T	1000	11	989	0:39.56

Id	Result	#total	#true	#false	Execution time (min:sec)
95	T	1000	11	989	0:53.79
96	T	1000	11	989	0:46.24
97	T	1000	11	989	0:45.05
98	T	1000	11	989	1:03.33
99	T	1000	11	989	0:35.24
100	T	1000	11	989	0:52.57
101	T	1000	11	989	1:01.35
102	T	1000	11	989	0:40.84
103	T	1000	11	989	0:41.14
104	T	1000	11	989	1:03.93
105	T	1000	11	989	0:46.63
106	T	1000	11	989	0:45.25
107	T	1000	11	989	0:29.18
108	T	1000	11	989	1:01.75
109	T	293	0	293	0:12.04
110	T	1000	11	989	0:35.30
111	T	1000	11	989	0:37.13
112	T	1000	11	989	0:36.44
113	T	1000	11	989	0:36.81
114	T	1000	11	989	0:33.46
115	T	1000	11	989	0:42.30
116	T	1000	11	989	0:40.34
117	T	1000	11	989	0:58.38
118	T	1000	11	989	0:55.53
119	T	1000	11	989	1:07.23
120	T	1000	11	989	0:26.69
121	T	1000	11	989	1:06.48
122	T	1000	11	989	0:42.96
123	T	1000	11	989	0:46.80
124	T	1000	11	989	1:01.48
125	T	1000	11	989	0:41.47
126	T	1000	11	989	0:49.31
127	T	1000	11	989	0:41.30
128	T	1000	11	989	1:07.01
129	T	1000	11	989	0:51.80
130	T	1000	11	989	0:46.19
131	T	1000	11	989	0:42.41
132	T	1000	11	989	0:52.81
133	T	1000	11	989	0:47.86
134	T	1000	11	989	0:40.53
135	T	1000	11	989	0:52.21
136	T	1000	11	989	0:40.60
137	T	1000	11	989	0:40.59
138	T	1000	11	989	0:43.21
139	T	1000	11	989	0:40.02
140	T	1000	11	989	0:42.25
141	T	1000	11	989	0:39.85
142	T	1000	11	989	0:48.07

Id	Result	#total	#true	#false	Execution time (min:sec)
143	T	1000	11	989	0:41.23
144	T	1000	11	989	0:35.28
145	T	1000	11	989	0:40.04
146	T	1000	11	989	0:54.04
147	T	1000	11	989	0:47.68
148	T	1000	11	989	0:39.60
149	T	1000	11	989	0:46.94
150	T	1000	11	989	0:36.30
151	T	1000	11	989	0:44.11
152	T	1000	11	989	0:38.62
153	T	1000	11	989	0:42.76
154	T	1000	11	989	0:41.00
155	T	1000	11	989	0:34.57
156	T	1000	11	989	0:45.69
157	T	1000	11	989	1:05.75
158	T	1000	11	989	0:52.71
159	T	1000	11	989	0:33.69
160	T	1000	11	989	0:45.20
161	T	1000	11	989	0:41.52
162	T	1000	11	989	0:50.47
163	T	1000	11	989	0:33.60
164	T	1000	11	989	0:42.17
165	T	1000	11	989	0:48.77
166	T	1000	11	989	0:32.97
167	T	1000	11	989	0:45.43
168	T	1000	11	989	0:36.27
169	T	1000	11	989	0:49.64
170	T	1000	11	989	0:51.75
171	T	1000	11	989	0:40.14
172	T	1000	11	989	0:41.04
173	T	1000	11	989	0:57.10
174	T	1000	11	989	0:27.11
175	T	1000	11	989	0:41.97
176	T	1000	11	989	0:47.45
177	T	1000	11	989	0:41.53
178	T	1000	11	989	0:54.98
179	T	1000	11	989	0:58.37
180	T	1000	11	989	0:58.20
181	T	1000	11	989	1:05.78
182	T	1000	11	989	0:41.49
183	T	1000	11	989	0:31.71
184	T	1000	11	989	1:00.14
185	T	1000	11	989	0:52.87
186	T	1000	11	989	0:40.97
187	T	1000	11	989	0:34.34
188	T	1000	11	989	0:43.38
189	T	1000	11	989	0:43.65
190	T	1000	11	989	0:46.79

Id	Result	#total	#true	#false	Execution time (min:sec)
191	T	1000	11	989	0:37.04
192	T	1000	11	989	0:41.05
193	T	1000	11	989	0:49.64
194	T	1000	11	989	0:41.32
195	T	1000	11	989	0:43.21
196	T	1000	11	989	0:41.89
197	T	1000	11	989	0:47.62
198	T	1000	11	989	0:42.99
199	T	1000	11	989	0:39.31
200	T	1000	11	989	0:35.83
201	T	1000	11	989	0:38.33
202	T	1000	11	989	0:42.37
203	T	1000	11	989	0:48.41
204	T	1000	11	989	0:43.99
205	T	1000	11	989	0:44.64
206	T	1000	11	989	0:48.65
207	T	1000	11	989	0:42.39
208	T	1000	11	989	0:47.12
209	T	1000	11	989	0:56.16
210	T	1000	11	989	0:41.27
211	T	1000	11	989	0:57.63
212	T	1000	11	989	0:42.43
213	T	1000	11	989	0:33.27
214	T	1000	11	989	0:50.85
215	T	1000	11	989	0:29.22
216	T	1000	11	989	0:42.68
217	T	1000	11	989	0:41.52
218	T	1000	11	989	0:40.66
219	T	1000	11	989	0:42.01
220	T	1000	11	989	0:52.82
221	F	94	6	88	0:04.02
222	T	1000	11	989	0:35.71
223	T	1000	11	989	0:49.06
224	T	1000	11	989	0:40.91
225	T	1000	11	989	0:56.13
226	T	1000	11	989	1:02.99
227	T	1000	11	989	0:45.48
228	T	1000	11	989	0:40.81
229	T	1000	11	989	0:42.18
230	T	1000	11	989	0:38.68
231	T	1000	11	989	0:58.03
232	T	1000	11	989	0:30.94
233	T	1000	11	989	0:28.33
234	T	1000	11	989	0:34.53
235	T	1000	11	989	0:41.32
236	T	1000	11	989	0:50.15
237	T	1000	11	989	0:41.50
238	T	1000	11	989	0:37.61

Id	Result	#total	#true	#false	Execution time (min:sec)
239	T	1000	11	989	0:29.01
240	T	1000	11	989	0:59.08
241	T	1000	11	989	0:41.20
242	T	1000	11	989	0:52.59
243	T	1000	11	989	0:41.41
244	T	1000	11	989	0:39.87
245	T	1000	11	989	1:00.68
246	T	1000	11	989	0:42.71
247	T	1000	11	989	0:48.53
248	T	1000	11	989	0:30.68
249	T	1000	11	989	0:52.62
250	T	1000	11	989	0:42.90
251	T	1000	11	989	0:40.60
252	T	1000	11	989	0:46.33
253	T	1000	11	989	0:40.86
254	T	1000	11	989	0:45.90
255	T	1000	11	989	0:51.55
256	T	1000	11	989	0:51.79
257	T	1000	11	989	0:57.92
258	T	1000	11	989	0:43.25
259	T	1000	11	989	0:45.58
260	T	1000	11	989	0:28.87
261	T	1000	11	989	0:34.70
262	T	1000	11	989	0:40.48
263	T	1000	11	989	0:49.35
264	T	1000	11	989	0:36.18
265	T	1000	11	989	0:37.38
266	T	1000	11	989	0:38.25
267	T	1000	11	989	0:37.98
268	T	1000	11	989	0:43.93
269	T	1000	11	989	0:40.55
270	T	1000	11	989	0:41.22
271	T	1000	11	989	0:41.31
272	T	1000	11	989	0:41.73
273	T	1000	11	989	0:48.39
274	T	1000	11	989	0:41.76
275	T	1000	11	989	0:38.76
276	T	1000	11	989	0:36.67
277	T	1000	11	989	0:39.94
278	T	1000	11	989	0:40.94
279	T	1000	11	989	0:53.25
280	T	1000	11	989	0:35.72
281	T	1000	11	989	0:44.33
282	T	1000	11	989	0:41.69
283	T	1000	11	989	0:35.15
284	T	1000	11	989	0:46.34
285	T	1000	11	989	0:48.94
286	T	1000	11	989	0:40.25

Id	Result	#total	#true	#false	Execution time (min:sec)
287	T	1000	11	989	0:42.03
288	T	1000	11	989	0:33.59
289	T	1000	11	989	0:33.54
290	T	1000	11	989	0:48.38
291	T	1000	11	989	0:28.99
292	T	1000	11	989	0:38.39
293	T	1000	11	989	0:45.72
294	T	1000	11	989	0:39.48
295	T	1000	11	989	0:40.49
296	T	1000	11	989	0:55.39
297	T	293	0	293	0:12.24
298	T	1000	11	989	0:32.57
299	T	1000	11	989	0:35.95
300	T	1000	11	989	0:41.50
301	T	293	0	293	0:12.24
302	T	1000	11	989	1:00.11
303	T	1000	11	989	0:51.97
304	T	1000	11	989	0:43.99
305	T	1000	11	989	0:35.95
306	T	1000	11	989	1:06.49
307	T	1000	11	989	0:55.31
308	T	1000	11	989	0:52.45
309	T	1000	11	989	0:35.76
310	T	1000	11	989	0:42.56
311	T	1000	11	989	0:40.98
312	T	1000	11	989	0:36.11
313	T	1000	11	989	0:44.60
314	T	293	0	293	0:17.02
315	T	1000	11	989	0:52.70
316	T	1000	11	989	0:44.84
317	T	1000	11	989	0:44.25
318	T	1000	11	989	0:40.73
319	T	1000	11	989	1:04.22
320	T	1000	11	989	0:40.66
321	T	1000	11	989	0:40.95
322	T	1000	11	989	0:51.59
323	T	1000	11	989	0:40.93
324	T	1000	11	989	0:38.02
325	T	1000	11	989	0:46.17
326	T	1000	11	989	0:47.51
327	T	1000	11	989	0:55.26
328	T	1000	11	989	0:26.86
329	T	1000	11	989	0:37.05
330	T	1000	11	989	0:44.19
331	T	1000	11	989	0:33.52
332	T	1000	11	989	0:54.91
333	T	1000	11	989	0:52.75
334	T	1000	11	989	0:39.04

Id	Result	#total	#true	#false	Execution time (min:sec)
335	T	1000	11	989	0:38.70
336	T	1000	11	989	0:42.68
337	T	1000	11	989	0:53.67
338	T	1000	11	989	0:42.58
339	T	1000	11	989	0:52.63
340	T	1000	11	989	0:50.94
341	T	1000	11	989	0:48.77
342	T	1000	11	989	0:52.53
343	T	1000	11	989	0:41.66
344	T	1000	11	989	0:43.76
345	T	1000	11	989	0:41.06
346	T	1000	11	989	0:48.31
347	T	1000	11	989	0:48.84
348	T	1000	11	989	0:48.37
349	T	1000	11	989	0:37.79
350	T	1000	11	989	0:38.60
351	T	1000	11	989	0:39.55
352	T	1000	11	989	0:51.49
353	T	1000	11	989	0:32.62
354	T	1000	11	989	0:41.41
355	T	1000	11	989	0:42.03
356	T	1000	11	989	0:40.83
357	T	1000	11	989	1:05.89
358	T	1000	11	989	0:35.40
359	T	1000	11	989	0:32.31
360	T	1000	11	989	0:56.55
361	T	293	0	293	0:18.66
362	T	1000	11	989	0:41.61
363	T	1000	11	989	0:34.19
364	T	1000	11	989	1:00.16
365	T	1000	11	989	0:53.72
366	T	1000	11	989	0:41.11
367	T	1000	11	989	0:40.50
368	T	1000	11	989	0:40.37
369	T	1000	11	989	0:46.72
370	T	1000	11	989	0:42.22
371	T	1000	11	989	0:42.78
372	T	1000	11	989	0:54.06
373	T	1000	11	989	0:56.39
374	T	1000	11	989	0:47.68
375	T	1000	11	989	0:39.49
376	T	1000	11	989	0:38.38
377	T	1000	11	989	0:40.00
378	T	1000	11	989	0:52.17
379	T	1000	11	989	0:41.60
380	T	1000	11	989	1:02.65
381	T	1000	11	989	0:41.49
382	T	1000	11	989	0:54.41

Id	Result	#total	#true	#false	Execution time (min:sec)
383	T	293	0	293	0:12.19
384	T	1000	11	989	0:40.33
385	T	1000	11	989	0:42.70
386	T	1000	11	989	0:43.34
387	T	1000	11	989	0:41.06
388	T	293	0	293	0:12.20
389	T	293	0	293	0:12.12
390	T	1000	11	989	0:47.69
391	T	1000	11	989	0:28.40
392	T	1000	11	989	0:45.53
393	T	293	0	293	0:16.86
394	T	1000	11	989	0:40.63
395	T	1000	11	989	0:39.33
396	T	1000	11	989	0:41.50
397	T	1000	11	989	0:50.53
398	T	1000	11	989	0:44.74
399	T	1000	11	989	1:02.75
400	T	1000	11	989	0:30.77
401	T	1000	11	989	1:02.69
402	T	1000	11	989	0:43.07
403	T	1000	11	989	1:00.13
404	T	1000	11	989	0:42.08
405	T	1000	11	989	0:32.26
406	T	1000	11	989	0:42.07
407	T	1000	11	989	0:56.48
408	T	1000	11	989	0:25.37
409	T	1000	11	989	0:26.30
410	T	1000	11	989	0:41.38
411	T	1000	11	989	0:38.55
412	T	1000	11	989	0:39.50
413	T	1000	11	989	0:48.73
414	T	293	0	293	0:12.27
415	T	1000	11	989	0:38.98
416	T	1000	11	989	0:52.67
417	T	1000	11	989	0:41.84
418	T	1000	11	989	0:52.65
419	T	1000	11	989	0:55.97
420	T	1000	11	989	0:39.81
421	T	1000	11	989	0:41.65
422	T	1000	11	989	0:26.70
423	T	1000	11	989	0:35.04
424	T	1000	11	989	0:32.43
425	T	1000	11	989	0:57.57
426	T	1000	11	989	0:42.72
427	T	1000	11	989	0:41.67
428	T	1000	11	989	0:35.08
429	T	1000	11	989	0:41.03
430	T	1000	11	989	1:05.23

Id	Result	#total	#true	#false	Execution time (min:sec)
431	T	1000	11	989	0:40.43
432	T	1000	11	989	0:35.05
433	T	1000	11	989	0:52.66
434	T	1000	11	989	0:41.59
435	T	1000	11	989	0:39.80
436	T	1000	11	989	0:40.46
437	T	1000	11	989	0:43.86
438	T	1000	11	989	0:41.94
439	T	1000	11	989	0:36.38
440	T	1000	11	989	0:47.33
441	T	1000	11	989	1:00.66
442	T	1000	11	989	0:55.14
443	T	1000	11	989	0:47.40
444	T	1000	11	989	0:40.18
445	T	1000	11	989	0:59.09
446	T	1000	11	989	0:44.33
447	T	1000	11	989	0:53.70
448	T	1000	11	989	0:38.51
449	T	1000	11	989	0:45.91
450	T	1000	11	989	0:41.25
451	T	1000	11	989	0:43.60
452	T	1000	11	989	0:41.35
453	T	1000	11	989	0:33.71
454	T	1000	11	989	0:48.40
455	T	1000	11	989	0:30.75
456	T	1000	11	989	0:43.62
457	T	1000	11	989	0:34.40
458	T	1000	11	989	0:45.30
459	T	1000	11	989	0:36.64
460	T	1000	11	989	0:56.36
461	T	1000	11	989	0:43.02
462	T	1000	11	989	0:53.85
463	T	1000	11	989	1:07.84
464	T	1000	11	989	0:27.33
465	T	1000	11	989	0:48.61
466	T	1000	11	989	0:55.21
467	T	1000	11	989	0:41.43
468	T	1000	11	989	0:41.21
469	T	1000	11	989	0:41.90
470	T	1000	11	989	0:43.10
471	T	1000	11	989	0:45.71
472	T	1000	11	989	0:41.51
473	T	1000	11	989	0:54.99
474	T	1000	11	989	0:48.78
475	T	1000	11	989	0:31.48
476	T	1000	11	989	0:34.22
477	T	1000	11	989	0:53.41
478	T	1000	11	989	0:46.39

Id	Result	#total	#true	#false	Execution time (min:sec)
479	T	1000	11	989	0:47.68
480	T	1000	11	989	0:40.59
481	T	1000	11	989	0:55.16
482	T	1000	11	989	0:50.41
483	T	293	0	293	0:12.46
484	T	1000	11	989	0:42.09
485	T	1000	11	989	0:51.41
486	T	1000	11	989	0:39.71
487	T	1000	11	989	0:41.38
488	T	1000	11	989	0:36.88
489	T	1000	11	989	0:54.65
490	T	1000	11	989	0:44.37
491	T	1000	11	989	0:41.62
492	T	1000	11	989	0:42.35
493	T	1000	11	989	0:53.16
494	T	1000	11	989	0:22.51
495	T	1000	11	989	0:42.10
496	T	1000	11	989	0:36.58
497	T	1000	11	989	0:40.76
498	T	1000	11	989	0:37.50
499	T	1000	11	989	0:41.52
500	T	1000	11	989	0:49.92

Table 7: Model checking results corresponding to logic property 7

Id	Result	#total	#true	#false	Execution time (min:sec)
1	T	69	68	1	0:02.92
2	T	58	58	0	0:02.48
3	T	58	58	0	0:02.49
4	T	58	58	0	0:02.51
5	T	193	183	10	0:13.06
6	T	69	68	1	0:02.95
7	T	152	145	7	0:06.42
8	T	207	196	11	0:08.75
9	T	83	81	2	0:03.54
10	T	83	81	2	0:03.51
11	T	166	158	8	0:07.00
12	T	138	132	6	0:05.81
13	T	83	81	2	0:03.51
14	T	96	93	3	0:04.17
15	T	193	183	10	0:08.16
16	T	83	81	2	0:03.53
17	T	262	247	15	0:11.08
18	T	96	93	3	0:04.05
19	T	110	106	4	0:04.70

Id	Result	#total	#true	#false	Execution time (min:sec)
20	T	96	93	3	0:04.04
21	T	58	58	0	0:02.49
22	T	96	93	3	0:04.09
23	T	83	81	2	0:03.52
24	T	83	81	2	0:03.53
25	T	110	106	4	0:04.64
26	T	58	58	0	0:02.46
27	T	235	222	13	0:10.00
28	T	96	93	3	0:04.07
29	T	221	209	12	0:09.27
30	T	138	132	6	0:05.81
31	T	96	93	3	0:04.13
32	T	166	158	8	0:10.57
33	T	110	106	4	0:04.64
34	T	69	68	1	0:04.52
35	T	138	132	6	0:05.85
36	T	193	183	10	0:06.53
37	T	96	93	3	0:04.09
38	T	69	68	1	0:02.97
39	T	83	81	2	0:03.53
40	T	96	93	3	0:04.07
41	T	58	58	0	0:02.49
42	T	83	81	2	0:03.50
43	T	83	81	2	0:03.56
44	T	179	170	9	0:07.52
45	T	124	119	5	0:07.17
46	T	110	106	4	0:04.68
47	T	138	132	6	0:05.82
48	T	124	119	5	0:05.25
49	T	138	132	6	0:05.83
50	T	110	106	4	0:04.65
51	T	69	68	1	0:02.95
52	T	83	81	2	0:03.51
53	T	83	81	2	0:04.17
54	T	124	119	5	0:05.22
55	T	69	68	1	0:02.92
56	T	58	58	0	0:02.47
57	T	96	93	3	0:04.05
58	T	110	106	4	0:04.70
59	T	83	81	2	0:03.54
60	T	124	119	5	0:05.19
61	T	69	68	1	0:02.94
62	T	83	81	2	0:03.56
63	T	58	58	0	0:02.46
64	T	166	158	8	0:07.22
65	T	166	158	8	0:06.95
66	T	58	58	0	0:02.48
67	T	96	93	3	0:04.11

Id	Result	#total	#true	#false	Execution time (min:sec)
68	T	152	145	7	0:06.50
69	T	69	68	1	0:02.92
70	T	83	81	2	0:03.52
71	T	96	93	3	0:04.08
72	T	83	81	2	0:03.51
73	T	58	58	0	0:02.46
74	T	166	158	8	0:06.99
75	T	193	183	10	0:08.12
76	T	152	145	7	0:06.42
77	T	69	68	1	0:02.95
78	T	69	68	1	0:02.97
79	T	69	68	1	0:02.96
80	T	58	58	0	0:02.51
81	T	138	132	6	0:05.83
82	T	83	81	2	0:03.56
83	T	96	93	3	0:04.08
84	T	124	119	5	0:08.54
85	T	138	132	6	0:05.84
86	T	69	68	1	0:02.94
87	T	83	81	2	0:03.52
88	T	193	183	10	0:13.45
89	T	138	132	6	0:05.94
90	T	69	68	1	0:02.93
91	T	138	132	6	0:05.81
92	T	96	93	3	0:04.09
93	T	166	158	8	0:07.01
94	T	83	81	2	0:03.58
95	T	138	132	6	0:05.81
96	T	110	106	4	0:04.69
97	T	124	119	5	0:05.25
98	T	110	106	4	0:05.43
99	T	152	145	7	0:06.43
100	T	96	93	3	0:04.11
101	T	83	81	2	0:03.50
102	T	124	119	5	0:05.19
103	T	138	132	6	0:05.85
104	T	179	170	9	0:07.53
105	T	124	119	5	0:05.25
106	T	110	106	4	0:04.66
107	T	152	145	7	0:06.41
108	T	58	58	0	0:02.49
109	T	96	93	3	0:04.07
110	T	124	119	5	0:05.25
111	T	124	119	5	0:05.25
112	T	69	68	1	0:02.90
113	T	110	106	4	0:04.66
114	T	110	106	4	0:04.64
115	T	179	170	9	0:07.56

Id	Result	#total	#true	#false	Execution time (min:sec)
116	T	124	119	5	0:05.25
117	T	69	68	1	0:02.91
118	T	138	132	6	0:08.26
119	T	166	158	8	0:07.97
120	T	96	93	3	0:04.08
121	T	83	81	2	0:04.21
122	T	110	106	4	0:04.66
123	T	96	93	3	0:05.52
124	T	124	119	5	0:05.25
125	T	179	170	9	0:07.53
126	T	83	81	2	0:03.54
127	T	152	145	7	0:06.40
128	T	96	93	3	0:04.06
129	T	83	81	2	0:03.50
130	T	96	93	3	0:04.06
131	T	152	145	7	0:06.38
132	T	83	81	2	0:03.88
133	T	124	119	5	0:05.22
134	T	83	81	2	0:03.54
135	T	96	93	3	0:04.04
136	T	83	81	2	0:03.56
137	T	69	68	1	0:02.95
138	T	110	106	4	0:04.71
139	T	69	68	1	0:02.95
140	T	96	93	3	0:04.10
141	T	83	81	2	0:03.50
142	T	248	234	14	0:12.71
143	T	110	106	4	0:04.67
144	T	69	68	1	0:02.91
145	T	96	93	3	0:04.09
146	T	69	68	1	0:02.91
147	T	58	58	0	0:02.45
148	T	83	81	2	0:03.57
149	T	69	68	1	0:02.94
150	T	69	68	1	0:02.98
151	T	83	81	2	0:03.52
152	T	69	68	1	0:02.94
153	T	138	132	6	0:09.09
154	T	83	81	2	0:03.53
155	T	96	93	3	0:04.08
156	T	110	106	4	0:04.66
157	T	110	106	4	0:04.67
158	T	58	58	0	0:02.48
159	T	138	132	6	0:05.84
160	T	262	247	15	0:09.40
161	T	124	119	5	0:05.27
162	T	83	81	2	0:03.53
163	T	166	158	8	0:07.01

Id	Result	#total	#true	#false	Execution time (min:sec)
164	T	69	68	1	0:02.94
165	T	110	106	4	0:04.66
166	T	58	58	0	0:03.76
167	T	207	196	11	0:08.70
168	T	83	81	2	0:03.53
169	T	96	93	3	0:04.07
170	T	179	170	9	0:07.52
171	T	124	119	5	0:05.20
172	T	152	145	7	0:06.40
173	T	138	132	6	0:05.84
174	T	166	158	8	0:07.01
175	T	58	58	0	0:02.48
176	T	83	81	2	0:03.54
177	T	124	119	5	0:05.36
178	T	110	106	4	0:04.75
179	T	124	119	5	0:05.25
180	T	96	93	3	0:04.07
181	T	69	68	1	0:02.92
182	T	83	81	2	0:03.53
183	T	58	58	0	0:02.45
184	T	124	119	5	0:05.25
185	T	69	68	1	0:02.95
186	T	96	93	3	0:04.09
187	T	110	106	4	0:04.67
188	T	83	81	2	0:03.57
189	T	110	106	4	0:04.67
190	T	138	132	6	0:05.81
191	T	110	106	4	0:04.69
192	T	193	183	10	0:08.09
193	T	69	68	1	0:02.93
194	T	221	209	12	0:13.42
195	T	58	58	0	0:02.46
196	T	69	68	1	0:02.94
197	T	58	58	0	0:02.47
198	T	83	81	2	0:03.56
199	T	179	170	9	0:07.59
200	T	152	145	7	0:06.39
201	T	110	106	4	0:04.71
202	T	110	106	4	0:04.64
203	T	235	222	13	0:09.87
204	T	69	68	1	0:02.96
205	T	235	222	13	0:08.47
206	T	96	93	3	0:04.09
207	T	110	106	4	0:04.66
208	T	110	106	4	0:04.67
209	T	124	119	5	0:05.27
210	T	110	106	4	0:04.71
211	T	96	93	3	0:04.06

Id	Result	#total	#true	#false	Execution time (min:sec)
212	T	58	58	0	0:02.45
213	T	96	93	3	0:04.08
214	T	207	196	11	0:08.77
215	T	83	81	2	0:03.53
216	T	69	68	1	0:04.30
217	T	83	81	2	0:03.51
218	T	96	93	3	0:04.06
219	T	69	68	1	0:02.94
220	T	138	132	6	0:05.81
221	T	124	119	5	0:05.30
222	T	276	260	16	0:13.14
223	T	124	119	5	0:05.24
224	T	69	68	1	0:02.93
225	T	83	81	2	0:03.50
226	T	58	58	0	0:02.49
227	T	138	132	6	0:08.27
228	T	96	93	3	0:04.04
229	T	83	81	2	0:03.54
230	T	69	68	1	0:02.92
231	T	69	68	1	0:02.94
232	T	110	106	4	0:04.65
233	T	110	106	4	0:04.66
234	T	58	58	0	0:02.48
235	T	124	119	5	0:05.22
236	T	96	93	3	0:04.06
237	T	138	132	6	0:05.86
238	T	96	93	3	0:04.06
239	T	96	93	3	0:04.05
240	T	96	93	3	0:04.07
241	T	69	68	1	0:02.96
242	T	69	68	1	0:02.95
243	T	124	119	5	0:05.24
244	T	69	68	1	0:02.92
245	T	58	58	0	0:02.48
246	T	166	158	8	0:07.02
247	T	152	145	7	0:06.47
248	T	124	119	5	0:05.20
249	T	58	58	0	0:02.47
250	T	69	68	1	0:02.99
251	T	110	106	4	0:05.85
252	T	166	158	8	0:07.71
253	T	83	81	2	0:03.55
254	T	124	119	5	0:06.12
255	T	69	68	1	0:02.95
256	T	58	58	0	0:02.48
257	T	69	68	1	0:02.96
258	T	58	58	0	0:02.50
259	T	83	81	2	0:03.56

Id	Result	#total	#true	#false	Execution time (min:sec)
260	T	124	119	5	0:05.21
261	T	83	81	2	0:03.59
262	T	96	93	3	0:04.03
263	T	193	183	10	0:08.12
264	T	58	58	0	0:02.46
265	T	179	170	9	0:11.15
266	T	96	93	3	0:04.09
267	T	207	196	11	0:08.78
268	T	83	81	2	0:03.52
269	T	58	58	0	0:02.49
270	T	179	170	9	0:07.58
271	T	69	68	1	0:02.95
272	T	179	170	9	0:07.54
273	T	58	58	0	0:02.48
274	T	96	93	3	0:04.12
275	T	58	58	0	0:02.46
276	T	138	132	6	0:05.87
277	T	58	58	0	0:02.48
278	T	110	106	4	0:05.91
279	T	96	93	3	0:04.07
280	T	83	81	2	0:03.51
281	T	110	106	4	0:04.72
282	T	110	106	4	0:04.65
283	T	83	81	2	0:03.50
284	T	179	170	9	0:07.48
285	T	110	106	4	0:04.65
286	T	110	106	4	0:04.68
287	T	58	58	0	0:02.50
288	T	69	68	1	0:02.95
289	T	69	68	1	0:02.92
290	T	58	58	0	0:02.46
291	T	58	58	0	0:02.46
292	T	124	119	5	0:05.26
293	T	69	68	1	0:02.91
294	T	83	81	2	0:05.62
295	T	83	81	2	0:03.51
296	T	83	81	2	0:03.55
297	T	69	68	1	0:02.94
298	T	69	68	1	0:02.89
299	T	96	93	3	0:04.06
300	T	124	119	5	0:05.25
301	T	69	68	1	0:02.94
302	T	124	119	5	0:05.20
303	T	69	68	1	0:02.94
304	T	96	93	3	0:04.08
305	T	69	68	1	0:02.92
306	T	193	183	10	0:08.19
307	T	83	81	2	0:03.49

Id	Result	#total	#true	#false	Execution time (min:sec)
308	T	83	81	2	0:03.58
309	T	193	183	10	0:08.15
310	T	96	93	3	0:04.04
311	T	83	81	2	0:03.53
312	T	58	58	0	0:02.47
313	T	69	68	1	0:02.90
314	T	193	183	10	0:12.96
315	T	83	81	2	0:03.58
316	T	124	119	5	0:05.23
317	T	83	81	2	0:03.55
318	T	83	81	2	0:03.52
319	T	110	106	4	0:04.66
320	T	124	119	5	0:05.25
321	T	96	93	3	0:04.08
322	T	110	106	4	0:04.70
323	T	207	196	11	0:10.79
324	T	124	119	5	0:05.25
325	T	83	81	2	0:03.54
326	T	110	106	4	0:06.50
327	T	69	68	1	0:02.97
328	T	58	58	0	0:02.45
329	T	138	132	6	0:05.86
330	T	69	68	1	0:02.91
331	T	69	68	1	0:02.92
332	T	96	93	3	0:04.08
333	T	83	81	2	0:03.50
334	T	83	81	2	0:03.57
335	T	166	158	8	0:07.48
336	T	110	106	4	0:04.63
337	T	221	209	12	0:09.34
338	T	69	68	1	0:04.78
339	T	110	106	4	0:04.68
340	T	83	81	2	0:03.52
341	T	124	119	5	0:05.20
342	T	58	58	0	0:02.46
343	T	83	81	2	0:03.54
344	T	235	222	13	0:09.87
345	T	83	81	2	0:03.53
346	T	69	68	1	0:02.95
347	T	69	68	1	0:02.93
348	T	69	68	1	0:02.95
349	T	58	58	0	0:02.48
350	T	83	81	2	0:03.58
351	T	193	183	10	0:08.25
352	T	58	58	0	0:02.47
353	T	83	81	2	0:03.49
354	T	96	93	3	0:04.12
355	T	110	106	4	0:06.06

Id	Result	#total	#true	#false	Execution time (min:sec)
356	T	124	119	5	0:05.24
357	T	138	132	6	0:05.82
358	T	96	93	3	0:04.09
359	T	83	81	2	0:03.54
360	T	83	81	2	0:03.50
361	T	138	132	6	0:05.83
362	T	110	106	4	0:04.66
363	T	138	132	6	0:05.87
364	T	110	106	4	0:04.68
365	T	152	145	7	0:06.51
366	T	58	58	0	0:04.03
367	T	58	58	0	0:02.48
368	T	69	68	1	0:02.90
369	T	221	209	12	0:09.27
370	T	179	170	9	0:07.52
371	T	58	58	0	0:02.81
372	T	152	145	7	0:06.46
373	T	110	106	4	0:04.64
374	T	124	119	5	0:04.79
375	T	124	119	5	0:05.25
376	T	179	170	9	0:07.51
377	T	83	81	2	0:03.54
378	T	96	93	3	0:04.15
379	T	96	93	3	0:04.08
380	T	58	58	0	0:02.49
381	T	96	93	3	0:04.14
382	T	83	81	2	0:03.55
383	T	166	158	8	0:07.04
384	T	83	81	2	0:05.71
385	T	96	93	3	0:05.52
386	T	166	158	8	0:07.01
387	T	58	58	0	0:02.51
388	T	152	145	7	0:06.40
389	T	207	196	11	0:08.77
390	T	96	93	3	0:04.12
391	T	83	81	2	0:03.57
392	T	110	106	4	0:04.68
393	T	124	119	5	0:07.32
394	T	83	81	2	0:03.52
395	T	69	68	1	0:02.91
396	T	58	58	0	0:02.47
397	T	152	145	7	0:06.47
398	T	110	106	4	0:04.65
399	T	110	106	4	0:04.65
400	T	96	93	3	0:06.01
401	T	83	81	2	0:04.30
402	T	124	119	5	0:05.23
403	T	96	93	3	0:04.09

Id	Result	#total	#true	#false	Execution time (min:sec)
404	T	83	81	2	0:03.52
405	T	138	132	6	0:05.87
406	T	152	145	7	0:06.46
407	T	83	81	2	0:03.51
408	T	179	170	9	0:07.57
409	T	83	81	2	0:03.52
410	T	58	58	0	0:04.03
411	T	152	145	7	0:06.43
412	T	69	68	1	0:02.93
413	T	96	93	3	0:06.13
414	T	138	132	6	0:07.44
415	T	83	81	2	0:03.51
416	T	83	81	2	0:03.53
417	T	138	132	6	0:05.84
418	T	110	106	4	0:04.69
419	T	96	93	3	0:04.06
420	T	124	119	5	0:05.26
421	T	69	68	1	0:02.92
422	T	83	81	2	0:03.56
423	T	110	106	4	0:04.68
424	T	58	58	0	0:02.48
425	T	69	68	1	0:02.95
426	T	96	93	3	0:04.07
427	T	69	68	1	0:04.12
428	T	124	119	5	0:05.27
429	T	83	81	2	0:03.51
430	T	58	58	0	0:02.48
431	T	207	196	11	0:09.88
432	T	69	68	1	0:02.96
433	T	58	58	0	0:02.52
434	T	83	81	2	0:03.51
435	T	110	106	4	0:04.66
436	T	138	132	6	0:05.81
437	T	69	68	1	0:02.95
438	T	83	81	2	0:03.56
439	T	83	81	2	0:03.51
440	T	96	93	3	0:04.08
441	T	124	119	5	0:05.27
442	T	83	81	2	0:03.52
443	T	58	58	0	0:02.48
444	T	58	58	0	0:02.51
445	T	83	81	2	0:03.54
446	T	58	58	0	0:02.47
447	T	69	68	1	0:02.96
448	T	96	93	3	0:04.07
449	T	69	68	1	0:02.94
450	T	83	81	2	0:03.50
451	T	83	81	2	0:03.55

Id	Result	#total	#true	#false	Execution time (min:sec)
452	T	152	145	7	0:06.43
453	T	58	58	0	0:02.47
454	T	110	106	4	0:04.70
455	T	179	170	9	0:09.86
456	T	58	58	0	0:02.50
457	T	96	93	3	0:04.09
458	T	58	58	0	0:02.49
459	T	124	119	5	0:06.49
460	T	152	145	7	0:06.39
461	T	124	119	5	0:05.25
462	T	58	58	0	0:02.50
463	T	152	145	7	0:09.30
464	T	69	68	1	0:02.96
465	T	124	119	5	0:05.32
466	T	83	81	2	0:03.53
467	T	83	81	2	0:03.69
468	T	83	81	2	0:03.58
469	T	69	68	1	0:02.93
470	T	69	68	1	0:02.91
471	T	58	58	0	0:02.47
472	T	58	58	0	0:02.47
473	T	110	106	4	0:04.64
474	T	69	68	1	0:02.97
475	T	83	81	2	0:03.52
476	T	83	81	2	0:03.52
477	T	96	93	3	0:04.05
478	T	179	170	9	0:07.56
479	T	83	81	2	0:03.52
480	T	110	106	4	0:04.73
481	T	69	68	1	0:02.93
482	T	69	68	1	0:03.46
483	T	138	132	6	0:05.82
484	T	124	119	5	0:05.23
485	T	110	106	4	0:04.64
486	T	179	170	9	0:07.58
487	T	69	68	1	0:02.94
488	T	124	119	5	0:06.76
489	T	58	58	0	0:02.48
490	T	207	196	11	0:05.80
491	T	110	106	4	0:04.68
492	T	96	93	3	0:04.08
493	T	179	170	9	0:07.49
494	T	152	145	7	0:06.41
495	T	96	93	3	0:04.08
496	T	96	93	3	0:05.71
497	T	83	81	2	0:03.58
498	T	138	132	6	0:05.81
499	T	83	81	2	0:04.80

Id	Result	#total	#true	#false	Execution time (min:sec)
500	T	124	119	5	0:05.25

Table 8: Model checking results corresponding to logic property 8

Id	Result	#total	#true	#false	Execution time (min:sec)
1	T	14	14	0	0:00.64
2	T	14	13	1	0:00.64
3	T	14	13	1	0:00.64
4	T	21	18	3	0:00.95
5	T	18	16	2	0:00.82
6	T	21	18	3	0:00.96
7	T	14	13	1	0:00.63
8	T	14	13	1	0:00.64
9	T	38	30	8	0:01.85
10	T	38	30	8	0:01.70
11	T	24	20	4	0:01.08
12	T	28	23	5	0:01.24
13	T	35	28	7	0:01.56
14	T	21	18	3	0:00.94
15	T	21	18	3	0:00.95
16	T	28	23	5	0:01.24
17	T	21	18	3	0:00.61
18	T	21	18	3	0:00.61
19	T	35	28	7	0:01.56
20	T	35	28	7	0:01.53
21	T	14	13	1	0:00.64
22	T	21	18	3	0:00.96
23	T	21	18	3	0:00.96
24	T	31	25	6	0:01.39
25	T	48	37	11	0:02.09
26	T	14	14	0	0:00.64
27	T	18	16	2	0:00.82
28	T	76	57	19	0:03.31
29	T	21	18	3	0:00.94
30	T	24	20	4	0:01.08
31	T	18	16	2	0:00.82
32	T	14	13	1	0:00.64
33	T	14	13	1	0:00.64
34	T	18	16	2	0:00.81
35	T	31	25	6	0:01.39
36	T	28	23	5	0:01.24
37	T	41	32	9	0:01.83
38	T	24	20	4	0:01.08
39	T	35	28	7	0:01.55
40	T	28	23	5	0:01.26

Id	Result	#total	#true	#false	Execution time (min:sec)
41	T	38	30	8	0:01.66
42	T	21	18	3	0:01.38
43	T	14	13	1	0:00.64
44	T	14	14	0	0:00.64
45	T	21	18	3	0:00.96
46	T	79	59	20	0:03.42
47	T	31	25	6	0:01.40
48	T	35	28	7	0:01.54
49	T	31	25	6	0:01.38
50	T	18	16	2	0:00.82
51	T	14	14	0	0:00.64
52	T	28	23	5	0:01.24
53	T	24	20	4	0:01.09
54	T	28	23	5	0:01.25
55	T	28	23	5	0:01.24
56	T	14	14	0	0:00.64
57	T	14	14	0	0:00.64
58	T	31	25	6	0:01.40
59	T	21	18	3	0:00.93
60	T	21	18	3	0:00.94
61	T	45	35	10	0:01.97
62	T	31	25	6	0:01.37
63	T	31	25	6	0:01.38
64	T	41	32	9	0:01.79
65	T	28	23	5	0:01.23
66	T	18	16	2	0:00.81
67	T	21	18	3	0:00.94
68	T	14	14	0	0:00.75
69	T	28	23	5	0:01.25
70	T	21	18	3	0:00.96
71	T	14	13	1	0:00.64
72	T	28	23	5	0:01.27
73	T	24	20	4	0:01.09
74	T	14	13	1	0:00.64
75	T	48	37	11	0:02.11
76	T	35	28	7	0:02.23
77	T	18	16	2	0:00.81
78	T	24	20	4	0:01.08
79	T	14	14	0	0:00.64
80	T	28	23	5	0:01.24
81	T	18	16	2	0:00.82
82	T	38	30	8	0:01.69
83	T	48	37	11	0:02.12
84	T	35	28	7	0:01.58
85	T	52	40	12	0:02.27
86	T	38	30	8	0:01.68
87	T	31	25	6	0:01.38
88	T	18	16	2	0:00.81

**Id**	**Result**	**#total**	**#true**	**#false**	**Execution time (min:sec)**
89	T	14	13	1	0:00.64
90	T	21	18	3	0:00.94
91	T	24	20	4	0:01.08
92	T	18	16	2	0:00.81
93	T	14	13	1	0:00.65
94	T	14	13	1	0:00.65
95	T	18	16	2	0:00.81
96	T	18	16	2	0:00.82
97	T	31	25	6	0:01.35
98	T	24	20	4	0:01.07
99	T	14	14	0	0:00.64
100	T	14	13	1	0:00.64
101	T	38	30	8	0:01.66
102	T	41	32	9	0:01.80
103	T	14	13	1	0:00.65
104	T	14	13	1	0:00.64
105	T	14	13	1	0:00.64
106	T	18	16	2	0:00.80
107	T	24	20	4	0:01.08
108	T	31	25	6	0:01.39
109	T	21	18	3	0:00.95
110	T	18	16	2	0:00.81
111	T	18	16	2	0:00.81
112	T	21	18	3	0:00.94
113	T	24	20	4	0:01.07
114	T	31	25	6	0:01.39
115	T	48	37	11	0:02.10
116	T	18	16	2	0:00.81
117	T	14	13	1	0:00.64
118	T	24	20	4	0:01.07
119	T	58	44	14	0:02.57
120	T	28	23	5	0:01.24
121	T	18	16	2	0:00.81
122	T	31	25	6	0:01.38
123	T	52	40	12	0:02.28
124	T	24	20	4	0:01.05
125	T	24	20	4	0:01.08
126	T	62	47	15	0:02.70
127	T	18	16	2	0:00.82
128	T	28	23	5	0:01.24
129	T	31	25	6	0:01.38
130	T	31	25	6	0:01.38
131	T	18	16	2	0:00.82
132	T	14	13	1	0:00.64
133	T	31	25	6	0:01.35
134	T	48	37	11	0:02.11
135	T	35	28	7	0:02.00
136	T	21	18	3	0:00.94

Id	Result	#total	#true	#false	Execution time (min:sec)
137	T	38	30	8	0:01.67
138	T	52	40	12	0:02.26
139	T	14	13	1	0:00.66
140	T	14	13	1	0:00.64
141	T	14	13	1	0:00.64
142	T	14	14	0	0:00.64
143	T	24	20	4	0:01.09
144	T	24	20	4	0:01.08
145	T	31	25	6	0:01.37
146	T	41	32	9	0:01.80
147	T	45	35	10	0:01.98
148	T	38	30	8	0:01.68
149	T	14	13	1	0:00.64
150	T	14	13	1	0:00.64
151	T	14	13	1	0:00.65
152	T	41	32	9	0:01.83
153	T	45	35	10	0:01.98
154	T	14	14	0	0:00.64
155	T	89	66	23	0:03.89
156	T	31	25	6	0:01.37
157	T	21	18	3	0:00.94
158	T	14	14	0	0:00.69
159	T	31	25	6	0:01.50
160	T	14	14	0	0:00.64
161	T	14	14	0	0:00.64
162	T	18	16	2	0:00.82
163	T	38	30	8	0:01.69
164	T	14	13	1	0:00.68
165	T	24	20	4	0:01.08
166	T	1000	819	181	0:40.45
167	T	18	16	2	0:00.80
168	T	18	16	2	0:00.80
169	T	14	14	0	0:01.03
170	T	14	13	1	0:00.64
171	T	21	18	3	0:00.95
172	T	21	18	3	0:00.94
173	T	14	14	0	0:00.65
174	T	14	14	0	0:00.64
175	T	14	14	0	0:00.63
176	T	41	32	9	0:01.81
177	T	21	18	3	0:00.95
178	T	14	14	0	0:00.63
179	T	14	14	0	0:00.63
180	T	14	14	0	0:00.64
181	T	14	13	1	0:00.64
182	T	14	13	1	0:00.63
183	T	14	13	1	0:00.65
184	T	14	13	1	0:00.65

Id	Result	#total	#true	#false	Execution time (min:sec)
185	T	35	28	7	0:01.55
186	T	14	13	1	0:00.65
187	T	14	13	1	0:00.66
188	T	14	13	1	0:00.63
189	T	24	20	4	0:01.08
190	T	24	20	4	0:01.09
191	T	14	13	1	0:00.64
192	T	35	28	7	0:01.55
193	T	14	14	0	0:00.64
194	T	24	20	4	0:01.07
195	T	41	32	9	0:01.81
196	T	14	13	1	0:00.65
197	T	14	13	1	0:00.64
198	T	18	16	2	0:00.81
199	T	24	20	4	0:01.06
200	T	35	28	7	0:01.53
201	T	21	18	3	0:00.95
202	T	24	20	4	0:01.05
203	T	24	20	4	0:01.06
204	T	41	32	9	0:01.79
205	T	58	44	14	0:02.53
206	T	65	49	16	0:03.72
207	T	31	25	6	0:01.37
208	T	38	30	8	0:01.69
209	T	72	54	18	0:03.16
210	T	28	23	5	0:01.24
211	T	14	13	1	0:00.64
212	T	28	23	5	0:01.25
213	T	89	66	23	0:03.86
214	T	18	16	2	0:00.82
215	T	24	20	4	0:01.08
216	T	14	13	1	0:00.64
217	T	14	13	1	0:00.64
218	T	14	13	1	0:00.65
219	T	14	13	1	0:00.65
220	T	14	13	1	0:00.65
221	T	18	16	2	0:00.82
222	T	24	20	4	0:01.08
223	T	14	13	1	0:00.63
224	T	18	16	2	0:00.80
225	T	18	16	2	0:00.80
226	T	38	30	8	0:01.67
227	T	28	23	5	0:01.27
228	T	14	13	1	0:00.64
229	T	35	28	7	0:01.55
230	T	21	18	3	0:00.93
231	T	24	20	4	0:01.09
232	T	28	23	5	0:01.25

Id	Result	#total	#true	#false	Execution time (min:sec)
233	T	41	32	9	0:01.79
234	T	18	16	2	0:00.82
235	T	18	16	2	0:00.82
236	T	21	18	3	0:00.95
237	T	58	44	14	0:02.55
238	T	14	13	1	0:00.66
239	T	14	13	1	0:00.66
240	T	21	18	3	0:00.95
241	T	18	16	2	0:00.81
242	T	72	54	18	0:03.15
243	T	14	14	0	0:00.64
244	T	14	13	1	0:00.64
245	T	14	13	1	0:00.64
246	T	35	28	7	0:01.53
247	T	14	13	1	0:00.64
248	T	14	13	1	0:00.64
249	T	31	25	6	0:01.39
250	T	62	47	15	0:02.68
251	T	14	13	1	0:00.66
252	T	14	13	1	0:00.64
253	T	14	13	1	0:00.64
254	T	14	14	0	0:00.64
255	T	21	18	3	0:00.96
256	T	14	14	0	0:00.64
257	T	14	14	0	0:00.64
258	T	14	13	1	0:00.63
259	T	14	13	1	0:00.63
260	T	28	23	5	0:01.27
261	T	55	42	13	0:02.40
262	T	76	57	19	0:03.32
263	T	14	13	1	0:00.66
264	T	21	18	3	0:00.95
265	T	58	44	14	0:02.53
266	T	35	28	7	0:01.55
267	T	21	18	3	0:00.93
268	T	41	32	9	0:01.80
269	T	14	13	1	0:00.64
270	T	14	13	1	0:00.64
271	T	38	30	8	0:01.64
272	T	35	28	7	0:01.57
273	T	38	30	8	0:01.67
274	T	14	13	1	0:00.65
275	T	14	13	1	0:00.64
276	T	21	18	3	0:00.95
277	T	31	25	6	0:01.38
278	T	31	25	6	0:01.37
279	T	14	14	0	0:00.74
280	T	14	14	0	0:00.64

Id	Result	#total	#true	#false	Execution time (min:sec)
281	T	14	14	0	0:00.64
282	T	28	23	5	0:01.78
283	T	65	49	16	0:02.84
284	T	31	25	6	0:01.37
285	T	21	18	3	0:00.93
286	F	85	57	28	0:03.75
287	T	28	23	5	0:01.23
288	T	21	18	3	0:00.95
289	T	28	23	5	0:01.23
290	T	21	18	3	0:00.96
291	T	14	13	1	0:00.64
292	T	24	20	4	0:01.06
293	T	24	20	4	0:01.06
294	T	28	23	5	0:01.26
295	T	21	18	3	0:00.96
296	T	48	37	11	0:02.10
297	T	14	13	1	0:00.63
298	T	35	28	7	0:01.55
299	T	18	16	2	0:00.82
300	T	45	35	10	0:01.99
301	T	14	13	1	0:00.63
302	T	35	28	7	0:01.56
303	T	35	28	7	0:01.55
304	T	14	13	1	0:00.64
305	T	35	28	7	0:01.56
306	T	24	20	4	0:01.08
307	T	14	13	1	0:00.64
308	T	21	18	3	0:00.95
309	T	18	16	2	0:00.82
310	T	14	13	1	0:00.65
311	T	14	13	1	0:00.64
312	T	62	47	15	0:02.72
313	T	21	18	3	0:00.94
314	T	14	13	1	0:00.64
315	T	14	13	1	0:00.64
316	T	28	23	5	0:01.25
317	T	14	14	0	0:00.64
318	T	31	25	6	0:01.37
319	T	14	13	1	0:00.64
320	T	18	16	2	0:00.80
321	T	28	23	5	0:01.27
322	T	38	30	8	0:01.67
323	T	14	13	1	0:00.64
324	T	14	13	1	0:00.65
325	T	14	13	1	0:00.64
326	T	14	13	1	0:00.64
327	T	14	13	1	0:00.64
328	T	14	14	0	0:00.64

Id	Result	#total	#true	#false	Execution time (min:sec)
329	T	18	16	2	0:00.82
330	T	28	23	5	0:01.25
331	T	24	20	4	0:01.07
332	T	14	13	1	0:00.65
333	T	1000	819	181	0:41.40
334	T	14	13	1	0:00.64
335	T	14	14	0	0:00.65
336	T	14	14	0	0:00.64
337	T	24	20	4	0:01.07
338	T	14	13	1	0:00.63
339	T	38	30	8	0:01.68
340	T	62	47	15	0:02.67
341	T	28	23	5	0:01.25
342	T	41	32	9	0:01.79
343	T	14	13	1	0:00.64
344	T	21	18	3	0:01.15
345	T	28	23	5	0:01.24
346	T	31	25	6	0:01.37
347	T	24	20	4	0:01.07
348	T	21	18	3	0:00.96
349	T	35	28	7	0:01.54
350	T	14	13	1	0:00.64
351	T	14	13	1	0:00.64
352	T	14	13	1	0:00.64
353	T	14	14	0	0:00.64
354	T	65	49	16	0:02.88
355	T	28	23	5	0:01.24
356	T	18	16	2	0:00.82
357	T	14	13	1	0:00.65
358	T	14	13	1	0:00.64
359	T	18	16	2	0:00.81
360	T	48	37	11	0:02.13
361	T	21	18	3	0:00.94
362	T	18	16	2	0:01.12
363	T	55	42	13	0:02.43
364	T	24	20	4	0:01.07
365	T	14	13	1	0:00.64
366	T	14	13	1	0:00.64
367	T	21	18	3	0:00.94
368	T	21	18	3	0:00.94
369	T	28	23	5	0:01.26
370	T	21	18	3	0:00.95
371	T	31	25	6	0:01.36
372	T	62	47	15	0:02.73
373	T	41	32	9	0:01.81
374	T	24	20	4	0:01.09
375	T	28	23	5	0:01.25
376	T	45	35	10	0:01.98

Id	Result	#total	#true	#false	Execution time (min:sec)
377	T	21	18	3	0:00.94
378	T	18	16	2	0:00.82
379	T	28	23	5	0:01.25
380	T	65	49	16	0:02.79
381	T	14	13	1	0:00.64
382	T	24	20	4	0:01.08
383	T	14	13	1	0:00.64
384	T	28	23	5	0:01.25
385	T	14	13	1	0:00.65
386	T	21	18	3	0:01.51
387	T	14	14	0	0:00.64
388	T	14	13	1	0:00.63
389	T	14	13	1	0:00.63
390	T	18	16	2	0:00.83
391	T	14	13	1	0:00.64
392	T	14	13	1	0:00.64
393	T	35	28	7	0:01.55
394	T	14	13	1	0:00.64
395	T	14	13	1	0:00.65
396	T	14	14	0	0:00.64
397	T	28	23	5	0:01.25
398	T	24	20	4	0:01.06
399	T	35	28	7	0:01.56
400	T	24	20	4	0:01.07
401	T	14	13	1	0:00.64
402	T	14	13	1	0:00.65
403	T	24	20	4	0:01.08
404	T	14	13	1	0:00.65
405	T	14	13	1	0:00.64
406	T	14	13	1	0:00.64
407	T	38	30	8	0:01.68
408	T	14	13	1	0:00.65
409	T	14	13	1	0:00.64
410	T	14	13	1	0:00.64
411	T	21	18	3	0:00.93
412	T	18	16	2	0:00.82
413	T	18	16	2	0:00.82
414	T	21	18	3	0:00.97
415	T	14	13	1	0:00.63
416	T	28	23	5	0:01.23
417	T	21	18	3	0:00.96
418	T	14	14	0	0:00.64
419	T	24	20	4	0:01.08
420	T	38	30	8	0:01.68
421	T	14	13	1	0:00.64
422	T	14	13	1	0:00.63
423	T	28	23	5	0:01.22
424	T	24	20	4	0:01.10

Id	Result	#total	#true	#false	Execution time (min:sec)
425	T	24	20	4	0:01.07
426	T	21	18	3	0:00.94
427	T	55	42	13	0:02.40
428	T	38	30	8	0:01.68
429	T	24	20	4	0:01.07
430	T	24	20	4	0:01.08
431	T	38	30	8	0:01.68
432	T	14	14	0	0:00.64
433	T	58	44	14	0:02.50
434	T	38	30	8	0:01.70
435	T	28	23	5	0:01.25
436	T	45	35	10	0:01.96
437	T	24	20	4	0:01.07
438	T	35	28	7	0:01.53
439	T	45	35	10	0:02.00
440	T	14	14	0	0:00.64
441	T	31	25	6	0:01.36
442	T	38	30	8	0:01.68
443	T	21	18	3	0:00.96
444	T	31	25	6	0:01.39
445	T	21	18	3	0:00.95
446	T	24	20	4	0:01.08
447	T	38	30	8	0:01.70
448	T	14	13	1	0:00.65
449	T	14	13	1	0:00.64
450	T	31	25	6	0:01.38
451	T	24	20	4	0:01.07
452	T	35	28	7	0:01.56
453	T	14	13	1	0:00.63
454	T	21	18	3	0:00.95
455	T	62	47	15	0:02.68
456	T	14	13	1	0:00.64
457	T	24	20	4	0:01.07
458	T	24	20	4	0:01.06
459	T	28	23	5	0:01.25
460	T	18	16	2	0:00.81
461	T	14	13	1	0:00.64
462	T	28	23	5	0:01.22
463	T	31	25	6	0:01.35
464	T	24	20	4	0:01.08
465	T	14	13	1	0:00.65
466	T	14	13	1	0:00.65
467	T	82	61	21	0:03.55
468	T	24	20	4	0:01.07
469	T	28	23	5	0:01.24
470	T	55	42	13	0:02.41
471	T	58	44	14	0:02.54
472	T	55	42	13	0:02.43

Id	Result	#total	#true	#false	Execution time (min:sec)
473	T	21	18	3	0:00.95
474	T	18	16	2	0:00.80
475	T	21	18	3	0:00.94
476	T	14	13	1	0:00.64
477	T	41	32	9	0:01.81
478	T	18	16	2	0:00.81
479	T	35	28	7	0:01.56
480	T	14	13	1	0:00.65
481	T	69	52	17	0:03.04
482	T	14	13	1	0:00.65
483	T	14	13	1	0:00.65
484	T	14	13	1	0:00.65
485	T	79	59	20	0:03.46
486	T	41	32	9	0:01.81
487	T	28	23	5	0:01.24
488	T	14	13	1	0:00.64
489	T	24	20	4	0:01.08
490	T	89	66	23	0:03.86
491	T	21	18	3	0:00.94
492	T	58	44	14	0:02.53
493	T	48	37	11	0:02.13
494	T	41	32	9	0:01.81
495	T	24	20	4	0:01.07
496	T	21	18	3	0:00.95
497	T	14	13	1	0:00.64
498	T	18	16	2	0:00.82
499	T	18	16	2	0:00.83
500	T	52	40	12	0:02.27
