# Supplementary material for: Automatic validation of computational models using pseudo-3D spatio-temporal model checking
Source: BMC Syst Biol. 2014 Dec 2;8:124. doi: 10.1186/s12918-014-0124-0 (PMC4272535; doi:10.1186/s12918-014-0124-0)
Supplement: Additional file 11 — Model checking results for the chemotaxis case study. The results obtained for each model checker execution and PBLSTL statement considering the chemotaxis case study. For statistical analysis of the results the pdf file can be converted to a Microsoft Word document, using the online converter (http://www.pdfonline.com/pdf-to-word-converter), from which the table of results can be directly copied in a spreadsheet-like software such as Microsoft Excel (or LibreOffice Calc). In case you would like to use statistical software such as R you can save the results from Excel/Calc in csv format and then import them into R. [file 12918_2014_124_MOESM11_ESM.pdf]

Model checking results for the chemotaxis case study

For the convenience of the reader the set of PBLSTL statements considered for the chemotaxis case study will be restated below:

$$P >= 0.9\ [F\ [0,200]\ (count(filter(clusters, (centroidX < add(703.5, sqrt(subtract(2500, power(subtract(678.5, centroidY), 2))))) \wedge (centroidX > subtract(703.5, sqrt(subtract(2500, power(subtract(678.5, centroidY), 2))))) \wedge (centroidY < add(678.5, 50)) \wedge (centroidY > subtract(678.5, 50)))) >= 1)]. \tag{9}$$

$$P >= 0.8\ [F\ [0,200]\ (d(avg(clusters, clusteredness)) > 0) \wedge (X\ (F\ [0,200]\ (d(avg(clusters, clusteredness)) > 0) \wedge (X\ (F\ [0,200]\ (d(avg(clusters, clusteredness)) > 0) \wedge (X\ (F\ [0,200]\ (d(avg(clusters, clusteredness)) > 0) \wedge (X\ (F\ [0,200]\ (d(avg(clusters, clusteredness)) > 0)))))))))]. \tag{10}$$

$$P <= 0.05\ [F\ [0,50]\ (\{avgClusterednessClusters\} < 0.5)]. \tag{11}$$

$$P > 0.75\ [F\ [0,200]\ (count(clusters) <= 5 \wedge G\ [0,200]\ (count(clusters) <= 5))]. \tag{12}$$

$$P >= 0.6\ [F\ [0,200]\ (count(filter(clusters, (circleMeasure > triangleMeasure) \wedge (circleMeasure > rectangleMeasure))) > 0)]. \tag{13}$$

$$
\begin{aligned}
P < 0.1\ [\neg\ ((F\ [0,200]\ ((count(clusters) > 0) \wedge \\
&(d(avg(clusters, density)) > 0) \wedge \\
&F\ [0,200]\ ((d(avg(clusters, density)) < 0)))) \vee \\
&(F\ [0,200]\ ((count(clusters) > 0) \wedge \\
&(d(avg(clusters, density)) < 0) \wedge \\
&F\ [0,200]\ ((d(avg(clusters, density)) > 0)))))].
\end{aligned}
\tag{14}
$$

Each table describes the results corresponding to one of the PBLSTL statements. The first column of each row represents the identifier of the model checking execution. The second column represents the evaluation result (T = true, F = false) of the PBLSTL statement for that particular model checker execution. The number of STML files against which the PBLSTL statement was executed, respectively how many of them evaluated true and how many evaluated false is provided in columns three, four and five. Finally column six presents the execution time (minutes:seconds format) corresponding to each model checker run. All executions of the model checker employed the frequentist statistical model checking approach with both probability of type I and type II errors equal to 5%. The path to an extra evaluation program was made available via a command line parameter such that Mudi could generate simulations (and implicitly STML files) on demand; the extra evaluation time was set to 60 (minutes) and was never exceeded in our experiments. In order to keep the chemotaxis and the phase variation model checking results comparable the extra evaluation time should not be considered. Thus the execution times reported in the table below have been recorded during a repeated evaluation of the PBLSTL statements when sufficient STML files were available and no extra evaluation was required.

Table 1: Model checking results corresponding to logic property 9

**Id**	**Result**	**#total**	**#true**	**#false**	**Execution time (min:sec)**
1	T	28	28	0	0:21.99
2	T	28	28	0	0:22.11
3	T	28	28	0	0:22.08
4	T	28	28	0	0:22.06
5	T	28	28	0	0:22.20
6	T	28	28	0	0:22.00
7	T	28	28	0	0:21.99
8	T	28	28	0	0:22.17
9	T	28	28	0	0:22.09
10	T	28	28	0	0:22.19
11	T	28	28	0	0:22.06
12	T	28	28	0	0:21.95
13	T	28	28	0	0:22.04
14	T	28	28	0	0:21.94
15	T	28	28	0	0:22.02
16	T	28	28	0	0:22.03
17	T	28	28	0	0:21.94

Id	Result	#total	#true	#false	Execution time (min:sec)
18	T	28	28	0	0:22.08
19	T	28	28	0	0:22.05
20	T	28	28	0	0:22.05
21	T	28	28	0	0:22.05
22	T	28	28	0	0:22.25
23	T	28	28	0	0:22.05
24	T	28	28	0	0:22.04
25	T	28	28	0	0:22.07
26	T	28	28	0	0:22.06
27	T	28	28	0	0:21.98
28	T	28	28	0	0:22.18
29	T	28	28	0	0:21.92
30	T	28	28	0	0:22.18
31	T	28	28	0	0:22.14
32	T	28	28	0	0:22.14
33	T	28	28	0	0:22.03
34	T	28	28	0	0:21.98
35	T	28	28	0	0:22.20
36	T	28	28	0	0:22.28
37	T	28	28	0	0:22.03
38	T	28	28	0	0:22.00
39	T	28	28	0	0:22.06
40	T	28	28	0	0:22.13
41	T	28	28	0	0:22.04
42	T	28	28	0	0:21.93
43	T	28	28	0	0:22.05
44	T	28	28	0	0:22.03
45	T	28	28	0	0:22.05
46	T	28	28	0	0:22.10
47	T	28	28	0	0:22.07
48	T	28	28	0	0:22.08
49	T	28	28	0	0:22.03
50	T	28	28	0	0:21.94
51	T	28	28	0	0:22.02
52	T	28	28	0	0:22.03
53	T	28	28	0	0:22.08
54	T	28	28	0	0:22.18
55	T	28	28	0	0:22.11
56	T	28	28	0	0:22.09
57	T	28	28	0	0:22.10
58	T	28	28	0	0:22.30
59	T	28	28	0	0:21.97
60	T	28	28	0	0:22.10
61	T	28	28	0	0:22.03
62	T	28	28	0	0:21.91
63	T	28	28	0	0:22.03
64	T	28	28	0	0:22.23
65	T	28	28	0	0:21.94

Id	Result	#total	#true	#false	Execution time (min:sec)
66	T	28	28	0	0:22.05
67	T	28	28	0	0:22.17
68	T	28	28	0	0:22.03
69	T	28	28	0	0:21.92
70	T	28	28	0	0:22.09
71	T	28	28	0	0:21.95
72	T	28	28	0	0:22.01
73	T	28	28	0	0:21.89
74	T	28	28	0	0:21.92
75	T	28	28	0	0:21.90
76	T	28	28	0	0:22.07
77	T	28	28	0	0:22.14
78	T	28	28	0	0:22.03
79	T	28	28	0	0:22.13
80	T	28	28	0	0:21.94
81	T	28	28	0	0:22.01
82	T	28	28	0	0:22.26
83	T	28	28	0	0:22.13
84	T	28	28	0	0:21.92
85	T	28	28	0	0:21.98
86	T	28	28	0	0:21.91
87	T	28	28	0	0:21.98
88	T	28	28	0	0:21.86
89	T	28	28	0	0:21.94
90	T	28	28	0	0:22.13
91	T	28	28	0	0:22.02
92	T	28	28	0	0:22.04
93	T	28	28	0	0:22.23
94	T	28	28	0	0:22.04
95	T	28	28	0	0:22.15
96	T	28	28	0	0:21.98
97	T	28	28	0	0:21.88
98	T	28	28	0	0:22.02
99	T	28	28	0	0:22.07
100	T	28	28	0	0:21.96
101	T	28	28	0	0:22.00
102	T	28	28	0	0:21.99
103	T	28	28	0	0:22.06
104	T	28	28	0	0:22.03
105	T	28	28	0	0:22.01
106	T	28	28	0	0:22.04
107	T	28	28	0	0:21.94
108	T	28	28	0	0:21.99
109	T	28	28	0	0:22.07
110	T	28	28	0	0:21.98
111	T	28	28	0	0:22.00
112	T	28	28	0	0:21.91
113	T	28	28	0	0:22.04

Id	Result	#total	#true	#false	Execution time (min:sec)
114	T	28	28	0	0:21.95
115	T	28	28	0	0:22.08
116	T	28	28	0	0:21.98
117	T	28	28	0	0:22.08
118	T	28	28	0	0:23.07
119	T	28	28	0	0:22.02
120	T	28	28	0	0:21.93
121	T	28	28	0	0:22.11
122	T	28	28	0	0:21.99
123	T	28	28	0	0:22.05
124	T	28	28	0	0:22.02
125	T	28	28	0	0:22.07
126	T	28	28	0	0:22.10
127	T	28	28	0	0:21.99
128	T	28	28	0	0:22.05
129	T	28	28	0	0:22.03
130	T	28	28	0	0:22.12
131	T	28	28	0	0:21.99
132	T	28	28	0	0:21.97
133	T	28	28	0	0:22.18
134	T	28	28	0	0:22.17
135	T	28	28	0	0:21.94
136	T	28	28	0	0:22.03
137	T	28	28	0	0:22.15
138	T	28	28	0	0:22.07
139	T	28	28	0	0:22.08
140	T	28	28	0	0:21.96
141	T	28	28	0	0:21.89
142	T	28	28	0	0:22.12
143	T	28	28	0	0:22.05
144	T	28	28	0	0:21.97
145	T	28	28	0	0:22.09
146	T	28	28	0	0:21.97
147	T	28	28	0	0:22.09
148	T	28	28	0	0:22.09
149	T	28	28	0	0:22.00
150	T	28	28	0	0:22.02
151	T	28	28	0	0:22.17
152	T	28	28	0	0:22.09
153	T	28	28	0	0:21.99
154	T	28	28	0	0:22.01
155	T	28	28	0	0:22.11
156	T	28	28	0	0:21.93
157	T	28	28	0	0:21.97
158	T	28	28	0	0:22.25
159	T	28	28	0	0:22.10
160	T	28	28	0	0:21.98
161	T	28	28	0	0:22.05

Id	Result	#total	#true	#false	Execution time (min:sec)
162	T	28	28	0	0:21.94
163	T	28	28	0	0:22.02
164	T	28	28	0	0:22.00
165	T	28	28	0	0:22.08
166	T	28	28	0	0:22.34
167	T	28	28	0	0:21.98
168	T	28	28	0	0:22.00
169	T	28	28	0	0:22.08
170	T	28	28	0	0:23.50
171	T	28	28	0	0:22.10
172	T	28	28	0	0:21.96
173	T	28	28	0	0:22.08
174	T	28	28	0	0:22.11
175	T	28	28	0	0:22.09
176	T	28	28	0	0:21.87
177	T	28	28	0	0:21.98
178	T	28	28	0	0:22.16
179	T	28	28	0	0:21.95
180	T	28	28	0	0:21.99
181	T	28	28	0	0:22.07
182	T	28	28	0	0:21.94
183	T	28	28	0	0:21.88
184	T	28	28	0	0:22.10
185	T	28	28	0	0:22.02
186	T	28	28	0	0:22.01
187	T	28	28	0	0:21.94
188	T	28	28	0	0:22.22
189	T	28	28	0	0:21.97
190	T	28	28	0	0:21.95
191	T	28	28	0	0:22.11
192	T	28	28	0	0:22.13
193	T	28	28	0	0:22.12
194	T	28	28	0	0:22.04
195	T	28	28	0	0:21.97
196	T	28	28	0	0:22.03
197	T	28	28	0	0:22.03
198	T	28	28	0	0:21.97
199	T	28	28	0	0:22.09
200	T	28	28	0	0:22.04
201	T	28	28	0	0:21.98
202	T	28	28	0	0:22.10
203	T	28	28	0	0:22.83
204	T	28	28	0	0:22.11
205	T	28	28	0	0:21.95
206	T	28	28	0	0:22.04
207	T	28	28	0	0:22.08
208	T	28	28	0	0:22.06
209	T	28	28	0	0:22.11

**Id**	**Result**	**#total**	**#true**	**#false**	**Execution time (min:sec)**
210	T	28	28	0	0:21.97
211	T	28	28	0	0:22.06
212	T	28	28	0	0:21.94
213	T	28	28	0	0:21.99
214	T	28	28	0	0:22.06
215	T	28	28	0	0:22.13
216	T	28	28	0	0:22.08
217	T	28	28	0	0:22.03
218	T	28	28	0	0:22.11
219	T	28	28	0	0:22.00
220	T	28	28	0	0:22.13
221	T	28	28	0	0:22.07
222	T	28	28	0	0:22.08
223	T	28	28	0	0:21.90
224	T	28	28	0	0:21.93
225	T	28	28	0	0:21.96
226	T	28	28	0	0:22.00
227	T	28	28	0	0:22.01
228	T	28	28	0	0:22.03
229	T	28	28	0	0:22.04
230	T	28	28	0	0:21.94
231	T	28	28	0	0:22.01
232	T	28	28	0	0:22.00
233	T	28	28	0	0:21.98
234	T	28	28	0	0:22.13
235	T	28	28	0	0:21.99
236	T	28	28	0	0:21.85
237	T	28	28	0	0:22.06
238	T	28	28	0	0:21.98
239	T	28	28	0	0:22.08
240	T	28	28	0	0:22.04
241	T	28	28	0	0:22.13
242	T	28	28	0	0:22.05
243	T	28	28	0	0:21.93
244	T	28	28	0	0:21.97
245	T	28	28	0	0:22.11
246	T	28	28	0	0:22.20
247	T	28	28	0	0:22.02
248	T	28	28	0	0:22.13
249	T	28	28	0	0:22.03
250	T	28	28	0	0:21.94
251	T	28	28	0	0:22.04
252	T	28	28	0	0:21.88
253	T	28	28	0	0:22.09
254	T	28	28	0	0:21.91
255	T	28	28	0	0:22.07
256	T	28	28	0	0:21.98
257	T	28	28	0	0:22.03

Id	Result	#total	#true	#false	Execution time (min:sec)
258	T	28	28	0	0:21.94
259	T	28	28	0	0:22.05
260	T	28	28	0	0:21.89
261	T	28	28	0	0:22.14
262	T	28	28	0	0:22.81
263	T	28	28	0	0:22.05
264	T	28	28	0	0:22.06
265	T	28	28	0	0:22.06
266	T	28	28	0	0:22.03
267	T	28	28	0	0:21.98
268	T	28	28	0	0:22.15
269	T	28	28	0	0:22.02
270	T	28	28	0	0:21.98
271	T	28	28	0	0:22.02
272	T	28	28	0	0:22.12
273	T	28	28	0	0:22.02
274	T	28	28	0	0:21.87
275	T	28	28	0	0:21.94
276	T	28	28	0	0:22.11
277	T	28	28	0	0:22.05
278	T	28	28	0	0:22.01
279	T	28	28	0	0:21.99
280	T	28	28	0	0:22.11
281	T	28	28	0	0:21.99
282	T	28	28	0	0:22.09
283	T	28	28	0	0:22.11
284	T	28	28	0	0:22.08
285	T	28	28	0	0:22.12
286	T	28	28	0	0:22.03
287	T	28	28	0	0:21.90
288	T	28	28	0	0:21.97
289	T	28	28	0	0:21.99
290	T	28	28	0	0:22.02
291	T	28	28	0	0:21.88
292	T	28	28	0	0:22.14
293	T	28	28	0	0:22.02
294	T	28	28	0	0:22.08
295	T	28	28	0	0:22.04
296	T	28	28	0	0:22.04
297	T	28	28	0	0:22.06
298	T	28	28	0	0:22.04
299	T	28	28	0	0:21.95
300	T	28	28	0	0:22.05
301	T	28	28	0	0:22.04
302	T	28	28	0	0:22.16
303	T	28	28	0	0:22.00
304	T	28	28	0	0:21.99
305	T	28	28	0	0:22.08

Id	Result	#total	#true	#false	Execution time (min:sec)
306	T	28	28	0	0:22.02
307	T	28	28	0	0:21.96
308	T	28	28	0	0:22.05
309	T	28	28	0	0:22.05
310	T	28	28	0	0:22.10
311	T	28	28	0	0:22.03
312	T	28	28	0	0:22.02
313	T	28	28	0	0:22.03
314	T	28	28	0	0:22.05
315	T	28	28	0	0:22.04
316	T	28	28	0	0:22.06
317	T	28	28	0	0:21.98
318	T	28	28	0	0:21.97
319	T	28	28	0	0:21.96
320	T	28	28	0	0:22.00
321	T	28	28	0	0:21.91
322	T	28	28	0	0:21.95
323	T	28	28	0	0:22.02
324	T	28	28	0	0:22.09
325	T	28	28	0	0:22.00
326	T	28	28	0	0:22.26
327	T	28	28	0	0:21.92
328	T	28	28	0	0:21.99
329	T	28	28	0	0:22.01
330	T	28	28	0	0:22.11
331	T	28	28	0	0:22.07
332	T	28	28	0	0:21.99
333	T	28	28	0	0:22.00
334	T	28	28	0	0:22.12
335	T	28	28	0	0:22.11
336	T	28	28	0	0:22.10
337	T	28	28	0	0:22.04
338	T	28	28	0	0:22.06
339	T	28	28	0	0:22.04
340	T	28	28	0	0:22.04
341	T	28	28	0	0:22.02
342	T	28	28	0	0:21.96
343	T	28	28	0	0:22.02
344	T	28	28	0	0:22.15
345	T	28	28	0	0:22.10
346	T	28	28	0	0:21.99
347	T	28	28	0	0:22.09
348	T	28	28	0	0:22.06
349	T	28	28	0	0:22.07
350	T	28	28	0	0:21.93
351	T	28	28	0	0:21.93
352	T	28	28	0	0:22.05
353	T	28	28	0	0:21.96

Id	Result	#total	#true	#false	Execution time (min:sec)
354	T	28	28	0	0:22.14
355	T	28	28	0	0:22.04
356	T	28	28	0	0:21.99
357	T	28	28	0	0:21.99
358	T	28	28	0	0:22.05
359	T	28	28	0	0:22.11
360	T	28	28	0	0:22.02
361	T	28	28	0	0:22.02
362	T	28	28	0	0:22.04
363	T	28	28	0	0:22.06
364	T	28	28	0	0:22.01
365	T	28	28	0	0:21.98
366	T	28	28	0	0:21.92
367	T	28	28	0	0:22.18
368	T	28	28	0	0:21.93
369	T	28	28	0	0:22.02
370	T	28	28	0	0:21.99
371	T	28	28	0	0:22.13
372	T	28	28	0	0:22.19
373	T	28	28	0	0:22.19
374	T	28	28	0	0:22.01
375	T	28	28	0	0:22.11
376	T	28	28	0	0:22.11
377	T	28	28	0	0:22.18
378	T	28	28	0	0:21.98
379	T	28	28	0	0:22.05
380	T	28	28	0	0:22.15
381	T	28	28	0	0:22.06
382	T	28	28	0	0:21.88
383	T	28	28	0	0:22.03
384	T	28	28	0	0:22.11
385	T	28	28	0	0:22.11
386	T	28	28	0	0:22.06
387	T	28	28	0	0:22.09
388	T	28	28	0	0:21.99
389	T	28	28	0	0:21.92
390	T	28	28	0	0:21.99
391	T	28	28	0	0:22.08
392	T	28	28	0	0:22.12
393	T	28	28	0	0:21.98
394	T	28	28	0	0:21.88
395	T	28	28	0	0:22.17
396	T	28	28	0	0:22.03
397	T	28	28	0	0:22.07
398	T	28	28	0	0:22.04
399	T	28	28	0	0:22.03
400	T	28	28	0	0:21.99
401	T	28	28	0	0:22.04

**Id**	**Result**	**#total**	**#true**	**#false**	**Execution time (min:sec)**
402	T	28	28	0	0:22.05
403	T	28	28	0	0:21.92
404	T	28	28	0	0:21.97
405	T	28	28	0	0:21.99
406	T	28	28	0	0:23.09
407	T	28	28	0	0:22.02
408	T	28	28	0	0:21.99
409	T	28	28	0	0:21.94
410	T	28	28	0	0:21.95
411	T	28	28	0	0:22.13
412	T	28	28	0	0:22.14
413	T	28	28	0	0:22.13
414	T	28	28	0	0:21.91
415	T	28	28	0	0:22.12
416	T	28	28	0	0:21.94
417	T	28	28	0	0:21.91
418	T	28	28	0	0:22.04
419	T	28	28	0	0:21.98
420	T	28	28	0	0:22.00
421	T	28	28	0	0:21.95
422	T	28	28	0	0:22.01
423	T	28	28	0	0:21.95
424	T	28	28	0	0:22.07
425	T	28	28	0	0:22.03
426	T	28	28	0	0:21.87
427	T	28	28	0	0:21.99
428	T	28	28	0	0:22.02
429	T	28	28	0	0:21.91
430	T	28	28	0	0:21.98
431	T	28	28	0	0:22.04
432	T	28	28	0	0:21.91
433	T	28	28	0	0:22.04
434	T	28	28	0	0:22.09
435	T	28	28	0	0:22.02
436	T	28	28	0	0:22.11
437	T	28	28	0	0:21.97
438	T	28	28	0	0:21.91
439	T	28	28	0	0:22.00
440	T	28	28	0	0:22.04
441	T	28	28	0	0:22.15
442	T	28	28	0	0:21.92
443	T	28	28	0	0:22.03
444	T	28	28	0	0:22.10
445	T	28	28	0	0:22.06
446	T	28	28	0	0:21.97
447	T	28	28	0	0:21.95
448	T	28	28	0	0:22.02
449	T	28	28	0	0:21.84

Id	Result	#total	#true	#false	Execution time (min:sec)
450	T	28	28	0	0:22.04
451	T	28	28	0	0:22.03
452	T	28	28	0	0:22.06
453	T	28	28	0	0:21.94
454	T	28	28	0	0:22.08
455	T	28	28	0	0:22.02
456	T	28	28	0	0:21.99
457	T	28	28	0	0:21.90
458	T	28	28	0	0:21.99
459	T	28	28	0	0:22.00
460	T	28	28	0	0:22.17
461	T	28	28	0	0:21.94
462	T	28	28	0	0:22.08
463	T	28	28	0	0:22.09
464	T	28	28	0	0:22.03
465	T	28	28	0	0:22.15
466	T	28	28	0	0:22.03
467	T	28	28	0	0:21.93
468	T	28	28	0	0:22.00
469	T	28	28	0	0:21.89
470	T	28	28	0	0:21.98
471	T	28	28	0	0:22.14
472	T	28	28	0	0:22.10
473	T	28	28	0	0:22.02
474	T	28	28	0	0:22.11
475	T	28	28	0	0:22.02
476	T	28	28	0	0:22.05
477	T	28	28	0	0:22.02
478	T	28	28	0	0:22.12
479	T	28	28	0	0:22.05
480	T	28	28	0	0:22.02
481	T	28	28	0	0:22.13
482	T	28	28	0	0:22.00
483	T	28	28	0	0:22.09
484	T	28	28	0	0:21.99
485	T	28	28	0	0:22.00
486	T	28	28	0	0:21.96
487	T	28	28	0	0:22.20
488	T	28	28	0	0:22.10
489	T	28	28	0	0:22.06
490	T	28	28	0	0:22.12
491	T	28	28	0	0:22.09
492	T	28	28	0	0:22.05
493	T	28	28	0	0:22.18
494	T	28	28	0	0:21.91
495	T	28	28	0	0:21.98
496	T	28	28	0	0:21.97
497	T	28	28	0	0:22.09

Id	Result	#total	#true	#false	Execution time (min:sec)
498	T	28	28	0	0:22.04
499	T	28	28	0	0:22.00
500	T	28	28	0	0:22.02

Table 2: Model checking results corresponding to logic property 10

Id	Result	#total	#true	#false	Execution time (min:sec)
1	T	14	14	0	0:11.53
2	T	14	14	0	0:11.57
3	T	14	14	0	0:11.42
4	T	14	14	0	0:11.46
5	T	14	14	0	0:11.41
6	T	14	14	0	0:11.49
7	T	14	14	0	0:11.38
8	T	14	14	0	0:11.40
9	T	14	14	0	0:11.53
10	T	14	14	0	0:11.55
11	T	14	14	0	0:11.46
12	T	14	14	0	0:11.47
13	T	14	14	0	0:11.54
14	T	14	14	0	0:11.55
15	T	14	14	0	0:11.50
16	T	14	14	0	0:11.48
17	T	14	14	0	0:11.54
18	T	14	14	0	0:11.48
19	T	14	14	0	0:11.47
20	T	14	14	0	0:11.41
21	T	14	14	0	0:11.48
22	T	14	14	0	0:11.56
23	T	14	14	0	0:11.51
24	T	14	14	0	0:11.41
25	T	14	14	0	0:11.51
26	T	14	14	0	0:11.49
27	T	14	14	0	0:11.47
28	T	14	14	0	0:11.49
29	T	14	14	0	0:11.53
30	T	14	14	0	0:11.43
31	T	14	14	0	0:11.45
32	T	14	14	0	0:11.46
33	T	14	14	0	0:11.47
34	T	14	14	0	0:11.56
35	T	14	14	0	0:11.52
36	T	14	14	0	0:11.51
37	T	14	14	0	0:11.50
38	T	14	14	0	0:11.55

Id	Result	#total	#true	#false	Execution time (min:sec)
39	T	14	14	0	0:11.59
40	T	14	14	0	0:11.47
41	T	14	14	0	0:11.46
42	T	14	14	0	0:11.47
43	T	14	14	0	0:11.47
44	T	14	14	0	0:11.45
45	T	14	14	0	0:11.48
46	T	14	14	0	0:11.44
47	T	14	14	0	0:11.60
48	T	14	14	0	0:11.57
49	T	14	14	0	0:11.53
50	T	14	14	0	0:11.43
51	T	14	14	0	0:11.38
52	T	14	14	0	0:11.47
53	T	14	14	0	0:11.44
54	T	14	14	0	0:11.45
55	T	14	14	0	0:11.51
56	T	14	14	0	0:11.43
57	T	14	14	0	0:11.42
58	T	14	14	0	0:11.49
59	T	14	14	0	0:11.47
60	T	14	14	0	0:11.47
61	T	14	14	0	0:11.45
62	T	14	14	0	0:11.43
63	T	14	14	0	0:11.49
64	T	14	14	0	0:11.43
65	T	14	14	0	0:11.47
66	T	14	14	0	0:11.37
67	T	14	14	0	0:11.45
68	T	14	14	0	0:11.45
69	T	14	14	0	0:11.46
70	T	14	14	0	0:11.49
71	T	14	14	0	0:11.52
72	T	14	14	0	0:11.48
73	T	14	14	0	0:11.44
74	T	14	14	0	0:11.42
75	T	14	14	0	0:11.51
76	T	14	14	0	0:11.47
77	T	14	14	0	0:11.52
78	T	14	14	0	0:11.48
79	T	14	14	0	0:11.45
80	T	14	14	0	0:11.50
81	T	14	14	0	0:11.46
82	T	14	14	0	0:11.48
83	T	14	14	0	0:11.48
84	T	14	14	0	0:11.47
85	T	14	14	0	0:11.47
86	T	14	14	0	0:11.51

Id	Result	#total	#true	#false	Execution time (min:sec)
87	T	14	14	0	0:11.45
88	T	14	14	0	0:11.44
89	T	14	14	0	0:11.46
90	T	14	14	0	0:11.50
91	T	14	14	0	0:11.38
92	T	14	14	0	0:11.51
93	T	14	14	0	0:11.45
94	T	14	14	0	0:11.45
95	T	14	14	0	0:11.46
96	T	14	14	0	0:11.55
97	T	14	14	0	0:11.47
98	T	14	14	0	0:11.51
99	T	14	14	0	0:11.43
100	T	14	14	0	0:11.53
101	T	14	14	0	0:11.44
102	T	14	14	0	0:11.44
103	T	14	14	0	0:11.44
104	T	14	14	0	0:11.50
105	T	14	14	0	0:11.55
106	T	14	14	0	0:12.33
107	T	14	14	0	0:11.54
108	T	14	14	0	0:11.56
109	T	14	14	0	0:11.47
110	T	14	14	0	0:11.44
111	T	14	14	0	0:11.50
112	T	14	14	0	0:11.56
113	T	14	14	0	0:11.43
114	T	14	14	0	0:11.49
115	T	14	14	0	0:11.45
116	T	14	14	0	0:11.45
117	T	14	14	0	0:11.57
118	T	14	14	0	0:11.45
119	T	14	14	0	0:11.43
120	T	14	14	0	0:11.53
121	T	14	14	0	0:11.44
122	T	14	14	0	0:11.43
123	T	14	14	0	0:11.50
124	T	14	14	0	0:11.51
125	T	14	14	0	0:11.47
126	T	14	14	0	0:11.56
127	T	14	14	0	0:11.51
128	T	14	14	0	0:11.43
129	T	14	14	0	0:11.51
130	T	14	14	0	0:11.47
131	T	14	14	0	0:11.47
132	T	14	14	0	0:11.42
133	T	14	14	0	0:11.44
134	T	14	14	0	0:11.49

Id	Result	#total	#true	#false	Execution time (min:sec)
135	T	14	14	0	0:11.41
136	T	14	14	0	0:11.55
137	T	14	14	0	0:11.43
138	T	14	14	0	0:11.52
139	T	14	14	0	0:11.42
140	T	14	14	0	0:11.51
141	T	14	14	0	0:11.53
142	T	14	14	0	0:11.46
143	T	14	14	0	0:11.51
144	T	14	14	0	0:11.44
145	T	14	14	0	0:11.53
146	T	14	14	0	0:11.49
147	T	14	14	0	0:11.45
148	T	14	14	0	0:12.37
149	T	14	14	0	0:11.47
150	T	14	14	0	0:11.50
151	T	14	14	0	0:11.52
152	T	14	14	0	0:11.50
153	T	14	14	0	0:11.42
154	T	14	14	0	0:11.48
155	T	14	14	0	0:11.42
156	T	14	14	0	0:11.50
157	T	14	14	0	0:11.47
158	T	14	14	0	0:11.69
159	T	14	14	0	0:11.61
160	T	14	14	0	0:11.51
161	T	14	14	0	0:11.48
162	T	14	14	0	0:11.53
163	T	14	14	0	0:11.49
164	T	14	14	0	0:11.46
165	T	14	14	0	0:11.51
166	T	14	14	0	0:11.53
167	T	14	14	0	0:11.49
168	T	14	14	0	0:11.50
169	T	14	14	0	0:11.50
170	T	14	14	0	0:11.55
171	T	14	14	0	0:11.45
172	T	14	14	0	0:11.50
173	T	14	14	0	0:11.49
174	T	14	14	0	0:11.50
175	T	14	14	0	0:11.59
176	T	14	14	0	0:11.51
177	T	14	14	0	0:11.51
178	T	14	14	0	0:11.47
179	T	14	14	0	0:11.53
180	T	14	14	0	0:11.51
181	T	14	14	0	0:11.43
182	T	14	14	0	0:11.43

Id	Result	#total	#true	#false	Execution time (min:sec)
183	T	14	14	0	0:11.44
184	T	14	14	0	0:11.53
185	T	14	14	0	0:11.55
186	T	14	14	0	0:11.52
187	T	14	14	0	0:11.50
188	T	14	14	0	0:11.51
189	T	14	14	0	0:11.51
190	T	14	14	0	0:11.53
191	T	14	14	0	0:11.45
192	T	14	14	0	0:11.56
193	T	14	14	0	0:11.51
194	T	14	14	0	0:11.51
195	T	14	14	0	0:11.52
196	T	14	14	0	0:11.52
197	T	14	14	0	0:11.60
198	T	14	14	0	0:11.47
199	T	14	14	0	0:11.49
200	T	14	14	0	0:11.52
201	T	14	14	0	0:11.46
202	T	14	14	0	0:11.44
203	T	14	14	0	0:11.51
204	T	14	14	0	0:11.55
205	T	14	14	0	0:11.56
206	T	14	14	0	0:11.61
207	T	14	14	0	0:11.46
208	T	14	14	0	0:11.54
209	T	14	14	0	0:11.64
210	T	14	14	0	0:11.48
211	T	14	14	0	0:11.50
212	T	14	14	0	0:11.48
213	T	14	14	0	0:11.48
214	T	14	14	0	0:11.53
215	T	14	14	0	0:11.50
216	T	14	14	0	0:11.53
217	T	14	14	0	0:11.49
218	T	14	14	0	0:11.45
219	T	14	14	0	0:11.53
220	T	14	14	0	0:11.55
221	T	14	14	0	0:11.55
222	T	14	14	0	0:11.56
223	T	14	14	0	0:11.51
224	T	14	14	0	0:11.48
225	T	14	14	0	0:11.50
226	T	14	14	0	0:11.56
227	T	14	14	0	0:11.53
228	T	14	14	0	0:11.51
229	T	14	14	0	0:11.44
230	T	14	14	0	0:11.47

Id	Result	#total	#true	#false	Execution time (min:sec)
231	T	14	14	0	0:11.50
232	T	14	14	0	0:11.49
233	T	14	14	0	0:11.49
234	T	14	14	0	0:11.48
235	T	14	14	0	0:11.49
236	T	14	14	0	0:11.44
237	T	14	14	0	0:11.56
238	T	14	14	0	0:11.52
239	T	14	14	0	0:11.55
240	T	14	14	0	0:11.47
241	T	14	14	0	0:11.55
242	T	14	14	0	0:11.50
243	T	14	14	0	0:11.43
244	T	14	14	0	0:11.52
245	T	14	14	0	0:11.45
246	T	14	14	0	0:11.52
247	T	14	14	0	0:11.51
248	T	14	14	0	0:11.55
249	T	14	14	0	0:11.46
250	T	14	14	0	0:11.48
251	T	14	14	0	0:11.53
252	T	14	14	0	0:11.47
253	T	14	14	0	0:11.59
254	T	14	14	0	0:11.49
255	T	14	14	0	0:11.53
256	T	14	14	0	0:11.44
257	T	14	14	0	0:11.53
258	T	14	14	0	0:11.50
259	T	14	14	0	0:11.48
260	T	14	14	0	0:11.53
261	T	14	14	0	0:11.50
262	T	14	14	0	0:11.48
263	T	14	14	0	0:11.51
264	T	14	14	0	0:11.51
265	T	14	14	0	0:11.48
266	T	14	14	0	0:11.41
267	T	14	14	0	0:11.45
268	T	14	14	0	0:11.48
269	T	14	14	0	0:11.49
270	T	14	14	0	0:11.52
271	T	14	14	0	0:11.48
272	T	14	14	0	0:11.54
273	T	14	14	0	0:11.61
274	T	14	14	0	0:11.58
275	T	14	14	0	0:11.48
276	T	14	14	0	0:11.51
277	T	14	14	0	0:11.51
278	T	14	14	0	0:11.54

Id	Result	#total	#true	#false	Execution time (min:sec)
279	T	14	14	0	0:11.52
280	T	14	14	0	0:11.51
281	T	14	14	0	0:11.54
282	T	14	14	0	0:11.52
283	T	14	14	0	0:11.53
284	T	14	14	0	0:11.58
285	T	14	14	0	0:11.56
286	T	14	14	0	0:11.50
287	T	14	14	0	0:11.51
288	T	14	14	0	0:11.43
289	T	14	14	0	0:11.57
290	T	14	14	0	0:11.50
291	T	14	14	0	0:11.56
292	T	14	14	0	0:11.45
293	T	14	14	0	0:11.67
294	T	14	14	0	0:11.58
295	T	14	14	0	0:11.54
296	T	14	14	0	0:11.47
297	T	14	14	0	0:11.50
298	T	14	14	0	0:11.61
299	T	14	14	0	0:11.45
300	T	14	14	0	0:11.46
301	T	14	14	0	0:11.48
302	T	14	14	0	0:11.50
303	T	14	14	0	0:11.50
304	T	14	14	0	0:11.54
305	T	14	14	0	0:11.55
306	T	14	14	0	0:11.51
307	T	14	14	0	0:11.50
308	T	14	14	0	0:11.53
309	T	14	14	0	0:11.47
310	T	14	14	0	0:11.55
311	T	14	14	0	0:11.44
312	T	14	14	0	0:11.57
313	T	14	14	0	0:11.47
314	T	14	14	0	0:11.51
315	T	14	14	0	0:11.52
316	T	14	14	0	0:11.51
317	T	14	14	0	0:11.50
318	T	14	14	0	0:11.55
319	T	14	14	0	0:11.45
320	T	14	14	0	0:11.53
321	T	14	14	0	0:11.57
322	T	14	14	0	0:11.48
323	T	14	14	0	0:11.47
324	T	14	14	0	0:11.48
325	T	14	14	0	0:11.52
326	T	14	14	0	0:11.54

Id	Result	#total	#true	#false	Execution time (min:sec)
327	T	14	14	0	0:11.46
328	T	14	14	0	0:11.52
329	T	14	14	0	0:11.53
330	T	14	14	0	0:11.51
331	T	14	14	0	0:11.53
332	T	14	14	0	0:11.54
333	T	14	14	0	0:11.45
334	T	14	14	0	0:11.54
335	T	14	14	0	0:11.52
336	T	14	14	0	0:11.59
337	T	14	14	0	0:11.59
338	T	14	14	0	0:11.46
339	T	14	14	0	0:11.46
340	T	14	14	0	0:11.53
341	T	14	14	0	0:11.57
342	T	14	14	0	0:11.52
343	T	14	14	0	0:11.48
344	T	14	14	0	0:11.45
345	T	14	14	0	0:11.45
346	T	14	14	0	0:11.54
347	T	14	14	0	0:11.53
348	T	14	14	0	0:11.42
349	T	14	14	0	0:11.49
350	T	14	14	0	0:11.57
351	T	14	14	0	0:11.44
352	T	14	14	0	0:11.54
353	T	14	14	0	0:11.50
354	T	14	14	0	0:11.47
355	T	14	14	0	0:11.54
356	T	14	14	0	0:11.49
357	T	14	14	0	0:11.51
358	T	14	14	0	0:11.46
359	T	14	14	0	0:11.56
360	T	14	14	0	0:11.53
361	T	14	14	0	0:11.54
362	T	14	14	0	0:11.49
363	T	14	14	0	0:11.45
364	T	14	14	0	0:11.49
365	T	14	14	0	0:11.49
366	T	14	14	0	0:11.46
367	T	14	14	0	0:11.54
368	T	14	14	0	0:11.57
369	T	14	14	0	0:11.48
370	T	14	14	0	0:11.39
371	T	14	14	0	0:11.40
372	T	14	14	0	0:11.54
373	T	14	14	0	0:11.56
374	T	14	14	0	0:11.47

Id	Result	#total	#true	#false	Execution time (min:sec)
375	T	14	14	0	0:11.50
376	T	14	14	0	0:11.52
377	T	14	14	0	0:11.49
378	T	14	14	0	0:11.50
379	T	14	14	0	0:11.50
380	T	14	14	0	0:11.51
381	T	14	14	0	0:11.46
382	T	14	14	0	0:11.51
383	T	14	14	0	0:11.54
384	T	14	14	0	0:11.60
385	T	14	14	0	0:11.57
386	T	14	14	0	0:11.49
387	T	14	14	0	0:11.58
388	T	14	14	0	0:11.52
389	T	14	14	0	0:11.52
390	T	14	14	0	0:11.41
391	T	14	14	0	0:11.52
392	T	14	14	0	0:12.24
393	T	14	14	0	0:11.51
394	T	14	14	0	0:11.52
395	T	14	14	0	0:11.46
396	T	14	14	0	0:11.47
397	T	14	14	0	0:11.38
398	T	14	14	0	0:11.45
399	T	14	14	0	0:11.49
400	T	14	14	0	0:11.55
401	T	14	14	0	0:11.54
402	T	14	14	0	0:11.48
403	T	14	14	0	0:11.51
404	T	14	14	0	0:11.56
405	T	14	14	0	0:11.48
406	T	14	14	0	0:11.48
407	T	14	14	0	0:11.46
408	T	14	14	0	0:11.42
409	T	14	14	0	0:11.56
410	T	14	14	0	0:11.41
411	T	14	14	0	0:11.47
412	T	14	14	0	0:11.48
413	T	14	14	0	0:11.50
414	T	14	14	0	0:11.51
415	T	14	14	0	0:11.60
416	T	14	14	0	0:11.56
417	T	14	14	0	0:11.45
418	T	14	14	0	0:11.45
419	T	14	14	0	0:11.51
420	T	14	14	0	0:11.50
421	T	14	14	0	0:11.52
422	T	14	14	0	0:11.47

Id	Result	#total	#true	#false	Execution time (min:sec)
423	T	14	14	0	0:11.36
424	T	14	14	0	0:11.55
425	T	14	14	0	0:11.52
426	T	14	14	0	0:11.51
427	T	14	14	0	0:11.51
428	T	14	14	0	0:11.56
429	T	14	14	0	0:11.58
430	T	14	14	0	0:11.46
431	T	14	14	0	0:11.48
432	T	14	14	0	0:11.47
433	T	14	14	0	0:11.49
434	T	14	14	0	0:11.51
435	T	14	14	0	0:11.55
436	T	14	14	0	0:11.47
437	T	14	14	0	0:11.44
438	T	14	14	0	0:11.47
439	T	14	14	0	0:11.45
440	T	14	14	0	0:11.52
441	T	14	14	0	0:11.42
442	T	14	14	0	0:11.45
443	T	14	14	0	0:11.48
444	T	14	14	0	0:11.52
445	T	14	14	0	0:11.45
446	T	14	14	0	0:11.40
447	T	14	14	0	0:11.50
448	T	14	14	0	0:11.50
449	T	14	14	0	0:11.59
450	T	14	14	0	0:11.60
451	T	14	14	0	0:11.50
452	T	14	14	0	0:11.44
453	T	14	14	0	0:11.47
454	T	14	14	0	0:11.49
455	T	14	14	0	0:11.44
456	T	14	14	0	0:11.52
457	T	14	14	0	0:11.55
458	T	14	14	0	0:11.48
459	T	14	14	0	0:11.53
460	T	14	14	0	0:11.52
461	T	14	14	0	0:11.48
462	T	14	14	0	0:11.57
463	T	14	14	0	0:11.57
464	T	14	14	0	0:11.52
465	T	14	14	0	0:11.48
466	T	14	14	0	0:11.47
467	T	14	14	0	0:11.43
468	T	14	14	0	0:11.48
469	T	14	14	0	0:11.58
470	T	14	14	0	0:11.51

Id	Result	#total	#true	#false	Execution time (min:sec)
471	T	14	14	0	0:11.64
472	T	14	14	0	0:11.54
473	T	14	14	0	0:11.48
474	T	14	14	0	0:11.52
475	T	14	14	0	0:11.44
476	T	14	14	0	0:11.50
477	T	14	14	0	0:11.60
478	T	14	14	0	0:11.45
479	T	14	14	0	0:11.53
480	T	14	14	0	0:11.47
481	T	14	14	0	0:11.54
482	T	14	14	0	0:11.50
483	T	14	14	0	0:11.52
484	T	14	14	0	0:11.52
485	T	14	14	0	0:11.53
486	T	14	14	0	0:11.47
487	T	14	14	0	0:11.60
488	T	14	14	0	0:11.57
489	T	14	14	0	0:11.37
490	T	14	14	0	0:11.59
491	T	14	14	0	0:11.54
492	T	14	14	0	0:11.47
493	T	14	14	0	0:11.39
494	T	14	14	0	0:11.46
495	T	14	14	0	0:11.42
496	T	14	14	0	0:11.56
497	T	14	14	0	0:11.45
498	T	14	14	0	0:11.51
499	T	14	14	0	0:11.51
500	T	14	14	0	0:11.52

Table 3: Model checking results corresponding to logic property 11

Id	Result	#total	#true	#false	Execution time (min:sec)
1	T	58	0	58	0:44.89
2	T	58	0	58	0:44.62
3	T	58	0	58	0:44.56
4	T	58	0	58	0:44.66
5	T	58	0	58	0:45.08
6	T	58	0	58	0:45.01
7	T	58	0	58	0:44.62
8	T	58	0	58	0:44.59
9	T	58	0	58	0:44.94
10	T	58	0	58	0:44.77
11	T	58	0	58	0:44.93

Id	Result	#total	#true	#false	Execution time (min:sec)
12	T	58	0	58	0:44.58
13	T	58	0	58	0:44.85
14	T	58	0	58	0:44.67
15	T	58	0	58	0:44.75
16	T	58	0	58	0:44.70
17	T	58	0	58	0:44.65
18	T	58	0	58	0:44.80
19	T	58	0	58	0:44.91
20	T	58	0	58	0:44.86
21	T	58	0	58	0:44.89
22	T	58	0	58	0:44.71
23	T	58	0	58	0:44.64
24	T	58	0	58	0:44.63
25	T	58	0	58	0:44.73
26	T	58	0	58	0:44.83
27	T	58	0	58	0:44.73
28	T	58	0	58	0:44.92
29	T	58	0	58	0:44.84
30	T	58	0	58	0:44.64
31	T	58	0	58	0:44.87
32	T	58	0	58	0:44.78
33	T	58	0	58	0:44.68
34	T	58	0	58	0:44.82
35	T	58	0	58	0:44.74
36	T	58	0	58	0:44.63
37	T	58	0	58	0:45.01
38	T	58	0	58	0:44.77
39	T	58	0	58	0:44.94
40	T	58	0	58	0:44.61
41	T	58	0	58	0:44.79
42	T	58	0	58	0:44.71
43	T	58	0	58	0:44.54
44	T	58	0	58	0:44.77
45	T	58	0	58	0:44.58
46	T	58	0	58	0:44.89
47	T	58	0	58	0:44.99
48	T	58	0	58	0:44.70
49	T	58	0	58	0:44.80
50	T	58	0	58	0:44.80
51	T	58	0	58	0:44.55
52	T	58	0	58	0:44.85
53	T	58	0	58	0:44.64
54	T	58	0	58	0:44.54
55	T	58	0	58	0:44.82
56	T	58	0	58	0:44.73
57	T	58	0	58	0:44.52
58	T	58	0	58	0:44.53
59	T	58	0	58	0:44.65

Id	Result	#total	#true	#false	Execution time (min:sec)
60	T	58	0	58	0:45.17
61	T	58	0	58	0:44.65
62	T	58	0	58	0:44.55
63	T	58	0	58	0:44.63
64	T	58	0	58	0:44.37
65	T	58	0	58	0:44.48
66	T	58	0	58	0:44.92
67	T	58	0	58	0:44.78
68	T	58	0	58	0:44.65
69	T	58	0	58	0:44.61
70	T	58	0	58	0:44.59
71	T	58	0	58	0:44.80
72	T	58	0	58	0:44.75
73	T	58	0	58	0:44.56
74	T	58	0	58	0:44.65
75	T	58	0	58	0:44.78
76	T	58	0	58	0:44.63
77	T	58	0	58	0:44.64
78	T	58	0	58	0:44.62
79	T	58	0	58	0:44.86
80	T	58	0	58	0:44.52
81	T	58	0	58	0:44.51
82	T	58	0	58	0:44.75
83	T	58	0	58	0:44.69
84	T	58	0	58	0:44.72
85	T	58	0	58	0:44.72
86	T	58	0	58	0:44.77
87	T	58	0	58	0:44.72
88	T	58	0	58	0:44.76
89	T	58	0	58	0:44.72
90	T	58	0	58	0:44.62
91	T	58	0	58	0:44.53
92	T	58	0	58	0:44.98
93	T	58	0	58	0:44.81
94	T	58	0	58	0:44.73
95	T	58	0	58	0:44.87
96	T	58	0	58	0:44.86
97	T	58	0	58	0:48.64
98	T	58	0	58	0:44.70
99	T	58	0	58	0:44.88
100	T	58	0	58	0:44.68
101	T	58	0	58	0:44.59
102	T	58	0	58	0:44.81
103	T	58	0	58	0:44.61
104	T	58	0	58	0:44.60
105	T	58	0	58	0:44.67
106	T	58	0	58	0:44.76
107	T	58	0	58	0:44.90

Id	Result	#total	#true	#false	Execution time (min:sec)
108	T	58	0	58	0:44.78
109	T	58	0	58	0:44.80
110	T	58	0	58	0:44.79
111	T	58	0	58	0:44.82
112	T	58	0	58	0:44.64
113	T	58	0	58	0:44.95
114	T	58	0	58	0:44.70
115	T	58	0	58	0:44.73
116	T	58	0	58	0:44.78
117	T	58	0	58	0:44.63
118	T	58	0	58	0:44.66
119	T	58	0	58	0:44.89
120	T	58	0	58	0:44.64
121	T	58	0	58	0:44.61
122	T	58	0	58	0:44.66
123	T	58	0	58	0:44.41
124	T	58	0	58	0:44.68
125	T	58	0	58	0:44.48
126	T	58	0	58	0:44.81
127	T	58	0	58	0:45.70
128	T	58	0	58	0:44.81
129	T	58	0	58	0:44.77
130	T	58	0	58	0:44.45
131	T	58	0	58	0:44.64
132	T	58	0	58	0:44.84
133	T	58	0	58	0:44.58
134	T	58	0	58	0:44.86
135	T	58	0	58	0:44.77
136	T	58	0	58	0:44.46
137	T	58	0	58	0:44.56
138	T	58	0	58	0:44.74
139	T	58	0	58	0:44.80
140	T	58	0	58	0:44.68
141	T	58	0	58	0:44.78
142	T	58	0	58	0:44.81
143	T	58	0	58	0:44.72
144	T	58	0	58	0:45.16
145	T	58	0	58	0:44.65
146	T	58	0	58	0:44.89
147	T	58	0	58	0:44.81
148	T	58	0	58	0:46.90
149	T	58	0	58	0:44.89
150	T	58	0	58	0:44.95
151	T	58	0	58	0:44.68
152	T	58	0	58	0:44.91
153	T	58	0	58	0:44.80
154	T	58	0	58	0:44.67
155	T	58	0	58	0:44.66

Id	Result	#total	#true	#false	Execution time (min:sec)
156	T	58	0	58	0:44.63
157	T	58	0	58	0:44.83
158	T	58	0	58	0:44.62
159	T	58	0	58	0:44.64
160	T	58	0	58	0:44.69
161	T	58	0	58	0:44.71
162	T	58	0	58	0:44.63
163	T	58	0	58	0:44.92
164	T	58	0	58	0:44.72
165	T	58	0	58	0:44.55
166	T	58	0	58	0:44.71
167	T	58	0	58	0:44.79
168	T	58	0	58	0:44.56
169	T	58	0	58	0:44.82
170	T	58	0	58	0:44.68
171	T	58	0	58	0:44.96
172	T	58	0	58	0:44.73
173	T	58	0	58	0:44.87
174	T	58	0	58	0:44.55
175	T	58	0	58	0:44.64
176	T	58	0	58	0:44.58
177	T	58	0	58	0:44.72
178	T	58	0	58	0:45.02
179	T	58	0	58	0:44.82
180	T	58	0	58	0:44.80
181	T	58	0	58	0:44.99
182	T	58	0	58	0:44.71
183	T	58	0	58	0:44.84
184	T	58	0	58	0:44.88
185	T	58	0	58	0:45.09
186	T	58	0	58	0:44.71
187	T	58	0	58	0:44.69
188	T	58	0	58	0:44.65
189	T	58	0	58	0:44.83
190	T	58	0	58	0:44.57
191	T	58	0	58	0:44.88
192	T	58	0	58	0:44.82
193	T	58	0	58	0:44.78
194	T	58	0	58	0:44.74
195	T	58	0	58	0:44.75
196	T	58	0	58	0:44.85
197	T	58	0	58	0:44.69
198	T	58	0	58	0:44.72
199	T	58	0	58	0:44.74
200	T	58	0	58	0:44.67
201	T	58	0	58	0:44.66
202	T	58	0	58	0:44.80
203	T	58	0	58	0:44.87

Id	Result	#total	#true	#false	Execution time (min:sec)
204	T	58	0	58	0:44.72
205	T	58	0	58	0:44.86
206	T	58	0	58	0:44.77
207	T	58	0	58	0:44.91
208	T	58	0	58	0:44.73
209	T	58	0	58	0:44.79
210	T	58	0	58	0:44.80
211	T	58	0	58	0:44.61
212	T	58	0	58	0:45.04
213	T	58	0	58	0:44.74
214	T	58	0	58	0:45.11
215	T	58	0	58	0:44.94
216	T	58	0	58	0:45.00
217	T	58	0	58	0:44.93
218	T	58	0	58	0:44.64
219	T	58	0	58	0:45.00
220	T	58	0	58	0:45.21
221	T	58	0	58	0:44.67
222	T	58	0	58	0:44.80
223	T	58	0	58	0:44.82
224	T	58	0	58	0:44.89
225	T	58	0	58	0:44.69
226	T	58	0	58	0:44.89
227	T	58	0	58	0:44.92
228	T	58	0	58	0:44.88
229	T	58	0	58	0:44.66
230	T	58	0	58	0:44.74
231	T	58	0	58	0:44.97
232	T	58	0	58	0:44.83
233	T	58	0	58	0:44.70
234	T	58	0	58	0:44.79
235	T	58	0	58	0:44.61
236	T	58	0	58	0:44.87
237	T	58	0	58	0:44.99
238	T	58	0	58	0:44.80
239	T	58	0	58	0:44.97
240	T	58	0	58	0:44.75
241	T	58	0	58	0:44.89
242	T	58	0	58	0:44.59
243	T	58	0	58	0:44.80
244	T	58	0	58	0:44.69
245	T	58	0	58	0:44.96
246	T	58	0	58	0:44.63
247	T	58	0	58	0:44.91
248	T	58	0	58	0:44.93
249	T	58	0	58	0:45.24
250	T	58	0	58	0:44.76
251	T	58	0	58	0:44.79

Id	Result	#total	#true	#false	Execution time (min:sec)
252	T	58	0	58	0:44.89
253	T	58	0	58	0:45.04
254	T	58	0	58	0:44.77
255	T	58	0	58	0:44.68
256	T	58	0	58	0:44.58
257	T	58	0	58	0:44.75
258	T	58	0	58	0:44.97
259	T	58	0	58	0:44.84
260	T	58	0	58	0:44.95
261	T	58	0	58	0:44.59
262	T	58	0	58	0:44.77
263	T	58	0	58	0:44.72
264	T	58	0	58	0:44.69
265	T	58	0	58	0:44.76
266	T	58	0	58	0:44.82
267	T	58	0	58	0:44.83
268	T	58	0	58	0:44.73
269	T	58	0	58	0:44.83
270	T	58	0	58	0:44.88
271	T	58	0	58	0:44.84
272	T	58	0	58	0:44.83
273	T	58	0	58	0:44.87
274	T	58	0	58	0:44.81
275	T	58	0	58	0:44.79
276	T	58	0	58	0:44.60
277	T	58	0	58	0:45.20
278	T	58	0	58	0:44.91
279	T	58	0	58	0:44.80
280	T	58	0	58	0:44.78
281	T	58	0	58	0:44.57
282	T	58	0	58	0:44.74
283	T	58	0	58	0:45.07
284	T	58	0	58	0:45.03
285	T	58	0	58	0:45.18
286	T	58	0	58	0:45.01
287	T	58	0	58	0:44.64
288	T	58	0	58	0:44.86
289	T	58	0	58	0:47.69
290	T	58	0	58	0:45.10
291	T	58	0	58	0:44.86
292	T	58	0	58	0:44.80
293	T	58	0	58	0:44.95
294	T	58	0	58	0:44.91
295	T	58	0	58	0:44.86
296	T	58	0	58	0:44.98
297	T	58	0	58	0:44.84
298	T	58	0	58	0:44.75
299	T	58	0	58	0:45.14

Id	Result	#total	#true	#false	Execution time (min:sec)
300	T	58	0	58	0:44.77
301	T	58	0	58	0:44.64
302	T	58	0	58	0:44.79
303	T	58	0	58	0:44.82
304	T	58	0	58	0:44.87
305	T	58	0	58	0:44.80
306	T	58	0	58	0:44.98
307	T	58	0	58	0:45.00
308	T	58	0	58	0:45.00
309	T	58	0	58	0:44.58
310	T	58	0	58	0:44.78
311	T	58	0	58	0:44.89
312	T	58	0	58	0:44.80
313	T	58	0	58	0:45.10
314	T	58	0	58	0:44.85
315	T	58	0	58	0:44.70
316	T	58	0	58	0:45.24
317	T	58	0	58	0:44.72
318	T	58	0	58	0:44.94
319	T	58	0	58	0:44.78
320	T	58	0	58	0:44.83
321	T	58	0	58	0:44.71
322	T	58	0	58	0:44.86
323	T	58	0	58	0:44.87
324	T	58	0	58	0:45.07
325	T	58	0	58	0:44.75
326	T	58	0	58	0:44.94
327	T	58	0	58	0:44.87
328	T	58	0	58	0:44.47
329	T	58	0	58	0:44.84
330	T	58	0	58	0:44.87
331	T	58	0	58	0:48.69
332	T	58	0	58	0:45.29
333	T	58	0	58	0:44.58
334	T	58	0	58	0:44.76
335	T	58	0	58	0:44.57
336	T	58	0	58	0:44.91
337	T	58	0	58	0:44.78
338	T	58	0	58	0:45.13
339	T	58	0	58	0:44.73
340	T	58	0	58	0:44.77
341	T	58	0	58	0:45.00
342	T	58	0	58	0:44.79
343	T	58	0	58	0:44.73
344	T	58	0	58	0:44.94
345	T	58	0	58	0:44.91
346	T	58	0	58	0:45.01
347	T	58	0	58	0:44.99

Id	Result	#total	#true	#false	Execution time (min:sec)
348	T	58	0	58	0:44.80
349	T	58	0	58	0:44.74
350	T	58	0	58	0:44.93
351	T	58	0	58	0:44.94
352	T	58	0	58	0:44.82
353	T	58	0	58	0:45.08
354	T	58	0	58	0:45.03
355	T	58	0	58	0:44.85
356	T	58	0	58	0:44.68
357	T	58	0	58	0:45.17
358	T	58	0	58	0:45.05
359	T	58	0	58	0:44.93
360	T	58	0	58	0:44.79
361	T	58	0	58	0:44.76
362	T	58	0	58	0:44.79
363	T	58	0	58	0:44.92
364	T	58	0	58	0:44.59
365	T	58	0	58	0:45.05
366	T	58	0	58	0:44.77
367	T	58	0	58	0:44.70
368	T	58	0	58	0:44.85
369	T	58	0	58	0:44.82
370	T	58	0	58	0:44.66
371	T	58	0	58	0:44.73
372	T	58	0	58	0:44.60
373	T	58	0	58	0:44.99
374	T	58	0	58	0:44.82
375	T	58	0	58	0:44.93
376	T	58	0	58	0:44.84
377	T	58	0	58	0:45.48
378	T	58	0	58	0:44.99
379	T	58	0	58	0:44.88
380	T	58	0	58	0:44.76
381	T	58	0	58	0:44.85
382	T	58	0	58	0:44.72
383	T	58	0	58	0:44.56
384	T	58	0	58	0:44.70
385	T	58	0	58	0:44.79
386	T	58	0	58	0:44.83
387	T	58	0	58	0:44.90
388	T	58	0	58	0:44.85
389	T	58	0	58	0:45.11
390	T	58	0	58	0:44.89
391	T	58	0	58	0:44.73
392	T	58	0	58	0:45.08
393	T	58	0	58	0:44.88
394	T	58	0	58	0:45.59
395	T	58	0	58	0:45.58

Id	Result	#total	#true	#false	Execution time (min:sec)
396	T	58	0	58	0:45.14
397	T	58	0	58	0:45.03
398	T	58	0	58	0:44.95
399	T	58	0	58	0:44.91
400	T	58	0	58	0:44.83
401	T	58	0	58	0:44.92
402	T	58	0	58	0:44.82
403	T	58	0	58	0:44.87
404	T	58	0	58	0:44.51
405	T	58	0	58	0:44.97
406	T	58	0	58	0:44.94
407	T	58	0	58	0:46.46
408	T	58	0	58	0:45.12
409	T	58	0	58	0:44.82
410	T	58	0	58	0:44.99
411	T	58	0	58	0:44.73
412	T	58	0	58	0:44.75
413	T	58	0	58	0:44.60
414	T	58	0	58	0:44.80
415	T	58	0	58	0:44.93
416	T	58	0	58	0:44.60
417	T	58	0	58	0:44.94
418	T	58	0	58	0:44.94
419	T	58	0	58	0:45.12
420	T	58	0	58	0:44.76
421	T	58	0	58	0:44.86
422	T	58	0	58	0:44.79
423	T	58	0	58	0:44.71
424	T	58	0	58	0:44.60
425	T	58	0	58	0:44.56
426	T	58	0	58	0:46.99
427	T	58	0	58	0:44.85
428	T	58	0	58	0:44.94
429	T	58	0	58	0:44.84
430	T	58	0	58	0:44.90
431	T	58	0	58	0:44.74
432	T	58	0	58	0:44.89
433	T	58	0	58	0:44.89
434	T	58	0	58	0:44.98
435	T	58	0	58	0:45.04
436	T	58	0	58	0:44.85
437	T	58	0	58	0:48.51
438	T	58	0	58	0:44.78
439	T	58	0	58	0:44.62
440	T	58	0	58	0:44.70
441	T	58	0	58	0:44.51
442	T	58	0	58	0:44.81
443	T	58	0	58	0:44.73

Id	Result	#total	#true	#false	Execution time (min:sec)
444	T	58	0	58	0:44.99
445	T	58	0	58	0:44.80
446	T	58	0	58	0:45.05
447	T	58	0	58	0:44.74
448	T	58	0	58	0:44.89
449	T	58	0	58	0:44.73
450	T	58	0	58	0:44.71
451	T	58	0	58	0:44.67
452	T	58	0	58	0:44.98
453	T	58	0	58	0:45.03
454	T	58	0	58	0:44.88
455	T	58	0	58	0:44.89
456	T	58	0	58	0:45.05
457	T	58	0	58	0:44.99
458	T	58	0	58	0:44.94
459	T	58	0	58	0:44.91
460	T	58	0	58	0:45.06
461	T	58	0	58	0:45.01
462	T	58	0	58	0:45.45
463	T	58	0	58	0:44.85
464	T	58	0	58	0:44.94
465	T	58	0	58	0:44.87
466	T	58	0	58	0:45.16
467	T	58	0	58	0:45.07
468	T	58	0	58	0:45.17
469	T	58	0	58	0:45.29
470	T	58	0	58	0:45.03
471	T	58	0	58	0:44.73
472	T	58	0	58	0:45.25
473	T	58	0	58	0:44.68
474	T	58	0	58	0:45.02
475	T	58	0	58	0:44.96
476	T	58	0	58	0:44.92
477	T	58	0	58	0:45.32
478	T	58	0	58	0:45.79
479	T	58	0	58	0:44.98
480	T	58	0	58	0:45.12
481	T	58	0	58	0:45.06
482	T	58	0	58	0:45.10
483	T	58	0	58	0:45.00
484	T	58	0	58	0:45.16
485	T	58	0	58	0:45.65
486	T	58	0	58	0:44.78
487	T	58	0	58	0:44.83
488	T	58	0	58	0:44.96
489	T	58	0	58	0:44.77
490	T	58	0	58	0:45.09
491	T	58	0	58	0:44.84

Id	Result	#total	#true	#false	Execution time (min:sec)
492	T	58	0	58	0:44.94
493	T	58	0	58	0:45.05
494	T	58	0	58	0:45.16
495	T	58	0	58	0:47.79
496	T	58	0	58	0:45.08
497	T	58	0	58	0:48.27
498	T	58	0	58	0:45.13
499	T	58	0	58	0:45.07
500	T	58	0	58	0:44.78

Table 4: Model checking results corresponding to logic property 12

Id	Result	#total	#true	#false	Execution time (min:sec)
1	T	10	9	1	0:08.71
2	T	11	11	0	0:09.51
3	T	10	9	1	0:08.69
4	T	11	11	0	0:09.64
5	T	11	11	0	0:09.41
6	T	11	11	0	0:09.42
7	T	11	11	0	0:09.39
8	T	11	11	0	0:09.46
9	T	11	11	0	0:09.37
10	T	11	11	0	0:09.35
11	T	11	11	0	0:09.45
12	T	11	11	0	0:09.47
13	T	11	11	0	0:09.47
14	T	11	11	0	0:09.39
15	T	11	11	0	0:09.40
16	T	11	11	0	0:09.39
17	T	11	11	0	0:09.39
18	T	11	11	0	0:09.38
19	T	11	11	0	0:09.34
20	T	11	11	0	0:09.40
21	T	11	11	0	0:09.34
22	T	11	11	0	0:09.27
23	T	11	11	0	0:09.31
24	T	11	11	0	0:09.33
25	T	11	11	0	0:09.35
26	T	11	11	0	0:09.34
27	T	11	11	0	0:09.38
28	T	11	11	0	0:09.35
29	T	11	11	0	0:09.36
30	T	11	11	0	0:09.37
31	T	11	11	0	0:09.41
32	T	11	11	0	0:09.36

Id	Result	#total	#true	#false	Execution time (min:sec)
33	T	10	9	1	0:08.63
34	T	11	11	0	0:09.42
35	T	11	11	0	0:09.38
36	T	11	11	0	0:09.33
37	T	11	11	0	0:09.37
38	T	11	11	0	0:09.30
39	T	11	11	0	0:09.44
40	T	11	11	0	0:09.36
41	T	11	11	0	0:09.25
42	T	11	11	0	0:09.32
43	T	11	11	0	0:09.47
44	T	11	11	0	0:09.44
45	T	11	11	0	0:09.35
46	T	11	11	0	0:09.37
47	T	11	11	0	0:09.31
48	T	10	9	1	0:08.56
49	T	11	11	0	0:09.28
50	T	11	11	0	0:09.43
51	T	11	11	0	0:09.38
52	T	11	11	0	0:09.34
53	T	11	11	0	0:09.34
54	T	11	11	0	0:09.25
55	T	11	11	0	0:09.35
56	T	11	11	0	0:09.34
57	T	11	11	0	0:09.29
58	T	10	9	1	0:08.62
59	T	11	11	0	0:09.38
60	T	11	11	0	0:09.29
61	T	11	11	0	0:09.30
62	T	11	11	0	0:09.29
63	T	11	11	0	0:09.41
64	T	11	11	0	0:09.34
65	T	11	11	0	0:09.24
66	T	11	11	0	0:09.43
67	T	11	11	0	0:09.28
68	T	11	11	0	0:09.27
69	T	11	11	0	0:09.24
70	T	11	11	0	0:09.39
71	T	11	11	0	0:09.40
72	T	11	11	0	0:09.38
73	T	11	11	0	0:09.30
74	T	11	11	0	0:09.24
75	T	10	9	1	0:08.50
76	T	11	11	0	0:09.24
77	T	11	11	0	0:09.34
78	T	11	11	0	0:09.38
79	T	11	11	0	0:09.36
80	T	11	11	0	0:09.31

Id	Result	#total	#true	#false	Execution time (min:sec)
81	T	11	11	0	0:09.28
82	T	11	11	0	0:09.39
83	T	11	11	0	0:09.25
84	T	11	11	0	0:09.39
85	T	11	11	0	0:09.29
86	T	11	11	0	0:09.37
87	T	11	11	0	0:09.46
88	T	11	11	0	0:09.31
89	T	11	11	0	0:09.29
90	T	11	11	0	0:09.27
91	T	11	11	0	0:09.31
92	T	11	11	0	0:09.31
93	T	11	11	0	0:09.44
94	T	11	11	0	0:09.46
95	T	11	11	0	0:09.22
96	T	11	11	0	0:09.27
97	T	11	11	0	0:09.23
98	T	11	11	0	0:09.35
99	T	11	11	0	0:09.45
100	T	11	11	0	0:09.35
101	T	11	11	0	0:09.40
102	T	11	11	0	0:09.28
103	T	11	11	0	0:09.95
104	T	11	11	0	0:09.29
105	T	11	11	0	0:09.26
106	T	11	11	0	0:09.21
107	T	11	11	0	0:09.41
108	T	11	11	0	0:09.41
109	T	11	11	0	0:09.14
110	T	11	11	0	0:09.45
111	T	11	11	0	0:09.33
112	T	11	11	0	0:09.12
113	T	11	11	0	0:09.22
114	T	11	11	0	0:09.28
115	T	11	11	0	0:09.33
116	T	11	11	0	0:09.22
117	T	11	11	0	0:09.35
118	T	11	11	0	0:09.34
119	T	11	11	0	0:09.31
120	T	11	11	0	0:09.39
121	T	11	11	0	0:09.39
122	T	10	9	1	0:08.62
123	T	11	11	0	0:09.53
124	T	11	11	0	0:09.30
125	T	10	9	1	0:08.62
126	T	11	11	0	0:09.34
127	T	11	11	0	0:09.43
128	T	11	11	0	0:09.44

Id	Result	#total	#true	#false	Execution time (min:sec)
129	T	11	11	0	0:09.43
130	T	11	11	0	0:09.52
131	T	11	11	0	0:09.52
132	T	11	11	0	0:09.37
133	T	11	11	0	0:09.54
134	T	11	11	0	0:09.42
135	T	11	11	0	0:09.35
136	T	11	11	0	0:09.44
137	T	11	11	0	0:09.45
138	T	11	11	0	0:09.45
139	T	11	11	0	0:09.52
140	T	11	11	0	0:09.51
141	T	11	11	0	0:09.31
142	T	11	11	0	0:09.25
143	T	11	11	0	0:09.50
144	T	11	11	0	0:09.30
145	T	11	11	0	0:09.23
146	T	11	11	0	0:09.41
147	T	11	11	0	0:09.37
148	T	11	11	0	0:09.34
149	T	10	9	1	0:08.66
150	T	11	11	0	0:09.55
151	T	11	11	0	0:09.33
152	T	11	11	0	0:09.40
153	T	11	11	0	0:09.45
154	T	11	11	0	0:09.33
155	T	11	11	0	0:09.53
156	T	11	11	0	0:09.51
157	T	11	11	0	0:09.44
158	T	11	11	0	0:09.42
159	T	11	11	0	0:09.47
160	T	11	11	0	0:09.46
161	T	11	11	0	0:09.44
162	T	11	11	0	0:09.60
163	T	11	11	0	0:09.36
164	T	11	11	0	0:09.69
165	T	11	11	0	0:09.48
166	T	11	11	0	0:09.44
167	T	11	11	0	0:09.33
168	T	11	11	0	0:09.58
169	T	11	11	0	0:09.51
170	T	11	11	0	0:09.44
171	T	11	11	0	0:09.50
172	T	11	11	0	0:09.47
173	T	11	11	0	0:09.39
174	T	11	11	0	0:09.36
175	T	11	11	0	0:09.23
176	T	11	11	0	0:09.23

Id	Result	#total	#true	#false	Execution time (min:sec)
177	T	11	11	0	0:09.22
178	T	11	11	0	0:09.28
179	T	11	11	0	0:09.41
180	T	11	11	0	0:09.32
181	T	11	11	0	0:09.43
182	T	11	11	0	0:09.29
183	T	11	11	0	0:09.39
184	T	11	11	0	0:09.26
185	T	11	11	0	0:09.29
186	T	11	11	0	0:09.31
187	T	11	11	0	0:09.48
188	T	11	11	0	0:09.28
189	T	11	11	0	0:09.43
190	T	11	11	0	0:09.39
191	T	11	11	0	0:09.40
192	T	11	11	0	0:09.34
193	T	11	11	0	0:09.23
194	T	11	11	0	0:09.34
195	T	11	11	0	0:09.30
196	T	11	11	0	0:09.34
197	T	11	11	0	0:09.28
198	T	11	11	0	0:09.31
199	T	11	11	0	0:09.25
200	T	11	11	0	0:09.47
201	T	11	11	0	0:09.24
202	T	11	11	0	0:09.23
203	T	11	11	0	0:09.29
204	T	11	11	0	0:09.32
205	T	11	11	0	0:09.25
206	T	11	11	0	0:09.30
207	T	11	11	0	0:09.28
208	T	11	11	0	0:09.25
209	T	11	11	0	0:09.20
210	T	11	11	0	0:09.23
211	T	11	11	0	0:09.13
212	T	11	11	0	0:09.31
213	T	11	11	0	0:09.25
214	T	11	11	0	0:09.31
215	T	11	11	0	0:09.17
216	T	11	11	0	0:09.35
217	T	11	11	0	0:09.27
218	T	11	11	0	0:09.38
219	T	10	9	1	0:08.54
220	T	11	11	0	0:09.24
221	T	10	9	1	0:08.49
222	T	11	11	0	0:09.15
223	T	11	11	0	0:09.53
224	T	11	11	0	0:09.30

Id	Result	#total	#true	#false	Execution time (min:sec)
225	T	11	11	0	0:09.22
226	T	11	11	0	0:09.25
227	T	11	11	0	0:09.23
228	T	11	11	0	0:09.25
229	T	11	11	0	0:09.20
230	T	11	11	0	0:09.24
231	T	11	11	0	0:09.24
232	T	11	11	0	0:09.28
233	T	11	11	0	0:09.28
234	T	11	11	0	0:09.36
235	T	11	11	0	0:09.45
236	T	11	11	0	0:09.17
237	T	11	11	0	0:09.24
238	T	11	11	0	0:09.38
239	T	11	11	0	0:09.32
240	T	11	11	0	0:09.23
241	T	11	11	0	0:09.26
242	T	11	11	0	0:09.18
243	T	11	11	0	0:09.27
244	T	11	11	0	0:09.22
245	T	11	11	0	0:09.30
246	T	11	11	0	0:09.33
247	T	11	11	0	0:09.34
248	T	11	11	0	0:09.25
249	T	10	9	1	0:08.65
250	T	11	11	0	0:09.47
251	T	11	11	0	0:09.30
252	T	11	11	0	0:09.36
253	T	11	11	0	0:09.23
254	T	11	11	0	0:09.26
255	T	11	11	0	0:09.21
256	T	11	11	0	0:09.24
257	T	11	11	0	0:09.33
258	T	11	11	0	0:09.40
259	T	11	11	0	0:09.54
260	T	11	11	0	0:09.31
261	T	10	9	1	0:08.58
262	T	11	11	0	0:09.32
263	T	11	11	0	0:09.30
264	T	11	11	0	0:09.57
265	T	11	11	0	0:09.25
266	T	11	11	0	0:09.28
267	T	11	11	0	0:09.33
268	T	11	11	0	0:09.26
269	T	11	11	0	0:09.24
270	T	10	9	1	0:08.65
271	T	11	11	0	0:09.35
272	T	11	11	0	0:09.15

Id	Result	#total	#true	#false	Execution time (min:sec)
273	T	11	11	0	0:09.25
274	T	11	11	0	0:09.33
275	T	11	11	0	0:09.26
276	T	11	11	0	0:09.22
277	T	11	11	0	0:09.23
278	T	11	11	0	0:09.21
279	T	11	11	0	0:09.32
280	T	11	11	0	0:09.35
281	T	11	11	0	0:09.27
282	T	11	11	0	0:09.32
283	T	11	11	0	0:09.35
284	T	11	11	0	0:09.51
285	T	11	11	0	0:09.32
286	T	11	11	0	0:09.27
287	T	11	11	0	0:09.32
288	T	11	11	0	0:09.29
289	T	11	11	0	0:09.23
290	T	11	11	0	0:09.34
291	T	11	11	0	0:09.39
292	T	11	11	0	0:09.24
293	T	11	11	0	0:09.24
294	T	11	11	0	0:09.28
295	T	11	11	0	0:09.28
296	T	11	11	0	0:09.12
297	T	11	11	0	0:09.22
298	T	11	11	0	0:09.18
299	T	10	9	1	0:08.57
300	T	10	9	1	0:08.50
301	T	11	11	0	0:09.26
302	T	11	11	0	0:09.30
303	T	11	11	0	0:09.25
304	T	11	11	0	0:09.28
305	T	11	11	0	0:09.22
306	T	11	11	0	0:09.20
307	T	11	11	0	0:09.27
308	T	11	11	0	0:09.33
309	T	11	11	0	0:09.35
310	T	11	11	0	0:09.33
311	T	11	11	0	0:09.21
312	T	11	11	0	0:09.32
313	T	11	11	0	0:09.26
314	T	11	11	0	0:09.29
315	T	11	11	0	0:09.31
316	T	11	11	0	0:09.35
317	T	11	11	0	0:09.23
318	T	11	11	0	0:09.41
319	T	11	11	0	0:09.29
320	T	11	11	0	0:09.25

Id	Result	#total	#true	#false	Execution time (min:sec)
321	T	11	11	0	0:09.32
322	T	11	11	0	0:09.24
323	T	11	11	0	0:09.29
324	T	11	11	0	0:09.37
325	T	11	11	0	0:09.33
326	T	11	11	0	0:09.21
327	T	11	11	0	0:09.17
328	T	11	11	0	0:09.23
329	T	11	11	0	0:09.20
330	T	11	11	0	0:09.38
331	T	11	11	0	0:09.28
332	T	11	11	0	0:09.20
333	T	11	11	0	0:09.36
334	T	11	11	0	0:09.20
335	T	11	11	0	0:09.22
336	T	11	11	0	0:09.31
337	T	11	11	0	0:09.27
338	T	11	11	0	0:09.23
339	T	11	11	0	0:09.29
340	T	10	9	1	0:08.52
341	T	11	11	0	0:09.29
342	T	11	11	0	0:09.25
343	T	11	11	0	0:09.27
344	T	11	11	0	0:09.16
345	T	11	11	0	0:09.22
346	T	11	11	0	0:09.36
347	T	11	11	0	0:09.39
348	T	11	11	0	0:09.26
349	T	11	11	0	0:09.18
350	T	11	11	0	0:09.26
351	T	11	11	0	0:09.17
352	T	11	11	0	0:09.20
353	T	11	11	0	0:09.30
354	T	11	11	0	0:09.22
355	T	11	11	0	0:09.20
356	T	11	11	0	0:09.22
357	T	11	11	0	0:09.31
358	T	11	11	0	0:09.26
359	T	11	11	0	0:09.25
360	T	11	11	0	0:09.15
361	T	11	11	0	0:09.18
362	T	11	11	0	0:09.19
363	T	11	11	0	0:09.22
364	T	11	11	0	0:09.27
365	T	10	9	1	0:08.46
366	T	11	11	0	0:09.28
367	T	11	11	0	0:09.23
368	T	11	11	0	0:09.19

Id	Result	#total	#true	#false	Execution time (min:sec)
369	T	11	11	0	0:09.25
370	T	11	11	0	0:09.22
371	T	11	11	0	0:09.22
372	T	11	11	0	0:09.28
373	T	11	11	0	0:09.22
374	T	11	11	0	0:09.22
375	T	11	11	0	0:09.27
376	T	11	11	0	0:09.26
377	T	11	11	0	0:09.17
378	T	11	11	0	0:09.23
379	T	11	11	0	0:09.28
380	T	11	11	0	0:09.26
381	T	11	11	0	0:09.28
382	T	11	11	0	0:09.24
383	T	11	11	0	0:09.16
384	T	11	11	0	0:09.25
385	T	11	11	0	0:09.26
386	T	11	11	0	0:09.28
387	T	11	11	0	0:09.24
388	T	11	11	0	0:09.18
389	T	11	11	0	0:09.34
390	T	11	11	0	0:09.25
391	T	11	11	0	0:09.23
392	T	11	11	0	0:09.16
393	T	11	11	0	0:09.22
394	T	11	11	0	0:09.19
395	T	11	11	0	0:09.22
396	T	11	11	0	0:09.32
397	T	11	11	0	0:09.28
398	T	11	11	0	0:09.22
399	T	11	11	0	0:09.17
400	T	11	11	0	0:09.51
401	T	11	11	0	0:09.46
402	T	11	11	0	0:09.19
403	T	11	11	0	0:09.17
404	T	11	11	0	0:09.26
405	T	11	11	0	0:09.29
406	T	11	11	0	0:09.27
407	T	11	11	0	0:09.28
408	T	11	11	0	0:09.26
409	T	11	11	0	0:09.28
410	T	11	11	0	0:09.24
411	T	11	11	0	0:09.23
412	T	11	11	0	0:09.21
413	T	11	11	0	0:09.18
414	T	11	11	0	0:09.23
415	T	11	11	0	0:09.31
416	T	11	11	0	0:09.23

Id	Result	#total	#true	#false	Execution time (min:sec)
417	T	11	11	0	0:09.20
418	T	11	11	0	0:09.27
419	T	11	11	0	0:09.10
420	T	11	11	0	0:09.22
421	T	11	11	0	0:09.21
422	T	11	11	0	0:09.22
423	T	11	11	0	0:09.26
424	T	11	11	0	0:09.29
425	T	11	11	0	0:09.19
426	T	11	11	0	0:09.18
427	T	11	11	0	0:09.23
428	T	11	11	0	0:09.22
429	T	11	11	0	0:09.24
430	T	11	11	0	0:09.18
431	T	11	11	0	0:09.15
432	T	11	11	0	0:09.27
433	T	11	11	0	0:09.20
434	T	11	11	0	0:09.24
435	T	11	11	0	0:09.19
436	T	11	11	0	0:09.15
437	T	11	11	0	0:09.21
438	T	11	11	0	0:09.17
439	T	11	11	0	0:09.22
440	T	11	11	0	0:09.25
441	T	11	11	0	0:09.21
442	T	11	11	0	0:09.21
443	T	11	11	0	0:09.33
444	T	11	11	0	0:09.23
445	T	11	11	0	0:09.25
446	T	11	11	0	0:09.21
447	T	11	11	0	0:09.23
448	T	11	11	0	0:09.31
449	T	11	11	0	0:09.36
450	T	11	11	0	0:09.14
451	T	11	11	0	0:09.19
452	T	11	11	0	0:09.19
453	T	11	11	0	0:09.24
454	T	11	11	0	0:09.17
455	T	11	11	0	0:09.26
456	T	11	11	0	0:09.22
457	T	11	11	0	0:09.17
458	T	11	11	0	0:09.13
459	T	11	11	0	0:09.30
460	T	11	11	0	0:09.24
461	T	11	11	0	0:09.28
462	T	11	11	0	0:09.32
463	T	11	11	0	0:09.24
464	T	11	11	0	0:09.18

Id	Result	#total	#true	#false	Execution time (min:sec)
465	T	11	11	0	0:09.27
466	T	11	11	0	0:09.29
467	T	11	11	0	0:09.19
468	T	11	11	0	0:09.20
469	T	11	11	0	0:09.19
470	T	11	11	0	0:09.22
471	T	11	11	0	0:09.28
472	T	11	11	0	0:09.29
473	T	11	11	0	0:09.32
474	T	11	11	0	0:09.24
475	T	11	11	0	0:09.27
476	T	11	11	0	0:09.25
477	T	11	11	0	0:09.24
478	T	11	11	0	0:09.20
479	T	11	11	0	0:09.26
480	T	11	11	0	0:09.22
481	T	11	11	0	0:09.30
482	T	11	11	0	0:09.29
483	T	11	11	0	0:09.28
484	T	11	11	0	0:09.27
485	T	11	11	0	0:09.22
486	T	11	11	0	0:09.29
487	T	11	11	0	0:09.29
488	T	11	11	0	0:09.26
489	T	11	11	0	0:09.26
490	T	11	11	0	0:09.27
491	T	11	11	0	0:09.15
492	T	11	11	0	0:09.29
493	T	11	11	0	0:09.28
494	T	11	11	0	0:09.25
495	T	11	11	0	0:09.19
496	T	11	11	0	0:09.23
497	T	11	11	0	0:09.29
498	T	11	11	0	0:09.33
499	T	11	11	0	0:09.17
500	T	11	11	0	0:09.20

Table 5: Model checking results corresponding to logic property 13

Id	Result	#total	#true	#false	Execution time (min:sec)
1	T	6	5	1	0:05.39
2	T	16	8	8	0:13.04
3	T	10	6	4	0:08.42
4	T	5	4	1	0:04.64
5	T	8	5	3	0:06.84

Id	Result	#total	#true	#false	Execution time (min:sec)
6	F	14	3	11	0:11.49
7	T	6	4	2	0:05.38
8	T	5	4	1	0:04.61
9	T	5	4	1	0:04.63
10	T	23	11	12	0:18.21
11	T	10	6	4	0:08.41
12	T	5	4	1	0:04.61
13	T	11	6	5	0:09.15
14	T	11	6	5	0:09.15
15	F	455	243	212	5:45.09
16	T	5	4	1	0:04.58
17	T	10	6	4	0:08.44
18	T	5	4	1	0:04.56
19	T	8	5	3	0:06.84
20	T	11	6	5	0:09.22
21	T	6	5	1	0:05.36
22	T	11	6	5	0:09.13
23	T	5	4	1	0:04.63
24	T	18	9	9	0:14.54
25	T	5	4	1	0:04.58
26	T	24	11	13	0:19.11
27	T	11	6	5	0:09.14
28	T	5	4	1	0:04.61
29	T	16	8	8	0:12.92
30	T	10	6	4	0:08.36
31	T	13	7	6	0:10.63
32	T	6	6	0	0:05.37
33	T	6	4	2	0:05.35
34	T	19	9	10	0:15.16
35	T	5	4	1	0:04.64
36	T	5	4	1	0:04.64
37	T	13	7	6	0:10.68
38	T	6	4	2	0:05.34
39	T	16	8	8	0:12.92
40	T	11	6	5	0:09.17
41	T	6	5	1	0:05.34
42	T	6	4	2	0:05.38
43	T	5	4	1	0:04.63
44	T	5	4	1	0:04.60
45	T	5	4	1	0:04.64
46	T	5	4	1	0:04.57
47	F	1077	605	472	13:34.64
48	T	6	4	2	0:05.41
49	F	526	288	238	6:38.02
50	F	16	4	12	0:12.94
51	T	18	9	9	0:14.55
52	T	8	5	3	0:06.85
53	T	5	4	1	0:04.60

Id	Result	#total	#true	#false	Execution time (min:sec)
54	T	6	4	2	0:05.33
55	T	10	6	4	0:08.42
56	T	8	5	3	0:06.87
57	T	24	11	13	0:19.04
58	F	10	1	9	0:08.46
59	T	13	7	6	0:10.65
60	T	6	4	2	0:05.33
61	T	6	4	2	0:05.36
62	T	6	4	2	0:05.34
63	T	11	6	5	0:09.10
64	T	5	4	1	0:04.59
65	T	26	12	14	0:20.60
66	T	16	8	8	0:12.82
67	T	6	4	2	0:05.32
68	T	5	4	1	0:04.63
69	T	5	4	1	0:04.59
70	T	8	5	3	0:06.87
71	T	13	7	6	0:10.70
72	T	5	4	1	0:04.68
73	T	6	4	2	0:05.30
74	T	11	6	5	0:09.22
75	T	31	14	17	0:24.28
76	T	6	4	2	0:05.35
77	F	14	3	11	0:11.48
78	T	6	4	2	0:05.32
79	T	16	8	8	0:13.03
80	T	6	4	2	0:05.35
81	T	5	4	1	0:04.63
82	T	10	6	4	0:08.38
83	T	5	4	1	0:04.63
84	T	5	4	1	0:04.57
85	T	16	8	8	0:12.94
86	T	6	4	2	0:05.36
87	T	5	4	1	0:04.59
88	T	8	5	3	0:06.89
89	T	5	4	1	0:04.61
90	T	6	4	2	0:05.33
91	T	15	8	7	0:12.24
92	T	13	7	6	0:10.75
93	T	8	5	3	0:06.79
94	T	24	11	13	0:19.04
95	T	28	13	15	0:22.08
96	T	19	9	10	0:15.22
97	T	21	10	11	0:16.75
98	T	5	4	1	0:04.61
99	T	8	5	3	0:07.40
100	T	11	6	5	0:09.14
101	T	5	4	1	0:04.57

Id	Result	#total	#true	#false	Execution time (min:sec)
102	T	11	6	5	0:09.20
103	T	6	4	2	0:05.35
104	T	6	4	2	0:05.39
105	T	11	6	5	0:09.12
106	T	6	4	2	0:05.35
107	T	5	4	1	0:04.62
108	T	5	4	1	0:04.57
109	T	5	4	1	0:04.62
110	T	10	6	4	0:08.37
111	T	10	6	4	0:08.43
112	T	11	6	5	0:09.15
113	T	6	4	2	0:05.35
114	T	16	8	8	0:13.03
115	T	11	6	5	0:09.22
116	T	6	4	2	0:05.32
117	T	5	4	1	0:04.64
118	T	15	8	7	0:12.14
119	T	6	6	0	0:05.34
120	T	5	4	1	0:04.59
121	T	8	5	3	0:06.86
122	T	10	6	4	0:08.42
123	T	6	4	2	0:05.37
124	T	16	8	8	0:13.00
125	T	19	9	10	0:15.28
126	T	23	11	12	0:18.31
127	T	5	4	1	0:04.62
128	T	5	4	1	0:04.62
129	F	12	2	10	0:09.99
130	T	19	9	10	0:15.21
131	T	8	5	3	0:06.89
132	T	28	13	15	0:22.03
133	T	6	4	2	0:05.40
134	T	6	4	2	0:05.35
135	T	6	4	2	0:05.39
136	T	5	4	1	0:04.61
137	T	5	4	1	0:04.60
138	T	6	4	2	0:05.40
139	T	19	9	10	0:15.13
140	T	18	9	9	0:14.57
141	T	5	4	1	0:04.59
142	T	11	6	5	0:09.18
143	T	5	4	1	0:04.61
144	T	6	4	2	0:05.37
145	T	8	5	3	0:06.89
146	T	5	4	1	0:04.59
147	T	11	6	5	0:09.20
148	T	5	4	1	0:04.64
149	T	6	6	0	0:05.35

Id	Result	#total	#true	#false	Execution time (min:sec)
150	T	24	11	13	0:19.00
151	T	6	4	2	0:05.34
152	T	5	4	1	0:04.63
153	T	16	8	8	0:12.90
154	T	6	4	2	0:05.35
155	T	24	11	13	0:19.08
156	T	6	4	2	0:05.36
157	T	13	7	6	0:10.74
158	T	28	13	15	0:22.11
159	T	5	4	1	0:04.58
160	T	6	6	0	0:05.33
161	F	20	6	14	0:16.00
162	T	11	6	5	0:09.21
163	T	16	8	8	0:12.94
164	T	10	6	4	0:08.39
165	T	5	4	1	0:04.60
166	T	6	6	0	0:05.36
167	T	5	4	1	0:04.63
168	T	5	4	1	0:04.57
169	T	33	15	18	0:25.93
170	F	10	1	9	0:08.37
171	T	19	9	10	0:15.29
172	T	11	6	5	0:09.20
173	T	19	9	10	0:15.30
174	T	10	6	4	0:08.48
175	T	5	4	1	0:04.61
176	T	6	4	2	0:05.42
177	T	5	4	1	0:04.60
178	T	5	4	1	0:04.57
179	T	11	6	5	0:09.17
180	T	5	4	1	0:04.62
181	T	24	11	13	0:19.00
182	T	8	5	3	0:06.88
183	T	5	4	1	0:04.58
184	T	6	4	2	0:05.31
185	T	6	4	2	0:05.40
186	T	29	13	16	0:22.84
187	T	5	4	1	0:04.60
188	T	6	4	2	0:05.40
189	T	5	4	1	0:04.57
190	T	11	6	5	0:09.13
191	T	5	4	1	0:04.62
192	T	5	4	1	0:04.58
193	T	6	4	2	0:05.35
194	T	8	5	3	0:06.88
195	T	8	5	3	0:06.87
196	T	6	4	2	0:05.36
197	T	18	9	9	0:14.42

Id	Result	#total	#true	#false	Execution time (min:sec)
198	F	14	3	11	0:11.43
199	T	10	6	4	0:08.36
200	T	6	4	2	0:05.38
201	T	6	4	2	0:05.38
202	T	10	6	4	0:08.42
203	T	6	4	2	0:05.34
204	T	18	9	9	0:14.46
205	T	6	4	2	0:05.35
206	T	5	4	1	0:04.63
207	T	23	11	12	0:18.36
208	T	10	6	4	0:08.38
209	T	11	6	5	0:09.17
210	T	15	8	7	0:12.19
211	T	11	6	5	0:09.17
212	T	10	6	4	0:08.48
213	T	5	4	1	0:04.63
214	T	6	4	2	0:05.34
215	T	6	6	0	0:05.31
216	T	6	4	2	0:05.37
217	T	5	4	1	0:04.61
218	T	5	4	1	0:04.56
219	T	6	6	0	0:05.34
220	T	6	4	2	0:05.35
221	T	24	11	13	0:19.06
222	T	11	6	5	0:09.19
223	T	6	4	2	0:05.41
224	T	10	6	4	0:08.50
225	T	6	4	2	0:05.35
226	T	5	4	1	0:04.64
227	T	5	4	1	0:04.60
228	T	5	4	1	0:04.61
229	F	41	14	27	0:32.03
230	T	15	8	7	0:12.20
231	T	24	11	13	0:19.14
232	T	6	4	2	0:05.39
233	T	6	4	2	0:05.40
234	T	18	9	9	0:14.52
235	T	8	5	3	0:06.83
236	T	5	4	1	0:04.61
237	T	19	9	10	0:15.22
238	F	18	5	13	0:14.47
239	T	6	4	2	0:05.38
240	T	5	4	1	0:04.59
241	T	5	4	1	0:04.62
242	T	6	4	2	0:05.41
243	T	13	7	6	0:10.63
244	T	13	7	6	0:10.68
245	T	15	8	7	0:12.21

Id	Result	#total	#true	#false	Execution time (min:sec)
246	T	6	4	2	0:05.32
247	T	13	7	6	0:10.68
248	T	13	7	6	0:10.66
249	T	8	5	3	0:07.30
250	T	11	6	5	0:09.16
251	T	6	5	1	0:05.35
252	T	5	4	1	0:04.58
253	T	6	4	2	0:05.39
254	T	21	10	11	0:16.72
255	T	6	4	2	0:05.33
256	T	6	4	2	0:05.36
257	T	8	5	3	0:06.87
258	T	10	6	4	0:08.36
259	T	23	11	12	0:18.30
260	T	8	5	3	0:06.91
261	T	10	6	4	0:08.51
262	T	11	6	5	0:09.20
263	T	5	4	1	0:04.61
264	T	5	4	1	0:04.59
265	T	5	4	1	0:04.58
266	T	11	6	5	0:09.16
267	T	8	5	3	0:06.82
268	T	23	11	12	0:18.25
269	T	6	4	2	0:05.36
270	T	16	8	8	0:12.97
271	T	6	4	2	0:05.34
272	T	5	4	1	0:04.61
273	T	8	5	3	0:06.86
274	T	8	5	3	0:06.86
275	T	6	4	2	0:05.36
276	T	34	15	19	0:26.57
277	T	10	6	4	0:08.40
278	T	15	8	7	0:12.22
279	T	5	4	1	0:04.62
280	T	5	4	1	0:04.57
281	T	5	4	1	0:04.63
282	T	5	4	1	0:04.60
283	T	5	4	1	0:04.61
284	T	11	6	5	0:09.18
285	T	5	4	1	0:04.59
286	T	16	8	8	0:12.96
287	T	13	7	6	0:10.61
288	T	5	4	1	0:04.58
289	T	11	6	5	0:09.15
290	T	21	10	11	0:16.77
291	T	6	4	2	0:05.34
292	T	15	8	7	0:12.18
293	T	11	6	5	0:09.18

Id	Result	#total	#true	#false	Execution time (min:sec)
294	T	5	4	1	0:04.62
295	T	6	5	1	0:05.36
296	T	6	4	2	0:05.43
297	T	6	4	2	0:05.39
298	T	15	8	7	0:12.19
299	T	6	4	2	0:05.37
300	T	10	6	4	0:08.40
301	T	8	5	3	0:06.91
302	T	10	6	4	0:08.41
303	T	6	4	2	0:05.39
304	T	23	11	12	0:18.30
305	T	39	17	22	0:30.26
306	T	6	4	2	0:05.36
307	T	8	5	3	0:06.89
308	T	13	7	6	0:10.68
309	T	6	4	2	0:05.41
310	T	5	4	1	0:04.58
311	T	6	4	2	0:05.39
312	T	13	7	6	0:10.72
313	T	24	11	13	0:18.97
314	T	5	4	1	0:04.58
315	T	16	8	8	0:13.05
316	T	10	6	4	0:08.37
317	T	11	6	5	0:09.19
318	T	13	7	6	0:10.69
319	T	6	4	2	0:05.36
320	T	5	4	1	0:04.60
321	T	6	4	2	0:05.38
322	T	21	10	11	0:16.72
323	T	6	4	2	0:05.34
324	T	8	5	3	0:06.81
325	T	8	5	3	0:06.87
326	T	5	4	1	0:04.60
327	T	26	12	14	0:20.48
328	T	5	4	1	0:04.62
329	T	5	4	1	0:04.65
330	T	44	19	25	0:34.15
331	T	11	6	5	0:09.18
332	T	5	4	1	0:04.59
333	T	5	4	1	0:04.58
334	T	6	6	0	0:05.35
335	T	5	4	1	0:04.59
336	T	5	4	1	0:04.65
337	T	5	4	1	0:04.58
338	T	24	11	13	0:19.13
339	T	16	8	8	0:12.95
340	T	5	4	1	0:04.57
341	T	6	4	2	0:05.37

Id	Result	#total	#true	#false	Execution time (min:sec)
342	T	6	6	0	0:05.35
343	T	6	4	2	0:05.35
344	F	535	293	242	6:46.42
345	T	11	6	5	0:09.16
346	T	6	5	1	0:05.37
347	T	5	4	1	0:04.67
348	T	6	4	2	0:05.33
349	T	6	4	2	0:05.39
350	T	19	9	10	0:15.28
351	T	6	4	2	0:05.36
352	T	8	5	3	0:06.86
353	T	5	4	1	0:04.61
354	T	5	4	1	0:04.60
355	T	5	4	1	0:04.60
356	T	8	5	3	0:06.92
357	T	6	4	2	0:05.38
358	T	10	6	4	0:08.41
359	T	10	6	4	0:08.36
360	T	6	5	1	0:05.34
361	T	5	4	1	0:04.58
362	T	18	9	9	0:14.55
363	T	29	13	16	0:22.94
364	T	13	7	6	0:10.65
365	T	5	4	1	0:04.60
366	T	8	5	3	0:06.89
367	T	15	8	7	0:12.23
368	F	36	14	22	0:27.97
369	T	5	4	1	0:04.56
370	T	6	4	2	0:05.36
371	T	8	5	3	0:06.85
372	T	5	4	1	0:04.59
373	T	5	4	1	0:04.64
374	T	8	5	3	0:06.92
375	T	10	6	4	0:08.47
376	T	18	9	9	0:14.53
377	T	13	7	6	0:10.76
378	F	20	6	14	0:15.97
379	T	6	4	2	0:05.33
380	T	6	6	0	0:05.36
381	T	8	5	3	0:06.89
382	T	6	4	2	0:05.40
383	T	13	7	6	0:10.68
384	T	6	6	0	0:05.37
385	T	11	6	5	0:09.10
386	F	24	8	16	0:18.95
387	T	16	8	8	0:12.97
388	T	13	7	6	0:10.74
389	T	10	6	4	0:08.40

Id	Result	#total	#true	#false	Execution time (min:sec)
390	T	24	11	13	0:19.08
391	T	6	4	2	0:05.41
392	T	19	9	10	0:15.19
393	T	5	4	1	0:04.62
394	T	8	5	3	0:06.84
395	T	5	4	1	0:04.55
396	T	19	9	10	0:15.35
397	T	5	4	1	0:04.61
398	T	6	5	1	0:05.36
399	T	8	5	3	0:06.88
400	T	5	4	1	0:04.61
401	T	16	8	8	0:13.01
402	T	13	7	6	0:10.75
403	T	6	4	2	0:05.36
404	T	6	4	2	0:05.39
405	T	8	5	3	0:06.90
406	T	16	8	8	0:12.91
407	T	16	8	8	0:12.90
408	T	5	4	1	0:04.59
409	T	21	10	11	0:16.84
410	T	8	5	3	0:06.95
411	T	11	6	5	0:09.20
412	T	19	9	10	0:15.27
413	T	21	10	11	0:16.82
414	T	8	5	3	0:06.88
415	T	11	6	5	0:09.17
416	T	13	7	6	0:10.69
417	F	14	3	11	0:11.44
418	T	6	6	0	0:05.35
419	F	12	2	10	0:09.98
420	T	11	6	5	0:09.10
421	T	28	13	15	0:22.03
422	T	11	6	5	0:09.18
423	T	8	5	3	0:06.91
424	T	5	4	1	0:04.58
425	T	29	13	16	0:22.95
426	T	11	6	5	0:09.15
427	T	8	5	3	0:06.83
428	T	5	4	1	0:04.60
429	T	10	6	4	0:08.41
430	T	5	4	1	0:04.61
431	T	6	4	2	0:05.32
432	T	5	4	1	0:04.61
433	T	10	6	4	0:08.39
434	T	10	6	4	0:08.43
435	T	19	9	10	0:15.18
436	T	6	4	2	0:05.36
437	T	5	4	1	0:04.62

Id	Result	#total	#true	#false	Execution time (min:sec)
438	T	16	8	8	0:13.03
439	T	6	4	2	0:05.37
440	T	16	8	8	0:12.99
441	T	6	4	2	0:05.37
442	T	6	5	1	0:05.33
443	T	5	4	1	0:04.60
444	T	8	5	3	0:06.92
445	T	5	4	1	0:04.57
446	F	25	8	17	0:19.77
447	T	6	4	2	0:05.35
448	T	5	4	1	0:04.62
449	T	5	4	1	0:04.60
450	T	10	6	4	0:08.38
451	T	6	4	2	0:05.40
452	T	8	5	3	0:06.86
453	T	5	4	1	0:04.65
454	T	11	6	5	0:09.10
455	F	909	508	401	11:28.53
456	T	8	5	3	0:06.88
457	T	5	4	1	0:04.61
458	T	15	8	7	0:12.24
459	T	28	13	15	0:22.13
460	T	10	6	4	0:08.43
461	T	16	8	8	0:12.91
462	T	6	6	0	0:05.33
463	T	5	4	1	0:04.60
464	T	11	6	5	0:09.15
465	T	6	4	2	0:05.32
466	T	6	4	2	0:05.37
467	T	5	4	1	0:04.64
468	T	6	5	1	0:05.35
469	T	5	4	1	0:04.62
470	T	5	4	1	0:04.62
471	T	5	4	1	0:04.62
472	T	11	6	5	0:09.14
473	T	6	6	0	0:05.40
474	T	5	4	1	0:04.62
475	T	13	7	6	0:10.69
476	F	16	4	12	0:13.02
477	T	6	4	2	0:05.36
478	T	13	7	6	0:10.65
479	T	5	4	1	0:04.57
480	T	5	4	1	0:04.63
481	T	6	6	0	0:05.32
482	T	15	8	7	0:12.28
483	T	15	8	7	0:12.20
484	T	13	7	6	0:10.70
485	T	5	4	1	0:04.58

Id	Result	#total	#true	#false	Execution time (min:sec)
486	T	5	4	1	0:04.62
487	T	15	8	7	0:12.14
488	T	6	4	2	0:05.41
489	T	6	4	2	0:05.35
490	T	6	4	2	0:05.38
491	T	11	6	5	0:09.18
492	T	26	12	14	0:20.60
493	T	5	4	1	0:04.59
494	T	6	4	2	0:05.36
495	T	6	4	2	0:05.40
496	T	8	5	3	0:06.91
497	T	5	4	1	0:04.57
498	T	6	4	2	0:05.38
499	T	21	10	11	0:16.91
500	T	5	4	1	0:04.62

Table 6: Model checking results corresponding to logic property 14

Id	Result	#total	#true	#false	Execution time (min:sec)
1	T	28	0	28	0:22.17
2	T	28	0	28	0:22.08
3	T	28	0	28	0:22.04
4	T	28	0	28	0:21.92
5	T	28	0	28	0:22.07
6	T	28	0	28	0:22.27
7	T	28	0	28	0:22.07
8	T	28	0	28	0:21.97
9	T	28	0	28	0:22.14
10	T	28	0	28	0:22.14
11	T	28	0	28	0:22.18
12	T	28	0	28	0:22.02
13	T	28	0	28	0:22.34
14	T	28	0	28	0:22.13
15	T	28	0	28	0:22.14
16	T	28	0	28	0:22.05
17	T	28	0	28	0:22.14
18	T	28	0	28	0:22.24
19	T	28	0	28	0:23.61
20	T	28	0	28	0:22.07
21	T	28	0	28	0:22.14
22	T	28	0	28	0:22.11
23	T	28	0	28	0:22.05
24	T	28	0	28	0:22.11
25	T	28	0	28	0:22.25
26	T	28	0	28	0:22.02

Id	Result	#total	#true	#false	Execution time (min:sec)
27	T	28	0	28	0:22.13
28	T	28	0	28	0:22.05
29	T	28	0	28	0:22.17
30	T	28	0	28	0:22.26
31	T	28	0	28	0:22.07
32	T	28	0	28	0:22.13
33	T	28	0	28	0:21.90
34	T	28	0	28	0:22.13
35	T	28	0	28	0:22.26
36	T	28	0	28	0:22.09
37	T	28	0	28	0:21.97
38	T	28	0	28	0:22.13
39	T	28	0	28	0:21.87
40	T	28	0	28	0:22.19
41	T	28	0	28	0:22.01
42	T	28	0	28	0:22.12
43	T	28	0	28	0:22.12
44	T	28	0	28	0:21.94
45	T	28	0	28	0:22.05
46	T	28	0	28	0:22.20
47	T	28	0	28	0:22.24
48	T	28	0	28	0:22.15
49	T	28	0	28	0:22.07
50	T	28	0	28	0:22.07
51	T	28	0	28	0:22.21
52	T	28	0	28	0:22.29
53	T	28	0	28	0:22.08
54	T	28	0	28	0:22.06
55	T	28	0	28	0:22.12
56	T	28	0	28	0:22.14
57	T	28	0	28	0:22.02
58	T	28	0	28	0:22.08
59	T	28	0	28	0:21.96
60	T	28	0	28	0:22.22
61	T	28	0	28	0:21.96
62	T	28	0	28	0:23.11
63	T	28	0	28	0:22.25
64	T	28	0	28	0:22.01
65	T	28	0	28	0:22.08
66	T	28	0	28	0:22.19
67	T	28	0	28	0:22.25
68	T	28	0	28	0:22.11
69	T	28	0	28	0:22.07
70	T	28	0	28	0:22.08
71	T	28	0	28	0:22.04
72	T	28	0	28	0:22.11
73	T	28	0	28	0:22.05
74	T	28	0	28	0:22.10

Id	Result	#total	#true	#false	Execution time (min:sec)
75	T	28	0	28	0:22.91
76	T	28	0	28	0:22.09
77	T	28	0	28	0:22.03
78	T	28	0	28	0:22.22
79	T	28	0	28	0:21.98
80	T	28	0	28	0:22.11
81	T	28	0	28	0:22.15
82	T	28	0	28	0:22.03
83	T	28	0	28	0:22.03
84	T	28	0	28	0:22.01
85	T	28	0	28	0:21.99
86	T	28	0	28	0:22.09
87	T	28	0	28	0:22.00
88	T	28	0	28	0:21.97
89	T	28	0	28	0:22.15
90	T	28	0	28	0:24.03
91	T	28	0	28	0:22.01
92	T	28	0	28	0:22.00
93	T	28	0	28	0:21.96
94	T	28	0	28	0:22.03
95	T	28	0	28	0:22.10
96	T	28	0	28	0:22.00
97	T	28	0	28	0:22.08
98	T	28	0	28	0:22.08
99	T	28	0	28	0:22.19
100	T	28	0	28	0:22.19
101	T	28	0	28	0:22.00
102	T	28	0	28	0:22.13
103	T	28	0	28	0:22.25
104	T	28	0	28	0:22.00
105	T	28	0	28	0:22.16
106	T	28	0	28	0:21.96
107	T	28	0	28	0:22.16
108	T	28	0	28	0:22.19
109	T	28	0	28	0:21.94
110	T	28	0	28	0:21.90
111	T	28	0	28	0:22.15
112	T	28	0	28	0:21.96
113	T	28	0	28	0:22.10
114	T	28	0	28	0:22.09
115	T	28	0	28	0:21.99
116	T	28	0	28	0:22.08
117	T	28	0	28	0:22.12
118	T	28	0	28	0:22.04
119	T	28	0	28	0:22.11
120	T	28	0	28	0:22.06
121	T	28	0	28	0:22.06
122	T	28	0	28	0:21.99

Id	Result	#total	#true	#false	Execution time (min:sec)
123	T	28	0	28	0:22.02
124	T	28	0	28	0:22.04
125	T	28	0	28	0:22.13
126	T	28	0	28	0:21.98
127	T	28	0	28	0:22.00
128	T	28	0	28	0:22.08
129	T	28	0	28	0:22.06
130	T	28	0	28	0:21.99
131	T	28	0	28	0:22.01
132	T	28	0	28	0:21.97
133	T	28	0	28	0:22.14
134	T	28	0	28	0:21.96
135	T	28	0	28	0:22.05
136	T	28	0	28	0:22.00
137	T	28	0	28	0:22.07
138	T	28	0	28	0:21.99
139	T	28	0	28	0:21.97
140	T	28	0	28	0:22.13
141	T	28	0	28	0:22.13
142	T	28	0	28	0:22.01
143	T	28	0	28	0:22.09
144	T	28	0	28	0:22.13
145	T	28	0	28	0:23.46
146	T	28	0	28	0:24.23
147	T	28	0	28	0:22.11
148	T	28	0	28	0:22.01
149	T	28	0	28	0:22.09
150	T	28	0	28	0:22.01
151	T	28	0	28	0:22.46
152	T	28	0	28	0:22.20
153	T	28	0	28	0:22.02
154	T	28	0	28	0:22.23
155	T	28	0	28	0:22.17
156	T	28	0	28	0:22.05
157	T	28	0	28	0:22.09
158	T	28	0	28	0:22.14
159	T	28	0	28	0:22.28
160	T	28	0	28	0:22.21
161	T	28	0	28	0:22.15
162	T	28	0	28	0:22.38
163	T	28	0	28	0:22.06
164	T	28	0	28	0:22.25
165	T	28	0	28	0:22.06
166	T	28	0	28	0:22.15
167	T	28	0	28	0:22.01
168	T	28	0	28	0:22.12
169	T	28	0	28	0:22.39
170	T	28	0	28	0:21.98

Id	Result	#total	#true	#false	Execution time (min:sec)
171	T	28	0	28	0:22.20
172	T	28	0	28	0:22.12
173	T	28	0	28	0:22.23
174	T	28	0	28	0:22.37
175	T	28	0	28	0:22.15
176	T	28	0	28	0:22.27
177	T	28	0	28	0:22.14
178	T	28	0	28	0:22.20
179	T	28	0	28	0:22.13
180	T	28	0	28	0:22.01
181	T	28	0	28	0:22.28
182	T	28	0	28	0:22.16
183	T	28	0	28	0:22.08
184	T	28	0	28	0:22.14
185	T	28	0	28	0:22.17
186	T	28	0	28	0:22.11
187	T	28	0	28	0:22.15
188	T	28	0	28	0:22.09
189	T	28	0	28	0:22.18
190	T	28	0	28	0:22.06
191	T	28	0	28	0:22.15
192	T	28	0	28	0:22.21
193	T	28	0	28	0:22.03
194	T	28	0	28	0:22.06
195	T	28	0	28	0:22.09
196	T	28	0	28	0:21.95
197	T	28	0	28	0:23.84
198	T	28	0	28	0:22.14
199	T	28	0	28	0:22.09
200	T	28	0	28	0:22.04
201	T	28	0	28	0:22.05
202	T	28	0	28	0:21.95
203	T	28	0	28	0:22.14
204	T	28	0	28	0:21.92
205	T	28	0	28	0:21.91
206	T	28	0	28	0:21.97
207	T	28	0	28	0:22.06
208	T	28	0	28	0:22.13
209	T	28	0	28	0:22.09
210	T	28	0	28	0:22.15
211	T	28	0	28	0:22.17
212	T	28	0	28	0:22.03
213	T	28	0	28	0:22.06
214	T	28	0	28	0:22.08
215	T	28	0	28	0:21.92
216	T	28	0	28	0:22.10
217	T	28	0	28	0:22.10
218	T	28	0	28	0:22.19

Id	Result	#total	#true	#false	Execution time (min:sec)
219	T	28	0	28	0:22.10
220	T	28	0	28	0:22.03
221	T	28	0	28	0:22.17
222	T	28	0	28	0:22.15
223	T	28	0	28	0:22.00
224	T	28	0	28	0:22.12
225	T	28	0	28	0:22.00
226	T	28	0	28	0:22.08
227	T	28	0	28	0:22.05
228	T	28	0	28	0:22.06
229	T	28	0	28	0:22.08
230	T	28	0	28	0:22.09
231	T	28	0	28	0:22.03
232	T	28	0	28	0:21.96
233	T	28	0	28	0:22.12
234	T	28	0	28	0:22.04
235	T	28	0	28	0:21.99
236	T	28	0	28	0:22.18
237	T	28	0	28	0:22.13
238	T	28	0	28	0:22.12
239	T	28	0	28	0:22.01
240	T	28	0	28	0:21.96
241	T	28	0	28	0:22.19
242	T	28	0	28	0:22.09
243	T	28	0	28	0:22.03
244	T	28	0	28	0:22.23
245	T	28	0	28	0:21.97
246	T	28	0	28	0:22.11
247	T	28	0	28	0:21.96
248	T	28	0	28	0:22.00
249	T	28	0	28	0:22.02
250	T	28	0	28	0:22.25
251	T	28	0	28	0:22.01
252	T	28	0	28	0:22.12
253	T	28	0	28	0:22.18
254	T	28	0	28	0:22.02
255	T	28	0	28	0:22.15
256	T	28	0	28	0:22.07
257	T	28	0	28	0:21.98
258	T	28	0	28	0:22.03
259	T	28	0	28	0:22.15
260	T	28	0	28	0:22.07
261	T	28	0	28	0:21.91
262	T	28	0	28	0:22.06
263	T	28	0	28	0:22.08
264	T	28	0	28	0:22.07
265	T	28	0	28	0:22.07
266	T	28	0	28	0:22.03

Id	Result	#total	#true	#false	Execution time (min:sec)
267	T	28	0	28	0:22.02
268	T	28	0	28	0:22.09
269	T	28	0	28	0:22.21
270	T	28	0	28	0:22.09
271	T	28	0	28	0:22.20
272	T	28	0	28	0:22.09
273	T	28	0	28	0:21.94
274	T	28	0	28	0:22.07
275	T	28	0	28	0:22.10
276	T	28	0	28	0:22.11
277	T	28	0	28	0:22.05
278	T	28	0	28	0:22.20
279	T	28	0	28	0:22.17
280	T	28	0	28	0:22.13
281	T	28	0	28	0:21.98
282	T	28	0	28	0:22.06
283	T	28	0	28	0:21.98
284	T	28	0	28	0:22.02
285	T	28	0	28	0:22.11
286	T	28	0	28	0:22.01
287	T	28	0	28	0:22.10
288	T	28	0	28	0:22.09
289	T	28	0	28	0:21.98
290	T	28	0	28	0:21.97
291	T	28	0	28	0:22.14
292	T	28	0	28	0:22.21
293	T	28	0	28	0:22.09
294	T	28	0	28	0:22.17
295	T	28	0	28	0:22.20
296	T	28	0	28	0:22.22
297	T	28	0	28	0:22.07
298	T	28	0	28	0:22.06
299	T	28	0	28	0:22.10
300	T	28	0	28	0:22.15
301	T	28	0	28	0:22.04
302	T	28	0	28	0:22.00
303	T	28	0	28	0:22.24
304	T	28	0	28	0:22.28
305	T	28	0	28	0:22.30
306	T	28	0	28	0:22.10
307	T	28	0	28	0:22.07
308	T	28	0	28	0:22.12
309	T	28	0	28	0:22.15
310	T	28	0	28	0:22.14
311	T	28	0	28	0:21.96
312	T	28	0	28	0:22.13
313	T	28	0	28	0:21.95
314	T	28	0	28	0:22.41

Id	Result	#total	#true	#false	Execution time (min:sec)
315	T	28	0	28	0:22.32
316	T	28	0	28	0:22.03
317	T	28	0	28	0:22.18
318	T	28	0	28	0:22.04
319	T	28	0	28	0:22.09
320	T	28	0	28	0:22.14
321	T	28	0	28	0:22.16
322	T	28	0	28	0:21.93
323	T	28	0	28	0:21.96
324	T	28	0	28	0:22.11
325	T	28	0	28	0:22.07
326	T	28	0	28	0:22.00
327	T	28	0	28	0:22.02
328	T	28	0	28	0:22.02
329	T	28	0	28	0:21.94
330	T	28	0	28	0:22.01
331	T	28	0	28	0:22.11
332	T	28	0	28	0:22.26
333	T	28	0	28	0:22.02
334	T	28	0	28	0:22.15
335	T	28	0	28	0:22.15
336	T	28	0	28	0:22.15
337	T	28	0	28	0:22.07
338	T	28	0	28	0:22.02
339	T	28	0	28	0:22.10
340	T	28	0	28	0:22.07
341	T	28	0	28	0:22.13
342	T	28	0	28	0:21.98
343	T	28	0	28	0:22.08
344	T	28	0	28	0:22.14
345	T	28	0	28	0:22.06
346	T	28	0	28	0:21.95
347	T	28	0	28	0:22.10
348	T	28	0	28	0:22.13
349	T	28	0	28	0:22.19
350	T	28	0	28	0:22.09
351	T	28	0	28	0:22.08
352	T	28	0	28	0:22.10
353	T	28	0	28	0:22.02
354	T	28	0	28	0:21.98
355	T	28	0	28	0:22.01
356	T	28	0	28	0:22.23
357	T	28	0	28	0:22.05
358	T	28	0	28	0:22.02
359	T	28	0	28	0:21.99
360	T	28	0	28	0:22.21
361	T	28	0	28	0:22.04
362	T	28	0	28	0:22.09

Id	Result	#total	#true	#false	Execution time (min:sec)
363	T	28	0	28	0:22.03
364	T	28	0	28	0:22.12
365	T	28	0	28	0:22.11
366	T	28	0	28	0:22.07
367	T	28	0	28	0:22.07
368	T	28	0	28	0:22.05
369	T	28	0	28	0:22.07
370	T	28	0	28	0:22.02
371	T	28	0	28	0:22.17
372	T	28	0	28	0:21.97
373	T	28	0	28	0:22.00
374	T	28	0	28	0:22.12
375	T	28	0	28	0:22.08
376	T	28	0	28	0:22.05
377	T	28	0	28	0:22.04
378	T	28	0	28	0:21.90
379	T	28	0	28	0:22.02
380	T	28	0	28	0:21.97
381	T	28	0	28	0:22.09
382	T	28	0	28	0:22.21
383	T	28	0	28	0:22.00
384	T	28	0	28	0:22.22
385	T	28	0	28	0:21.96
386	T	28	0	28	0:22.18
387	T	28	0	28	0:21.96
388	T	28	0	28	0:22.01
389	T	28	0	28	0:22.05
390	T	28	0	28	0:22.03
391	T	28	0	28	0:22.14
392	T	28	0	28	0:21.84
393	T	28	0	28	0:22.22
394	T	28	0	28	0:21.99
395	T	28	0	28	0:22.10
396	T	28	0	28	0:22.05
397	T	28	0	28	0:22.05
398	T	28	0	28	0:22.17
399	T	28	0	28	0:22.29
400	T	28	0	28	0:22.06
401	T	28	0	28	0:22.10
402	T	28	0	28	0:22.08
403	T	28	0	28	0:22.07
404	T	28	0	28	0:22.07
405	T	28	0	28	0:22.19
406	T	28	0	28	0:21.99
407	T	28	0	28	0:22.13
408	T	28	0	28	0:22.16
409	T	28	0	28	0:22.01
410	T	28	0	28	0:22.13

Id	Result	#total	#true	#false	Execution time (min:sec)
411	T	28	0	28	0:22.00
412	T	28	0	28	0:22.16
413	T	28	0	28	0:22.02
414	T	28	0	28	0:22.17
415	T	28	0	28	0:22.05
416	T	28	0	28	0:22.07
417	T	28	0	28	0:22.16
418	T	28	0	28	0:22.14
419	T	28	0	28	0:22.04
420	T	28	0	28	0:22.03
421	T	28	0	28	0:21.90
422	T	28	0	28	0:21.99
423	T	28	0	28	0:22.01
424	T	28	0	28	0:22.04
425	T	28	0	28	0:22.12
426	T	28	0	28	0:22.12
427	T	28	0	28	0:21.98
428	T	28	0	28	0:21.94
429	T	28	0	28	0:21.96
430	T	28	0	28	0:22.07
431	T	28	0	28	0:22.02
432	T	28	0	28	0:22.06
433	T	28	0	28	0:22.10
434	T	28	0	28	0:22.05
435	T	28	0	28	0:22.02
436	T	28	0	28	0:22.05
437	T	28	0	28	0:21.95
438	T	28	0	28	0:22.01
439	T	28	0	28	0:22.00
440	T	28	0	28	0:21.97
441	T	28	0	28	0:21.91
442	T	28	0	28	0:22.16
443	T	28	0	28	0:22.11
444	T	28	0	28	0:22.07
445	T	28	0	28	0:22.09
446	T	28	0	28	0:22.01
447	T	28	0	28	0:22.09
448	T	28	0	28	0:22.09
449	T	28	0	28	0:22.15
450	T	28	0	28	0:22.28
451	T	28	0	28	0:22.06
452	T	28	0	28	0:21.92
453	T	28	0	28	0:22.08
454	T	28	0	28	0:22.09
455	T	28	0	28	0:21.92
456	T	28	0	28	0:22.20
457	T	28	0	28	0:22.07
458	T	28	0	28	0:22.06

Id	Result	#total	#true	#false	Execution time (min:sec)
459	T	28	0	28	0:22.01
460	T	28	0	28	0:21.81
461	T	28	0	28	0:22.04
462	T	28	0	28	0:22.01
463	T	28	0	28	0:22.12
464	T	28	0	28	0:22.02
465	T	28	0	28	0:22.07
466	T	28	0	28	0:22.19
467	T	28	0	28	0:22.03
468	T	28	0	28	0:22.11
469	T	28	0	28	0:22.02
470	T	28	0	28	0:22.03
471	T	28	0	28	0:22.07
472	T	28	0	28	0:21.93
473	T	28	0	28	0:21.86
474	T	28	0	28	0:22.27
475	T	28	0	28	0:22.16
476	T	28	0	28	0:22.06
477	T	28	0	28	0:22.10
478	T	28	0	28	0:22.07
479	T	28	0	28	0:22.00
480	T	28	0	28	0:22.11
481	T	28	0	28	0:22.12
482	T	28	0	28	0:22.02
483	T	28	0	28	0:21.87
484	T	28	0	28	0:22.07
485	T	28	0	28	0:22.16
486	T	28	0	28	0:22.13
487	T	28	0	28	0:21.98
488	T	28	0	28	0:22.04
489	T	28	0	28	0:22.08
490	T	28	0	28	0:22.11
491	T	28	0	28	0:22.07
492	T	28	0	28	0:22.07
493	T	28	0	28	0:22.05
494	T	28	0	28	0:22.02
495	T	28	0	28	0:22.36
496	T	28	0	28	0:22.02
497	T	28	0	28	0:22.12
498	T	28	0	28	0:21.99
499	T	28	0	28	0:22.01
500	T	28	0	28	0:22.03
